# Supplementary material for: C5 methylation confers accessibility, stability and selectivity to picrotoxinin
Source: Nat Commun. 2023 Dec 14;14:8308. doi: 10.1038/s41467-023-44030-3 (PMC10721898; doi:10.1038/s41467-023-44030-3)
Supplement: Supplementary file 1 — Supplementary Information [file 41467_2023_44030_MOESM1_ESM.pdf]

Supplementary Information

C5 Methylation Confers Accessibility, Stability and Selectivity to Picrotoxinin

Guanghu Tong,[1] Samantha Griffin,[2] Avery Sader,[2] Anna B. Crowell,[3] Ken Beavers,[2] Jerry Watson,[2] Zachary Buchan,[2] Shuming Chen,[3,*] Ryan A. Shenvi[1,*]

[1]Department of Chemistry, Scripps Research, 10550 North Torrey Pines Road, La Jolla, California 92037, United States.

[2]Corteva Agriscience, 9330 Zionsville Road, Indianapolis, Indiana 46268, United States.

[3]Department of Chemistry and Biochemistry, Oberlin College, 119 Woodland Street, Oberlin, Ohio 44074, United States.

[*]Correspondence to: shuming.chen@oberlin.edu, rshenvi@scripps.edu

Table of Contents

1. Materials and methods S4
2. Experimental procedures and characterization data S5
 2.1 5MeBrPXN (9) S13
 2.2 PTN (10) S14
 2.3 5MePTN (11) S14
 2.4 12FPXN (12) S14
 2.5 12F5MePXN (13) S15
 2.6 CypPXN (14) S16
 2.7 Cyp5MePXN (15) S16
 2.8 12MePXN (16) S17
 2.9 12Me5MePXN (17) S18
 2.10 8,9doPXN (18) S18
 2.11 8,9do5MePXN (19) S19
 2.12 HPXN (20) S20
 2.13 H5MePXN (21) S20
 2.14 6FPXN (22) S21
 2.15 6F5MePXN (23) S21
 2.16 6FPTN (24) S22
 2.17 6F5MePTN (25) S23
 2.18 6FHPXN (26) S23
 2.19 6FH5MePXN (27) S24
 2.20 6F12FPXN (28) S25
 2.21 6F12F5MePXN (30) S25
3. Stability studies S28
 3.1 Methanolysis of PXN under stoichiometric amount of NaOMe in MeOH S28
 3.2 Methanolysis of PXN under catalytic amount of NaOMe in CD_3OD S29
 3.3 Competitive methanolysis of PXN and 5MePXN under 5 mol% NaOMe in CD_3OD. S32
 3.4 Methanolysis of 5MePXN under different equivalent of NaOMe in CD_3OD S34
 3.5 Competitive degradation of PXN and 5MePXN under dilute acidic solution S35
 3.6 Stability of PXN and 5MePXN in mouse and human plasma. S35
4. Rat $GABA_A$ receptors binding assay S37
5. RDL receptors binding assay S39
6. Computational models for stability S40

6.1 Computational methods S40
6.2 Calculated energies S40
7. Computational models for RDL selectivity S42
8. NMR spectra S43
9. X-ray crystallographic data S116
5MePTN (11) (CCDC 2048201) S116
12F5MePXN (13) (CCDC 2081332) S123
CypPXN (14) (CCDC 2071501) S130
HPXN (20) (CCDC 2048202) S144
H5MePXN (21) (CCDC 2048203) S151
6F5MePXN (23) (CCDC 2069870) S158
6F12F5MePXN (30) (CCDC 2079783) S165
Alkene (31) (CCDC 2078168) S172
d_9-34 (CCDC 2087432) S179
10. References S186

1. Materials and methods

Hexane, dichloromethane (DCM), toluene, ethyl acetate (EtOAc), diethyl ether (Et_2O), tetrahydrofuran (THF), acetone, dimethylsulfoxide (DMSO), methanol (MeOH), isopropanol (*i*-PrOH), *N*-dimethylformamide (DMF), Acetonitrile (MeCN) and triethylamine (Et_3N) were purchased from Sigma Aldrich, Fisher Chemicals or Acros Organics and used without further purification. All anhydrous solvents were purchased from Fisher Chemicals, Sigma Aldrich or Acros Organics and used without further purification, unless otherwise stated. Reactions were monitored by thin layer chromatography (TLC) with pre-coated silica gel plates from EMD Chemicals (TLC Silica gel 60 F254, 250 μm thickness) using UV light as the visualizing agent and an acidic mixture of anisaldehyde, phosphomolybdic acid (PMA), chromic acid, iodine vapor, Seebach's stain, or basic aqueous potassium permanganate ($KMnO_4$), and heat as developing agents. Preparatory thin layer chromatography (PTLC) was performed using the aforementioned silica gel plates. Flash column chromatography was performed over silica gel 60 (particle size 0.035- 0.07 mm) from Acros Organics. NMR spectra were recorded on Bruker DRX-600 (equipped with a 5mm DCH Cryoprobe), AV-600, DRX-500 or DPX-400 and calibrated using residual non-deuterated solvent as an internal reference ($CHCl_3$ @ 7.26 ppm ^1H NMR, 77.16 ppm ^{13}C NMR; $(CD_3)_2CO$ @ 2.05 ^1H NMR, 206.26 ^{13}C NMR; CD_3OD @ 3.31 ^1H NMR, 49.00 ^{13}C NMR). The following abbreviations (or combinations thereof) were used to explain the multiplicities: s = singlet, d = doublet, t = triplet, q = quartet, p = pentet, sex = sextet, sep = septet m = multiplet, br = broad. LC/MS analysis was performed on an Agilent 1200 series HPLC/MS equipped with an Agilent SB-C18 2.1 mm x 50 mm column, with mass spectra recorded on a 6120 Quadrupole mass spectrometer (API-ES), using MeCN and H_2O as the mobile phase (0.1% formic acid). LC-MS runs used the following method unless otherwise specified: flow rate of 0.5 mL / min is used, initial equilibration of 5% MeCN / H_2O with a linear gradient to 95% MeCN / H_2O over 5 minutes, then a hold at 95% MeCN / H_2O for an additional 3 minutes. Optical rotations were measured digitally on an Autopol III polarimeter from Rudolph Research Analytic, using a flow cell with a 0.5 decimeter pathlength and the sodium lamp D-line wavelength (λ=589.3 nm). High resolution mass spectrometric data were obtained on a Waters Xevo G2-XS QTOF instrument. Commercial picrotoxinin was obtained from Millipore Sigma (USA) and used for relative analog preparation.

Unless otherwise noted, all experiments were run in flame-dried glassware under an atmosphere of argon. A stir bar is always present in the reaction vessel.

2. Experimental procedures and characterization data

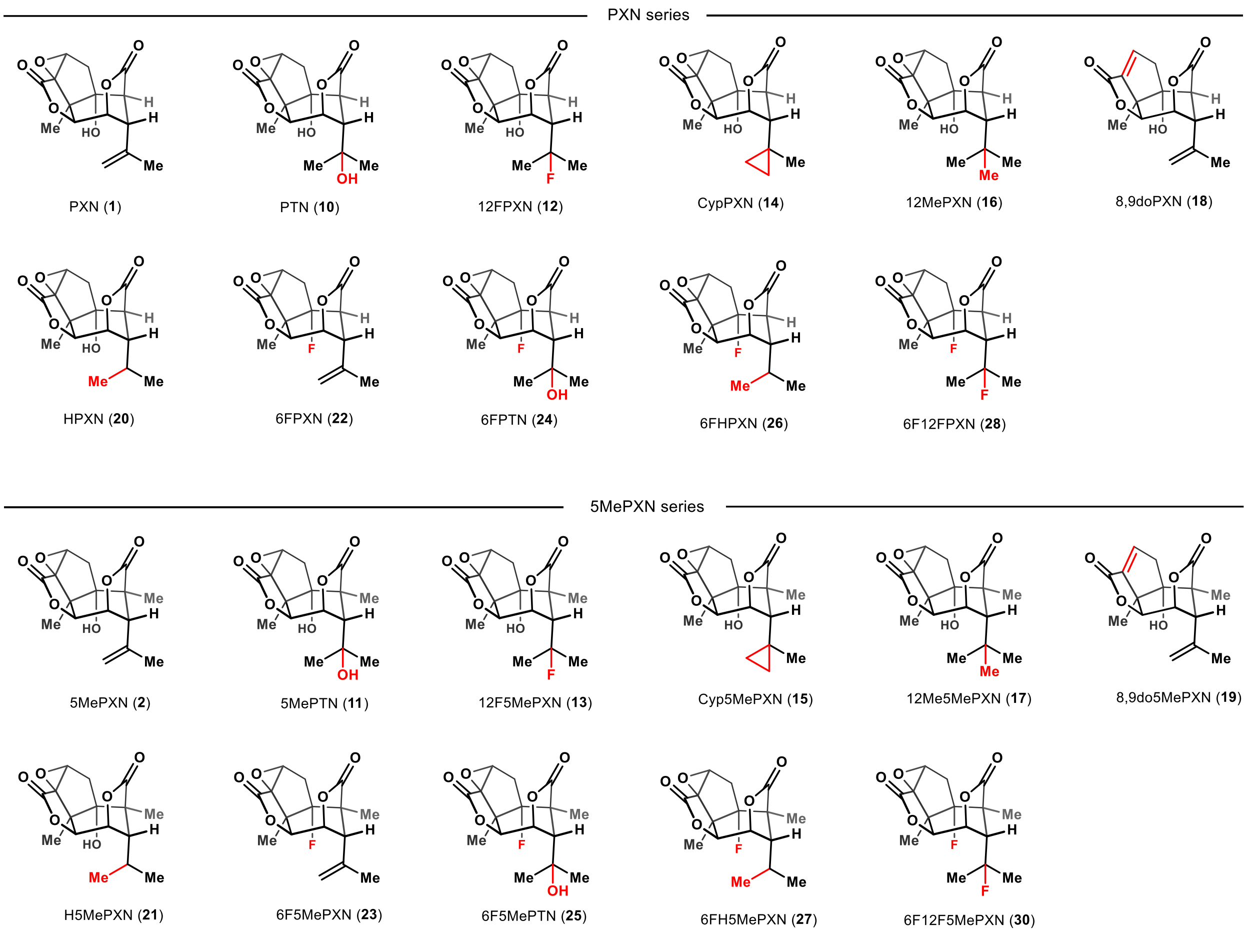

Supplementary Figure 1. List of synthetic and test compounds in this work.

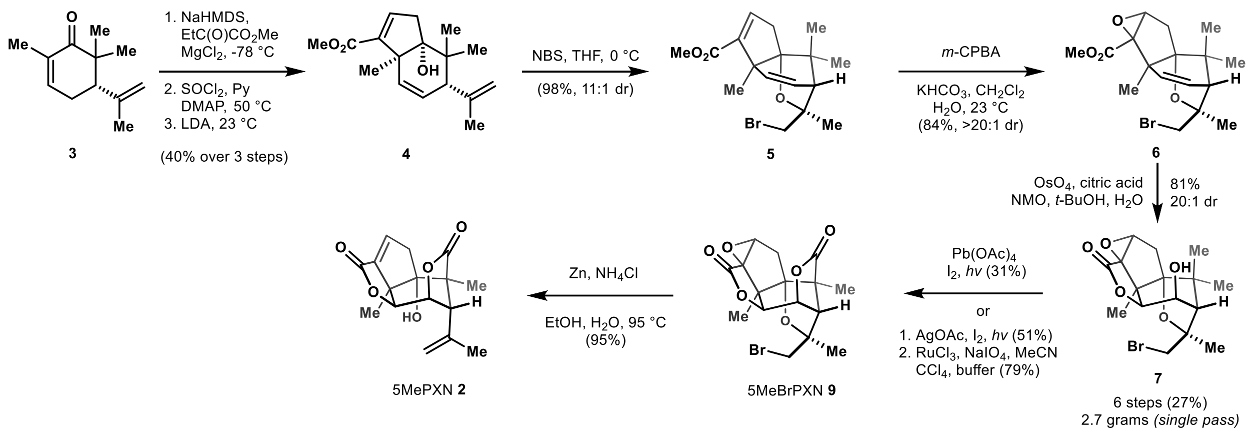

Supplementary Figure 2. 5MePXN (**2**) was synthesized from dimethylcarvone (**3**).[1]

Procedure:

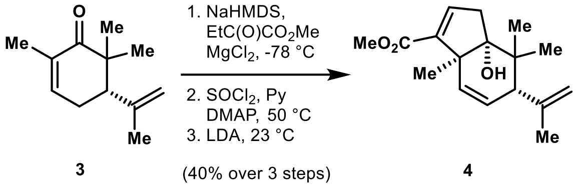

To a flame-dried round bottom flask charged with anhydrous $MgCl_2$ (1.9 g, 20.0 mmol, 2 equiv, NOTE 1) was added a solution of freshly distilled dimethylcarvone **3** (1.78 g, 10.0 mmol, 1 equiv) in anhydrous THF (100 mL) at 23 ºC for 20 minutes. NaHMDS (1 M in THF, 15 mL, 15.0 mmol, 1.5 equiv) was added to the above mixture at -78 ºC. The reaction was vigorously stirred for an additional 30 minutes at -78 ºC. The solution was placed in a 0 ºC ice/water bath and stirred for 60 minutes at 0 ºC. Once the enolate was fully formed (NOTE 2), the solution was cooled back to -78 ºC and methyl-2-oxobutanoate (3.35 mL, 30.0 mmol, 3.0 equiv) was added neat to the enolate solution in a steady stream within a period of 5 minutes. After 75 minutes, the mixture was quenched by addition of saturated NH_4Cl/H_2O (1:1, v:v, 20 mL) at -78 ºC (NOTE 3). The reaction was then warmed to room temperature and extracted with EtOAc (3 x 150 mL). The organic layer was washed with brine, dried over Na_2SO_4, filtered, and concentrated *in vacuo*. The crude residue was purified by flash column chromatography over silica gel (5% EtOAc/hex to 10% EtOAc/hex). The desired aldol addition products were collected together (1.91 g, 6.7 mmol, 67%, dr ~ 3.3:1 at C-9, NOTE 4) as a pale-yellow oil.

NOTE 1: $MgCl_2$ powder (weighed out assuming it is anhydrous) is further dried by heating with a heat gun under high vacuum until no more ‘bubbling’ of the powder occurs. The vessel is then placed under an argon atmosphere and other reagents are added to this reaction vessel.

NOTE 2: After 60 minutes, it is useful to TLC the enolate solution to confirm that the enolate has fully formed. Protonation at C-1 occurs upon TLC analysis to give the deconjugated isomer of dimethyl-carvone, which is less polar than dimethyl-carvone and not UV-active. Disappearance of the dimethyl-carvone spot is observed when the enolate has fully formed.

NOTE 3: The reaction must be quenched at -78 ºC, because the retro-aldol reaction of the product occurs at warmer temperatures (*ca* >~ -20 ºC).

NOTE 4: The yield of this reaction between different runs ranged from 60-70% and the diastereoselectivity between 2-3.3:1 at the C-9 alcohol. The opposite diastereomer at C-1 was never observed. The C-9 diastereomers

can be collected separately after chromatographic separation. A 2.2:1 diastereomeric mixture at C-9 were separated and used for characterization of the two C-9 diastereomers.

Characterization data of aldol addition adduct (major diastereomer):

Description	Faint yellow/colorless oil. Became a white waxy crystalline solid upon storage at -20 ºC.
R_f	0.23 in 15% EtOAc/hex. Weakly UV active. Stain purple in anisaldehyde.
Opt. Rot.	α_{obs} = +58.9º, c=1.00 in CH_2Cl_2, T=25.6 ºC
^{1}H NMR	(600 MHz, Chloroform-*d*)<br>δ 5.91 (dd, *J* = 10.3, 2.4 Hz, 1H), 5.80 (dd, *J* = 10.3, 3.1 Hz, 1H), 5.05 (dq, *J* = 2.0, 1.3 Hz, 1H), 4.77 (dt, *J* = 1.7, 0.8 Hz, 1H), 3.87 (s, 3H), 3.76 (s, 1H), 3.15 (t, *J* = 2.8 Hz, 1H), 1.98 (dq, *J* = 13.7, 7.3 Hz, 1H), 1.81 (dd, *J* = 1.5, 0.8 Hz, 3H), 1.67 (dq, *J* = 13.6, 7.4 Hz, 1H), 1.34 (s, 3H), 1.11 (s, 3H), 1.02 (s, 3H), 0.76 (t, *J* = 7.3 Hz, 3H).
^{13}C NMR	(151 MHz, Chloroform-*d*)<br>δ 218.4, 176.1, 144.7, 132.9, 130.6, 115.9, 83.3, 55.0, 53.0, 50.2, 48.4, 26.6, 24.7, 23.1, 23.0, 22.0, 7.6.
HRMS	Calculated $C_{17}H_{27}O_4$ [M+H]: 295.1909 | Found: 295.1906

Characterization data of aldol addition adduct (minor diastereomer):

Description	Faint yellow/colorless oil. Became a white waxy crystalline solid upon extended storage at -20 ºC.
R_f	0.3 in 15% EtOAc/hex. Weakly UV active. Stain purple in anisaldehyde.
Opt. Rot.	α_{obs} = -45.9º, c=1.00 in CH_2Cl_2, T=24.2 ºC
^{1}H NMR	(600 MHz, Chloroform-*d*)<br>δ 5.86 (dd, *J* = 10.3, 2.3 Hz, 1H), 5.52 (dd, *J* = 10.4, 3.2 Hz, 1H), 5.11 – 4.98 (m, 1H), 4.75 (dq, *J* = 1.7, 0.8 Hz, 1H), 4.09 (d, *J* = 1.6 Hz, 1H), 3.76 (s, 3H), 3.18 (t, *J* = 2.8 Hz, 1H), 2.11 (dq, *J* = 14.6, 7.3 Hz, 1H), 1.90 (dq, *J* = 14.7, 7.4 Hz, 1H), 1.84 – 1.78 (m, 3H), 1.32 (s, 3H), 1.13 (s, 3H), 0.99 (s, 3H), 0.79 (t, *J* = 7.3 Hz, 3H).
^{13}C NMR	(151 MHz, Chloroform-*d*)<br>δ 218.5, 175.2, 144.7, 133.1, 130.4, 116.0, 84.3, 52.7, 52.4, 50.2, 48.5, 26.0, 24.5, 23.2, 22.6, 22.4, 8.1.
HRMS	Calculated $C_{17}H_{27}O_4$ [M+H]: 295.1909 | Found: 295.1905

Two separate solutions were made: First, a solution of above diastereomeric (3.3:1 d.r.) substrate (1.47 g, 5.0 mmol, 1.0 equiv), DMAP (3.05 g, 25.0 mmol, 5.0 equiv), and non-anhydrous MeCN (50 mL, [0.1 M] with respect to substrate) was made in a 250 mL round bottom flask. Second, a solution of $SOCl_2$ (725 µL, 10.0 mmol, 2 equiv) in anhydrous pyridine (10 mL, [1 M] with respect to $SOCl_2$) under an argon atmosphere was made. The substrate/DMAP/MeCN solution was slowly added $SOCl_2$/pyr solution with vigorous stirring at 50 ºC (oil bath). The reaction was monitored by TLC. After 45 minutes, the resulting orange solution was cooled to room temperature, then poured onto a 1:1 mixture of H_2O:EtOAc (50 mL each). The aqueous layer was extracted with EtOAc (3 x 50 mL). The combined organic layer was washed with 1 M HCl (20 mL), saturated $NaHCO_3$ (20 mL), and brine. The combined organic layer was dried over Na_2SO_4, filtered and concentrated *in vacuo*. The crude residue was purified by flash column chromatography over silica gel (5% EtOAc/hex to 10% EtOAc/hex) to obtain corresponding enone (926 mg, 3.35 mmol, 67%) as a clear yellow oil.

Characterization data**:**

Description	viscous yellow oil
R_f	0.52 in 10% EtOAc/hex. UV active. Stains purple in anisaldehyde.
Opt. Rot.	α_{obs} = -289.5º, c=1.00 in CH_2Cl_2, T=22.3 ºC
^{1}H NMR	(600 MHz, Chloroform-*d*) δ 5.84 (q, *J* = 7.1 Hz, 1H), 5.69 (dd, *J* = 10.1, 3.8 Hz, 1H), 5.66 (dd, *J* = 10.3, 0.7 Hz, 1H), 4.88 – 4.84 (m, 1H), 4.76 – 4.72 (m, 1H), 3.72 (s, 3H), 3.08 (dd, *J* = 3.7, 0.7 Hz, 1H), 1.72 (d, *J* = 7.1 Hz, 3H), 1.57 – 1.55 (m, 3H), 1.37 (s, 3H), 1.22 (s, 3H), 1.00 (s, 3H).
^{13}C NMR	(151 MHz, Chloroform-*d*) δ 214.5, 168.7, 144.5, 137.3, 131.5, 129.4, 129.0, 115.4, 57.7, 51.6, 50.5, 47.5, 27.6, 26.4, 22.8, 21.2, 15.5.
HRMS	Calculated $C_{17}H_{25}O_3$ [M+H] 277.1804 | Found: 277.1810

A [0.5M] solution of LDA in THF was made by addition of *n*BuLi ([2.67M in hex], 12.0 mmol, 4.5 mL) to a solution of HN^iPr_2 (freshly distilled off CaH_2, 15.0 mmol, 2.10 mL) in anhydrous THF (17.4 mL) while cooled to -78 ºC under an argon atmosphere. After addition of *n*BuLi, the solution was stirred at 0 ºC for 20 minutes, then cooled back to -78 ºC.

A solution of above enone (>99% purity, 2.49 mmol, 687 mg) in anhydrous THF under an argon atmosphere in a flame-dried 100 mL round bottom flask was cooled to 0 ºC. The LDA solution ([0.5M], 1.1 equiv., 5.5 mL) was added in a slow but steady stream via syringe to this solution at 0 ºC, which caused the solution to turn from clear and pale yellow to clear and orange/red. The solution stirred at 0 ºC for 30 minutes, then warmed to 23 ºC and monitored by TLC (20% EtOAc/hex, Anis.). Starting material still remained after 3 hours, so 100 µL more [0.5 M] LDA was added dropwise at 23 ºC to this solution. TLC analysis indicated full consumption of starting material 1 hour after this (4 hours after the initial LDA addition).

The reaction was quenched by addition of NH_4Cl (aq., saturated, ~20 mL) and dilution with EtOAc (~ 20 mL). The reaction was extracted with EtOAc (3x ~ 20 mL). The organic layer was dried over $MgSO_4$, filtered, and concentrated *in vacuo*. The crude mass (860 mg) was purified on silica gel (~ 250 mL) with 2L of 10% EtOAc/hex. The desired compound **4** was isolated as a yellow-white solid. (620 mg, 90% yield; see NOTE 1).

NOTE 1: The yield drops significantly if the substrate is impure. The excess LDA solution is maintained at -78 ºC for the duration of the intramolecular aldol reaction. Use of a larger excess of LDA can lead to transposition of the α,β-unsaturated ester alkene and incorporation of molecular oxygen, so reaction monitoring by TLC and subsequent addition of small portions of LDA to drive the reaction to completion is preferable to use of more than 1.1 equivalents of LDA at the start of the reaction.

Characterization data of intramolecular aldol product **4:**

Description	yellow-white solid
R_f	0.52 in 10% EtOAc/hex. UV active and stains purple in anisaldehyde
Opt. Rot.	α_{obs} = -27.6º, c=1.00 in CH_2Cl_2, T=21.0 ºC
^{1}H NMR	(600 MHz, Chloroform-*d*)

δ 6.67 (br dd, *J* = 3.2, 2.0 1H), 5.98 (br d, *J* = 10.3 Hz, 1H), 5.32 (br d, *J* = 10.3 Hz, 1H), 5.02 – 4.99 (br m, 1H), 4.73 (br s, 1H), 3.72 (s, 3H), 2.93 (br s, 1H), 2.92 (br d, *J* = 18.5 Hz, 1H), 2.31 (dd, *J* = 18.9, 3.2 Hz, 1H), 1.79 (s, 3H), 1.71 (br s, 1H), 1.42 (s, 3H), 1.06 (s, 3H), 1.00 (s, 3H).

^{13}C NMR (151 MHz, Chloroform-*d*)

δ 165.1, 145.8, 141.0, 140.6, 133.6, 126.7, 116.0, 85.4, 53.3, 52.1, 51.4, 42.6, 40.3, 24.0, 23.3, 18.8, 18.5.

HRMS Calculated $C_{17}H_{25}O_3$ [M+H]: 277.1804 | Found: 277.1810

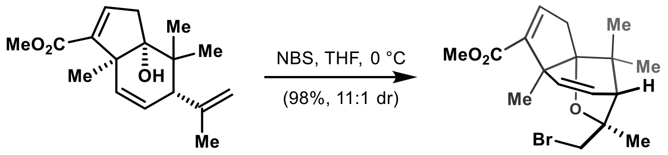

4 5

A flame-dried 50 mL round bottom flask was charged with solid triene substrate **4** (1.0 equiv, 610 mg, 2.21 mmol) and a stir bar and placed under an argon atmosphere at 0 ºC in an ice-water bath. A separate flame-dried 50 mL round bottom flask was charged with NBS (recrystallized, 2.0 equiv, 4.42 mmol, 787 mg) and anhydrous THF ([0.2 M], wrt NBS) at 0 ºC in an ice-water bath and kept out of light (wrapped in foil and hood light turned off). Without stirring (See NOTE 1), the NBS/THF solution was then transferred by cannula (see NOTE 2) with differential pressure under an Argon atmosphere at 0 ºC. The solution after transfer was clear and pale yellow. The reaction was stirred at 0 ºC in the dark (fume hood lights off and the reaction flask was wrapped in foil) and monitored by TLC (15% EtOAc/hex, Anis.). No starting material remained after 2 hours.

The reaction was worked-up by addition to a mixture of NH_4Cl (saturated, aq., ~50 mL) and EtOAc (~50 mL). The aqueous layer was extracted with EtOAc (3x ~ 100 mL) and then washed with $NaHCO_3$ (saturated, 1x ~ 50 mL). The organic layer was dried over $MgSO_4$, filtered, and concentrated *in vacuo*. The crude mass (~1.2 g) was dry-loaded with celite onto a silica column (~100 mL of silica) and purified by flash column chromatography with 15% EtOAc/hex. The desired compound **5** was isolated as a yellow-tinged white solid. (776 mg, 98% yield, 11:1 diastereoselectivity in favor of the endo diastereomer shown above.). Isolated yields for this reaction ranged between 92 – 98% for different runs.

NOTE 1: Dissolution of the substrate in THF prior to addition of the NBS/THF solution diminishes the diastereoselectivity of the reaction. Although the diastereoselectivity of this step is technically inconsequential because the stereocenter is erased by the final zinc reduction step, low diastereoselectivity at this step complicates analysis and characterization of the products of subsequent steps.

NOTE 2: The cannula was cooled during transfer by contact with solid dry ice.

Characterization data of bromoetherification product **5:**

Description yellow-white powder. Can be obtained as white crystalline material by recrystallization

R_f 0.37 in 10% EtOAc/hx. UV active and stains purple in anisaldehyde

Opt. Rot. α_{obs} = -14.7º, c=1.00 in CH_2Cl_2, T=21.5 ºC (on 11:1 diastereomeric mixture)

^{1}H NMR (600 MHz, Chloroform-*d*)

δ 6.53 (dd, *J* = 3.5, 2.2 Hz, 1H), 6.25 (d, *J* = 9.6 Hz, 1H), 5.97 (dd, *J* = 9.6, 6.9 Hz, 1H), 3.72 (s, 3H), 3.66 (dd, *J* = 9.2, 1.1 Hz, 1H), 3.40 (d, *J* = 9.2 Hz, 1H), 2.59 (ddd, *J* = 18.1, 2.2, 0.7 Hz, 1H), 2.46 (ddd, *J* = 18.2, 3.6, 0.8 Hz, 1H), 2.21 (d, *J* = 6.9 Hz, 1H), 1.65 (d, *J* = 1.0 Hz, 3H), 1.29 (s, 3H), 1.23 (s, 3H), 0.98 (s, 3H).

^{13}C NMR (151 MHz, Chloroform-*d*)

δ 164.3, 142.7, 139.1, 132.7, 128.0, 95.9, 85.2, 52.9, 52.8, 51.4, 46.2, 42.9, 32.1, 27.2, 27.0, 26.2, 20.6.

HRMS Calculated $C_{17}H_{24}BrO_3$ [M+H] 355.0909 | Found: 355.0907

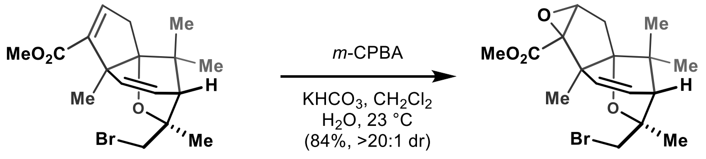

5 6

To a suspension of substrate **5** (2.5 g, 7.0 mmol, 1 equiv) in CH_2Cl_2/H_2O (5:1, v:v, 120 mL) was added $KHCO_3$ (5.6 g, 56.0 mmol, 8 equiv) and stirred vigorously for 15 min. The biphasic reaction mixture was then cooled to 0 ºC and *m*CPBA (50% purity, 9.66 g, 28.0 mmol, 4 equiv) was slowly added portion-wise. The solution was stirred vigorously for 2 hours at room temperature, during which time a white precipitate formed. TLC indicated starting material consumption. The reaction was quenched by slowly adding a saturated $Na_2S_2O_3$ solution (aq., 20 mL) at 0ºC, then diluted by H_2O (100 mL) and CH_2Cl_2 (200 mL). The aqueous layer was extracted with CH_2Cl_2 (3 x 100 mL). The combined organics were dried over Na_2SO_4, filtered, and concentrated *in vacuo*. The crude product was purified by column chromatography over silica gel (5% EtOAc/Hexane) to afford epoxide **6** (2.18 g, 5.88 mmol, 84%) as a single diastereomer.

Characterization data of epoxidation product **6:**

Description yellow-white powder. Can be obtained as white crystalline material by recrystallization

R_f 0.28 in 10% EtOAc/hex. Stains purple in anisaldehyde.

Opt. Rot. α_{obs} = -36.0º, c=1.00 in CH_2Cl_2, T=22.0 ºC

^{1}H NMR (600 MHz, Chloroform-*d*)

δ 6.20 (dd, *J* = 9.6, 0.7 Hz, 1H), 6.02 (dd, *J* = 9.6, 6.9 Hz, 1H), 3.75 (dd, *J* = 4.1, 1.0 Hz, 1H), 3.71 (s, 3H), 3.60 (dd, *J* = 9.2, 1.1 Hz, 1H), 3.31 (d, *J* = 9.2 Hz, 1H), 2.23 (d, *J* = 6.9 Hz, 1H), 2.12 (ddd, *J* = 14.6, 4.2, 0.9 Hz, 1H), 1.84 (dt, *J* = 14.6, 1.0 Hz, 1H), 1.61 (d, *J* = 1.1 Hz, 3H), 1.29 (s, 4H), 1.16 (s, 3H), 1.14 (s, 3H).

^{13}C NMR (151 MHz, Chloroform-*d*)

δ 168.1, 133.1, 127.6, 99.4, 85.9, 66.0, 61.5, 52.5, 52.0 (observed by HSQC), 48.2, 46.6, 42.6, 29.3, 28.1, 26.2, 24.2, 17.1.

HRMS Calculated $C_{17}H_{24}BrO_4$ [M+H]: 371.0858 | Found: 371.0856

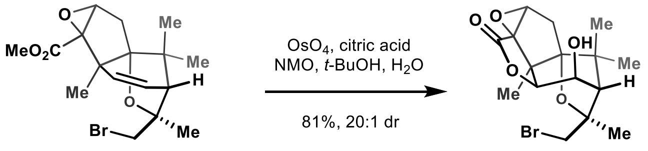

6 7

A 1 L round bottom flask was charged with a magnetic stir bar, the alkene substrate **6** (3.31 g, 8.92 mmol, 1 equiv), citric acid (3.43 g, 17.84 mmol, 2 equiv), NMO (3.13 g, 26.76 mmol, 3 equiv), and *t*-BuOH (57 mL). During this time, the fume hood lights were turned off and the reaction was wrapped with aluminum foil to minimize exposure to ambient light. OsO_4 (57 mL, 4 wt% in H_2O, 9.37 mmol, 1.05 equiv, NOTE 1) was then added at room temperature in the dark. The reaction was capped with a yellow cap (a 24/40 polyethylene flask stopper), sealed with duct tape, and stirred vigorously (>1000 rpm) for 7 days at room temperature. During the course of the reaction, the yellow cap was stained black by osmium tetroxide. The reaction was quenched by adding a saturated solution of $Na_2S_2O_3$ (20 mL) after cooling the reaction to 0 ºC. After stirring for 15 min, the dark yellow solution changed to black suspension, hood lamps were turned on, the reaction mixture was diluted with EtOAc (200 mL), then extracted with EtOAc (3 x 300 mL). The combined organic layer was dried over Na_2SO_4, filtered, and concentrated *in vacuo*. The crude mixture was purified by column chromatography over silica gel (30% EtOAc/Hexane to 50% EtOAc/Hexane) to afford desired dihydroxylation product **7** (2.7 g, 7.23 mmol, 81%, 89% brsm) as single diastereoisomer. Starting material **6** (298 mg, 0.8 mmol, 9%) was recovered.

NOTE 1: OsO_4 4 wt% in H_2O is a commercially available greenish solution. It may also be made from OsO_4 solid in deionized H_2O. OsO_4 is a highly toxic volatile solid so use of an efficient fume hood is strongly recommended for this procedure.

Characterization data of dihydroxylation product **7:**

Description	white foam/powder
R_f	0.41 in 50% EtOAc/Hex. Anis. Stains dark blue/purple.
Opt. Rot.	α_{obs} = 2.4º, c=1.00 in CH_2Cl_2, T=20.7 ºC
^{1}H NMR	(600 MHz, Chloroform-*d*)
	δ 4.78 (d, *J* = 8.8 Hz, 1H), 4.70 (ddd, *J* = 8.9, 3.5, 1.5 Hz, 1H), 3.89 (dd, *J* = 3.9, 1.6 Hz, 1H), 3.35 (d, *J* = 10.4 Hz, 1H), 3.28 (d, *J* = 10.4 Hz, 1H), 2.23 (dd, *J* = 14.6, 3.9 Hz), 2.24 – 2.21 (m, 2H), 1.96 (ddd, *J* = 14.6, 1.6, 0.9 Hz, 1H), 1.65 (s, 3H), 1.31 (s, 3H), 1.25 (s, 3H), 1.23 (s, 3H).
^{13}C NMR	(151 MHz, Chloroform-*d*)
	δ 170.7, 100.6, 82.4, 79.4, 68.4, 67.7, 63.1, 56.9, 44.3, 44.1, 40.9, 31.8, 29.8, 27.5, 22.7, 18.7.
HRMS	Calculated $C_{16}H_{22}BrO_5$ [M+H]: 373.0651 | Found: 373.0646

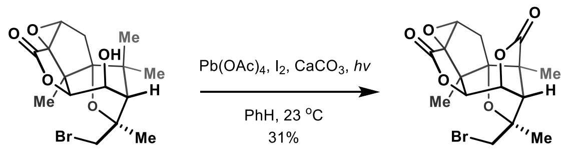

7 5MeBrPXN **9**

To a solution of $Pb(OAc)_4$ (592 mg, 1.34 mmol, 5 equiv, NOTE 1) in benzene (10 mL) was added I_2 (340 mg, 1.34 mmol, 5 equiv) in the dark (covered with aluminum foil and hood light turned-off) at room temperature and stirred for 30 min. To a suspension of **7** (100 mg, 0.27 mmol, 1 equiv) and $CaCO_3$ (270 mg, 2.7 mmol, 10 equiv) in benzene (5 mL) was added the $Pb(OAc)_4/I_2$/benzene solution at room temperature. The hood lamps were turned on and the reaction was vigorously stirred at room temperature under ambient light for 2.5 h. Consumption of **7** was monitored by TLC. The reaction was quenched by saturated $Na_2S_2O_3$ (5 mL) at 0 ºC, then filtered through a short pad of Celite, washed with EtOAc (3 x 10 mL). The combined organic layer was dried over

Na_2SO_4, filtered, and concentrated *in vacuo*. The crude product was purified by column chromatography over silica gel (50% EtOAc/Hexane) to afford 5MeBrPXN **9** (32 mg, 0.084 mmol, 31%) as a white solid.

NOTE 1: Commercially available $Pb(OAc)_4$ is stabilized with AcOH, pure $Pb(OAc)_4$ could be freshly recrystallized from AcOH, then washed three times with hexane prior to use.

Characterization data of 5MeBrPXN **9:**

Description clear to white amphorous solid

R_f 0.51 in 50% EtOAc/hex. Stains weakly grey/green in anisaldehyde.

Opt. Rot. α_{obs} = -17.0º, c=0.50 in CH_2Cl_2, T=20.0 ºC

^{1}H NMR (600 MHz, Chloroform-*d*)
δ 5.22 (t, *J* = 5.3 Hz, 1H), 4.76 (d, *J* = 5.2 Hz, 1H), 3.96 (dd, *J* = 3.6, 1.5 Hz, 1H), 3.52 (dd, *J* = 10.6, 1.2 Hz, 1H), 3.41 (d, *J* = 10.7 Hz, 1H), 3.16 (dd, *J* = 5.5, 0.9 Hz, 1H), 2.46 (dd, *J* = 14.4, 3.6 Hz, 1H), 1.94 (dt, *J* = 14.4, 1.3 Hz, 1H), 1.61 (d, *J* = 1.0 Hz, 3H), 1.56 (s, 3H), 1.36 (s, 3H).

^{13}C NMR (151 MHz, Chloroform-*d*)
δ 173.6, 168.3, 97.3, 84.2, 78.3, 75.8, 67.7, 63.3, 60.5, 53.9, 47.8, 36.8, 33.9, 26.5, 19.7, 17.4.

HRMS Calculated $C_{16}H_{18}BrO_6$ [M+H]: 385.0287 | Found: 385.0279

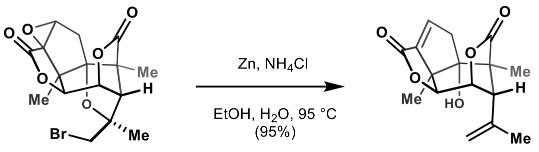

5MeBrPXN **9** 5MePXN **2**

Zinc powder (55 mg, 0.84 mmol, 10 equiv), NH_4Cl (90 mg, 1.68 mmol, 20 equiv) were added into a solution of 5MeBrPXN **9** (32 mg, 0.084 mmol, 1 equiv) in EtOH/H_2O (v:v, 10:1, 6.6 mL). Then the reaction was warmed up to 95 ºC and stirred at this temperature for 2 h. The reaction mixture was diluted by EtOAc (5 mL) then filtered through a short pad of Celite and washed with EtOAc (3 x 3 mL). The reaction mixture was concentrated and purified by column chromatography over silica gel (10% EtOAc/CH_2Cl_2) to give 5MePXN **2** (24.4 mg, 0.08 mmol, 95%) as a white solid.

Characterization data of 5MePXN **2**:

Description white crystalline solid

R_f 0.31 in 40% EtOAc/hex. Stains brown in anisaldehyde.

Opt. Rot. α_{obs} = -1.2º, c=0.10 in CH_2Cl_2, T=20.0 ºC

^{1}H NMR (600 MHz, Chloroform-*d*)
δ 5.11 (p, *J* = 1.6 Hz, 1H), 5.05 (d, *J* = 2.1 Hz, 1H), 5.02 (dd, *J* = 5.2, 3.4 Hz, 1H), 4.83 (d, *J* = 3.4 Hz, 1H), 3.70 (dd, *J* = 3.6, 0.7 Hz, 1H), 3.14 (d, *J* = 5.1 Hz, 1H), 2.97 (dd, *J* = 15.3, 3.6 Hz, 1H), 1.96 (dt, *J* = 1.6, 0.9 Hz, 3H), 1.88 (s, 1H), 1.75 (d, *J* = 15.3 Hz, 1H), 1.53 (s, 3H), 1.26 (s, 3H).

^{13}C NMR (151 MHz, Chloroform-*d*)
δ 177.2, 168.9, 139.4, 115.6, 88.7, 80.2, 76.1, 72.6, 62.0, 53.9, 51.0, 47.4, 42.5, 24.3, 17.1, 17.0.

HRMS Calculated $C_{16}H_{19}O_6$ [M+H]: 307.1182 | Found: 307.1178

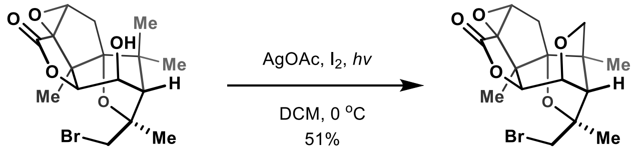

7 **S1**

Two reactions were carried out in parallel. To a suspension of alcohol **7** (2 x (50 mg, 0.13 mmol, 1 equiv)) and AgOAc (2 x (109 mg, 0.65 mmol, 5 equiv)) in CH_2Cl_2 (2 x 5 mL) was added I_2 (2 x (165 mg, 0.65 mmol, 5 equiv) as solid at 0ºC. The reaction was vigorously stirred under ambient light for 1 hour, then quenched with saturated $Na_2S_2O_3$ (aq., 0.5 mL each) at 0 ºC. The two reactions were combined, filtered through a short pad of celite and washed with CH_2Cl_2 (3 x 10 mL). The crude product was concentrated *in vacuo* and then purified by column chromatography over silica gel (5% EtOAc/CH_2Cl_2 to 10% EtOAc/CH_2Cl_2) to give ether **S1** (49 mg, 0.13 mmol, 51%) as colorless foam.

Characterization data of ether **S1**:

Description	colorless oil/foam
R_f	0.51 in 10% EtOAc/CH_2Cl_2. Stains dark blue in anisaldehyde.
Opt. Rot.	α_{obs} = -64.3º, c=2.00 in CH_2Cl_2, T=20.0 ºC
^{1}H NMR	(600 MHz, $CDCl_3$)<br>δ 4.75 (dd, *J* = 5.8 Hz, *J* = 4.9 Hz, 1H), 4.63 (d, *J* = 5.8 Hz, 1H), 4.02 (d, *J* = 11.1 Hz, 1H), 3.93 (dd, *J* = 3.8, 1.5 Hz, 1H), 3.71 (d, *J* = 11.1 Hz, 1H), 3.42 (d, *J* = 10.7 Hz, 1H), 3.34 (d, *J* = 10.7 Hz, 1H), 2.68 (d, *J* = 4.9 Hz, 1H), 2.32 (dd, *J* = 14.6, 3.8 Hz, 1H), 1.99 (d, *J* = 14.6 Hz, 1H), 1.59 (s, 3H), 1.38 (s, 3H), 1.31 (s, 3H).
^{13}C NMR	(150 MHz, $CDCl_3$)<br>δ 169.8, 100.1, 81.8, 81.6, 77.0 (overlaps with $CDCl_3$), 71.8, 69.0, 62.4, 60.6, 54.7, 46.1, 38.7, 33.5, 26.7, 21.6, 18.1.
HRMS	Calculated $C_{16}H_{20}BrO_5$ [M+H]: 371.0494 | Found: 371.0489

2.1 5MeBrPXN (9)

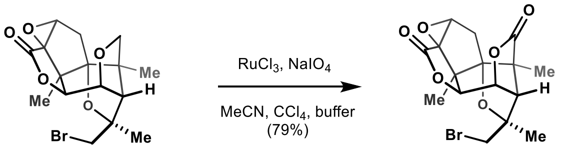

S1 5MeBrPXN **9**

Due to the Suárez reaction's ($Pb(OAc)_4$, I_2, *hv*) scalability limit, large scale (450 mg/single pass) of 5MeBrPXN (**9**) was obtained by modified procedure shown here. To a biphasic solution of ether **S1** (55 mg, 0.15 mmol, 1.0 equiv) in a mixture of MeCN (2 mL)/CCl_4 (2 mL)/ phosphate buffer (PH 6.9, 2.5 mL) was added solid $RuCl_3{\cdot}3H_2O$ (54 mg, 0.15 mmol, 1.0 equiv) at room temperature, the colorless aqueous solution immediately turned to black while ruthenium salt dissolving. $NaIO_4$ (633 mg, 2.97 mmol, 20.0 equiv) was added as solid to the above dark solution, then the aqueous solution turned to dark green. The resulting biphasic solution was vigorously stirred (1000 rpm) at 50 ºC (oil bath), after 30 min, dark green solution gradually turned to orange then yellow. After stirring 12 h, another portion of $RuCl_3{\cdot}3H_2O$ (54 mg, 0.15 mmol, 1.0 equiv) and $NaIO_4$ (633 mg, 2.97 mmol, 20.0 equiv) were sequentially added. The reaction mixture was stirred for another 12 h at the same temperature (50 ºC), which was then cooled to room temperature and quenched by adding a mixture of sat $Na_2S_2O_3$ (2 mL) and EtOAc (5 mL). In the meantime, light yellow solution with white precipitate turned to dark purple/black while $Na_2S_2O_3$ solution was added. This mixture was filtered through a short pad of Celite (~3 cm),

followed by washing with EtOAc (3 × 5 mL), and extraction with EtOAc (5 × 5 mL). The resulting yellow solution was concentrated *in vacuo* to give a dark orange residue, which was further purified through silica gel column (DCM/EtOAc, 30:1 to 20:1) to afford 5MeBrPXN **9** as white solid (46 mg, 0.12 mmol, 79%). 10 vials (total 550 mg scale) were running in parallel under the same condition, after combined workup and purification, 450 mg 5MeBrPXN was isolated. The ^{1}H and ^{13}C NMR of **9** are consistent with reported data.[1]

2.2 PTN (10)

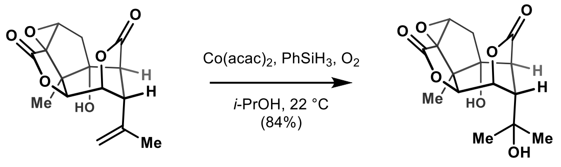

To a solution of PXN **1** (18 mg, 0.062 mmol, 1.0 equiv) in ***i***-PrOH (0.4 mL) was added Co(acac)$_2$ (1.7 mg, 0.0068 mmol, 0.11 equiv) and PhSiH$_3$ (7.4 mg, 0.068 mmol, 1.1 equiv). The reaction was fitted with a balloon of O$_2$ and purged with sonication for 5 min. The reaction was stirred at room temperature for 2 hours at which time TLC analysis showed consumption of starting material. The reaction mixture was concentrated and purified by silica column chromatography to give PTN **10** (16 mg, 84%) as a white solid. The ^{1}H and ^{13}C NMR of **10** are consistent with reported data.[1]

2.3 5MePTN (11)

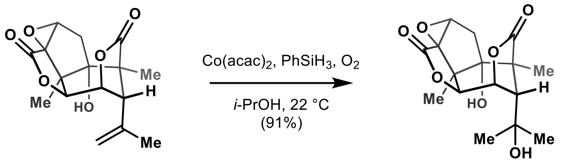

To a solution of 5MePXN **2** (10 mg, 0.033 mmol, 1.0 equiv) and Co(acac)$_2$ (1.7 mg, 6.5 μmol, 0.2 equiv) in *i*-PrOH (2 mL) was added PhSiH$_3$ (8.1 μL, 0.066 mmol, 2.0 equiv) at room temperature. The reaction was bubbled with a balloon of O$_2$ for 1 h, during this period, the pink solution turned to dark green. The reaction was allowed to stir for an additional 1 h at which time TLC analysis monitored consumption of starting material. The reaction mixture was concentrated *in vacuo* and purified by silica column chromatography (50% EtOAc/Hexane) to give 5MePTN **11** (9.7 mg, 0.03 mmol, 91%) as white solid.

Characterization data of **11**:

Description	white solid
R_f	0.40 in 75% EtOAc/Hexane. Stains dark brown in anisaldehyde.
Opt. Rot.	α_{obs} = -65.3º, c = 0.34 in MeOH, T = 20.0 ºC
^{1}H NMR	(600 MHz, CD$_3$OD)<br>δ 4.99 (dd, *J* = 5.3, 3.3 Hz, 1H), 4.89 (d, *J* = 3.3 Hz, 2H), 3.59 (d, *J* = 3.4 Hz, 1H), 2.74 (dd, *J* = 15.1, 3.4 Hz, 1H), 2.61 (d, *J* = 5.3 Hz, 1H), 1.79 (d, *J* = 15.1 Hz, 1H), 1.47 (s, 3H), 1.43 (s, 3H), 1.39 (s, 3H), 1.23 (s, 3H).
^{13}C NMR	(150 MHz, CD$_3$OD)<br>δ 179.5, 172.1, 88.8, 82.4, 77.8, 74.4, 70.5, 63.5, 58.3, 51.9, 49.8, 40.9, 30.9, 29.8, 17.4, 16.8.
HRMS	Calculated $C_{16}H_{21}O_7$ $[M+H]^+$: 325.1287, Found: 325.1291.
X-ray	Crystals were grown by slow evaporation from EtOAc in a 5 mL scintillation vial covered with Teflon tape at room temperature. (CCDC 2048201)

2.4 12FPXN (12)

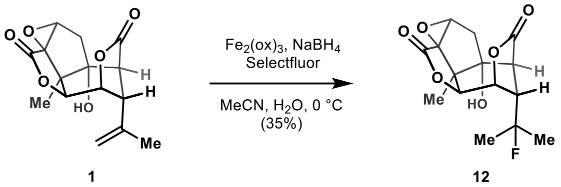

To a 25 mL vial charged $Fe_2(ox)_3{\cdot}6H_2O$ (33 mg, 0.068 mmol, 2.0 equiv) was added H_2O (2 mL) and then stirred at room temperature for 2 h. After degassing by bubbling with Ar for 10 min, the yellow-green solution of $Fe_2(ox)_3$ was sequentially added a solution of Selectfluor (24 mg, 0.068 mmol, 2.0 equiv) in MeCN (1 mL) and a solution of PXN **1** (10 mg, 0.034 mmol, 1.0 equiv) in MeCN (1 mL) at 0 ºC. $NaBH_4$ (8 mg, 0.22 mmol, 6.4 equiv) was added as solid, at this moment, the color of solution turned from yellow-green to yellow with a large amount of H_2 evolving. This solution was stirred at 0 ºC for 30 min at which time the reaction was quenched by adding 30% aq. NH_4OH (1 mL), then extracted by ethyl acetate (3 × 3 mL). The reaction mixture was concentrated and purified by silica column chromatography (30% EtOAc/Hexane) to give 12FPXN **12** (3.7 mg, 0.012 mmol, 35%) as a white solid and HPXN **20** (2.6 mg, 8.8 μmol, 26%) as white solid.

Characterization data of **12**:

Description	white solid
R_f	0.37 in 50% EtOAc/Hexane. Stains dark purple in anisaldehyde.
Opt. Rot.	α_{obs} = -10.8º, c = 0.37 in MeOH, T = 20.0 ºC
^{1}H NMR	(600 MHz, $CDCl_3$)<br>δ 5.00 (dd, *J* = 4.8, 3.6 Hz, 1H), 4.62 (t, *J* = 3.6 Hz, 1H), 3.74 (d, *J* = 3.4 Hz, 1H), 3.09 (d, *J* = 23.3 Hz, 1H), 2.93 (dd, *J* = 15.0, 3.6 Hz, 2H), 2.85 – 2.72 (m, 1H), 2.20 – 2.12 (m, 1H), 1.71 – 1.57 (m, 6H), 1.26 (d, *J* = 2.0 Hz, 3H).
^{13}C NMR	(150 MHz, $CDCl_3$)<br>δ 173.1, 169.0, 95.8 (d, *J* = 165.4 Hz), 85.6, 79.8 (d, *J* = 7.7 Hz), 76.5 (d, *J* = 2.1 Hz), 73.3, 61.8, 51.3 (d, *J* = 20.9 Hz), 50.1, 48.3, 43.7, 26.5 (t, *J* = 24.2 Hz), 16.1 (d, *J* = 5.5 Hz).
^{19}F NMR	(375 MHz, $CDCl_3$)<br>δ -142.07.
HRMS	Calculated $C_{15}H_{18}FO_6$ $[M+H]^+$: 313.1087, Found: 313.1089.

2.5 12F5MePXN (13)

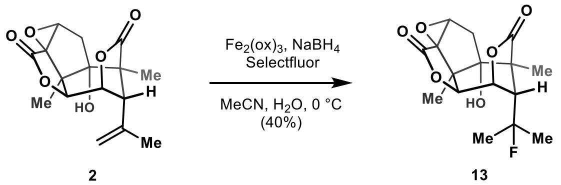

To a 25 mL vial charged $Fe_2(ox)_3{\cdot}6H_2O$ (32 mg, 0.066 mmol, 2.0 equiv) was added H_2O (2 mL) and then stirred at room temperature for 2 h. After degassing by bubbling with Ar for 10 min, the above yellow-green solution of $Fe_2(ox)_3$ was sequentially added a solution of Selectfluor (23 mg, 0.066 mmol, 2.0 equiv) in MeCN (1 mL) and a solution of 5MePXN **2** (10 mg, 0.033 mmol, 1.0 equiv) in MeCN (1 mL) at 0 ºC. $NaBH_4$ (8 mg, 0.22 mmol, 6.4 equiv) was added as solid, at this time, the color of solution turned from yellow-green to yellow with a large amount of H_2 evolving. This solution was stirred at 0 ºC for 30 min at which time the reaction was quenched by adding 30% aq. NH_4OH (1 mL), then extracted by ethyl acetate (3 × 3 mL). The reaction mixture was concentrated and purified by silica column chromatography to give 12F5MePXN **13** (4.3 mg, 0.013 mmol, 40%) as a white solid and H5MePXN **21** (3.4 mg, 0.011 mmol, 34%) as white solid.

Characterization data of **13**:

Description	white solid

R_f	0.38 in 50% EtOAc/Hexane. Stains blue in anisaldehyde.
Opt. Rot.	α_{obs} = -23.8º, c = 0.50 in MeOH, T = 20.0 ºC
^{1}H NMR	(600 MHz, $CDCl_3$) δ 4.95 (dd, *J* = 5.3, 3.4 Hz, 1H), 4.64 (t, *J* = 3.4 Hz, 1H), 3.70 (d, *J* = 3.5 Hz, 1H), 2.82 (dd, *J* = 15.3, 3.5 Hz, 1H), 2.73 (d, *J* = 22.9 Hz, 1H), 2.48 (dd, *J* = 39.0, 5.3 Hz, 1H), 1.92 (d, *J* = 15.3 Hz, 1H), 1.68 (d, *J* = 22.1 Hz, 3H), 1.62 (d, *J* = 22.7 Hz, 3H), 1.54 (s, 3H), 1.27 (d, *J* = 2.1 Hz, 3H).
^{13}C NMR	(150 MHz, $CDCl_3$) δ 176.5, 169.2, 96.6 (d, *J* = 165.8 Hz, 1H), 88.1, 80.0 (d, *J* = 8.2 Hz), 75.2 (d, *J* = 2.4 Hz), 73.2, 62.2, 56.1 (d, *J* = 21.1 Hz), 50.6 (d, *J* = 1.6 Hz), 48.7, 40.9, 27.7 (d, *J* = 25.4 Hz), 27.2 (d, *J* = 23.3 Hz), 17.2, 16.5 (d, *J* = 6.1 Hz).
^{19}F NMR	(375 MHz, $CDCl_3$) δ -141.48.
HRMS	Calculated $C_{16}H_{20}FO_6$ $[M+H]^+$: 327.1244, Found: 327.1245.
X-ray	Crystals were grown by slow evaporation from $CDCl_3$ in a 5 mL scintillation vial at room temperature. (CCDC 2081332)

2.6 CypPXN (14)

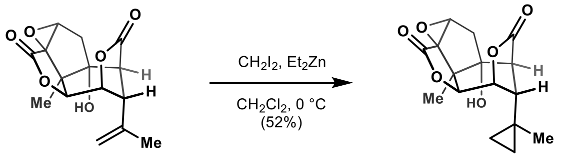

Et_2Zn (140 μL, 1 M in hexane, 0.14 mmol, 3.0 equiv) was added into the solution of PXN **1** (13.5 mg, 0.046 mmol, 1.0 equiv) and CH_2I_2 (19 μL, 0.23 mmol, 5.0 equiv) in CH_2Cl_2 (1 mL) at 0 ºC. The reaction was slowly warmed up to room temperature over 1 h, and stirred at room temperature for an additional 6 h. The reaction was quenched by adding sat. NH_4Cl (1 mL), extracted by EtOAc (3 × 3 mL), and dried over Na_2SO_4. The combined organic layers were concentrated *in vacuo* and purified by silica column chromatography (30% EtOAc/Hexane) to give CypPXN **14** (7.2 mg, 0.024 mmol, 52%) as a white solid and recovered PXN (6 mg, 0.021 mmol, 45%).

Characterization data of **14**:

Description	white solid
R_f	0.58 in 50% EtOAc/Hexane. Stains dark blue in anisaldehyde.
Opt. Rot.	α_{obs} = -0.8º, c = 0.72 in MeOH, T = 20.0 ºC
^{1}H NMR	(600 MHz, $CDCl_3$) δ 4.72 (dd, *J* = 4.7, 3.5 Hz, 1H), 4.33 (d, *J* = 3.5 Hz, 1H), 3.75 (d, *J* = 3.1 Hz, 1H), 3.17 (t, *J* = 4.7 Hz, 1H), 2.90 (dd, *J* = 14.9, 3.5 Hz, 1H), 2.81 (d, *J* = 4.0 Hz, 1H), 2.36 (s, 1H), 2.06 (d, *J* = 14.9 Hz, 1H), 1.36 (s, 3H), 1.25 (s, 3H), 0.68 (d, *J* = 10.5 Hz, 1H), 0.62 (d, *J* = 9.8 Hz, 1H), 0.55 (d, *J* = 2.1 Hz, 2H).
^{13}C NMR	(150 MHz, $CDCl_3$) δ 174.0, 168.9, 85.8, 79.5, 77.7, 72.8, 61.7, 50.2, 49.1, 47.3, 44.4, 25.9, 15.7, 13.9, 12.9, 11.0.
HRMS	Calculated $C_{16}H_{19}O_6$ $[M+H]^+$: 307.1182, Found: 307.1188.
X-ray	Crystals were grown by slow evaporation from CH_2Cl_2/EtOAc (10:1) in a 5 mL scintillation vial covered with Teflon tape at room temperature. (CCDC 2071501)

2.7 Cyp5MePXN (15)

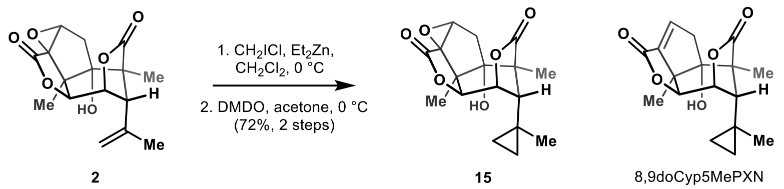

To a flame dried 10 mL screw cap tube charged a solution of CH_2ICl (95 µL, 1.3 mmol, 20.0 equiv) in dry CH_2Cl_2 (1 mL) was slowly added Et_2Zn (0.65 mL, 1 M in hexane, 0.65 mmol, 10.0 equiv) at 0 ºC (ice water bath) under Ar. A large amount of white precipitate formed after stirring for 5 minutes. After stirring another 10 min, a solution of 5MePXN **2** (20 mg, 0.065 mmol, 1.0 equiv) in CH_2Cl_2 (1 mL) was added to above white suspension. The resulting mixture was stirred for 12 h at room temperature at which time LC-MS analysis showed consumption of starting material. The reaction was quenched by sat. NH_4Cl (0.5 mL), and extracted by CH_2Cl_2 (5 × 5 mL). The combined organic layer was dried over Na_2SO_4 and then concentrated *in vacuo* to give a mixture of Cyp5MePXN and 8,9doCyp5MePXN (~ 1:3 from crude ^{1}H NMR), which was used to next step without further purification. To a solution of above crude mixture in CH_2Cl_2 (1 mL) was slowly added freshly prepared DMDO (6.5 mL, 0.06 M in acetone, 0.39 mmol, 6.0 equiv) at 0 ºC. The resulting solution was warmed up to room temperature. When LC-MS analysis showed the complete transformation of 8,9doCyp5MePXN to Cyp5MePXN (~12 h), the reaction was quenched by sat $Na_2S_2O_3$ (1 mL), then extracted with CH_2Cl_2 (3 × 5 mL). The combined organic layers were concentrated *in vacuo* and purified by silica column chromatography to afford Cyp5MePXN **15** (15 mg, 0.047 mmol, 72%, two steps) as white solid.

Characterization data of **15**:

Description	white solid
R_f	0.43 in 10% EtOAc/ CH_2Cl_2. Stains blue in anisaldehyde.
Opt. Rot.	α_{obs} = -10.1º, c = 0.36 in CH_2Cl_2, T = 20.0 ºC
^{1}H NMR	(600 MHz, $CDCl_3$) δ 4.76 (dd, *J* = 5.2, 3.2 Hz, 1H), 4.58 (d, *J* = 3.2 Hz, 1H), 3.71 (d, *J* = 3.5 Hz, 1H), 2.96 (dd, *J* = 15.1, 3.5 Hz, 1H), 2.34 (d, *J* = 5.2 Hz, 1H), 2.09 (s, 1H), 1.77 (d, *J* = 15.1 Hz, 1H), 1.38 (s, 3H), 1.37 (s, 3H), 1.24 (s, 3H), 0.81 (dd, *J* = 9.2, 4.7 Hz, 1H), 0.55 (dd, *J* = 9.0, 4.7 Hz, 1H), 0.48 – 0.41 (m, 2H).
^{13}C NMR	(150 MHz, $CDCl_3$) δ 177.5, 169.1, 87.7, 80.2, 77.4, 73.2, 61.9, 55.7, 50.6, 47.8, 42.9, 25.6, 16.1 (2 carbons), 13.3, 12.8 (2 carbons).
HRMS	Calculated $C_{17}H_{21}O_6$ $[M+H]^+$: 321.1338, Found: 321.1336.

2.8 12MePXN (16)

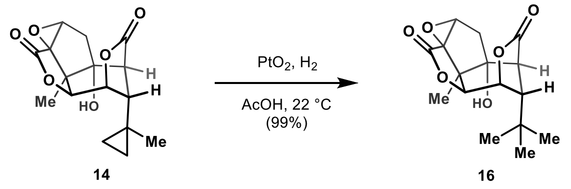

A 15 mL screw cap test tube was charged with PtO_2 (1.4 mg, 6 µmol, 0.5 equiv). The tube was evacuated and backfilled with H_2 (balloon) three times before adding a solution of CypPXN **14** (3.7 mg, 0.012 mmol, 1.0 equiv) in AcOH (1 mL). The reaction mixture was vigorous stirred for 8 h at which time LC-MS analysis detected consumption of starting material. The reaction was quenched by sat $NaHCO_3$ (2 mL)/EtOAc (3 mL). Then more $NaHCO_3$ solid was added until no CO_2 evolving. The resulting mixture was filtered through a short pad of Celite, washed with EtOAc (3 × 1 mL), and dried over Na_2SO_4. The crude product was concentrated *in vacuo* and purified by prep thin layer chromatography (50% EtOAc/Hexane) to furnish 12MePXN **16** (3.6 mg, 0.012 mmol, 99%) as white solid. The ^{1}H and ^{13}C NMR of **16** are consistent with reported data.[2]

Characterization data of **16**:

Description white solid

R_f 0.61 in 50% EtOAc/Hexane. Stains purple/brown in anisaldehyde.

^{1}H NMR (600 MHz, $CDCl_3$)
δ 4.91 (dd, *J* = 4.4, 3.3 Hz, 1H), 4.72 (d, *J* = 3.3 Hz, 1H), 3.75 (d, *J* = 3.4 Hz, 1H), 3.06 (dd, *J* = 14.9, 3.4 Hz, 1H), 2.75 (d, *J* = 3.9 Hz, 1H), 2.67 – 2.55 (m, 1H), 2.09 (s, 1H), 1.97 (d, *J* = 14.9 Hz, 1H), 1.35 (s, 3H), 1.17 (s, 9H).

^{13}C NMR (150 MHz, $CDCl_3$)
δ 174.6, 168.9, 85.3, 80.2, 78.3, 73.2, 61.4, 55.7, 50.7, 47.0, 46.3, 31.3, 29.8, 15.8.

LCMS Calculated: $C_{16}H_{21}O_6$ $[M+H]^+$: 309.1, Found: 309.1.

2.9 12Me5MePXN (17)

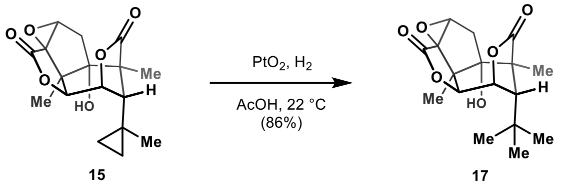

A flame dried 10 mL screw cap test tube was charged with PtO_2 (2.5 mg, 50 wt%) under Ar. The vial was then evacuated and backfilled with H_2 (balloon) for three times. A solution of Cyp5MePXN **15** (5 mg, 0.016 mmol, 1.0 equiv) in AcOH (1 mL) was added into above vial. H_2 (balloon, 1 atm) was introduced through a long needle in order to control bubbling in solvent. The reaction was vigorously stirred and bubbled at room temperature for 2 hours. As LC-MS shown all starting material consumption, the reaction was slowly quenched by a mixture of CH_2Cl_2/ sat $NaHCO_3$ (1:1, 4 mL). More solid $NaHCO_3$ was added to the reaction until no CO_2 evolving. The organic layer was separated, the aqueous layer was washed with CH_2Cl_2 (3 × 2 mL). Then combined organic layer was filtered through a pad of Celite and concentrated as pale yellow residue, which was purified by prep thin layer chromatography (10% EtOAc/ CH_2Cl_2) to afford 12Me5MePXN **17** (4.5 mg, 0.014 mmol, 86%) as white solid.

Characterization data of **17**:

Description white solid

R_f 0.40 in 10% EtOAc/ CH_2Cl_2. Stains purple in anisaldehyde.

Opt. Rot. α_{obs} = -10.3º, c = 0.26 in CH_2Cl_2, T = 20.0 ºC

^{1}H NMR (600 MHz, $CDCl_3$)
δ 4.85 (dd, *J* = 5.0, 3.2 Hz, 1H), 4.74 (d, *J* = 3.2 Hz, 1H), 3.71 (d, *J* = 3.4 Hz, 1H), 3.03 (dd, *J* = 15.1, 3.6 Hz, 1H), 2.36 (d, *J* = 5.0 Hz, 1H), 2.06 (s, 1H), 1.71 (d, *J* = 15.1 Hz, 1H), 1.44 (s, 3H), 1.35 (s, 3H), 1.18 (s, 9H).

^{13}C NMR (150 MHz, $CDCl_3$)
δ 177.7, 169.1, 87.8, 80.3, 77.0, 73.3, 61.8, 60.1, 51.2, 47.6, 43.6, 32.7, 30.8, 17.4, 16.1.

HRMS Calculated $C_{17}H_{23}O_6$ $[M+H]^+$: 323.1495, Found: 323.1504.

2.10 8,9doPXN (18)

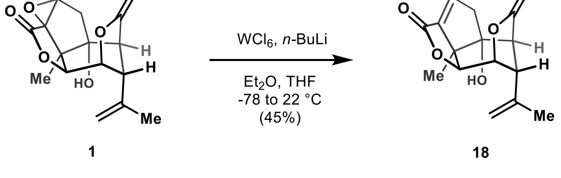

To a solution of WCl_6 (198 mg, 0.5 mmol, 5.0 equiv) in Et_2O (5 mL) was added *n*BuLi (0.44 mL, 2.5 M in hexane, 1.1 mmol, 11.0 equiv) at –78 ºC dry ice/acetone bath. After stirring for 5 min, the reaction was moved to ice water bath, at the same time, a solution of PXN **1** (29.2 mg, 0.1 mmol, 1.0 equiv) in THF (0.5 mL) was slowly added at –78 ºC. The reaction was stirred at room temperature for 2 h, then filtered through a pad of Celite, washed with EtOAc (3 × 5 mL). The solution was concentrated and purified by silica column chromatography to afford 8,9-doPXN **18** (12 mg, 0.045 mmol, 45%) as a white solid. The ^{1}H and ^{13}C NMR of **18** are consistent with reported data.[3]

Characterization data of **18**:

Description	white solid
R_f	0.40 in 60% EtOAc/Hexane. Stains dark blue in anisaldehyde.
^{1}H NMR	(600 MHz, $CDCl_3$)
	δ 6.46 – 6.32 (m, 1H), 5.15 – 5.04 (m, 1H), 4.98 (ddd, *J* = 4.8, 3.5, 0.5 Hz, 1H), 4.86 (d, *J* = 1.8 Hz, 1H), 4.70 (d, *J* = 3.4 Hz, 1H), 3.39 (s, 1H), 3.32 (dd, *J* = 17.8, 3.8 Hz, 1H), 3.09 (dd, *J* = 17.8, 2.0 Hz, 1H), 2.92 (d, *J* = 4.5 Hz, 1H), 2.24 (s, 1H), 2.00 – 1.89 (m, 3H), 1.32 (s, 3H).
^{13}C NMR	(150 MHz, $CDCl_3$)
	δ 174.5, 163.6, 142.4, 140.3, 134.9, 113.8, 83.7, 79.4, 78.2, 53.8, 49.9, 49.6, 49.3, 23.6, 19.4.
LCMS	Calculated $C_{15}H_{17}O_5$ $[M+H]^+$: 277.1, Found: 277.1.

2.11 8,9do5MePXN (19)

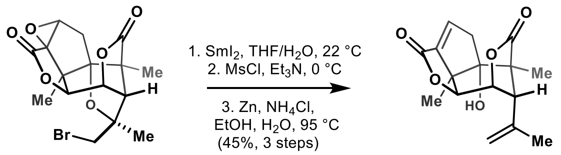

9 19

To a solution of 5MeBrPXN **9** (20 mg, 0.052 mmol, 1.0 equiv) in degassed THF/H_2O (v:v, 20:1, 2.1 mL) was added SmI_2 (2.0 mL, 0.1 M in THF, 0.2 mmol, 4.0 equiv) at room temperature under Ar. The reaction was stirred until TLC detected starting material consumption (~5 h), which was then quenched by adding sat NH_4Cl (0.5 mL), and extracted by EtOAc (3 × 5 mL). The organic layer was filtered through a short pad of Celite, washed with EtOAc (3 x 3 mL). The combined organic layer was dried over Na_2SO_4. The crude product was concentrated *in vacuo* to give a residue which was then dissolved in DCM (2 mL). Et_3N (72 μL, 0.52 mmol, 10.0 equiv), MsCl (20 μL, 0.26 mmol, 5.0 equiv) were sequentially added to the reaction mixture at 0 ºC. The resulting yellow solution was stirred for 1.5 h at room temperature. A solution of sat $NaHCO_3$ (0.5 mL) was added to quench the reaction. The organic layer was filtered through a short pad of Celite, washed with DCM (3 × 3 mL). The crude product was concentrated *in vacuo* to give a corresponding residue which was then dissolved in a mixture of EtOH (1.5 mL) and H_2O (0.15 mL). Zinc (34 mg, 0.52 mmol, 10.0 equiv), solid NH_4Cl (56 mg, 1.04 mmol, 20.0 equiv) were added to above colorless solution to form a gray suspension. The suspension was heated at 90 ºC until no starting material remaining based on LC-MS (ca. 2.5 h). The reaction was cooled down to room temperature, then filtered through a pad of Celite, washed with EtOAc (3 × 5 mL). The combined organic layer was concentrated *in vacuo* and purified by silica column chromatography to give 8,9do5MePXN **19** (6.7 mg, 0.023 mmol, 45%, 3 steps) as white solid.

Characterization data of **19**:

Description	white solid
R_f	0.37 in 10% EtOAc/CH_2Cl_2. UV active. Stains dark blue in anisaldehyde.
Opt. Rot.	α_{obs} = +18.7º, c = 0.43 in CH_2Cl_2, T = 20.0 ºC
^{1}H NMR	(600 MHz, $CDCl_3$)

δ 6.34 (dd, J = 3.8, 1.9 Hz, 1H), 5.17 – 5.08 (m, 1H), 4.98 (d, J = 1.6 Hz, 1H), 4.91 (dd, J = 5.0, 3.4 Hz, 1H), 4.67 (d, J = 3.4 Hz, 1H), 3.28 (dd, J = 18.0, 3.8 Hz, 1H), 3.07 (d, J = 5.0 Hz, 1H), 2.86 (dd, J = 18.0, 1.9 Hz, 1H), 1.98 (s, 3H), 1.53 (s, 3H), 1.34 (s, 3H).

^{13}C NMR (150 MHz, $CDCl_3$)
δ 177.8, 163.8, 142.2, 140.4, 134.8, 115.5, 85.8, 79.6, 76.6, 55.0, 54.4, 49.9, 46.8, 24.7, 19.9, 16.5.

HRMS Calculated $C_{16}H_{19}O_5$ $[M+H]^+$: 291.1232, Found: 291.1239.

2.12 HPXN (20)

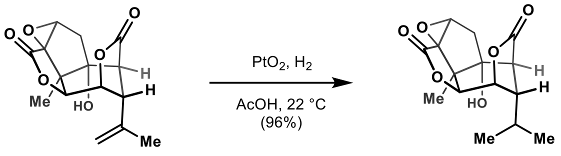

1 → 20

A solution of PXN **1** (10 mg, 0.034 mmol, 1.0 equiv) and PtO_2 (1.6 mg, 6.8 μmol, 0.2 equiv) in AcOH (0.5 mL) was bubbled under H_2 (1 atm) at room temperature for 30 min. The reaction was further stirred under H_2 balloon atmosphere overnight at which time LC-MS detected all starting material consumption. The reaction mixture was quenched by adding sat $NaHCO_3$/EtOAc (v:v, 1:1, 6 mL). Then solid $NaHCO_3$ was added in portions until no CO_2 evolving. The organic layer was separated and aqueous layer was extracted by EtOAc (3 × 3 mL). The combined organic layer was dried over Na_2SO_4, and concentrated *in vacuo* to give HPXN **20** (9.6 mg, 0.033 mmol, 96%) as white solid. The ^{1}H and ^{13}C NMR of **20** are consistent with reported data.[4]

Characterization data of **20**:

Description white solid

R_f 0.49 in 50% EtOAc/Hexane. Stains brown in anisaldehyde.

^{1}H NMR (600 MHz, $CDCl_3$)
δ 4.86 (dd, J = 5.5, 3.3 Hz, 1H), 4.46 (d, J = 3.3 Hz, 1H), 3.75 (d, J = 3.4 Hz, 1H), 3.05 (dd, J = 15.0, 3.4 Hz, 1H), 2.74 (d, J = 4.5 Hz, 1H), 2.45 – 2.35 (m, 1H), 1.94 (d, J = 15.0 Hz, 1H), 1.83 (tt, J = 12.8, 6.4 Hz, 1H), 1.34 (s, 3H), 1.06 (d, J = 6.2 Hz, 3H), 1.03 (d, J = 6.6 Hz, 3H).

^{13}C NMR (150 MHz, $CDCl_3$)
δ 174.9, 168.8, 86.3, 80.1, 78.2, 72.6, 61.4, 50.9, 50.3, 47.2, 45.7, 25.6, 22.1, 21.0, 16.3.

LCMS Calculated $C_{15}H_{19}O_6$ $[M+H]^+$: 295.1, Found: 295.1.

X-ray Crystals were grown by slow evaporation from $CDCl_3/CH_2Cl_2$ (1:1) in a 5 mL scintillation vial covered with Teflon tape at room temperature. (CCDC 2048202)

2.13 H5MePXN (21)

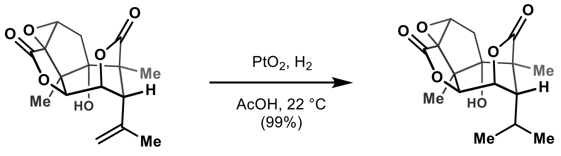

2 → 21

A solution of 5MePXN **2** (10 mg, 0.033 mmol, 1.0 equiv) and PtO_2 (1.5 mg, 6.5 μmol, 0.2 equiv) in AcOH (2 mL) was bubbled under H_2 (1 atm) at room temperature for 30 min. Then the reaction was stirred overnight at which time LC-MS detected all starting material consumption (~15 h). The reaction mixture was quenched by adding a mixture of sat $NaHCO_3$/EtOAc (v:v, 1:1, 6 mL). Then solid $NaHCO_3$ was added in portions until no CO_2 evolving. The organic layer was separated and aqueous layer was extracted by EtOAc (3 × 3 mL). The

combined organic layer was dried over Na_2SO_4 then concentrated *in vacuo* and purified by silica column chromatography (10% EtOAc/CH_2Cl_2) to give H5MePXN **21** (10.2 mg, 0.033 mmol, 99%) as white solid.

Characterization data of **21**:

Description	white solid
R_f	0.45 in 50% EtOAc/Hexane. Stains dark brown in anisaldehyde.
Opt. Rot.	α_{obs} = +20.4º, c = 0.26 in MeOH, T = 20.0 ºC
^{1}H NMR	(600 MHz, $CDCl_3$)<br>δ 4.81 (dd, *J* = 5.6, 3.3 Hz, 1H), 4.48 (d, *J* = 3.3 Hz, 1H), 3.71 (d, *J* = 3.5 Hz, 1H), 2.99 (dd, *J* = 15.1, 3.5 Hz, 1H), 2.10 (dd, *J* = 11.6, 5.7 Hz, 1H), 1.91 (s, 1H), 1.85 – 1.78 (m, 1H), 1.69 (d, *J* = 15.1 Hz, 1H), 1.43 (s, 3H), 1.35 (s, 3H), 1.12 (d, *J* = 6.3 Hz, 3H), 1.04 (d, *J* = 6.6 Hz, 3H).
^{13}C NMR	(150 MHz, $CDCl_3$)<br>δ 178.0, 168.8, 88.7, 80.4, 76.8, 72.5, 61.9, 56.0, 50.3, 47.7, 43.1, 26.7, 22.4, 22.3, 17.1, 17.0.
HRMS	Calculated $C_{16}H_{21}O_6$ $[M+H]^+$: 309.1338, Found: 309.1342.
X-ray	Crystals were grown by slow evaporation from $CDCl_3$/ CH_2Cl_2 (1:1) in a 5 mL scintillation vial at room temperature. (CCDC 2048203)

2.14 6FPXN (22)

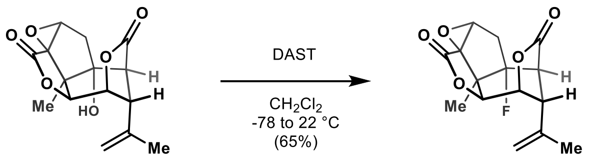

DAST (1.7 μL, 0.013 mmol, 1.0 equiv) was added to a solution of PXN **1** (3.8 mg, 0.013 mmol, 1.0 equiv) in CH_2Cl_2 (0.5 mL) under Ar at –78 ºC. The reaction mixture was slowly warmed up to room temperature over 1 h, then stirred at room temperature for additional 5 h. The reaction was quenched by sat $NaHCO_3$ (5 drops). The crude residue was purified by prep thin layer chromatography (33% EtOAc/Hexane) to afford 6FPXN **22** (2.5 mg, 8.5 μmol, 65%) as white solid.

Characterization data[5] of **22**:

Description	white solid
R_f	0.43 in 30% EtOAc/Hexane. Stains dark brown in anisaldehyde.
Opt. Rot.	α_{obs} = -18.1º, c = 0.32 in MeOH, T = 20.0 ºC
^{1}H NMR	(600 MHz, $CDCl_3$)<br>δ 5.16 (dd, *J* = 4.6, 3.7 Hz, 1H), 5.08 (dd, *J* = 2.3, 1.4 Hz, 1H), 4.93 (d, *J* = 3.7 Hz, 1H), 4.83 (d, *J* = 1.9 Hz, 1H), 3.74 (dd, *J* = 6.2, 3.2 Hz, 1H), 3.50 (s, 1H), 3.14 (dd, *J* = 9.6, 4.6 Hz, 1H), 2.97 (dd, *J* = 15.2, 3.5 Hz, 1H), 2.29 (dd, *J* = 19.4, 15.2 Hz, 1H), 1.84 (s, 3H), 1.27 (d, *J* = 4.9 Hz, 3H).
^{13}C NMR	(150 MHz, $CDCl_3$)<br>δ 172.2 (d, *J* = 18.7 Hz), 168.1, 138.4, 113.8 (d, *J* = 2.1 Hz), 103.4 (d, *J* = 212.5 Hz), 79.0, 77.2, 71.2 (d, *J* = 3.4 Hz), 60.8 (d, *J* = 6.4 Hz), 49.2 (d, *J* = 27.6 Hz), 48.1 (d, *J* = 2.3 Hz), 46.2, 41.1 (d, *J* = 25.4 Hz), 22.6 (d, *J* = 7.8 Hz), 16.1 (d, *J* = 11.0 Hz).
^{19}F NMR	(375 MHz, $CDCl_3$)<br>δ -143.15.
HRMS	Calculated $C_{15}H_{16}FO_5$ $[M+H]^+$: 295.0982, Found: 295.0988.

2.15 6F5MePXN (23)

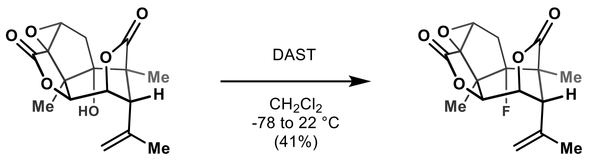

2 23

To a solution of 5MePXN **2** (10 mg, 0.033 mmol, 1.0 equiv) in CH_2Cl_2 (1.5 mL) was added DAST (5.2 μL, 0.04 mmol, 1.2 equiv) at –78 ºC. The reaction mixture was slowly warmed up to room temperature over 1 h, then stirred at room temperature for additional 5 h. The reaction was quenched by slowly adding sat $NaHCO_3$ (5 drops) at 0 ºC. The organic layer was separated and concentrated *in vacuo*. The crude residue was purified by prep thin layer chromatography (33% EtOAc/Hexane) to afford 6F5MePXN **23** (4.2 mg, 13.5 μmol, 41%) as white solid.

Characterization data of **23**:

Description white solid

R_f 0.53 in 30% EtOAc/Hexane. Stains blue in anisaldehyde.

Opt. Rot. α_{obs} = -26.8º, c = 0.25 in MeOH, T = 20.0 ºC

^{1}H NMR (600 MHz, $CDCl_3$)
δ 5.14 (d, *J* = 1.5 Hz, 1H), 5.08 (dd, *J* = 5.4, 3.3 Hz, 1H), 5.06 (d, *J* = 1.9 Hz, 1H), 4.74 (d, *J* = 3.3 Hz, 1H), 3.71 (dd, *J* = 6.2, 3.3 Hz, 1H), 3.19 (d, *J* = 4.8 Hz, 1H), 2.97 (dd, *J* = 15.6, 3.5 Hz, 1H), 2.16 – 2.05 (m, 1H), 1.89 (s, 3H), 1.51 (d, *J* = 1.3 Hz, 3H), 1.28 (d, *J* = 5.3 Hz, 3H).

^{13}C NMR (150 MHz, $CDCl_3$)
δ 175.7 (d, *J* = 13.8 Hz), 168.2, 137.2, 116.2 (d, *J* = 8.6 Hz), 106.0 (d, *J* = 216.1 Hz), 79.8, 76.2, 71.6 (d, *J* = 2.8 Hz), 61.3 (d, *J* = 6.0 Hz), 53.3 (d, *J* = 1.5 Hz), 50.4 (d, *J* = 25.9 Hz), 47.1 (d, *J* = 23.4 Hz), 39.1 (d, *J* = 27.1 Hz), 23.6 (d, *J* = 4.9 Hz), 16.6 (d, *J* = 12.3 Hz), 15.7 (d, *J* = 4.4 Hz).

^{19}F NMR (375 MHz, $CDCl_3$)
δ -145.89.

HRMS Calculated $C_{16}H_{18}FO_5$ $[M+H]^+$: 309.1138, Found: 309.1143.

X-ray Crystals were grown by slow evaporation from CH_2Cl_2 in a 5 mL scintillation vial covered with Teflon tape at room temperature. (CCDC 2069870)

2.16 6FPTN (24)

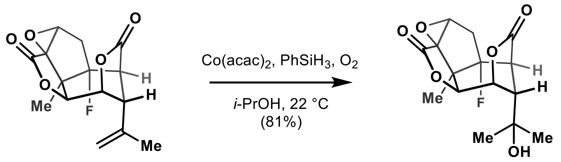

22 24

A solution of 6FPXN **22** (5.6 mg, 0.019 mmol, 1.0 equiv) and $Co(acac)_2$ (1 mg, 3.8 μmol, 0.2 equiv) in *i*-PrOH (1 mL) was bubbled under O_2 (balloon, 1 atm) for 5 min. $PhSiH_3$ (7 μL, 0.057 mmol, 3.0 equiv) was added dropwise while O_2 bubbling, after 10 min, the pink solution turned to slightly green. The reaction was allowed to stir for an additional 3 h at which time TLC monitored consumption of starting material. The reaction mixture was concentrated *in vacuo* and purified by silica column chromatography (50% EtOAc/Hexane) to give 6FPTN **24** (4.8 mg, 0.015 mmol, 81%) as white solid.

Characterization data of **24**:

Description white solid

R_f 0.40 in 50% EtOAc/Hexane. Stains blue in anisaldehyde.

Opt. Rot. α_{obs} = -16.3º, c = 0.37 in CH_2Cl_2, T = 20.0 ºC

^{1}H NMR (600 MHz, $CDCl_3$)
δ 5.06 – 5.02 (m, 1H), 4.99 (d, *J* = 3.4 Hz, 1H), 3.76 (dd, *J* = 6.8, 3.3 Hz, 1H), 3.03 – 2.95 (m, 2H), 2.75 (t, *J* = 4.5 Hz, 1H), 2.37 (dd, *J* = 19.2, 15.0 Hz, 1H), 1.41 (s, 3H), 1.39 (s, 3H), 1.35 (d, *J* = 3.7 Hz, 3H).

^{13}C NMR (150 MHz, $CDCl_3$)
δ 172.0, 167.9, 104.1 (d, *J* = 208.4 Hz), 79.5, 77.9, 71.9 (d, *J* = 3.4 Hz), 68.5, 60.4 (d, *J* = 7.1 Hz), 53.4 (d, *J* = 2.2 Hz), 48.3 (d, *J* = 27.9 Hz), 47.4 (d, *J* = 20.5 Hz), 41.4 (d, *J* = 26.5 Hz), 29.8 (d, *J* = 1.7 Hz), 29.0 (d, *J* = 2.4 Hz), 15.6 (d, *J* = 7.8 Hz).

^{19}F NMR (375 MHz, $CDCl_3$)
δ -141.48.

HRMS Calculated $C_{15}H_{18}FO_6$ $[M+H]^+$: 313.1087, Found: 313.1091.

2.17 6F5MePTN (25)

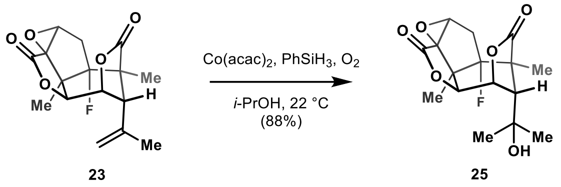

A solution of 6F5MePXN **23** (8 mg, 0.026 mmol, 1.0 equiv) and $Co(acac)_2$ (1.3 mg, 5.2 μmol, 0.2 equiv) in *i*-PrOH (1 mL) was bubbled under O_2 (balloon, 1 atm) at room temperature. $PhSiH_3$ (10 μL, 0.078 mmol, 3.0 equiv) was slowly added as dropwise while O_2 bubbling, the pink solution slowly turned to deep green. The reaction was stirred for 3 h at room temperature. The crude product was concentrated *in vacuo* and purified by prep thin layer chromatography to furnish 6F5MePTN **25** (7.5 mg, 0.023 mmol, 88%) as white solid.

Characterization data of **25**:

Description white solid

R_f 0.46 in 50% EtOAc/Hexane. Stains dark brown in anisaldehyde.

Opt. Rot. α_{obs} = -12.9º, c = 0.31 in CH_2Cl_2, T = 20.0 ºC

^{1}H NMR (600 MHz, $CDCl_3$)
δ 5.02 (d, *J* = 3.4 Hz, 1H), 4.98 (dd, *J* = 5.2, 3.4 Hz, 1H), 3.71 (dd, *J* = 6.8, 3.3 Hz, 1H), 2.93 (dd, *J* = 15.3, 3.5 Hz, 1H), 2.47 (d, *J* = 5.2 Hz, 1H), 2.14 (dd, *J* = 19.1, 15.3 Hz, 1H), 2.05 (d, *J* = 13.5 Hz, 1H), 1.51 (d, *J* = 0.9 Hz, 3H), 1.47 (s, 3H), 1.39 (s, 3H), 1.34 (d, *J* = 4.1 Hz, 3H).

^{13}C NMR (150 MHz, $CDCl_3$)
δ 175.2 (d, *J* = 15.4 Hz), 168.6 (d, *J* = 2.7 Hz), 106.4 (d, *J* = 212.3 Hz), 79.6, 76.5, 71.8 (d, *J* = 3.5 Hz), 69.5, 60.8 (d, *J* = 7.1 Hz), 58.1 (d, *J* = 2.2 Hz), 49.7 (d, *J* = 26.4 Hz), 47.8 (d, *J* = 21.5 Hz), 38.6 (d, *J* = 26.5 Hz), 30.8, 30.0 (d, *J* = 2.1 Hz), 16.6 (d, *J* = 4.3 Hz), 15.9 (d, *J* = 8.7 Hz).

^{19}F NMR (375 MHz, $CDCl_3$)
δ -148.71.

HRMS Calculated $C_{16}H_{20}FO_6$ $[M+H]^+$: 327.1244, Found: 327.1250.

2.18 6FHPXN (26)

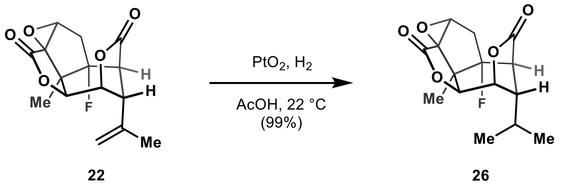

A 10 mL screw cap test tube was charged with PtO_2 (2.7 mg, 0.012 mmol, 0.5 equiv). The tube was evacuated and backfilled with H_2 for three times before adding a solution of 6FPXN **22** (7.1 mg, 0.024 mmol, 1.0 equiv) in AcOH (1 mL). The reaction mixture was vigorously stirred for 12 h at which time LC-MS analysis showed consumption of starting material. The reaction was quenched by sat $NaHCO_3$ (3 mL)/ CH_2Cl_2 (3 mL). Then $NaHCO_3$ solid was added until no more CO_2 evolving. The resulting mixture was filtered through a short pad of Celite, washed with CH_2Cl_2 (3 × 3 mL), and dried over Na_2SO_4. The crude product was concentrated *in vacuo* and purified by prep thin layer chromatography (50% EtOAc/Hexane) to furnish 6FHPXN **26** (7 mg, 0.024 mmol, 99%) as white solid.

Characterization data of **26**:

Description white solid

R_f 0.46 in 30% EtOAc/Hexane. Stains blue in anisaldehyde.

Opt. Rot. α_{obs} = -18.8º, c = 0.57 in CH_2Cl_2, T = 20.0 ºC

^{1}H NMR (600 MHz, $CDCl_3$)

δ 4.92 (dd, *J* = 5.5, 3.4 Hz, 1H), 4.51 (d, *J* = 3.4 Hz, 1H), 3.75 (dd, *J* = 6.3, 3.3 Hz, 1H), 3.03 – 2.88 (m, 2H), 2.52 – 2.41 (m, 1H), 2.30 (dd, *J* = 19.3, 15.2 Hz, 1H), 1.72 (ddd, *J* = 12.3, 6.2, 2.8 Hz, 1H), 1.38 (d, *J* = 4.4 Hz, 3H), 1.05 (dd, *J* = 9.4, 6.4 Hz, 6H).

^{13}C NMR (150 MHz, $CDCl_3$)

δ 172.9 (d, *J* = 19.2 Hz), 168.0, 103.9 (d, *J* = 211.8 Hz), 79.4, 78.0, 71.4 (d, *J* = 2.9 Hz), 60.7 (d, *J* = 6.2 Hz), 50.5 (d, *J* = 2.2 Hz), 48.8 (d, *J* = 27.0 Hz), 46.7 (d, *J* = 22.1 Hz), 41.2 (d, *J* = 25.4 Hz), 25.6 (d, *J* = 2.4 Hz), 21.7 (d, *J* = 3.3 Hz), 21.0, 16.1 (d, *J* = 10.0 Hz).

^{19}F NMR (375 MHz, $CDCl_3$)

δ -146.34.

HRMS Calculated $C_{15}H_{18}FO_5$ $[M+H]^+$: 297.1138, Found: 297.1147.

2.19 6FH5MePXN (27)

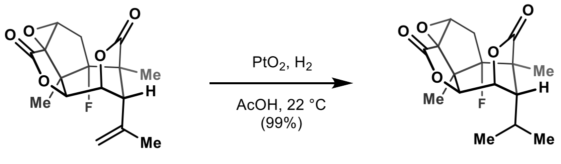

A 10 mL screw cap test tube was charged with PtO_2 (1 mg, 4.4 μmol, 0.5 equiv). The tube was evacuated and backfilled with H_2 for three times before adding a solution of 6F5MePXN **23** (2.7 mg, 8.8 μmol, 1.0 equiv) in AcOH (1 mL). The reaction mixture was vigorously stirred for 12 h at which time LC-MS analysis showed consumption of starting material. The reaction was quenched by sat $NaHCO_3$ (3 mL)/ CH_2Cl_2 (3 mL). Then $NaHCO_3$ solid was added until no CO_2 evolving. The resulting mixture was filtered through a short pad of Celite, washed with CH_2Cl_2 (3 × 3 mL), and dried over Na_2SO_4. The crude product was concentrated *in vacuo* and purified by prep thin layer chromatography (30% EtOAc/Hexane) to furnish 6FH5MePXN **27** (2.7 mg, 8.7 μmol, 99%) as white solid.

Characterization data of **27**:

Description white solid

R_f 0.53 in 30% EtOAc/Hexane. Stains slightly brown in anisaldehyde.

Opt. Rot. α_{obs} = -6.8º, c = 0.18 in CH_2Cl_2, T = 20.0 ºC

^{1}H NMR (600 MHz, $CDCl_3$)

δ 4.87 (dd, *J* = 5.7, 3.4 Hz, 1H), 4.53 (d, *J* = 3.4 Hz, 1H), 3.71 (dd, *J* = 6.3, 3.5 Hz, 1H), 2.90 (dd, *J* = 15.4, 3.5 Hz, 1H), 2.17 (dd, *J* = 11.6, 5.7 Hz, 1H), 2.08 (dd, *J* = 19.2, 15.4 Hz, 1H), 1.77 (ttd, *J* = 12.9, 6.5, 2.9 Hz, 1H), 1.45 (s, 3H), 1.38 (d, *J* = 4.8 Hz, 3H), 1.10 (d, *J* = 6.3 Hz, 3H), 1.07 (d, *J* = 6.6 Hz, 3H).

^{13}C NMR (150 MHz, $CDCl_3$)
δ 176.1 (d, *J* = 13.8 Hz), 168.1, 106.1 (d, *J* = 215.8 Hz), 79.7, 76.6, 71.3 (d, *J* = 2.9 Hz), 61.2 (d, *J* = 6.2 Hz), 55.6 (d, *J* = 2.1 Hz), 50.0 (d, *J* = 25.9 Hz), 47.1 (d, *J* = 23.2 Hz), 38.5 (d, *J* = 25.7 Hz), 26.6 (d, *J* = 2.2 Hz), 22.2, 22.1 (d, *J* = 2.4 Hz), 16.6 (d, *J* = 11.0 Hz), 16.4 (d, *J* = 4.6 Hz).

^{19}F NMR (375 MHz, $CDCl_3$)
δ -153.85.

HRMS Calculated $C_{16}H_{20}FO_5$ $[M+H]^+$: 311.1295, Found: 311.1297.

2.20 6F12FPXN (28)

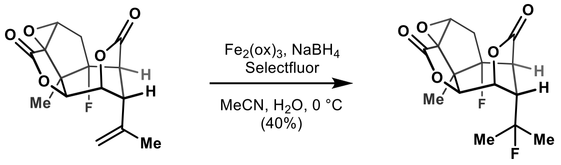

Vigorously stirring $Fe_2(ox)_3·6H_2O$ (32 mg, 0.066 mmol, 2.0 equiv) in H_2O (2 mL) at room temperature for 5 h formed a light yellow clear solution, which was then degassed for 30 min under Ar. To above solution was sequentially added 6FPXN **22** (10 mg, 0.033 mmol, 1.0 equiv) in MeCN (1 mL) and Selectfluor (23 mg, 0.066 mmol, 2.0 equiv) in MeCN (1 mL) at rt. After stirring 10 min, the mixture was cooled to 0 ºC in ice water bath followed by slowly adding solid $NaBH_4$ (8 mg, 0.22 mmol, 6.4 equiv). Note: A large amount of H_2 evolved during $NaBH_4$ addition. In the meantime, light yellow solution turned to pale orange. This solution was stirred at 0 ºC for 2 h at which time the reaction was quenched by addition of 30% aq. NH_4OH (1 mL), extracted by EtOAc (3 × 3 mL). The reaction mixture was concentrated and purified by silica gel column chromatography to afford 6F12FPXN **28** (4.1 mg, 0.013 mmol, 40%) as white solid.

Characterization data of **28**:

Description white solid

R_f 0.57 in 50% EtOAc/Hexane. Stains blue in anisaldehyde.

Opt. Rot. α_{obs} = -6.2º, c = 0.26 in CH_2Cl_2, T = 20.0 ºC

^1H NMR (600 MHz, $CDCl_3$)
δ 5.08 (dd, *J* = 4.6, 3.8 Hz, 1H), 4.76 (t, *J* = 3.8 Hz, 1H), 3.73 (dd, *J* = 6.2, 3.4 Hz, 1H), 3.05 (dd, *J* = 9.6, 4.0 Hz, 1H), 2.97 (dd, *J* = 15.2, 3.4 Hz, 1H), 2.79 (dt, *J* = 34.9, 4.6 Hz, 1H), 2.34 (dd, *J* = 18.6, 15.2 Hz, 1H), 1.56 (d, *J* = 11.8 Hz, 3H), 1.53 (d, *J* = 11.8 Hz, 3H), 1.33 (dd, *J* = 3.8, 1.0 Hz, 3H).

^{13}C NMR (150 MHz, $CDCl_3$)
δ 171.8, 168.4, 102.7 (d, *J* = 214.9 Hz), 91.9 (d, *J* = 174.4 Hz), 79.6 (d, *J* = 10.4 Hz), 76.9, 71.7 (d, *J* = 2.9 Hz), 60.5 (d, *J* = 6.3 Hz), 51.5 (dd, *J* = 21.7, 1.7 Hz), 48.1 (d, *J* = 28.7 Hz), 47.5 (d, *J* = 21.7 Hz), 41.5 (d, *J* = 26.0 Hz), 27.2 (d, *J* = 25.5 Hz), 26.0 (dd, *J* = 24.5, 4.1 Hz), 15.4 (dd, *J* = 9.3, 8.4 Hz).

^{19}F NMR (375 MHz, $CDCl_3$)
δ -145.10 (d, J_{F-F} = 31.2 Hz), -150.27 (d, J_{F-F} = 31.2 Hz).

HRMS Calculated $C_{15}H_{17}F_2O_5$ $[M+H]^+$: 315.1044, Found: 315.1051.

2.21 6F12F5MePXN (30)

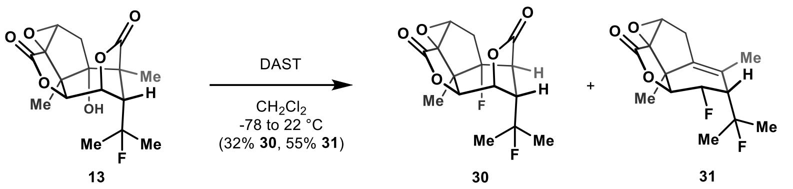

DAST (3.5 µL, 0.027 mmol, 1.0 equiv) was added to a solution of 12F5MePXN **13** (3.8 mg, 0.027 mmol, 1.0 equiv) in CH_2Cl_2 (0.5 mL) under Ar at –78 ºC. The reaction was slowly warmed up to room temperature over 1 h, then an additional portion of DAST (7 µL, 0.054 mmol, 2.0 equiv) was added at room temperature. The reaction was stirred at room temperature for additional 5 h. The reaction was quenched by sat $NaHCO_3$ (5 drops), filtered through a short pad of Celite, washed with DCM (5 × 3 mL). The crude residue was purified by prep thin layer chromatography (33% EtOAc/Hexane) to afford 6F12F5MePXN **30** (2.5 mg, 8.5 µmol, 32%) as white solid and alkene **31** (4.1 mg, 0.015 mmol, 55%) as white solid.

Characterization data of **30**:

Description	white solid
R_f	0.32 in 30% EtOAc/Hexane. Stains blue in anisaldehyde.
Opt. Rot.	α_{obs} = -10.1º, c = 0.22 in CH_2Cl_2, T = 20.0 ºC
^{1}H NMR	(600 MHz, $CDCl_3$)<br>δ 5.03 (dd, *J* = 5.3, 3.5 Hz, 1H), 4.79 (t, *J* = 3.9 Hz, 1H), 3.69 (dd, *J* = 6.3, 3.4 Hz, 1H), 2.89 (dd, *J* = 15.4, 3.6 Hz, 1H), 2.49 (dd, *J* = 34.6, 5.3 Hz, 1H), 2.11 (dd, *J* = 18.5, 15.4 Hz, 1H), 1.59 (d, *J* = 12.5 Hz, 3H), 1.58 (d, *J* = 21.5 Hz, 3H), 1.56 (d, *J* = 20.5, 3H), 1.32 (dd, *J* = 4.3, 1.5 Hz, 3H).
^{13}C NMR	(150 MHz, $CDCl_3$)<br>δ 175.0 (d, *J* = 14.0 Hz), 168.6 (d, *J* = 2.8 Hz), 105.0 (d, *J* = 219.0 Hz), 92.9 (d, *J* = 174.6 Hz), 79.8 (d, *J* = 12.0 Hz), 75.7 (d, *J* = 2.6 Hz), 71.6 (d, *J* = 3.0 Hz), 61.0 (d, *J* = 6.2 Hz), 56.3 (dd, *J* = 22.0, 1.6 Hz), 49.6 (dd, *J* = 27.1, 0.9 Hz), 47.9 (d, *J* = 23.0 Hz), 38.7 (d, *J* = 26.1 Hz), 28.4 (d, *J* = 24.9 Hz), 26.6 (dd, *J* = 25.1, 2.9 Hz), 16.6 (d, *J* = 4.2 Hz), 15.7 (dd, *J* = 10.2, 9.1 Hz).
^{19}F NMR	(375 MHz, $CDCl_3$)<br>δ -148.33 (d, $J_{F\text{-}F}$ = 27.8 Hz), -153.20 (d, $J_{F\text{-}F}$ = 27.8 Hz).
HRMS	Calculated $C_{16}H_{19}F_2O_5$ $[M+H]^+$: 329.1201, Found: 329.1202.
X-ray	Crystals were grown by slow evaporation from $CDCl_3$ in a 5 mL scintillation vial at room temperature. (CCDC 2079783)

Characterization data of **31**:

Description	white solid
R_f	0.39 in 30% EtOAc/Hexane. Stains blue in anisaldehyde.
Opt. Rot.	α_{obs} = +3.0º, c = 0.20 in CH_2Cl_2, T = 20.0 ºC
^{1}H NMR	(600 MHz, $CDCl_3$)<br>δ 5.03 (dd, *J* = 5.3, 3.5 Hz, 1H), 4.79 (t, *J* = 3.9 Hz, 1H), 3.69 (dd, *J* = 6.3, 3.4 Hz, 1H), 2.89 (dd, *J* = 15.4, 3.6 Hz, 1H), 2.49 (dd, *J* = 34.6, 5.3 Hz, 1H), 2.11 (dd, *J* = 18.5, 15.4 Hz, 1H), 1.59 (d, *J* = 12.5 Hz, 3H), 1.58 (d, *J* = 21.5 Hz, 3H), 1.56 (d, *J* = 20.5, 3H), 1.32 (dd, *J* = 4.3, 1.5 Hz, 3H).
^{13}C NMR	(150 MHz, $CDCl_3$)<br>δ 170.4, 145.9, 124.3, 97.2, 95.5 (d, *J* = 193.3 Hz), 84.9 (dd, *J* = 22.3, 8.6 Hz), 72.4 (d, *J* = 27.9 Hz), 66.5, 54.2 (dd, *J* = 22.6, 16.1 Hz), 45.2, 32.6, 28.7 (dd, *J* = 25.3, 8.6 Hz), 27.9 (d, *J* = 25.0 Hz), 21.5, 19.0 (d, *J* = 11.0 Hz).
^{19}F NMR	(375 MHz, $CDCl_3$)<br>δ -142.31, -184.58.
HRMS	Calculated $C_{15}H_{19}F_2O_3$ $[M+H]^+$: 285.1302, Found: 285.1309.

X-ray Crystals were grown by slow evaporation from EtOAc/CH_2Cl_2 (1:10) in a 5 mL scintillation vial covered with Teflon tape at room temperature. (CCDC 2078168)

3. Stability studies

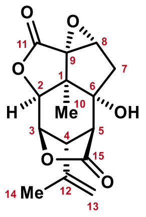

<u>^{1}H NMR</u> (600 MHz, CD_3OD)
δ 5.16 (dd, *J* = 4.7, 3.6 Hz, 1H, H3), 5.06 (d, *J* = 3.6 Hz, 1H, H2), 4.92 (s, 1H, H13), 4.86 (d, *J* = 2.0 Hz, 1H, H13), 3.58 (d, *J* = 3.5 Hz, 1H, H8), 3.51 (s, 1H, H5), 2.91 (d, *J* = 4.7 Hz, 1H, H4), 2.82 (dd, *J* = 14.9, 3.5 Hz, 1H, H7), 1.96 (d, *J* = 14.9 Hz, 1H, H7), 1.89 (s, 3H, H14), 1.17 (s, 3H, H10).

<u>^{13}C NMR</u> (150 MHz, CD_3OD)
δ 176.7 (C15), 171.8 (C11), 141.9 (C12), 112.2 (C13), 86.8 (C6), 81.9 (C2), 79.0 (C3), 73.6 (C9), 63.0 (C8), 52.5 (C4), 49.7 (C5), 47.9 (C1), 45.1 (C7), 23.2 (C14), 16.7 (C10).

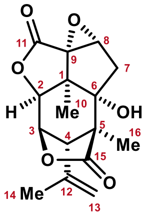

<u>^{1}H NMR</u> (600 MHz, CD_3OD)
δ 5.14 – 5.08 (m, 2H, H13 and H3), 4.97 (m, 2H, H13 and H2), 3.55 (d, *J* = 3.4 Hz, 1H, H8), 3.25 – 3.19 (m, 1H, H4), 2.83 (dd, *J* = 15.3, 3.4 Hz, 1H, H7), 1.95 (s, 3H, H14), 1.76 (d, *J* = 15.3 Hz, 1H, H7), 1.46 (s, 3H, H16), 1.18 (s, 3H, H10).

<u>^{13}C NMR</u> (150 MHz, CD_3OD)
δ 180.1 (C15), 171.9 (C11), 141.4 (C12), 114.7 (C13), 89.4 (C6), 82.5 (C2), 77.8 (C3), 73.8 (C9), 63.4 (C8), 54.8 (C4), 52.5 (C5), 48.7 (C1), 42.3 (C7), 24.2 (C14), 17.4 (C10), 17.2 (C16).

3.1 Methanolysis of PXN under stoichiometric amount of NaOMe in MeOH

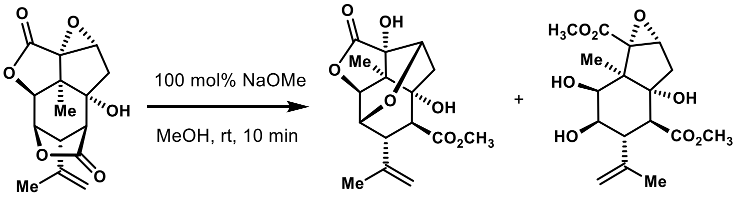

To a solution of PXN (30 mg, 0.1 mmol, 1.0 equiv) in MeOH (2 mL) was added NaOMe (5.4 mg, 0.1 mmol, 1.0 equiv) at room temperature. After starting material consumption monitored by TLC (~10 min), the solvent was removed in *vacuo*. The crude product was purified by column chromatography over silica gel (30% EtOAc/CH_2Cl_2) to give **33** (14 mg, 0.043 mmol, 43%) and **34** (16 mg, 0.045 mmol, 45%).

<u>Characterization data of **33**:</u>
<u>*Description*</u> white solid
<u>R_f</u> 0.2 in 30% EtOAc/CH_2Cl_2. Stains purple in anisaldehyde.

^{1}H NMR (600 MHz, $CDCl_3$)
δ 4.93 (d, *J* = 1.8 Hz, 1H), 4.86 (s, 1H), 4.27 (d, *J* = 2.3 Hz, 1H), 4.18 (d, *J* = 3.8 Hz, 1H), 3.86 (d, *J* = 2.3 Hz, 1H), 3.75 (s, 3H), 3.09 (d, *J* = 14.8 Hz, 1H), 3.06 (d, *J* = 11.4 Hz, 1H), 2.78 (d, *J* = 11.5 Hz, 1H), 2.21 – 2.15 (dd, *J* = 14.4 3.8, Hz, 1H), 1.81 (d, *J* = 1.3 Hz, 3H), 1.25 (s, 3H).

^{13}C NMR (150 MHz, $CDCl_3$)
δ 175.9, 173.2, 141.9, 112.9, 85.6, 81.9, 80.4, 80.0, 72.9, 58.3, 52.6, 48.0, 47.3, 43.4, 22.3, 10.4.

Characterization data of **34:**

Description white solid

R_f 0.35 in 30% EtOAc/CH_2Cl_2. Stains blue in anisaldehyde.

^{1}H NMR (600 MHz, $CDCl_3$)
δ 5.00 (s, 1H), 4.93 (s, 1H), 4.43 (s, 1H), 4.04 (s, 1H), 3.79 (s, 3H), 3.72 (d, *J* = 8.6 Hz, 1H), 3.64 (s, 3H), 3.21 (s, 1H), 2.79 (d, *J* = 15.1 Hz, 1H), 2.75 – 2.68 (m, 3H), 2.65 (s, 1H), 2.05 (d, *J* = 15.1 Hz, 2H), 1.75 (s, 3H), 1.30 (s, 3H).

^{13}C NMR (150 MHz, $CDCl_3$)
δ 172.1, 168.1, 141.8, 117.5, 78.9, 74.2, 68.4, 67.2, 64.2, 52.8, 51.9, 50.9, 50.3, 46.1, 38.3, 18.1, 13.5.

^{1}H NMR (600 MHz, d_6-acetone)
δ 4.80 (s, 1H), 4.73 (s, 1H), 4.31 (dd, *J* = 6.1, 3.0 Hz, 1H), 4.02 (s, 1H), 3.86 (d, *J* = 3.2 Hz, 1H), 3.78 (ddd, *J* = 10.7, 6.2, 2.4 Hz, 1H), 3.71 (s, 3H), 3.58 (d, *J* = 6.3 Hz, 1H), 3.52 (s, 3H), 3.08 (s, 1H), 2.75 (d, *J* = 15.0 Hz, 1H), 2.73 – 2.65 (m, 2H), 1.94 (d, *J* = 15.0 Hz, 1H), 1.73 (s, 3H), 1.29 (s, 3H).

^{13}C NMR (150 MHz, d_6-acetone)
δ 172.7, 168.4, 144.4, 115.2, 79.6, 76.0, 69.6, 67.7, 64.5, 52.4, 51.9, 51.2, 51.1, 46.4, 39.0, 18.9, 13.8.

3.2 Methanolysis of PXN under catalytic amount of NaOMe in CD_3OD

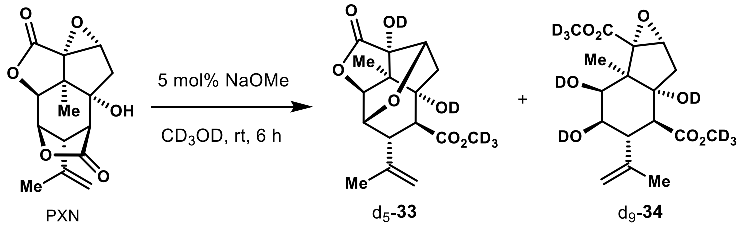

To a solution of PXN (10 mg, 34 μmol, 1.0 equiv) in CD_3OD (0.6 mL) in 5 mm NMR tube was added a solution of NaOMe (20 μL, 0.0085 M in CD_3OD, 1.7 μmol, 0.05 equiv) at room temperature. The reaction was detected by ^{1}H NMR in each 30 min. After 6 hours, the reaction was concentrated under vacuum. The crude product was purified by prep TLC (30% EtOAc/CH_2Cl_2) to give d_5-**33** (4.9 mg, 0.015 mmol, 45%) and d_9-**34** (5.7 mg, 0.016 mmol, 46%).

Characterization data of d_5-**33**:

Description white solid

R_f 0.2 in 30% EtOAc/CH_2Cl_2. Stains purple in anisaldehyde.

^{1}H NMR (600 MHz, $CDCl_3$)
δ 4.93 (s, 1H), 4.86 (s, 1H), 4.27 (d, *J* = 2.3 Hz, 1H), 4.18 (d, *J* = 3.7 Hz, 1H), 3.86 (d, *J* = 2.3 Hz, 1H), 3.13 (s, 1H), 3.09 (d, *J* = 14.8 Hz, 1H), 3.06 (d, *J* = 11.6 Hz, 1H), 2.78 (d, *J* = 11.6 Hz, 1H), 2.44 (s, 1H), 2.18 (dd, *J* = 14.8, 3.7 Hz, 1H), 1.81 (s, 3H), 1.25 (s, 3H).

^{13}C NMR (150 MHz, $CDCl_3$)
δ 175.9, 173.3, 141.9, 112.9, 85.7, 81.9, 80.4, 80.0, 72.9, 58.3, 48.0, 47.3, 43.3, 22.3, 10.5.

^{1}H NMR	(600 MHz, CD_3OD)
	δ 4.91 (s, 2H), 4.34 (d, *J* = 2.3 Hz, 1H), 4.10 (d, *J* = 3.8 Hz, 1H), 3.78 (d, *J* = 2.3 Hz, 1H), 3.05 (d, *J* = 14.7 Hz, 1H), 3.01 (d, *J* = 11.7 Hz, 1H), 2.83 (d, *J* = 11.7 Hz, 1H), 2.17 (dd, *J* = 14.7, 3.8 Hz, 1H), 1.78 (s, 3H), 1.21 (s, 3H).
^{13}C NMR	(150 MHz, CD_3OD)
	δ 177.7, 175.2, 143.8, 113.3, 86.7, 82.5, 81.4, 81.0, 74.8, 59.2, 50.1, 48.6, 42.9, 21.9, 10.9.
LRMS	Calculated $C_{16}H_{18}D_3O_7$ $[M+H]^+$: 328.1, Found: 328.1.

Characterization data of d$_9$-**34**:

Description	white solid
R_f	0.35 in 30% EtOAc/CH_2Cl_2. Stains blue in anisaldehyde.
^{1}H NMR	(600 MHz, d$_6$-acetone)
	δ 4.80 (d, *J* = 1.4 Hz, 1H), 4.73 (d, *J* = 1.5 Hz, 1H), 4.31 (dd, *J* = 6.1, 3.0 Hz, 1H), 4.02 (s, 1H), 3.85 (t, *J* = 2.7 Hz, 1H), 3.78 (ddd, *J* = 10.6, 6.3, 2.4 Hz, 1H), 3.58 (d, *J* = 6.3 Hz, 1H), 3.08 (s, 1H), 2.75 (d, *J* = 15.0 Hz, 1H), 2.71 – 2.65 (m, 2H), 1.94 (d, *J* = 15.0 Hz, 1H), 1.74 (s, 3H), 1.29 (s, 3H).
^{13}C NMR	(150 MHz, d$_6$-acetone)
	δ 172.7, 168.4, 144.4, 115.2, 79.6, 76.0, 69.6, 67.7, 64.5, 51.9, 51.1, 46.4, 39.0, 18.9, 13.8.
^{1}H NMR	(600 MHz, CD_3OD)
	δ 4.78 (d, *J* = 1.1 Hz, 1H), 4.28 (d, *J* = 2.7 Hz, 1H), 4.00 (s, 1H), 3.70 (dd, *J* = 10.6, 2.8 Hz, 1H), 2.82 – 2.62 (m, 3H), 1.93 (d, *J* = 15.0 Hz, 1H), 1.75 (s, 2H), 1.29 (s, 3H).
^{13}C NMR	(150 MHz, CD_3OD)
	δ 174.1, 169.3, 144.4, 115.8, 80.2, 76.2, 70.1, 68.1, 65.0, 52.8, 51.8, 46.5, 39.1, 13.9.
LRMS	Calculated $C_{17}H_{19}D_6O_8$ $[M+H]^+$: 363.2, Found: 363.2.

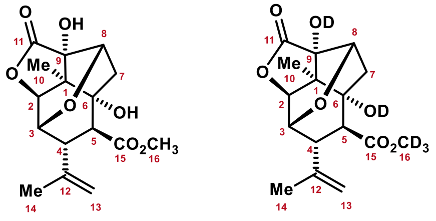

Methyl picrotoxate d$_5$-Methyl picrotoxate

Supplementary Table 1. ^{1}H NMR comparison of reported methyl picrotoxate and d$_5$-methyl picrotoxate in this work

No.	**Natural**[7,a]	**Trost**[6] (300 MHz, $CDCl_3$)	**This work** (Methyl picrotoxate) (600 MHz, $CDCl_3$)	**This work** (d$_5$-Methyl picrotoxate) (600 MHz, $CDCl_3$)
1	-	-	-	-
2	4.22 (d, *J* = 2 Hz, 1H)	4.26 (d, *J* = 2.4 Hz, 1H)	4.27 (d, *J* = 2.3 Hz, 1H)	4.27 (d, *J* = 2.3 Hz, 1H)
3	3.82 (d, *J* = 2 Hz, 1H)	3.86 (d, *J* = 2.2 Hz, 1H)	3.86 (d, *J* = 2.3 Hz, 1H)	3.86 (d, *J* = 2.3 Hz, 1H)
4	2.98 (d, *J* = 12 Hz, 1H)	3.08 (d, *J* = 11.3 Hz, 1H)	3.06 (d, *J* = 11.4 Hz, 1H)	3.06 (d, *J* = 11.6 Hz, 1H)
5	2.80 (d, *J* = 12 Hz, 1H)	2.77 (d, *J* = 11.3 Hz, 1H)	2.78 (d, *J* = 11.5 Hz, 1H)	2.78 (d, *J* = 11.6 Hz, 1H)
6	1.43 (s, 1H)	-	-	-
7	3.09 (d, *J* = 15 Hz, 1H); 2.19 (dd, *J* = 15, 3 Hz, 1H)	3.10 (d, *J* = 14.8 Hz, 1H); 2.14-2.20 (dd, *J* = 14.0, 3.9 Hz, 1H)	3.09 (d, *J* = 14.8 Hz, 1H); 2.15 – 2.21 (dd, *J* = 14.4 3.8, Hz, 1H)	3.09 (d, *J* = 14.8 Hz, 1H); 2.18 (dd, *J* = 14.8, 3.7 Hz, 1H)
8	4.17 (d, *J* = 3 Hz, 1H)	4.11 (d, *J* = 3.6 Hz, 1H)	4.18 (d, *J* = 3.8 Hz, 1H)	4.18 (d, *J* = 3.7 Hz, 1H)
9	-	-	-	-
10	1.21 (s, 3H)	1.25 (s, 3H)	1.25 (s, 3H)	1.25 (s, 3H)
11	-	-	-	-
12	-	-	-	-

13	4.87 (s, 1H); 4.83 (s, 1H)	4.93 (s, 1H); 4.86 (s, 1H)	4.93 (d, J = 1.8 Hz, 1H), 4.86 (s, 1H)	4.93 (s, 1H); 4.86 (s, 1H)
14	1.76 (s, 3H)	1.81 (s, 3H)	1.81 (d, J = 1.3 Hz, 3H)	1.81 (s, 3H)
15	-	-	-	-
16	3.69 (s, 3H)	3.75 (s, 3H)	3.75 (s, 3H)	Not found

[a]NMR field and solvent were not reported.

Supplementary Table 2. ^{13}C NMR comparison of reported methyl picrotoxate and d$_5$-methyl picrotoxate in this work

No.	**Natural**[7,a]	**Trost**[6] (75 MHz, $CDCl_3$)	**This work** (Methyl picrotoxate) (150 MHz, $CDCl_3$)	**This work** (d$_5$-Methyl picrotoxate) (150 MHz, $CDCl_3$)
1	58.20	58.1	58.3	58.3
2	81.85	81.7	81.9	81.9
3	79.70*	72.7	72.9	72.9
4	47.20	47.8	48.0	48.0
5	47.80	47.0	47.3	47.3
6	85.50	80.3	80.4	80.4
7	42.90	43.0	43.4	43.3
8	72.80*	79.6	80.0	80.0
9	80.30	85.5	85.6	85.7
10	10.35	10.3	10.4	10.5
11	176.10	175.9	175.9	175.9
12	141.70	141.6	141.9	141.9
13	112.80	112.8	112.9	112.9
14	22.10	22.1	22.3	22.3
15	173.30	173.2	173.2	173.3
16	52.30	52.4	52.6	Not found

[a]NMR field and solvent were not reported. *misassigned carbons at C3 and C8.

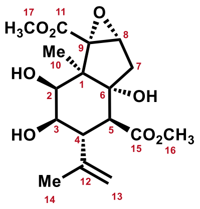

Dimethyl picrotoxinindicarboxylate

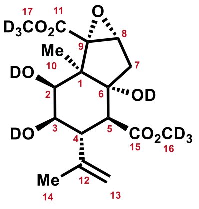

d$_9$-Dimethyl picrotoxinindicarboxylate

Supplementary Table 3. ^{1}H NMR comparison of reported dimethyl picrotoxinindicarboxylate and d$_9$-dimethyl picrotoxinindicarboxylate in this work

No.	**Trost**[6] (400 MHz, d$_6$-acetone)	**This work** (Dimethyl picrotoxinindicarboxylate) (600 MHz, d$_6$-acetone)	**This work** (d$_9$-Dimethyl picrotoxinindicarboxylate) (600 MHz, d$_6$-acetone)
1	-	-	-
2	3.87 (d, J = 3.3 Hz, 1H)	3.86 (d, J = 3.2 Hz, 1H)	3.85 (t, J = 2.7 Hz, 1H)
3	4.30 (dd, J_1 = J_2 = 3.0 Hz, 1H)	4.31 (dd, J = 6.1, 3.0 Hz, 1H)	4.31 (dd, J = 6.1, 3.0 Hz, 1H)
4	2.68 (m, 1H)	2.65 – 2.73 (m, 2H)	2.67-2.72 (m, 2H)
5			
6	-	-	-
7	2.73 (d, J = 15.0 Hz, 1H); 1.92 (d, J = 15.0 Hz, 1H)	2.75 (d, J = 15.0 Hz, 1H); 1.94 (d, J = 15.0 Hz, 1H)	2.75 (d, J = 15.0 Hz, 1H); 1.94 (d, J = 15.0 Hz, 1H)
8	4.01 (s, 1H)	4.02 (s, 1H)	4.02 (s, 1H)
9	-	-	-
10	1.61 (s, 3H)	1.29 (s, 3H)	1.29 (s, 3H)
11	-	-	-

12	-	-	-
13	4.79 (s, 1H); 4.71 (s, 1H)	4.80 (s, 1H); 4.73 (s, 1H)	4.80 (s, 1H); 4.73 (s, 1H)
14	1.72 (s, 3H)	1.73 (s, 3H)	1.73 (s, 3H)
15	-	-	-
16	3.70 (s, 3H)	3.71 (s, 3H)	Not found
17	3.51 (s, 3H)	3.52 (s, 3H)	Not found

Supplementary Table 4. ^{13}C NMR comparison of reported dimethyl picrotoxinindicarboxylate and d_9-dimethyl picrotoxinindicarboxylate in this work

No.	**Trost**[6] (75 MHz, d_6-acetone)	**This work** (Dimethyl picrotoxinindicarboxylate) (150 MHz, d_6-acetone)	**This work** (d_9-Dimethyl picrotoxinindicarboxylate) (150 MHz, d_6-acetone)
1	50.8	51.1	51.1
2	75.7	76.0	76.0
3	69.4	69.6	69.6
4	46.1	46.4	46.4
5	51.7	51.9	51.9
6	79.4	79.6	79.6
7	38.7	39.0	39.0
8	64.3	64.5	64.5
9	67.4	67.7	67.7
10	13.6	13.8	13.8
11	168.2	168.4	168.4
12	144.2	144.4	144.4
13	114.9	115.2	115.2
14	18.6	18.9	18.9
15	172.5	172.7	172.7
16	51.0	51.2	Not found
17	52.2	52.4	Not found

3.3 Competitive methanolysis of PXN and 5MePXN under 5 mol% NaOMe in CD_3OD

To a mixture of PXN (1 mg, 0.0034 mmol) and 5MePXN (1 mg, 0.0033 mmol) in CD_3OD (0.1 mL) in 3 mm NMR tube was added a solution of NaOMe (10 µL, 0.017 M in CD_3OD, 0.05 equiv) at room temperature. NMR tube was shaken bottom up for 5 times to make solution homogenous, then NMR experiment was running in 5 min.

d_5-**33**

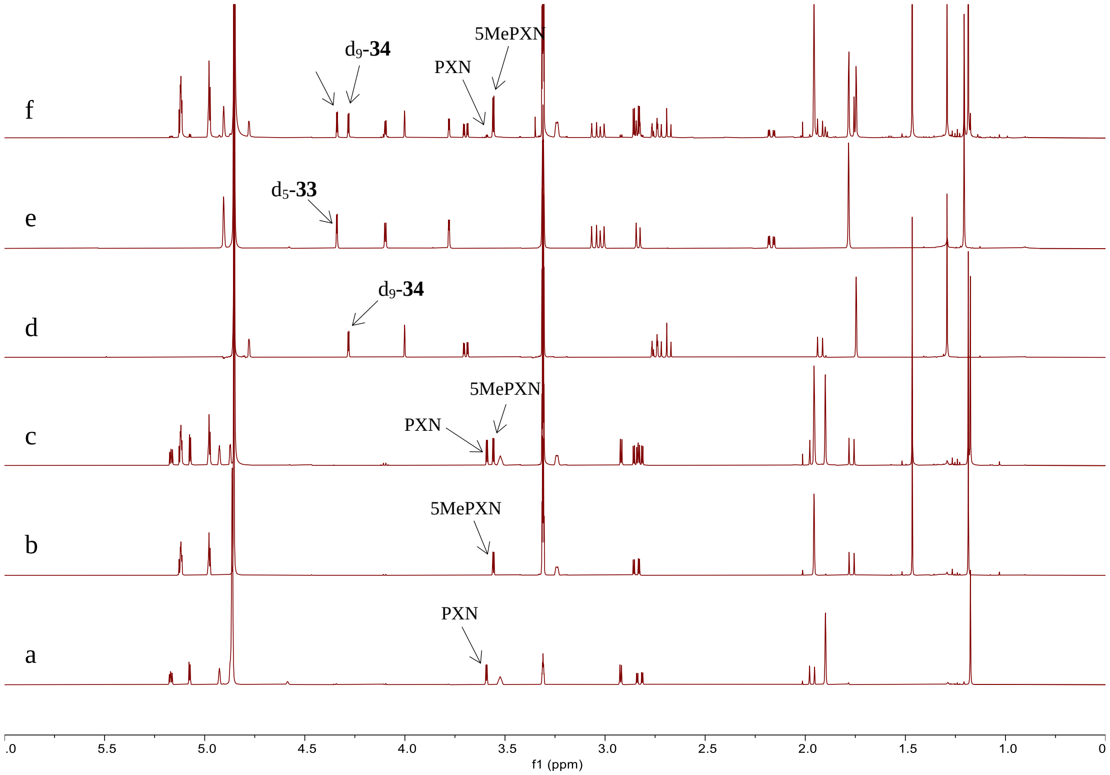

Supplementary Figure 3. Stacked NMR spectra for analyzing methanolysis of PXN and 5MePXN. (a) PXN, (b) 5MePXN, (c) 1:1 mixture of PXN and 5MePXN, (d) d_9-**33**, (e) d_9-**34**, (f) Treatment of 1:1 mixture of PXN and 5MePXN with 5 mol% NaOMe in 280 min.

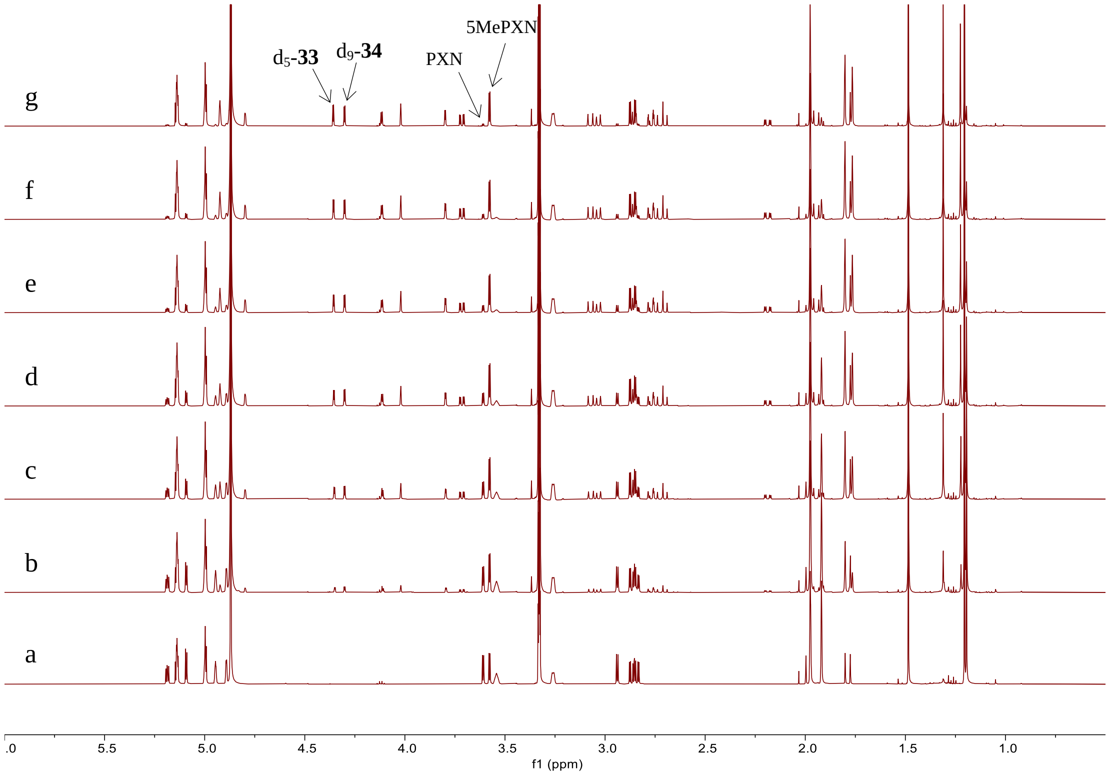

Supplementary Figure 4. Competitive methanolysis of PXN and 5MePXN under 5 mol% NaOMe in CD_3OD. (a) 0 min, (b) 10 min, (c) 30 min, (d) 60 min, (e) 100 min, (f) 140 min, (g) 280 min.

Supplementary Table 5. Concentration of each component in competitive methanolysis.

Time (min)	[PXN] (mM)	[d_5-**33**] (mM)	[d_9-**34**] (mM)	[5MePXN] (mM)
0	3.4246	0	0	3.2679738
5	2.952241379	0.23617931	0.23617931	3.2679738
10	2.499708029	0.449947445	0.474944526	3.2679738
15	2.181273885	0.588943949	0.654382166	3.2679738
20	1.93480226	0.715876836	0.773920904	3.2679738
25	1.67872549	0.839362745	0.906511765	3.2679738
30	1.563744292	0.891334247	0.969521461	3.2679738
35	1.495458515	0.927184279	1.001957205	3.2679738
40	1.463504274	0.936642735	1.024452991	3.2679738
45	1.375341365	0.976492369	1.072766265	3.2679738
50	1.307099237	1.019537405	1.097963359	3.2679738
55	1.223071429	1.064072143	1.137456429	3.2679738
60	1.119150327	1.107958824	1.19749085	3.2679738
65	1.044085366	1.138053049	1.242461585	3.2679738
70	0.961966292	1.183218539	1.279415169	3.2679738
75	0.925567568	1.203237838	1.295794595	3.2679738
80	0.85615	1.2414175	1.3270325	3.2679738
85	0.800140187	1.264221495	1.360238318	3.2679738
90	0.773047404	1.275528217	1.376024379	3.2679738
95	0.730191898	1.307043497	1.387364606	3.2679738
100	0.704650206	1.317695885	1.402253909	3.2679738
110	0.611535714	1.363724643	1.449339643	3.2679738
120	0.560490998	1.390017676	1.474091326	3.2679738
130	0.514977444	1.400738647	1.50888391	3.2679738
140	0.466566757	1.427694278	1.530338965	3.2679738
280	0.2157908	1.55585167	1.65295753	3.2679738

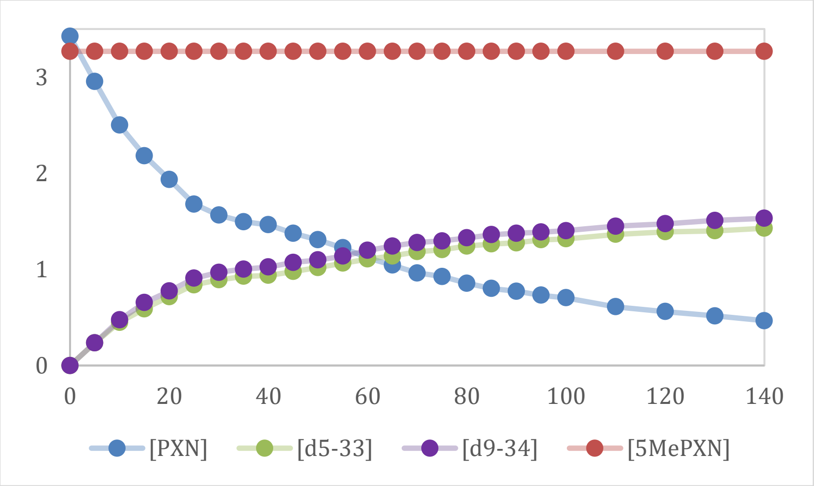

Supplementary Figure 5. Concentration of PXN, d_5-**33**, d_9-**34** and 5MePXN vs. time (min)

3.4 Methanolysis of 5MePXN under different equivalent of NaOMe in CD_3OD

To a solution of 5MePXN (2.5 mg, 0.0082 mmol) in CD_3OD (0.4 mL) in 5 mm NMR tube was added a solution of NaOMe in CD_3OD (0.1, 0.2, 0.5, 0.8, 1.0, 1.5 and 2.0 equiv) at room temperature. The NMR tube was shaken bottom up 5 times to make solution homogenous, then measured by ^{1}H NMR.

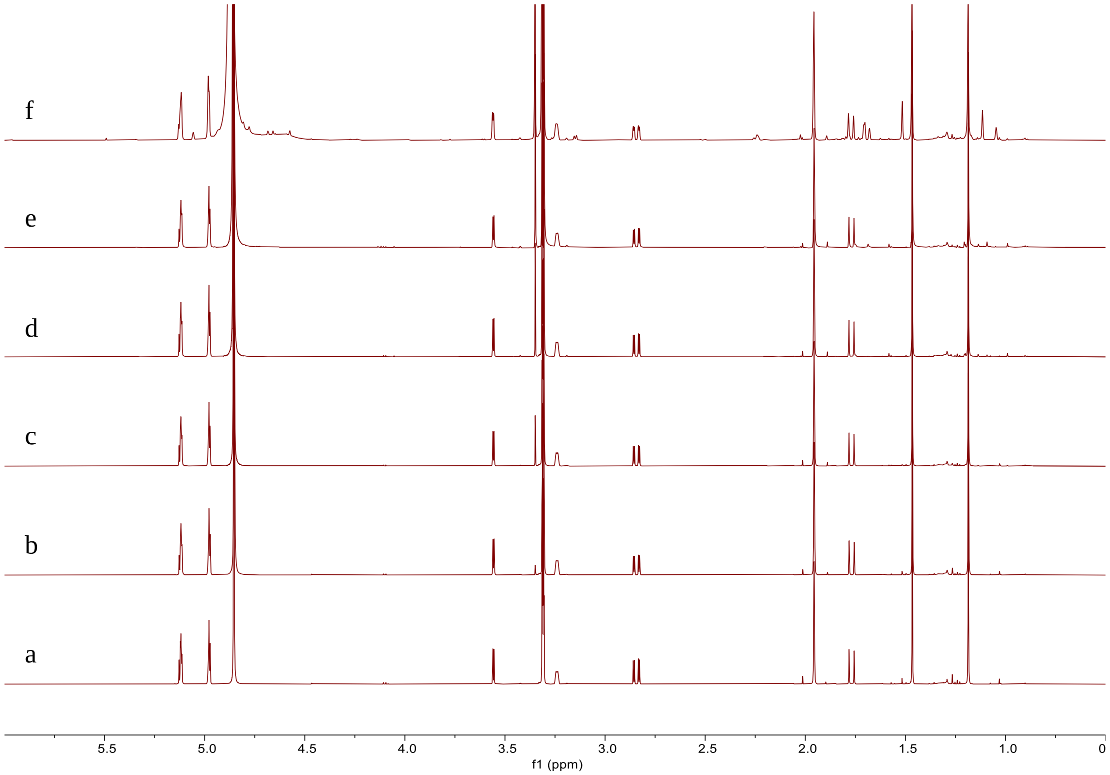

Supplementary Figure 6. Treatment of 5MePXN with different equivalent NaOMe in CD_3OD. (a) 5MePXN, (b) 0.1 equiv NaOMe, (c) 0.5 equiv NaOMe, (d) 1.0 equiv NaOMe, (e) 2.0 equiv NaOMe, (f) 2.0 equiv NaOMe after 109 days.

3.5 Competitive degradation of PXN and 5MePXN under dilute acidic solution

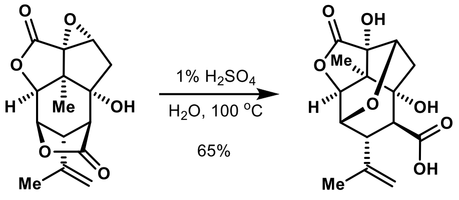

Acidic degradation of PXN was running according to previous literature.[8] PXN (10 mg, 0.034 mmol) was refluxed in 1% H_2SO_4 in H_2O (1 mL) at 100 ºC for 24 h. When the reaction mixture was cooled to room temperature, 5 mL EtOAc was added while vigorous stirring, then organic layer was separated. The aqueous layer was extracted with EtOAc (2 x 5 mL). The combined organic layer was dried over Na_2SO_4, and concentrated *in vacuo* to yield a mixture of PXN and picrotoxic acid. PXN could be further separated by washing with $CHCl_3$ (3 x 5 mL). The undissolved white solid that was soluble in d_6-acetone was identified as picrotoxic acid **32** (7 mg, 0.022 mmol, 65%).

5MePXN (10 mg) was treated under the same conditions. No degradation was observed based on crude ^{1}H NMR. 5MePXN was fully recovered after above workup.

3.6 Stability of PXN and 5MePXN in mouse and human plasma.

Stability experiments of PXN and 5MePXN in 2 species of plasma were conducted by WuXi AppTec Co. Ltd.(Shanghai), China.

Materials

Test compound working solution: 5 µL of tested compound stock solution (10 mM in DMSO) were diluted with 495 µL of DMSO (concentration: 100 µM, 100% DMSO). Propantheline bromide working solution: 5 µL of propantheline bromide stock solution (10 mM in DMSO) were diluted with 495 µL of H_2O (concentration: 100 µM, 1% DMSO)

Supplementary Table 6. Test system.

Species/ Matrix	Minimum No. of individuals		Anticoagulant Used	Vendor
CD-1 Mouse Plasma	20 Male		EDTA-K2	Beijing Vital River Laboratory Animal Technology
Human Plasma	3 Male & 3 Female		EDTA-K2	BioreclamationIVT

Method

The pooled frozen plasma was thawed in a water bath at 37 ºC prior to experiment. Plasma was centrifuged at 1968 g (4000 rpm) for 5 min and the clots were removed. Using an Apricot automation work station, 98 µL/well of blank plasma were added to all 96-well reaction plates. (Blank, T0, T10, T30, T60 and T120). An Apricot automation workstation was used to add 2 µL/well of working solution (100 µM) to all reaction plates except Blank. (T0, T10, T30, T60 and T120). All reaction plates containing mixtures of compound and plasma were incubated at 37 ºC in water bath. The reaction plates were incubated at 37 ºC, and timer was started. At the end of incubation, 400 µL of stock solution was added (200 ng/mL tolbutamide and 200 ng/mL labetalol in MeCN) to precipitate protein and mixed thoroughly. Each plate was sealed and shaken for 20 minutes, then centrifuged at 1968 g (4000 rpm) and 4 ºC for 20 minutes. After centrifugation, an Apricot automation workstation was used to transfer 150 µL supernatant. Each bioanalysis plate was sealed and shaken for 10 minutes prior to LC-MS/MS analysis.

Data analysis

The % remaining of test compound after incubation in plasma was calculated using following equation:
% Remaining= 100 x (PAR at appointed incubation time / PAR at T0 time)
where PAR is the peak area ratio of analyte versus internal standard (IS)
The appointed incubation time points are T0 (0 min), Tn (n=0, 10, 30, 60, 120 min)

Supplementary Table 7. %Remaining of test compound in two plasmas.

Compound	Time point (min)	% Remaining		$T_{½}$ (min)	
		Mouse	Human	Mouse	Human
PXN	0	100.0	100.0	97.3	108.3
	10	102.5	130.2		
	30	87.1	113.6		
	60	67.4	72.9		
	120	44.7	55.8		
5MePXN	0	100.0	100.0	206.9	289.1
	10	97.9	103.2		
	30	87.9	93.7		
	60	75.7	84.5		
	120	68.0	77.7		

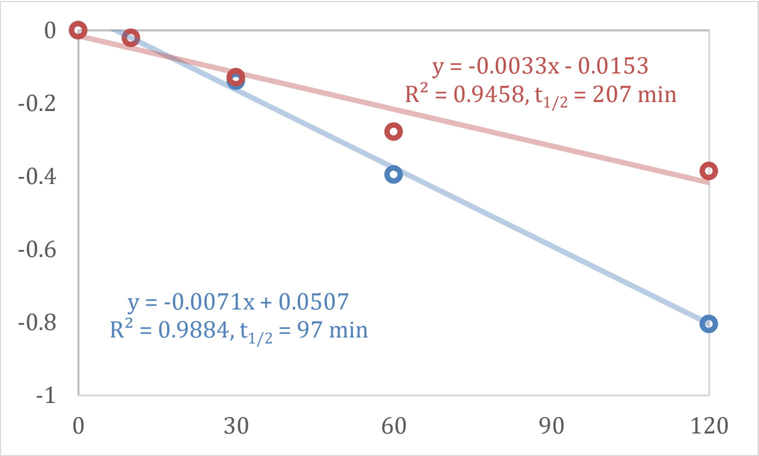

Supplementary Figure 7. Ln (%Remaining) of PXN (blue) & 5MePXN (red) vs. time (min) in mouse plasma

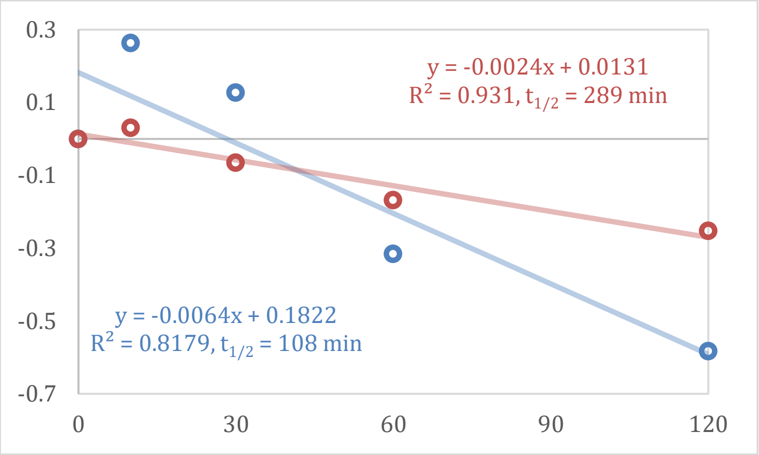

Supplementary Figure 8. Ln (%Remaining) of PXN (blue) & 5MePXN (red) vs. time (min) in human plasma

4. Rat $GABA_A$ receptors binding assay

Non-selective rat $GABA_A$ ion channel [3H] TBOB binding (Antagonist Radioligand) assay was conducted by Eurofins Pharma Discovery Service of Eurofins Cerep, France.

Method

Compound binding was calculated as a % inhibition of the binding of the radioactively labeled ligand [3H] TBOB (*t*-[^{3}H]Butylbicycloorthobenzoate) for rat $GABA_A$ ion channels.[9] A 20.0 mM stock solution was prepared in DMSO from each test compound to evaluate radioligand displacement of [3H] TBOB from rat cerebral cortex $GABA_A$ receptors at different concentrations.

Data analysis

The results are expressed as a percent of control specific binding

$$\frac{\text{measured specific binding}}{\text{control specific binding}} * 100$$

and as a percent inhibition of control specific binding

$$100 - \left(\frac{\text{measured specific binding}}{\text{control specific binding}} * 100\right)$$

obtained in the presence of the test compounds.
The IC_{50} values (concentration causing a half-maximal inhibition of control specific binding) and Hill coefficients (nH) were determined by non-linear regression analysis of the competition curves generated with mean replicate values using Hill equation curve fitting

$$Y=D+\left[\frac{A-D}{1+(C/C_{50})^{nH}}\right]$$

where Y = specific binding, A = left asymptote of the curve, D = right asymptote of the curve, C = compound concentration, C_{50} = IC_{50}, and nH = slope factor. This analysis was performed using software developed at Cerep (Hill software) and validated by comparison with data generated by the commercial software SigmaPlot® 4.0 for Windows® (© 1997 by SPSS Inc.).
The inhibition constants (K_i) were calculated using the Cheng Prusoff equation

$$K_i=\frac{IC_{50}}{(1+L/K_D)}$$

where L = concentration of radioligand in the assay, and K_D = affinity of the radioligand for the receptor. A scatchard plot is used to determine the K_D.

Supplementary Table 8. IC_{50} values of test compound

Compound	IC_{50} (μM)	Compound	IC_{50} (μM)
PXN (**1**)[10]	0.21	5MePXN (**2**)	1.8
PTN (**10**)	54	5MePTN (**11**)	>200
12FPXN (**12**)	5.4	12F5MePXN (**13**)	100
CypPXN (**14**)	0.39	Cyp5MePXN (**15**)	7.7
12MePXN (**16**)	0.31	12Me5MePXN (**17**)	7.4
8,9doPXN (**18**)	0.55	8,9do5MePXN (**19**)	9.2
HPXN (**20**)	1.0	H5MePXN (**21**)	23
6FPXN (**22**)	0.43	6F5MePXN (**23**)	14
6FPTN (**24**)	56	6F5MePTN (**25**)	>200
6FHPXN (**26**)	6.5	6FH5MePXN (**27**)	52
6F12FPXN (**28**)	32	6F12F5MePXN (**30**)	>200

5. RDL receptors binding assay

Method

The *Drosophila* RDL sequence (NP_01261616.1) was cloned into the pGH19 backbone via Gibson assembly for capped cRNA synthesis using the T7 mMessage mMachine kit (Invitrogen). cRNA samples were stored at -80 ºC until use. Ready to inject *Xenopus* oocytes were obtained from Ecocyte (Austin, TX) and microinjected using a Nanoinject III (Drummond Scientific) with 4 nL of cRNA (1 μg/μL). The oocytes were then incubated at 16 ºC in ND96 storage solution (93.5 mM NaCl, 2 mM KCl, 1.8 mM $CaCl_2·2H_2O$, 2 mM $MgCl_2·6H_2O$, 5 mM HEPES at pH 7.5). Recording were made 1-6 days post injection.

Electrophysiology experiments were performed using an automated two-electrode voltage clamp set-up (Roboocyte 2, Mutlichannel Systems). Recording head were filled with 3M KCl and gave resistance of 100-2000 kΩ in medium. Membrane potential was held at -60 mV and current signal was sampled at 20 kHz. Experiments were performed at room temperature using Modified Barth's Saline (MBS) (88 mM NaCl, 1 mM KCl, 2.4 mM $NaHCO_3$, 15 mM HEPES, 0.3 mM $Ca(NO_3)_2$, 0.41 mM $CaCl_2$, and 0.82 mM $MgSO_4·7H_2O$ at pH 7.4). Oocytes were perfused with MBS at 5 mL/min in all assays. GABA and all compounds were dissolved in MBS and a 0.1% DMSO negative control was utilized to correct for any solvent effect.

Initial block potential of each compound was assessed utilizing a pre-incubation method (MBS (30 sec), 10 μM GABA (15 sec), MBS wash (60 sec), compound (60 sec), compound + 10 μM GABA (15 sec), compound (60 sec), compound + 10 μM GABA (15 sec), MBS wash (60 sec) with 10 μM of each test compound. The maximum signal of the second co-application was compared to the initial GABA response to determine the % block and then standardized against the corresponding value of the 0.1% DMSO control. Dose responses were performed for any compound showing >50% block in the initial test using 10, 1, and 0.1 μM. The IC_{50} values were fit using an inhibitor vs response 4-parameter fit (GraphPad Prism 8.1.2).

Supplementary Table 9. IC_{50} values of test compound

Compound	IC_{50} (μM)	Compound	IC_{50} (μM)
PXN (**1**)	0.7998	5MePXN (**2**)	1.71
PTN (**10**)	0.834	5MePTN (**11**)	>10
12FPXN (**12**)	>10	12F5MePXN (**13**)	>10
CypPXN (**14**)	0.6931	Cyp5MePXN (**15**)	1.615
12MePXN (**16**)	0.6763	12Me5MePXN (**17**)	2.878
8,9doPXN (**18**)	2.603	8,9do5MePXN (**19**)	4.41
HPXN (**20**)	1.075	H5MePXN (**21**)	>10
6FPXN (**22**)	1.046	6F5MePXN (**23**)	0.593
6FPTN (**24**)	4.918	6F5MePTN (**25**)	1.55
6FHPXN (**26**)	0.561	6FH5MePXN (**27**)	2.743
6F12FPXN (**28**)	2.239	6F12F5MePXN (**30**)	0.994

6. Computational models for stability

6.1 Computational methods

All calculations were performed with Gaussian 16.[11] All molecular geometries were optimized using the ωB97X-D[12] functional with the def2-SVP basis set for all atoms. Frequency calculations were performed at the same level of theory as that used for geometry optimization to confirm the stationary points as either minima (no negative frequencies) or saddle points (one negative frequency) on the potential energy surface. Thermal contributions to free energies were calculated from the vibrational frequencies using the quasi-rigid rotor-harmonic oscillator (RRHO) approach proposed by Grimme[13] and implemented through the GoodVibes[14] Python script. Intrinsic Reaction Coordinate (IRC) calculations were performed to confirm the saddle points as real transition states connecting the expected reactants and products. Single-point energies were calculated using the ωB97X-D functional with the def2-TZVPPD basis set for all atoms. Solvation effects were incorporated using the SMD[15] model in both geometry optimizations and single-point energy calculations with water as the solvent. Molecular visualizations were created using CYLview.[16] Conformational searches were performed with the MMFF force field as implemented in Spartan '20[17] to ensure that the lowest-energy conformers are presented in the manuscript. All conformers within 10.0 kcal/mol of the lowest-energy conformation were re-optimized at the DFT levels of theory described above.

Translational entropy corrections were applied to the calculated free energies to account for the concentration gradients in the methanolysis reactions according to the equation[18] (eq 1)

$$\Delta G = \Delta G° + RT \ln Q$$

where $\Delta G°$ is the computed free energy, R is the gas constant (= 1.987 cal·K^{-1}·mol^{-1}), T is the reaction temperature in Kelvin. The reaction quotient Q is defined as being equal to [P]/[R], with [P] and [R] representing the concentrations of the product and the reactant, respectively. In our calculations, we assumed [P] to be equal to the concentration of the limiting reactant, while [R] was the concentration of the reactant present in excess. For instance, the effective Q value for the reaction between picrotoxinin (PXN) and methoxide was 1/20 due to methoxide being used at 5 mol% catalyst loading, while the overall methanolysis reaction between PXN and methanol to yield product **D** has a Q value of about 1/726. In both cases, the concentration gradient resulted in a decrease in the free energies of the products and bimolecular transition states, in line with Le Chatelier's principle.

6.2 Calculated energies

Supplementary Table 10. Calculated energies in Hartrees.

	**ΔG [ωB97X-D/def2-SVP, SMD (H_2O)]**	**E [ωB97X-D/def2-TZVPPD, SMD (H_2O)]**	**G (ΔG + E)**	**Imaginary Frequency (cm^{-1})**
**MeO$^-$**	0.014094	-115.239382	-115.225288	-
**MeOH**	0.029380	-115.745505	-115.716125	-
**PXN**	0.256136	-1032.655892	-1032.399756	-
**5MePXN**	0.282502	-1071.972954	-1071.690452	-
**TS-1**	0.289722	-1147.894677	-1147.604955	-101.86
**A**	0.295026	-1147.909903	-1147.614877	-
**B**	0.290654	-1147.915972	-1147.625318	-

**TS-2**	0.289739	-1147.892628	-1147.602889	-607.08
**C**	0.355751	-1264.180870	-1263.825119	-
**D**	0.307361	-1148.452589	-1148.145228	-
**TS-3**	0.317047	-1187.206922	-1186.889875	-118.59
**E**	0.322067	-1187.221335	-1186.899268	-
**F**	0.319031	-1187.220431	-1186.901400	-
**TS-4**	0.318125	-1187.199616	-1186.881491	-604.84
**G**	0.382366	-1303.481967	-1303.099601	-
**H**	0.335985	-1187.761174	-1187.425189	-

7. Computational models for RDL selectivity

The helical region of Human $GABA_A$ receptors with picrotoxinin bound (PDB 6X40)[19] was used as a template structure to build homology models of GABA in *R. norvegicus* and Rdl in *D. melanogaster*. Two sequences of the β-unit in *D. melanogaster* Rdl (NCBI AAA28559, UniProt P25123) were analyzed for similarity to the template structure. The NCBI sequence was used to build the Rdl model due to greater homology with each chain of the template structure. Protein structures were prepared and protonation issues were fixed using Structure Preparation in MOE.[20]

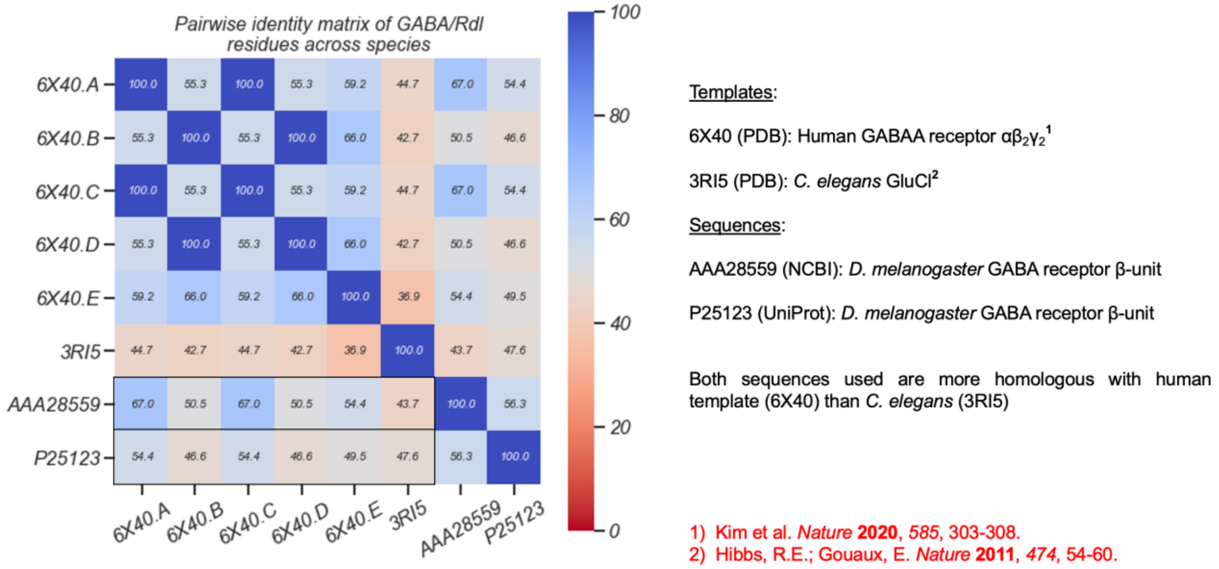

Supplementary Figure 9. Pairwise identity matrix of GABA/Rdl residues across species.

Initially, 50 poses each of PXN (**1**) and 6F12F5MePXN (**30**) were generated in each homology model using the Triangle Matcher placement algorithm with the London dG scoring function. Subsequent refinement with the Induced Fit algorithm and GBVI/WSA dG scoring function narrowed down the set to 20 final poses of each ligand. No external constraints were applied to influence the binding poses.

Protein-ligand complexes were subsequently energy minimized and equilibrated using the ff14SB force field[21] within the Sander module of Amber21.[22] These systems were gradually heated from a temperature of 10 K to 298.15 K in 50 ps using an implicit solvent Generalized-Born Surface-Area (GBSA) model. A time step of 2.0 fs was used with a cutoff of 12 Å for nonbonded interactions. The SHAKE algorithm was employed to keep all bonds involven hydrogen atoms rigid. Long-range interactions were handled using the particle mesh Ewald algorithm. During the energy minimization and MD simulations, only the ligand and residue side chains in the binding pocket were permitted to move. During the 50 ps MD simulations, the coordinates of the system were collected every 0.4 ps. The stable MD trajectory obtained for each complex was used to estimate the binding free energy by using the MM-GBSA module within AMBER. Error bars in the relative binding free energies were computed by combining the standard deviation values for each complex ($s_{\Delta G}$) with that of bound PXN ($s_{\Delta G,ref}$) in each organism. (Eq 2)

$$\Delta\Delta G_{err} = \sqrt{\left(s_{\Delta G}^2 + s_{\Delta G,ref}^2\right)}$$

8. NMR spectra

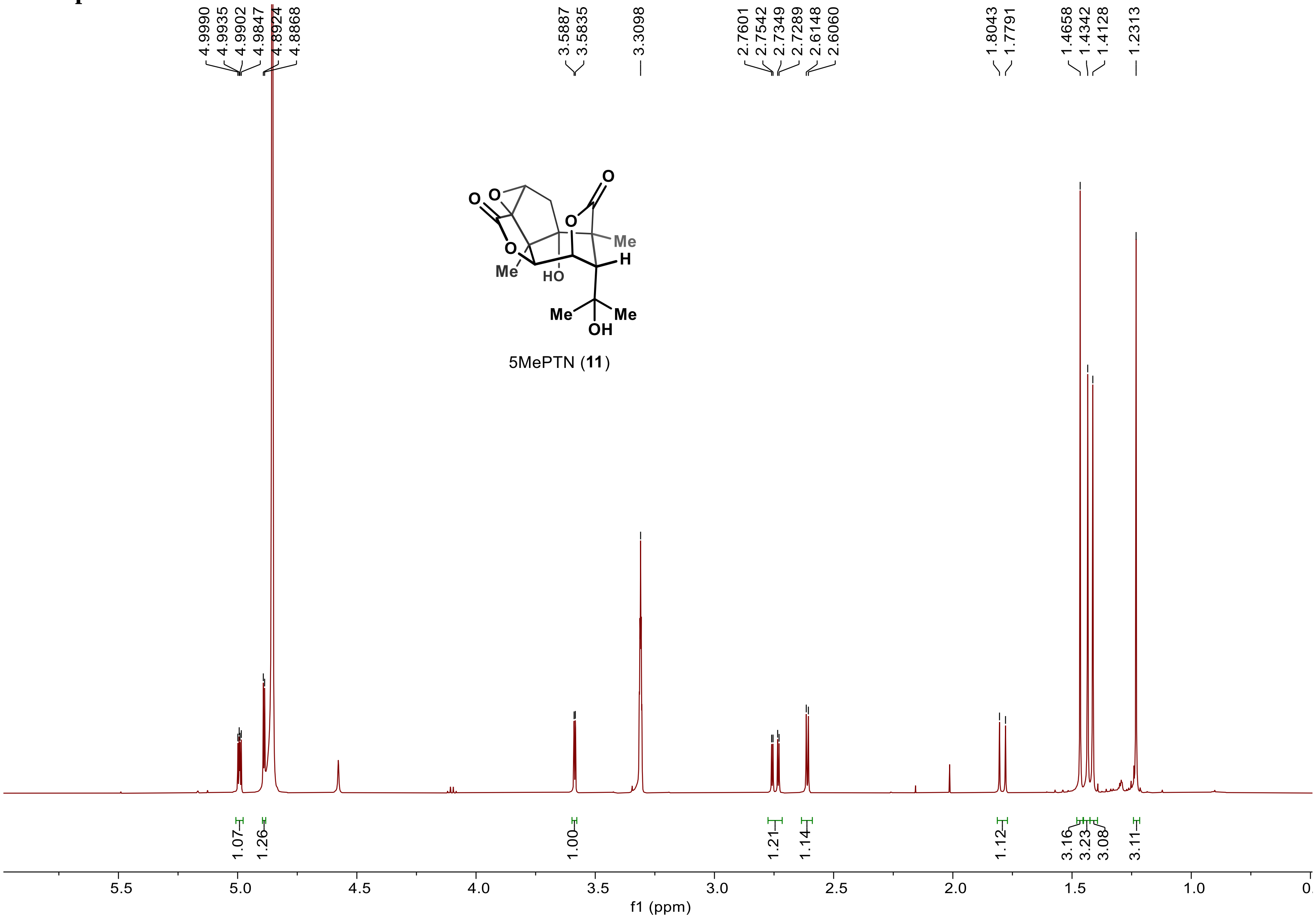

^1H NMR, CD_3OD, 600 MHz

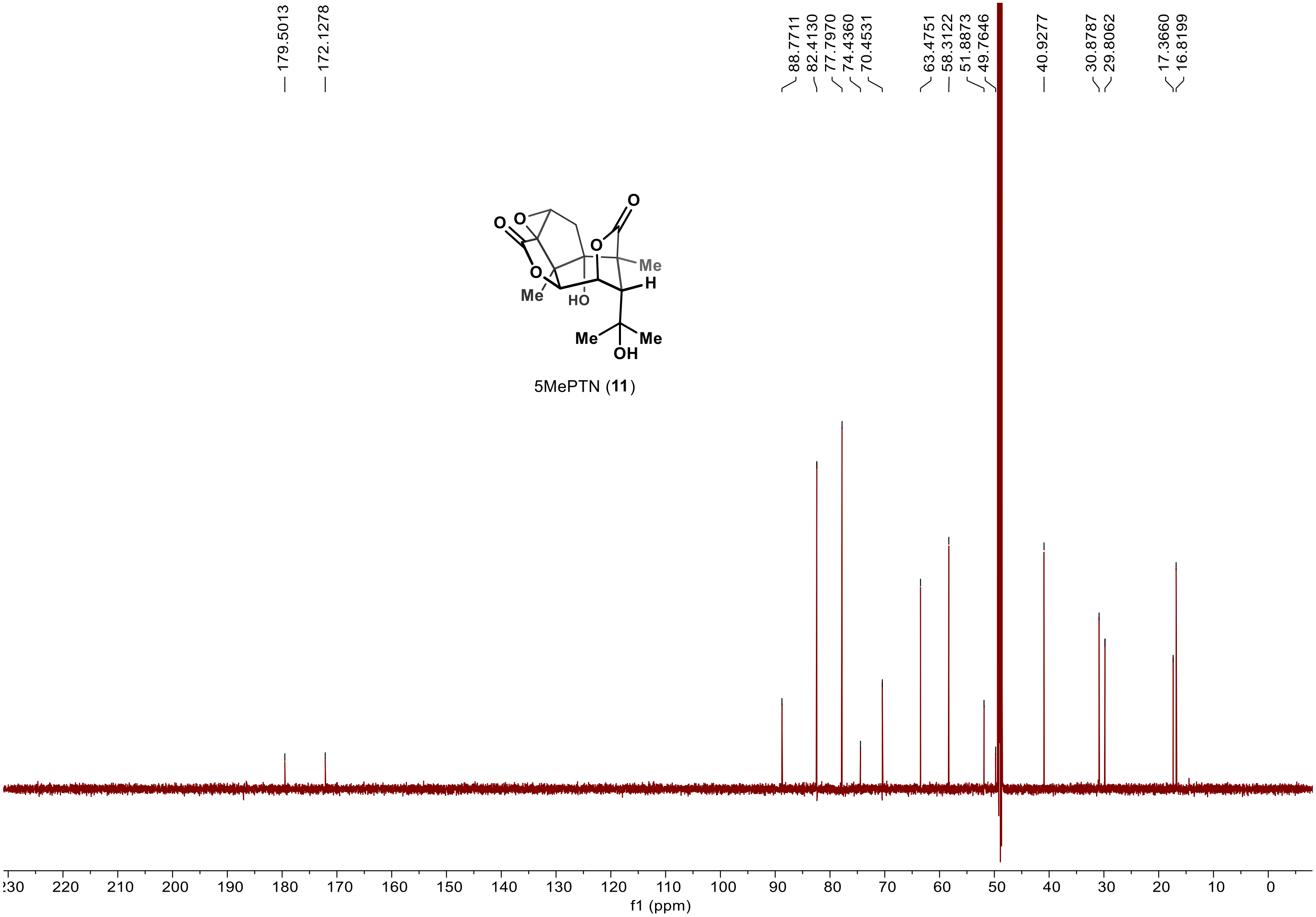

^{13}C NMR, CD_3OD, 150 MHz

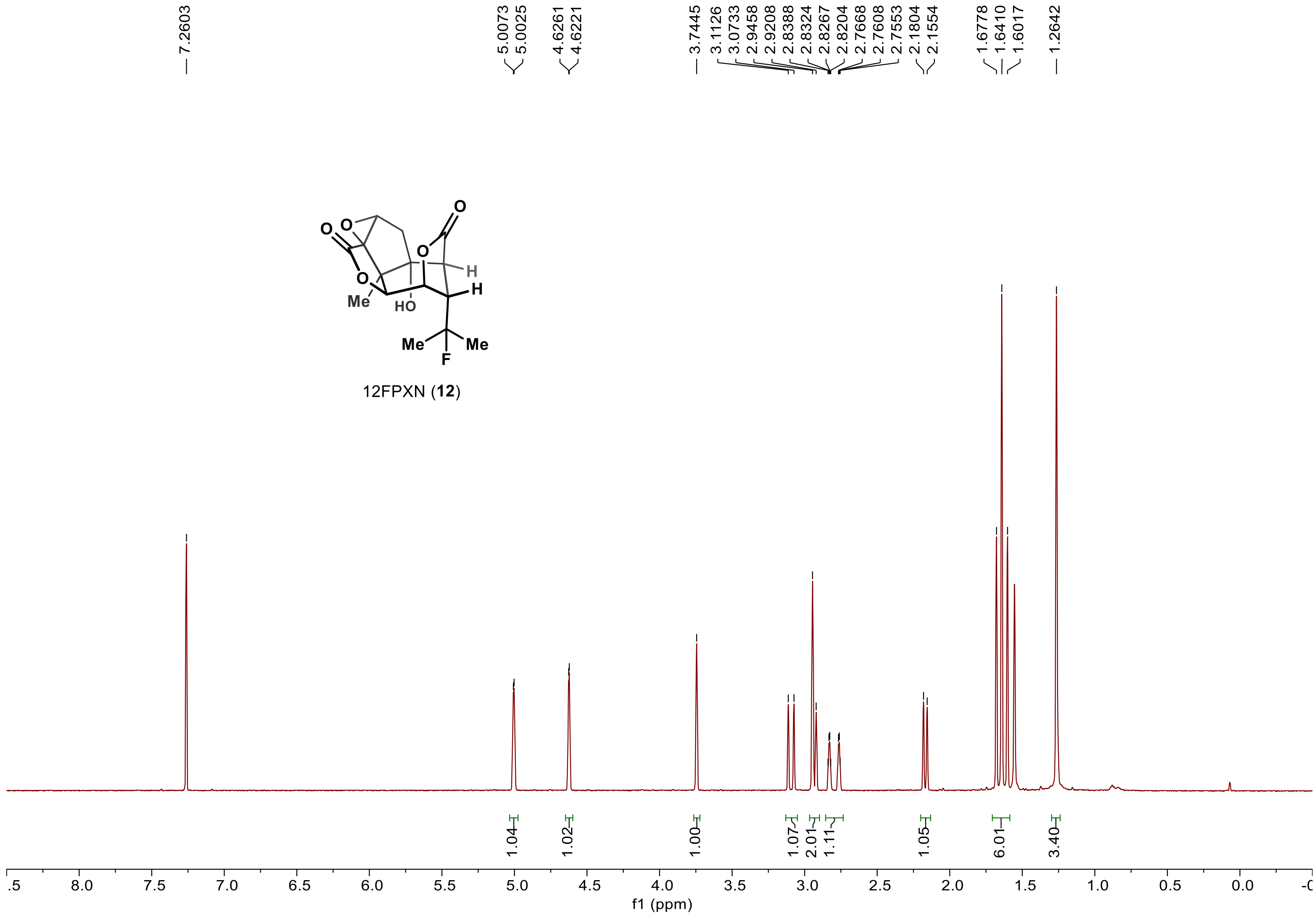

12FPXN (**12**)

^{1}H NMR, $CDCl_3$, 600 MHz

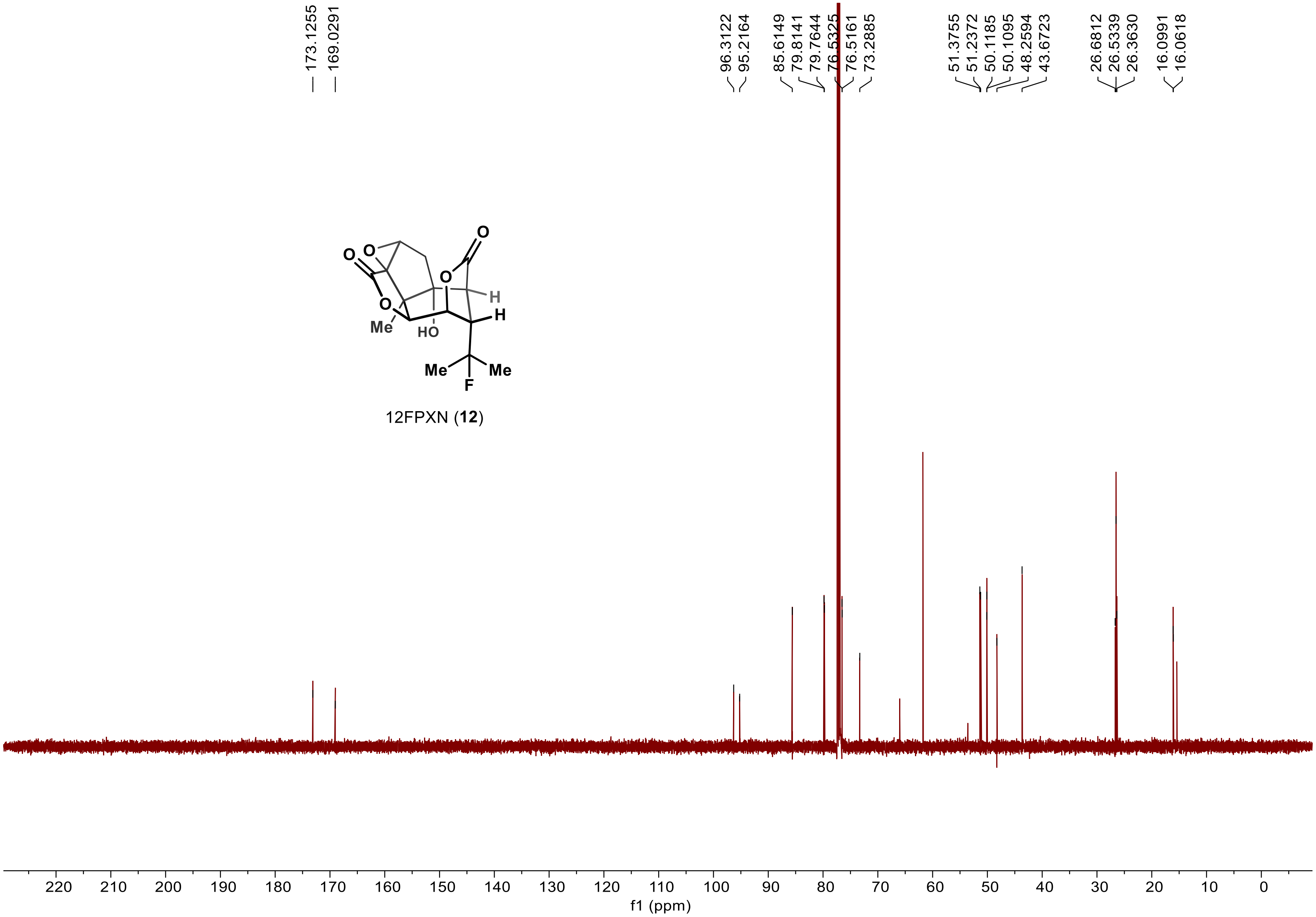

^{13}C NMR, $CDCl_3$, 150 MHz

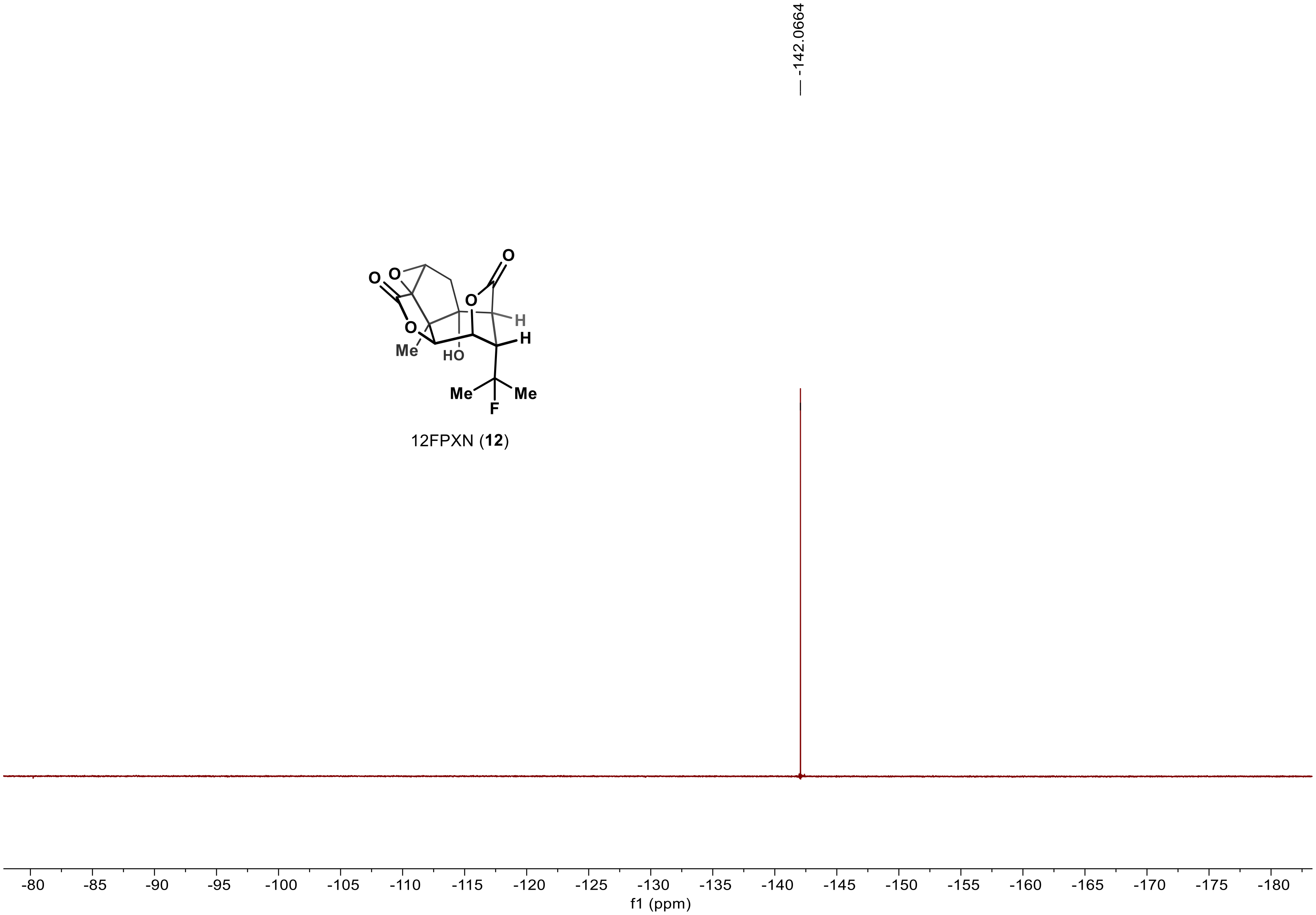

12FPXN (**12**)

^{19}F NMR, $CDCl_3$, 375 MHz

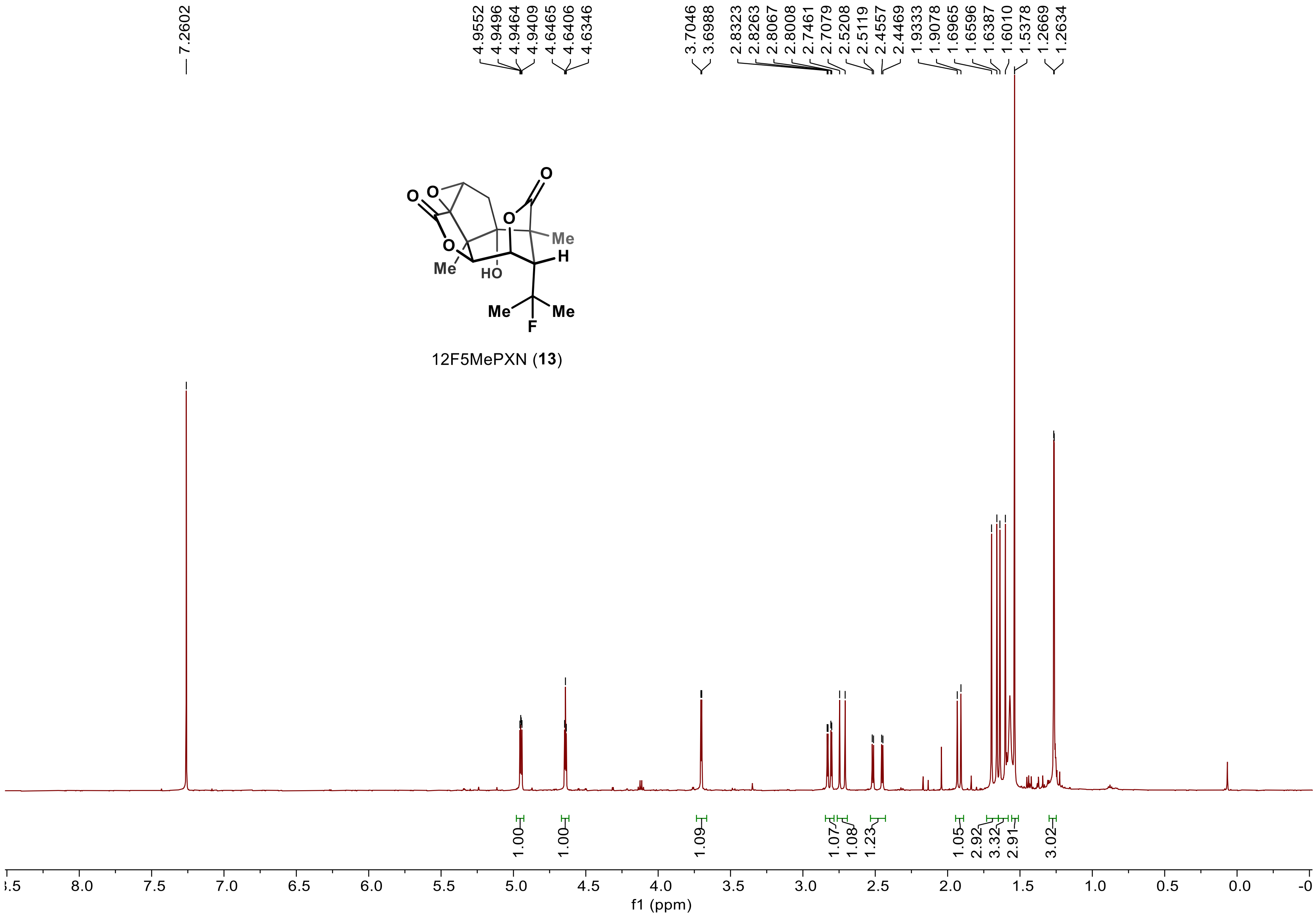

^{1}H NMR, $CDCl_3$, 600 MHz

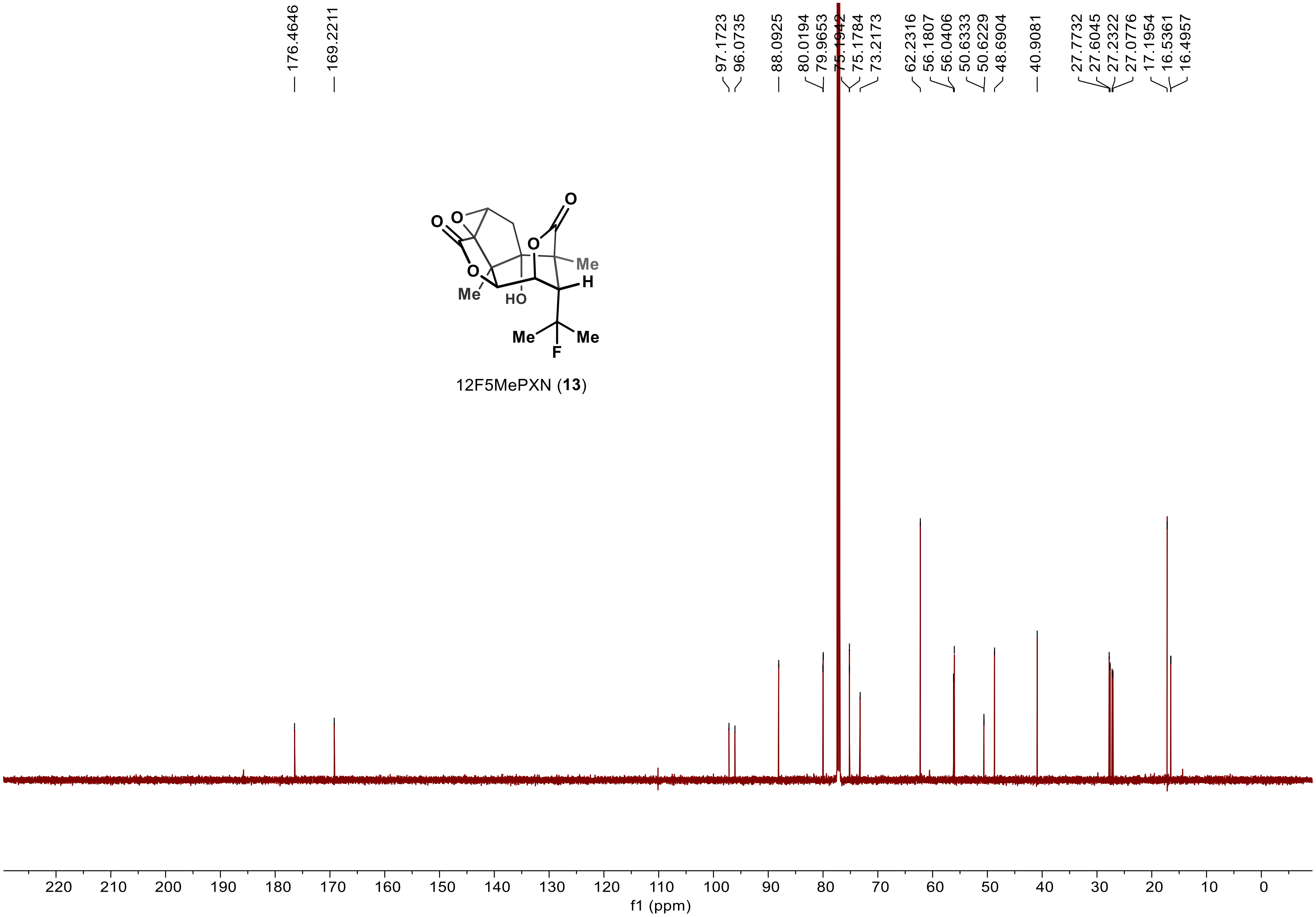

^{13}C NMR, $CDCl_3$, 150 MHz

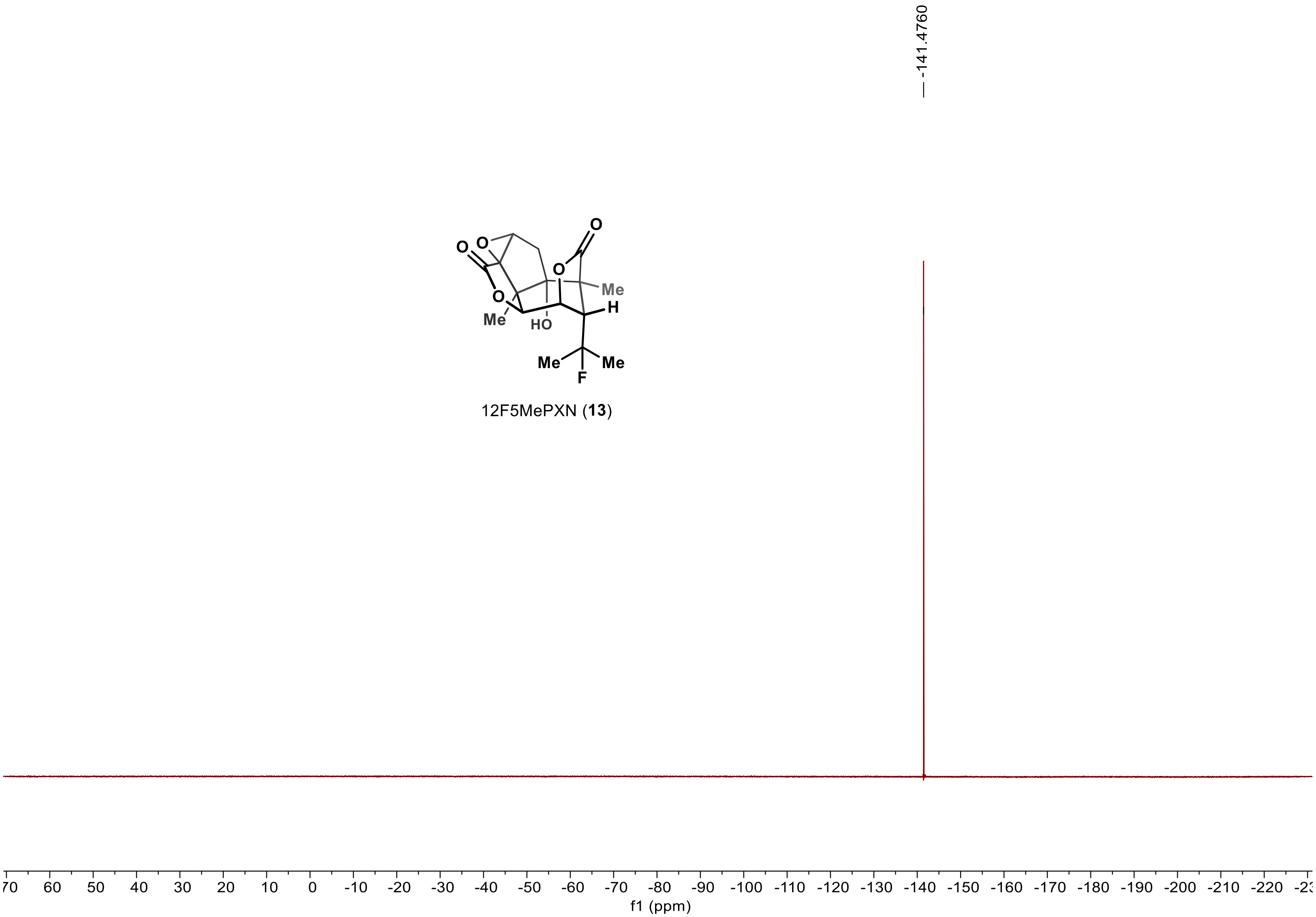

12F5MePXN (**13**)

^{19}F NMR, $CDCl_3$, 375 MHz

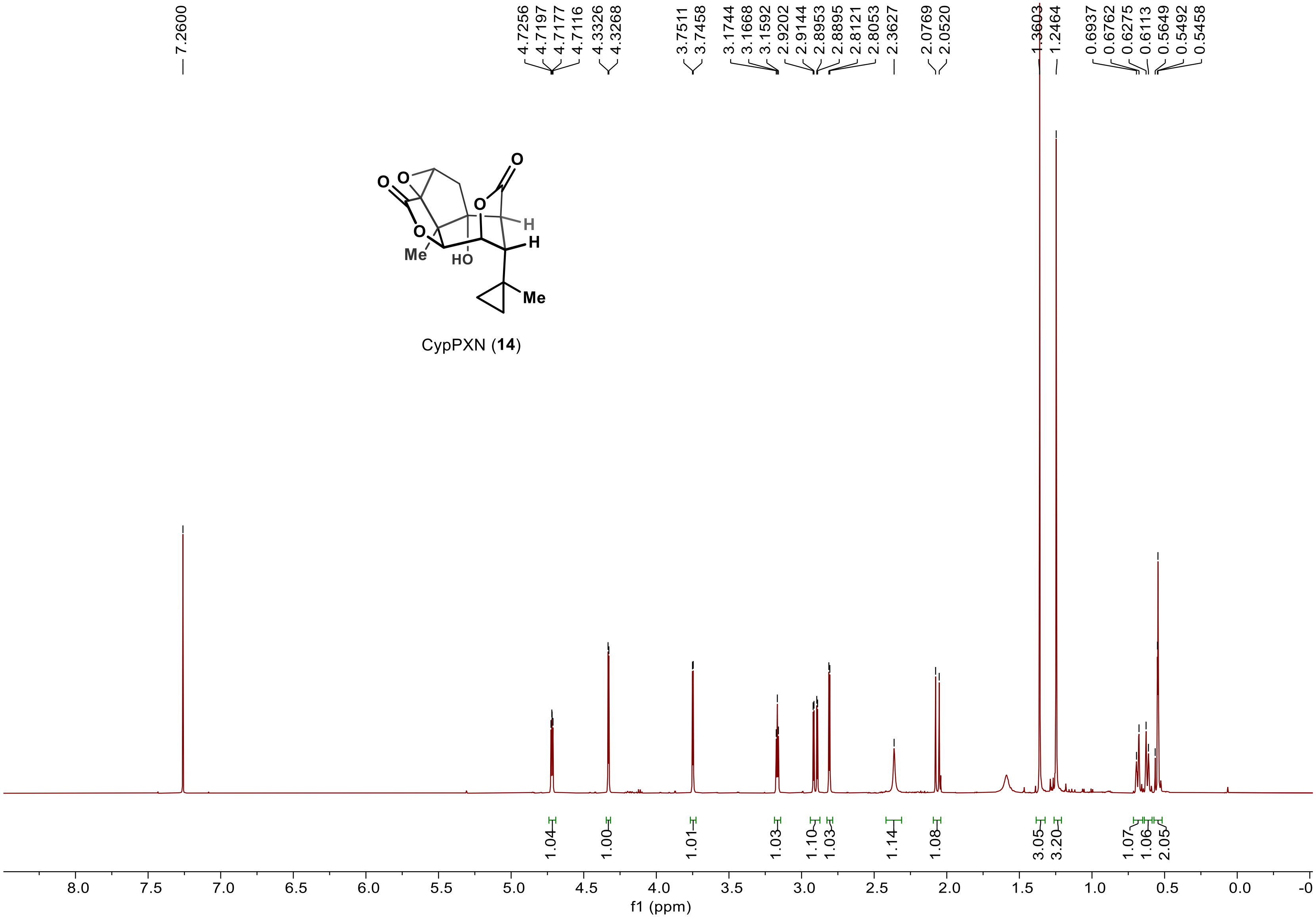

^{1}H NMR, $CDCl_3$, 600 MHz

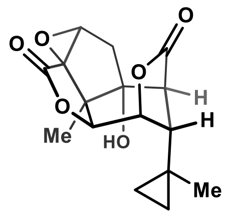

CypPXN (**14**)

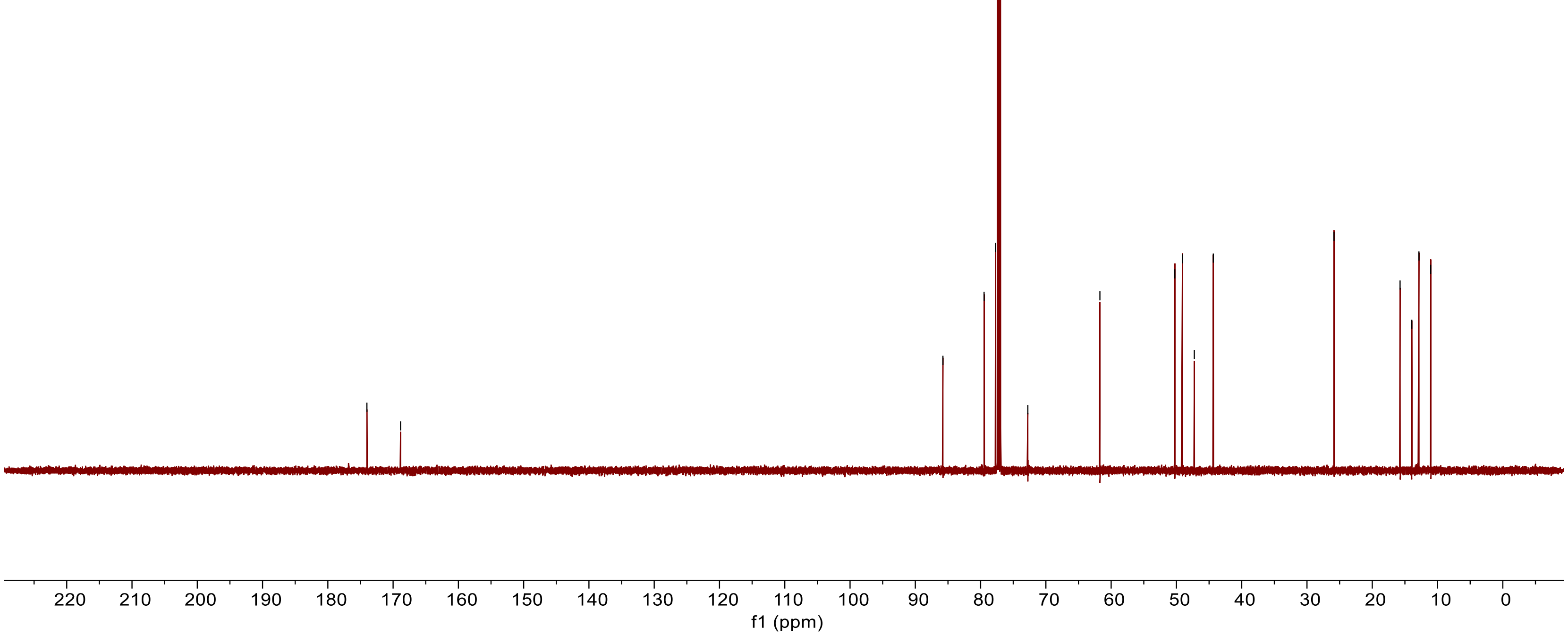

^{13}C NMR, $CDCl_3$, 150 MHz

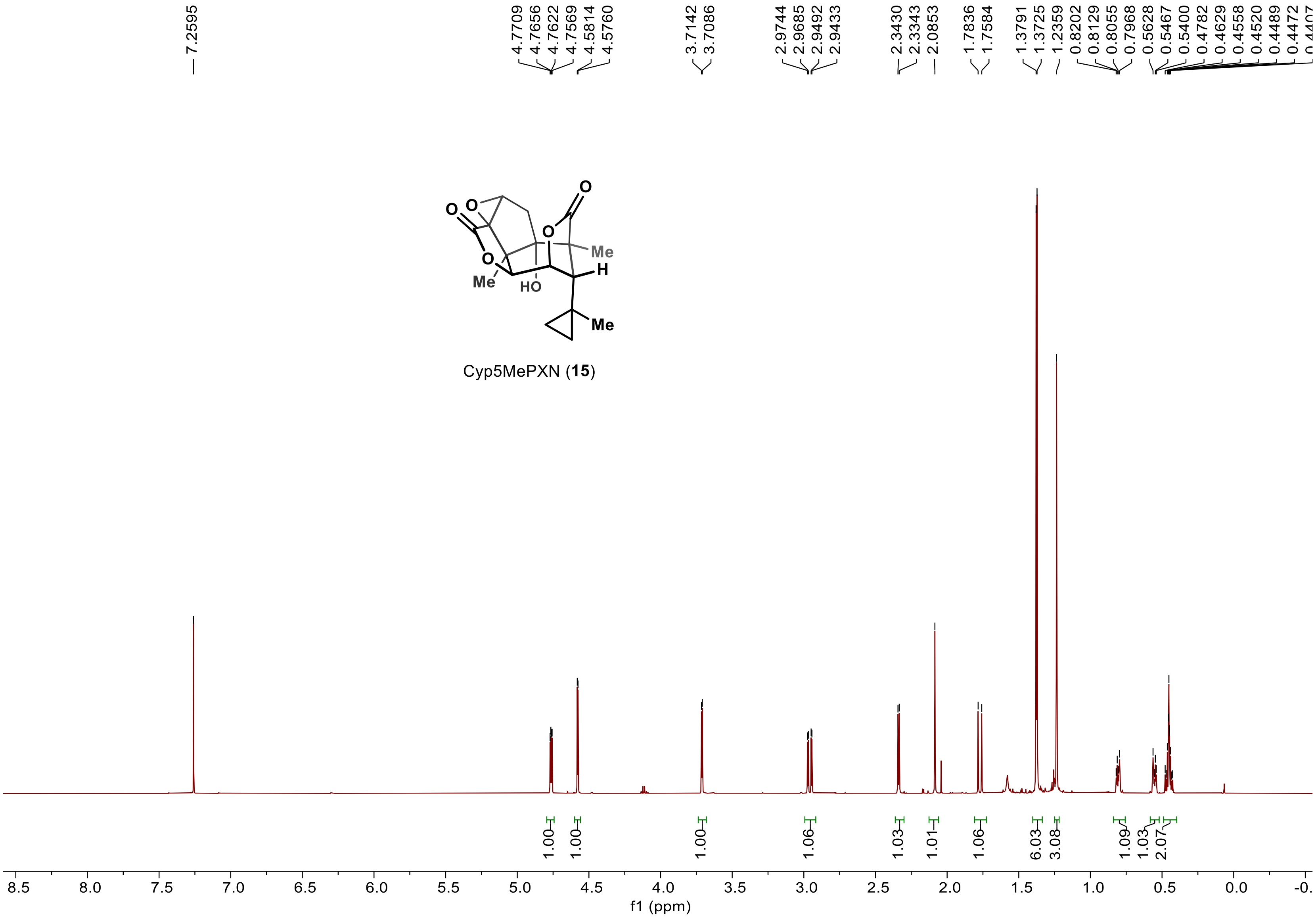

^{1}H NMR, $CDCl_3$, 600 MHz

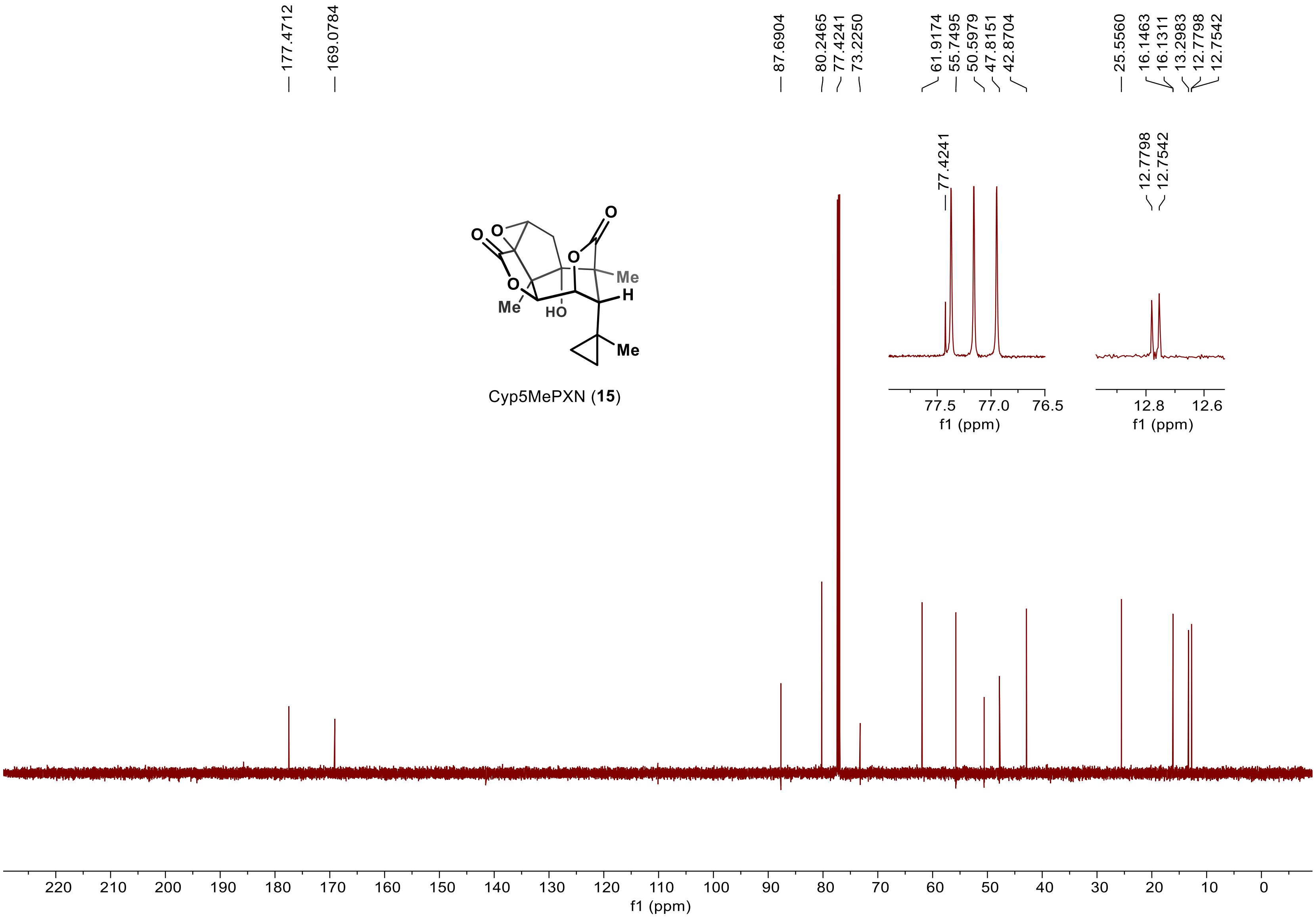

^{13}C NMR, $CDCl_3$, 150 MHz

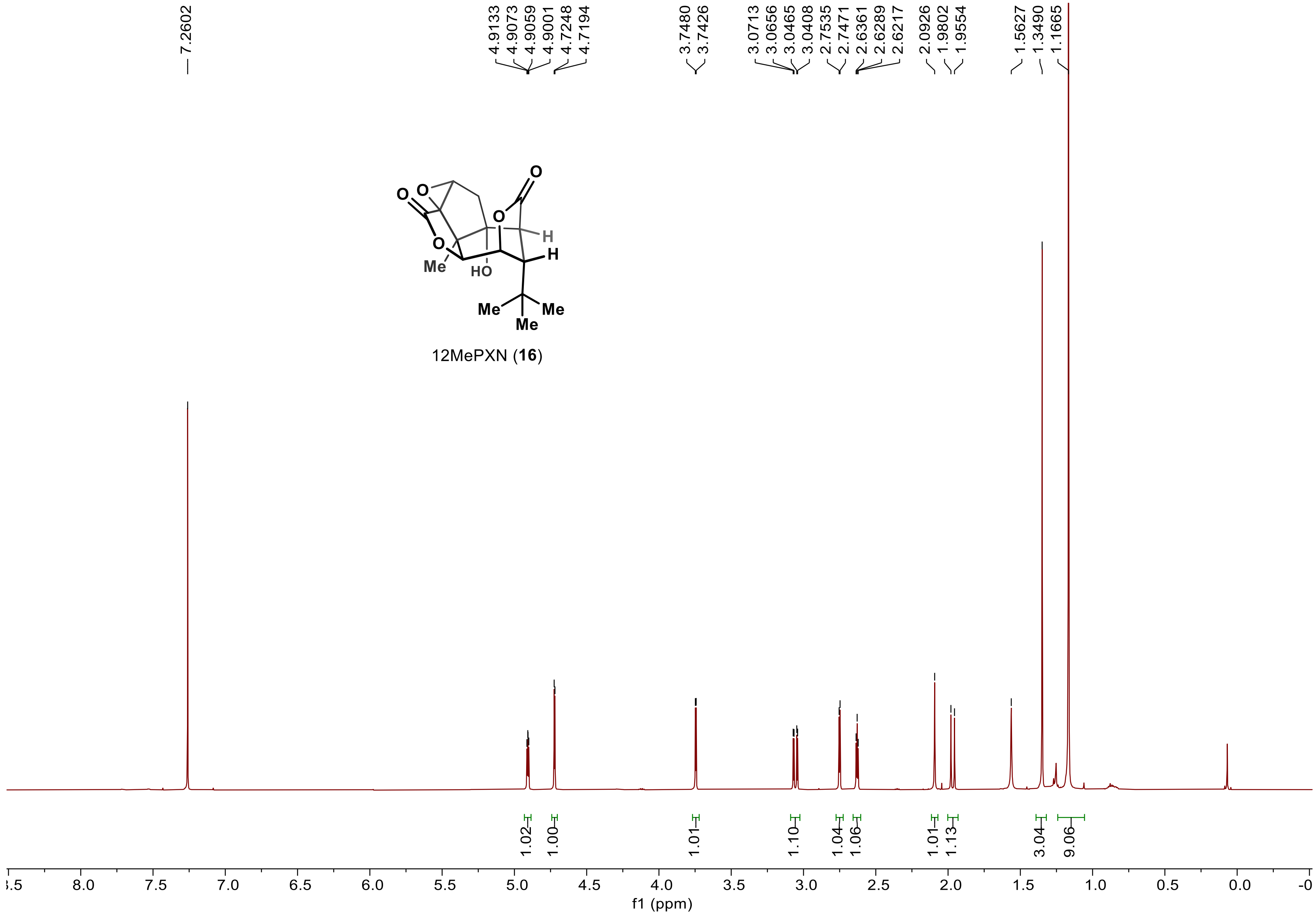

^{1}H NMR, $CDCl_3$, 600 MHz

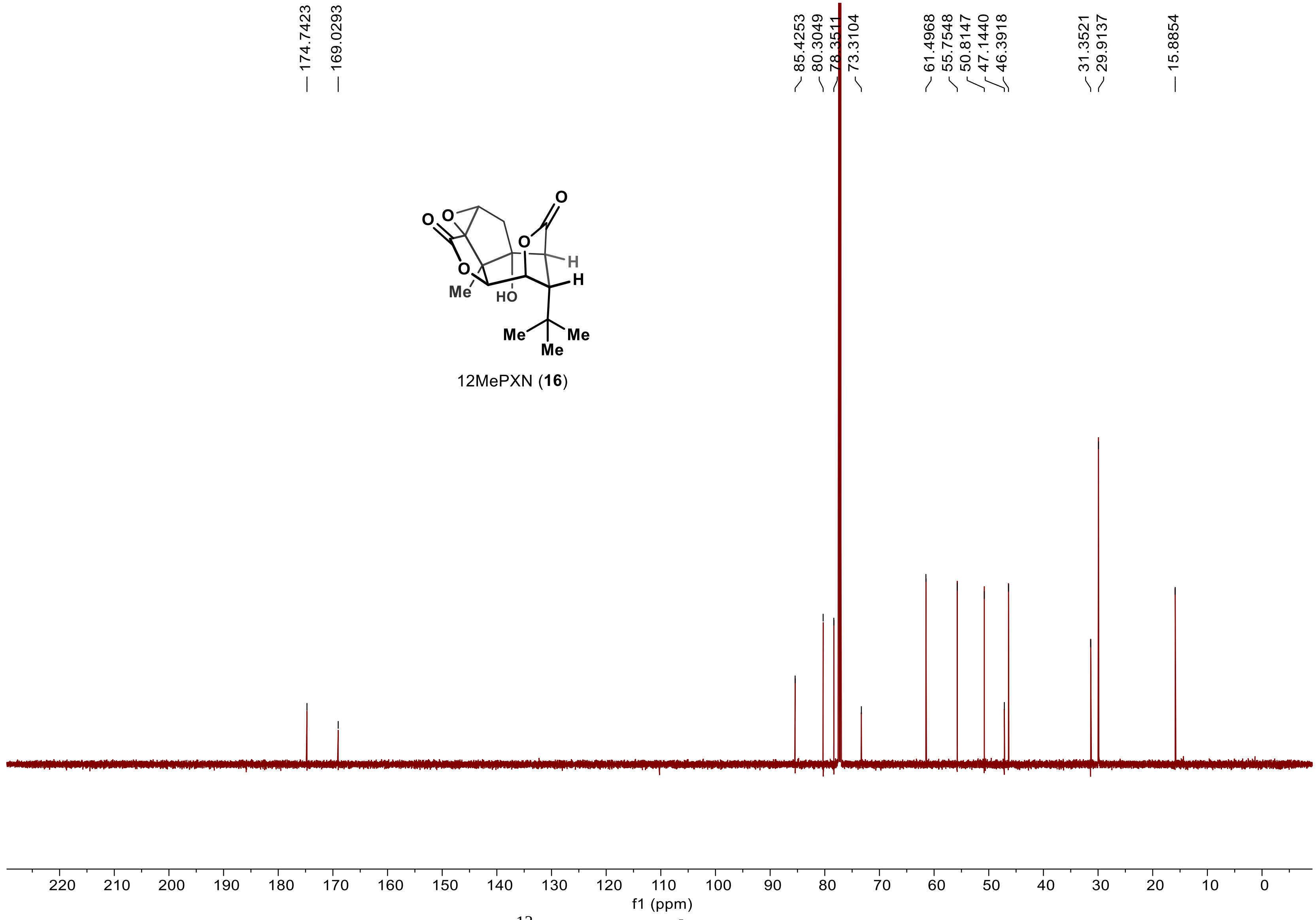

^{13}C NMR, $CDCl_3$, 150 MHz

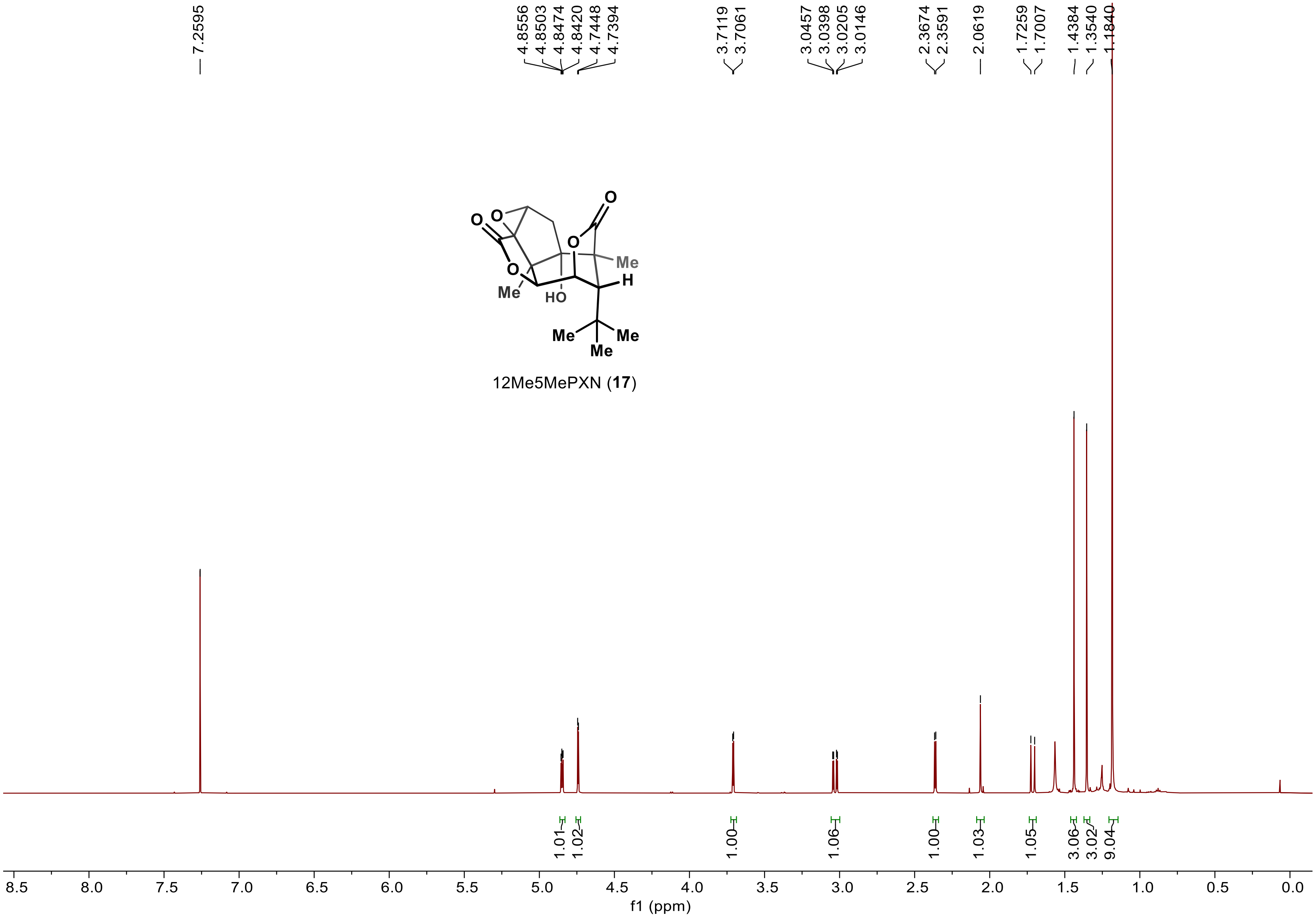

^{1}H NMR, $CDCl_3$, 600 MHz

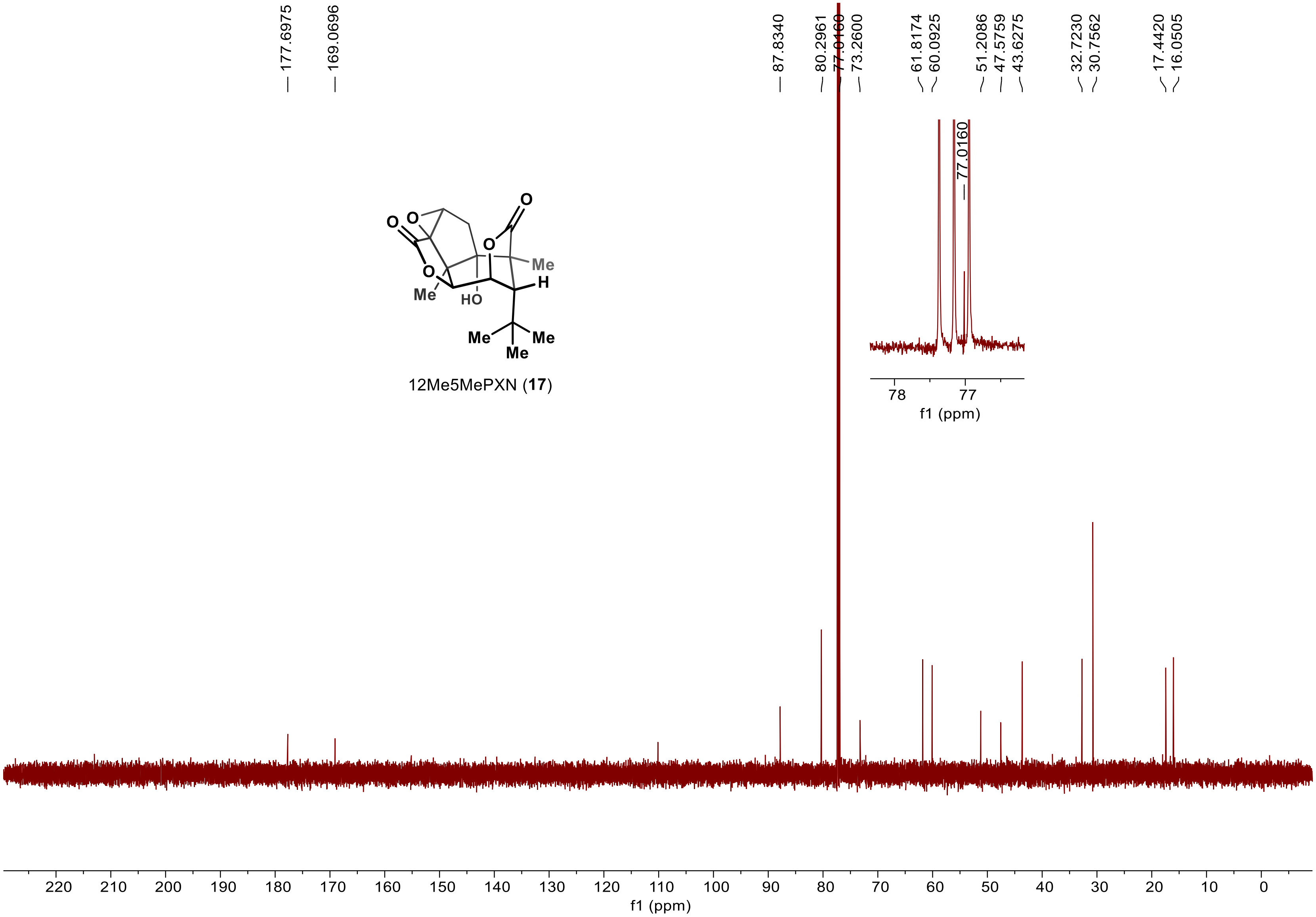

^{13}C NMR, $CDCl_3$, 150 MHz

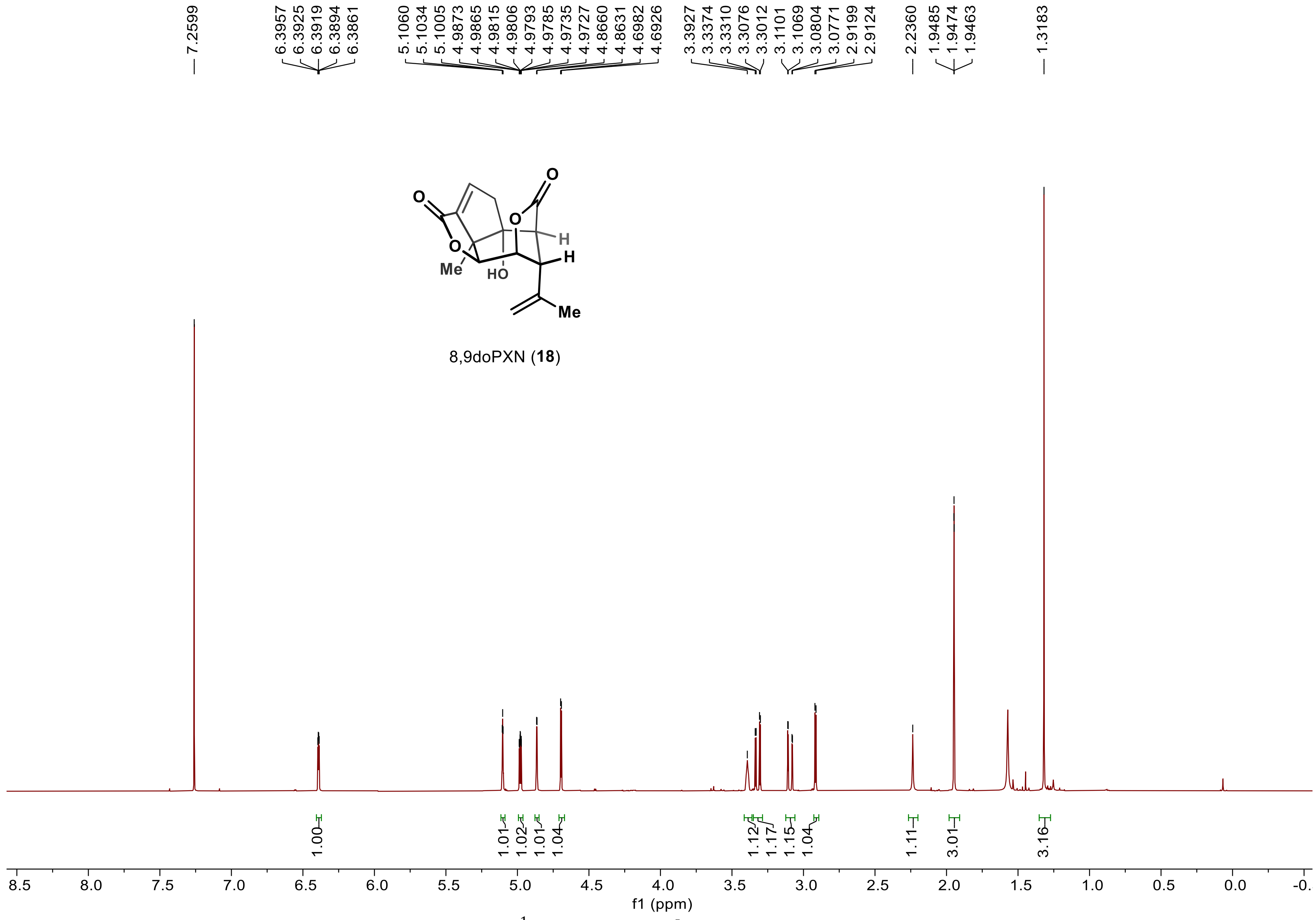

8,9doPXN (**18**)

^{1}H NMR, $CDCl_3$, 600 MHz

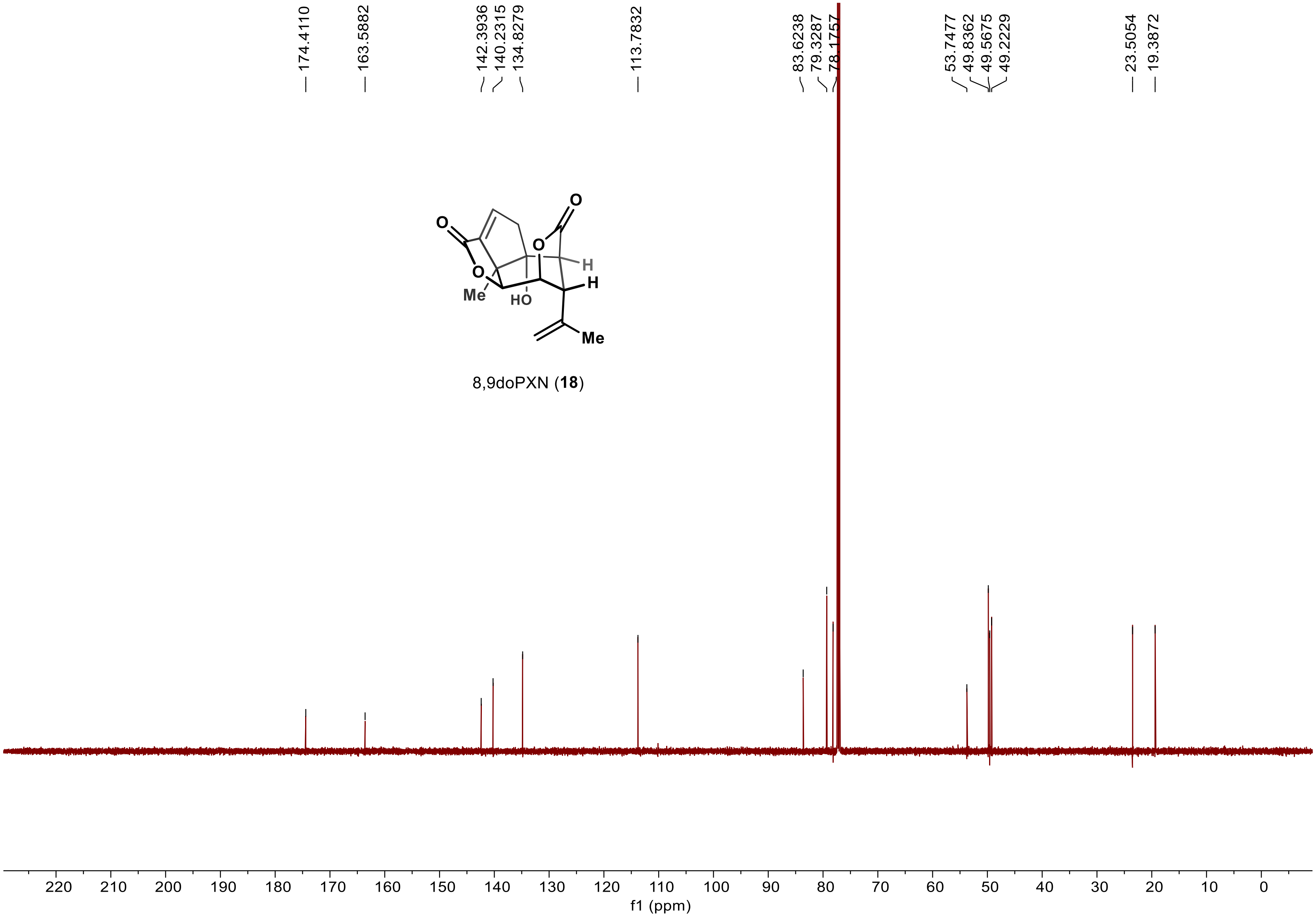

^{13}C NMR, $CDCl_3$, 150 MHz

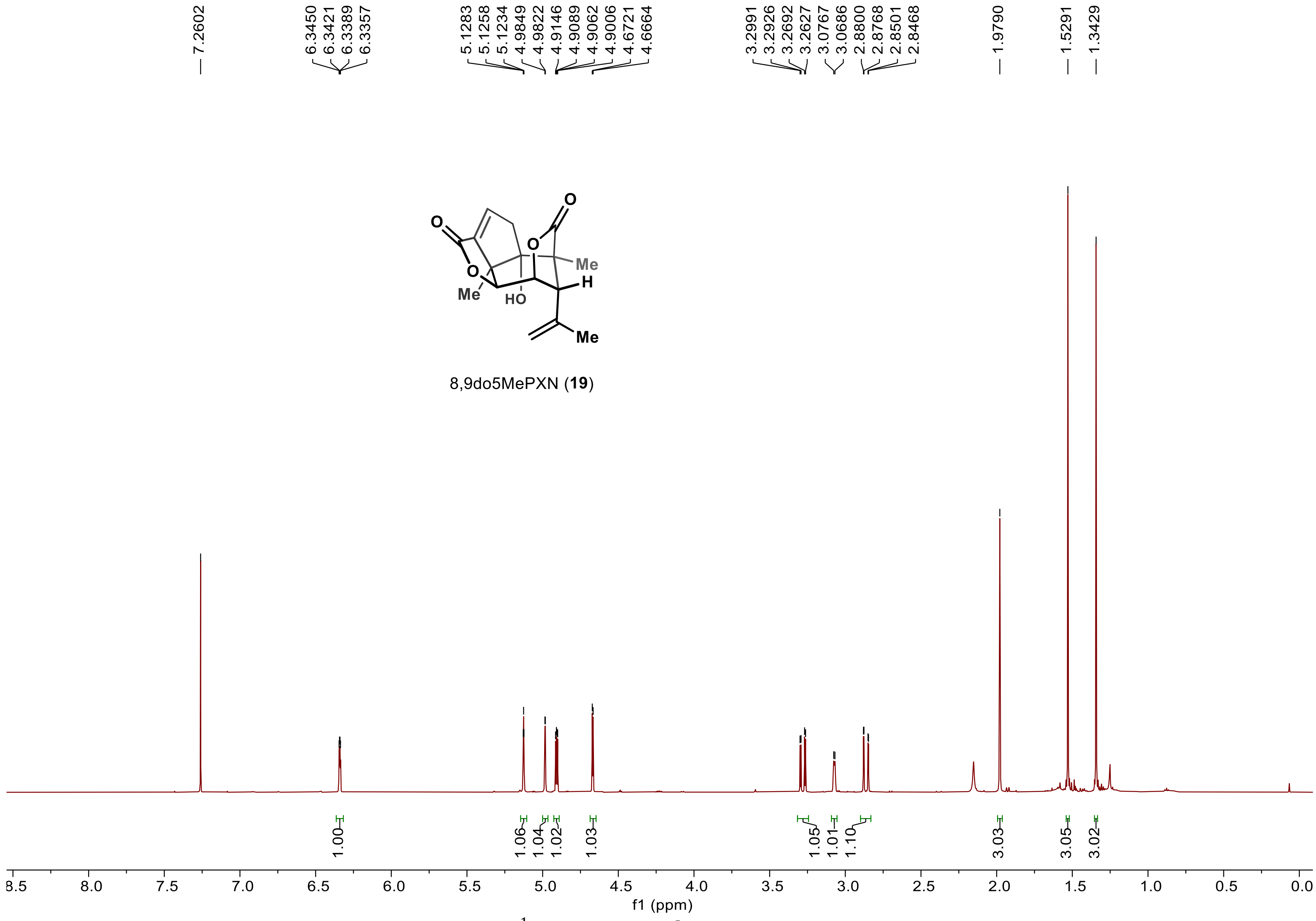

^{1}H NMR, $CDCl_3$, 600 MHz

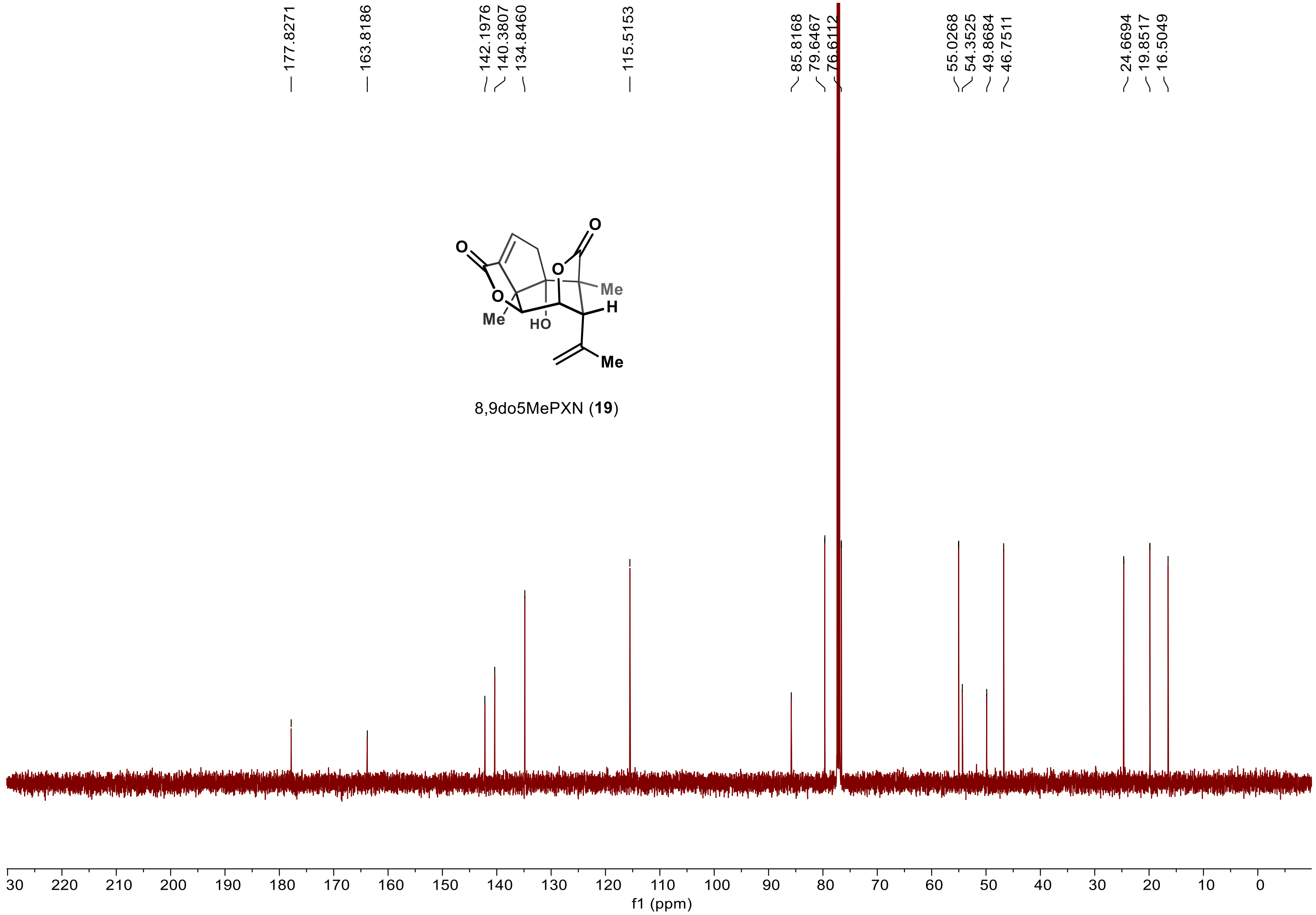

^{13}C NMR, $CDCl_3$, 150 MHz

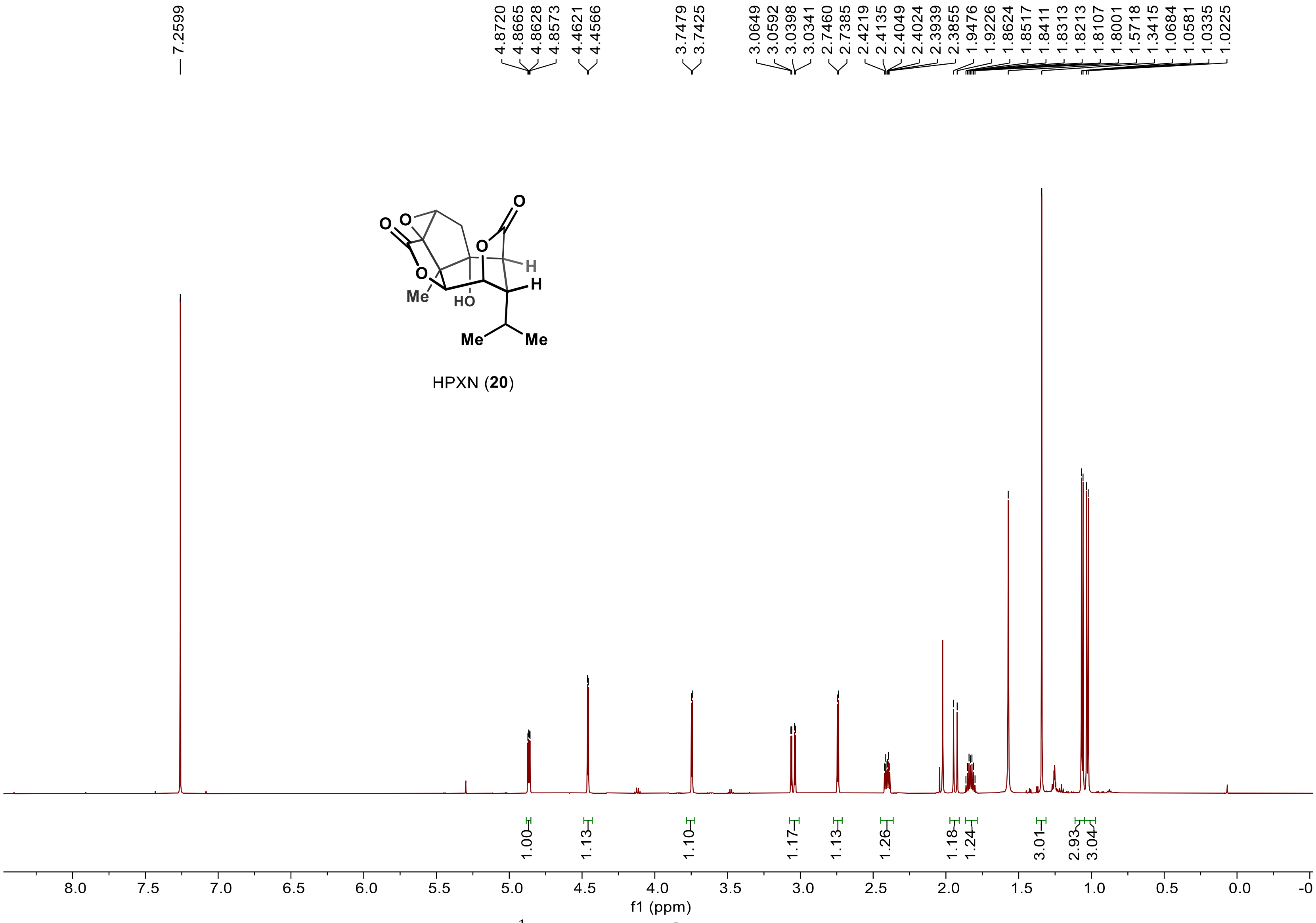

^{1}H NMR, $CDCl_3$, 600 MHz

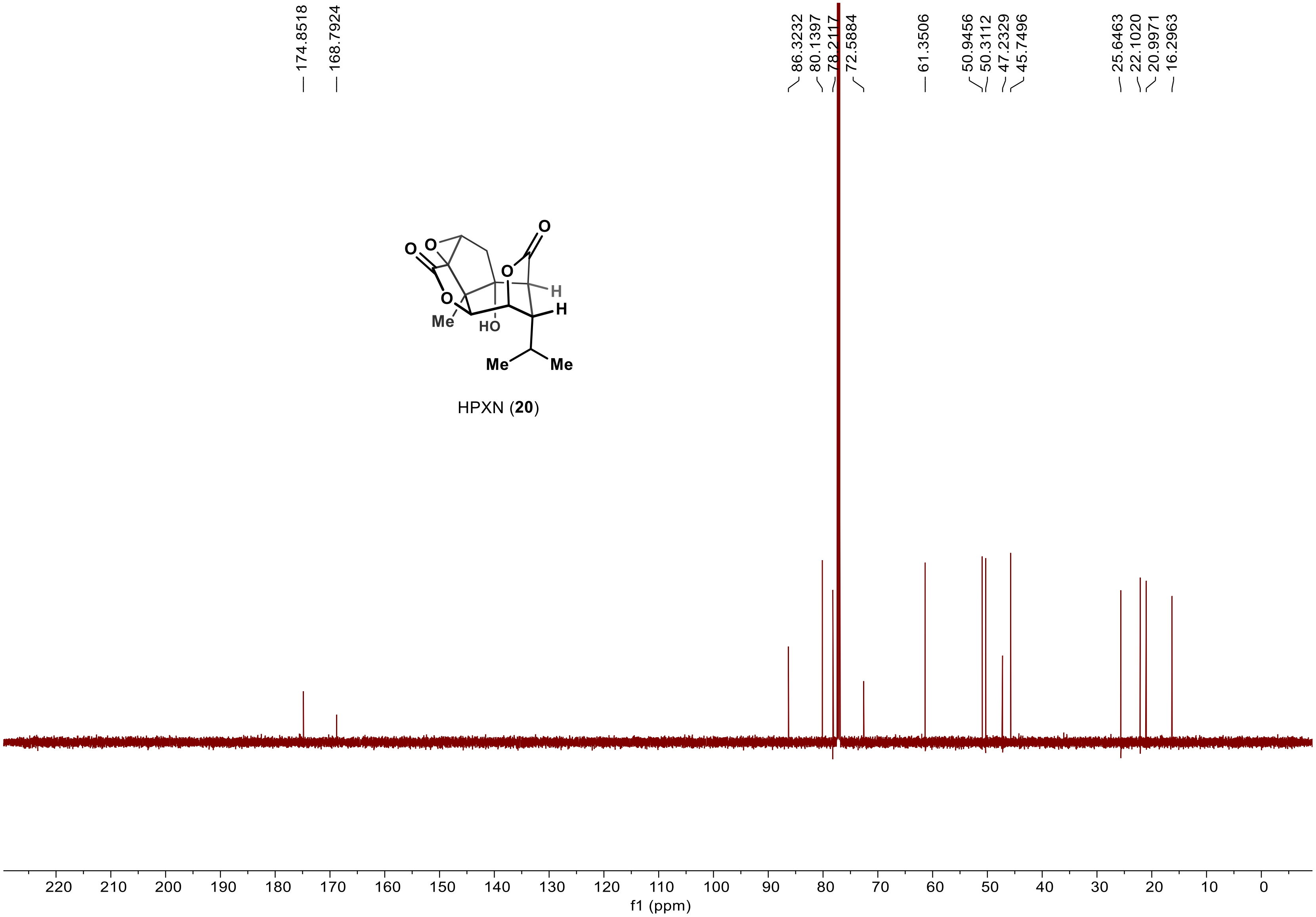

^{13}C NMR, $CDCl_3$, 150 MHz

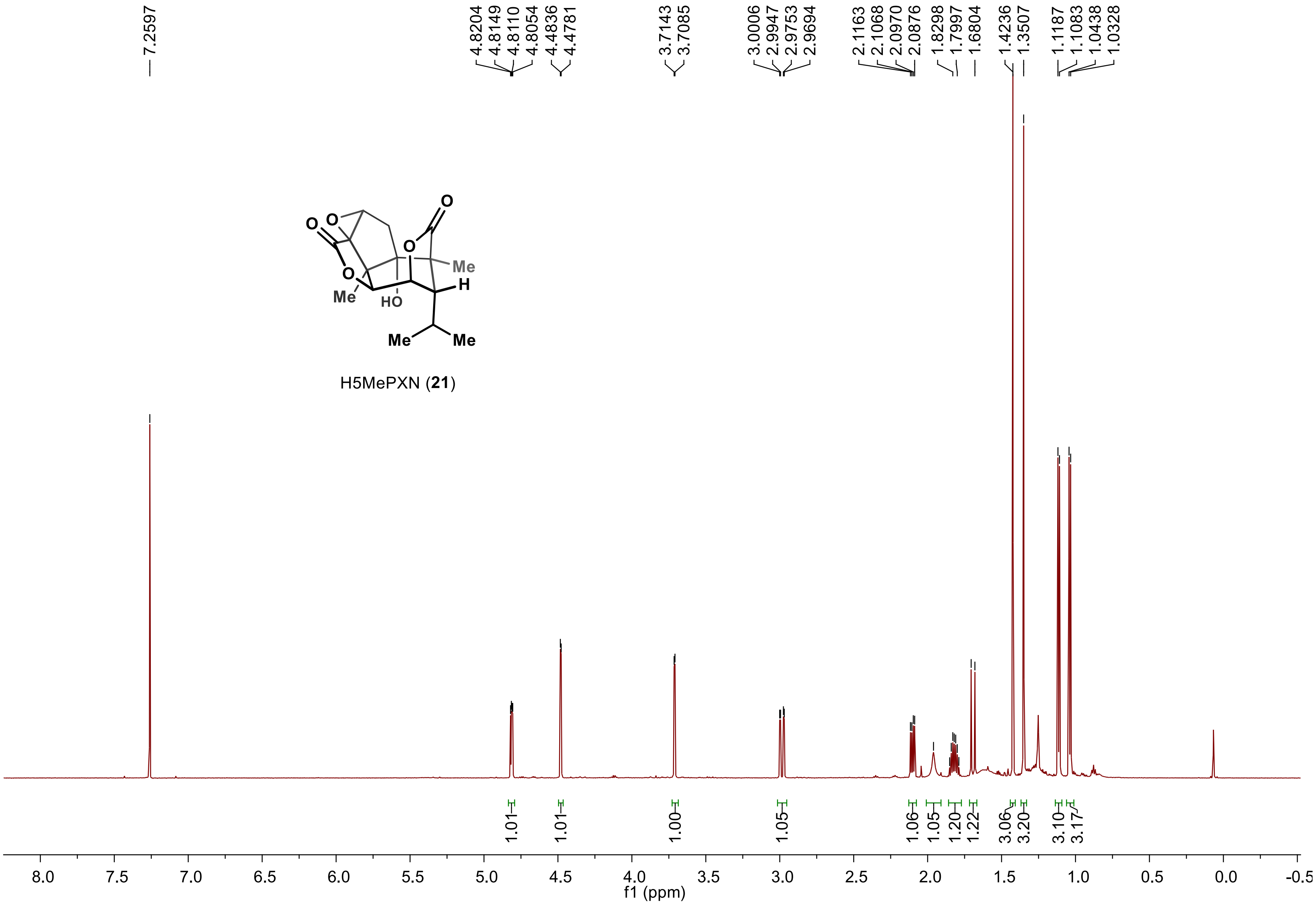

^{1}H NMR, $CDCl_3$, 600 MHz

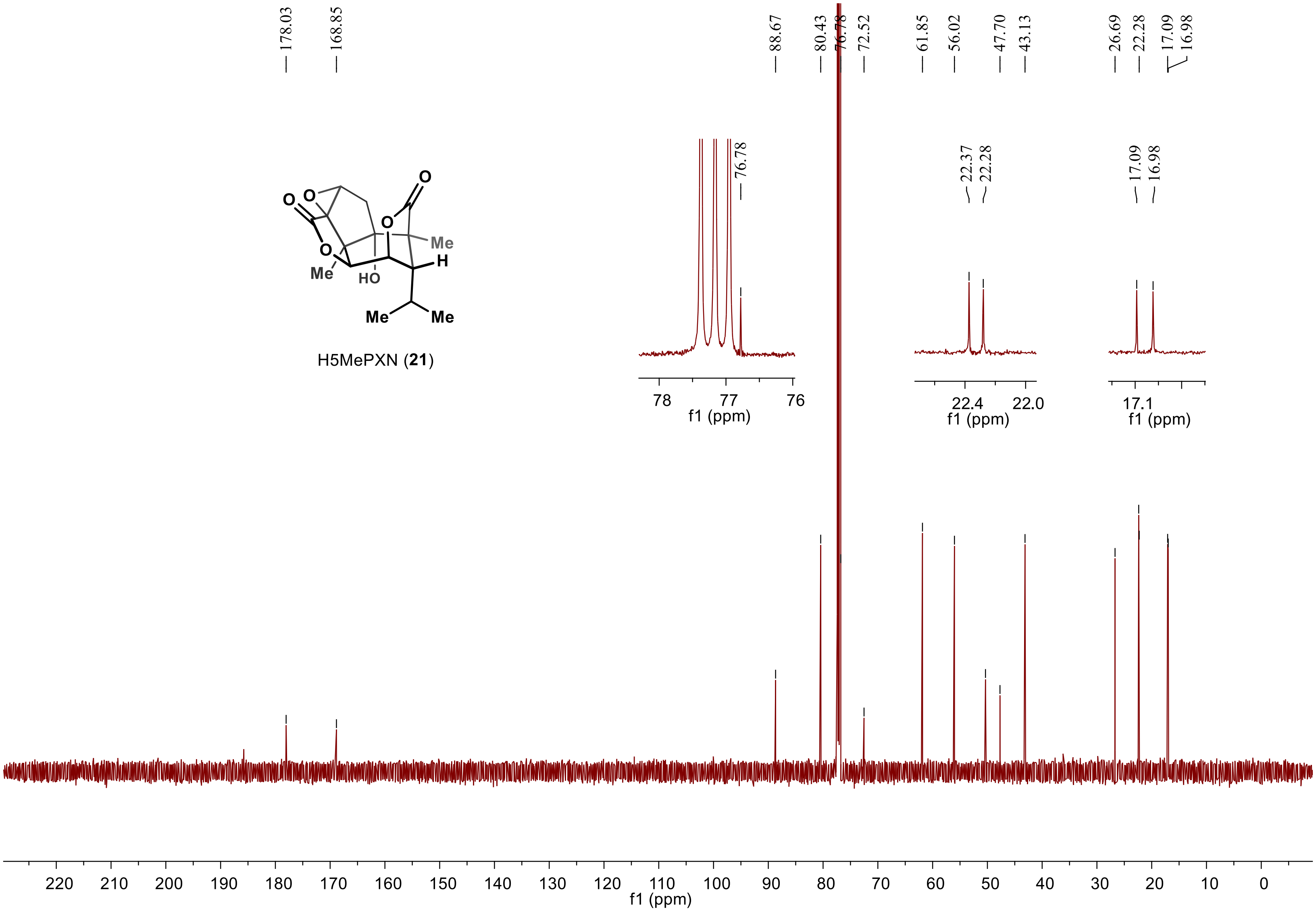

^{13}C NMR, $CDCl_3$, 150 MHz

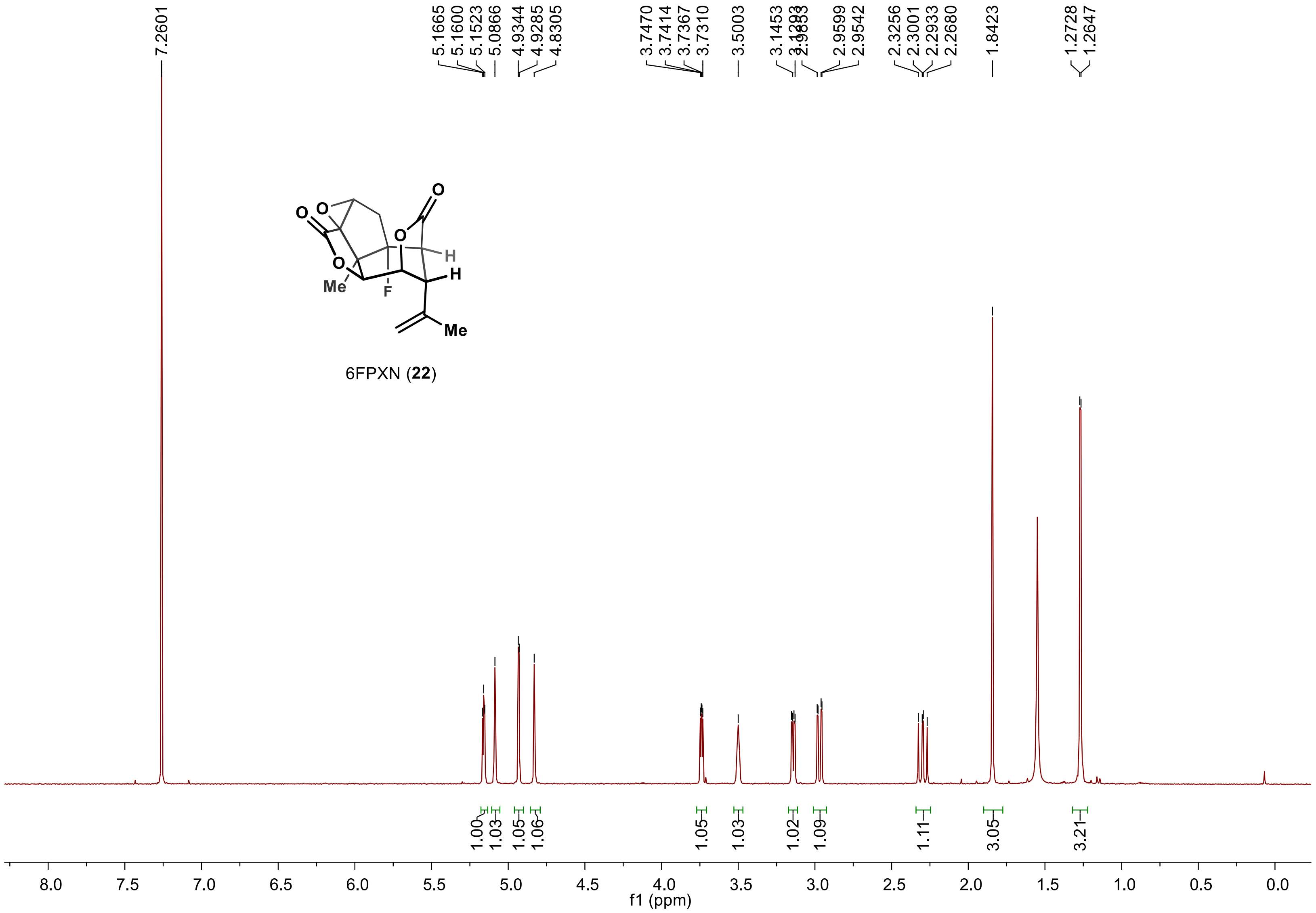

^{1}H NMR, $CDCl_3$, 600 MHz

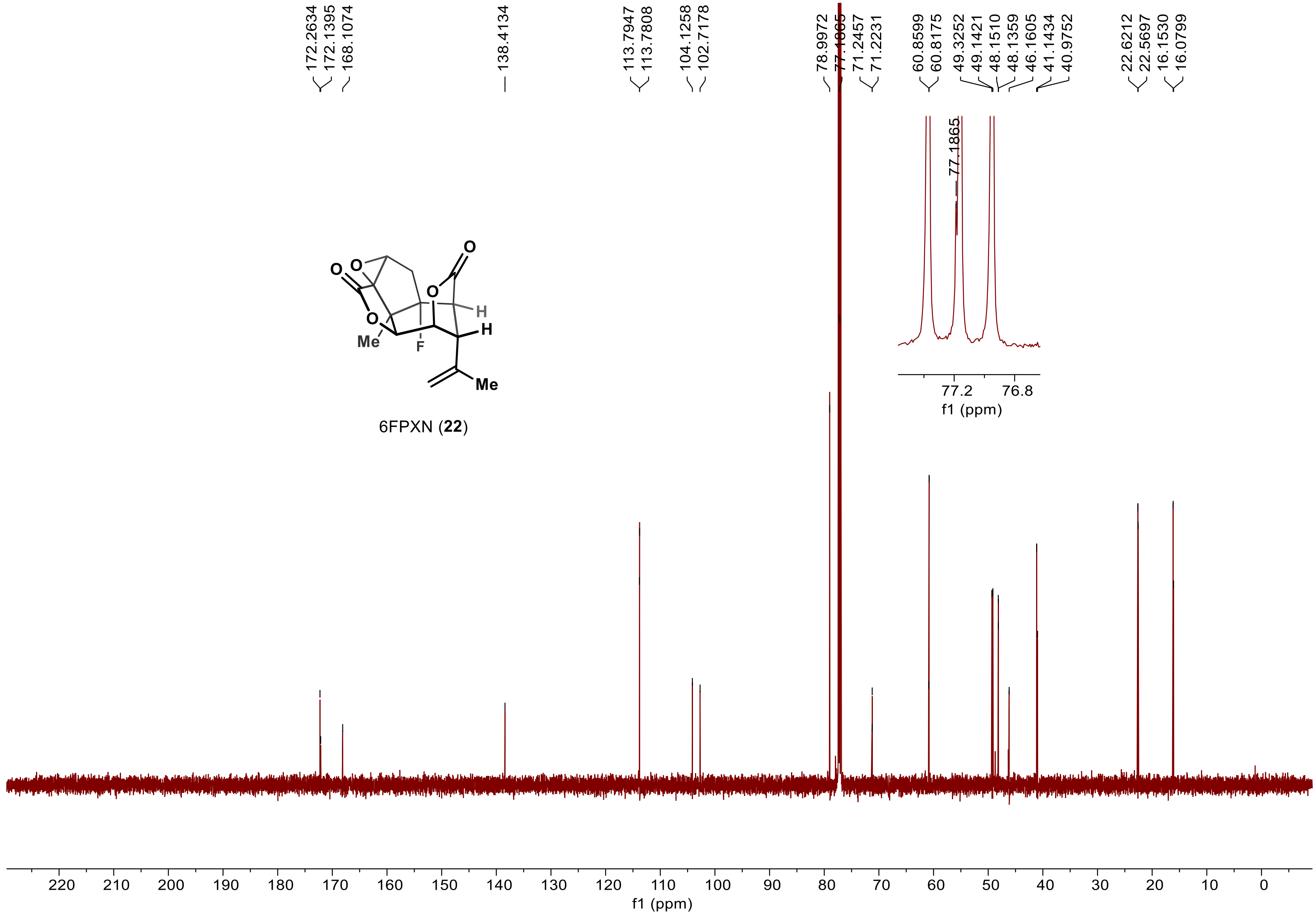

^{13}C NMR, $CDCl_3$, 150 MHz

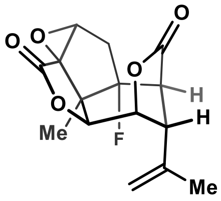

6FPXN (**22**)

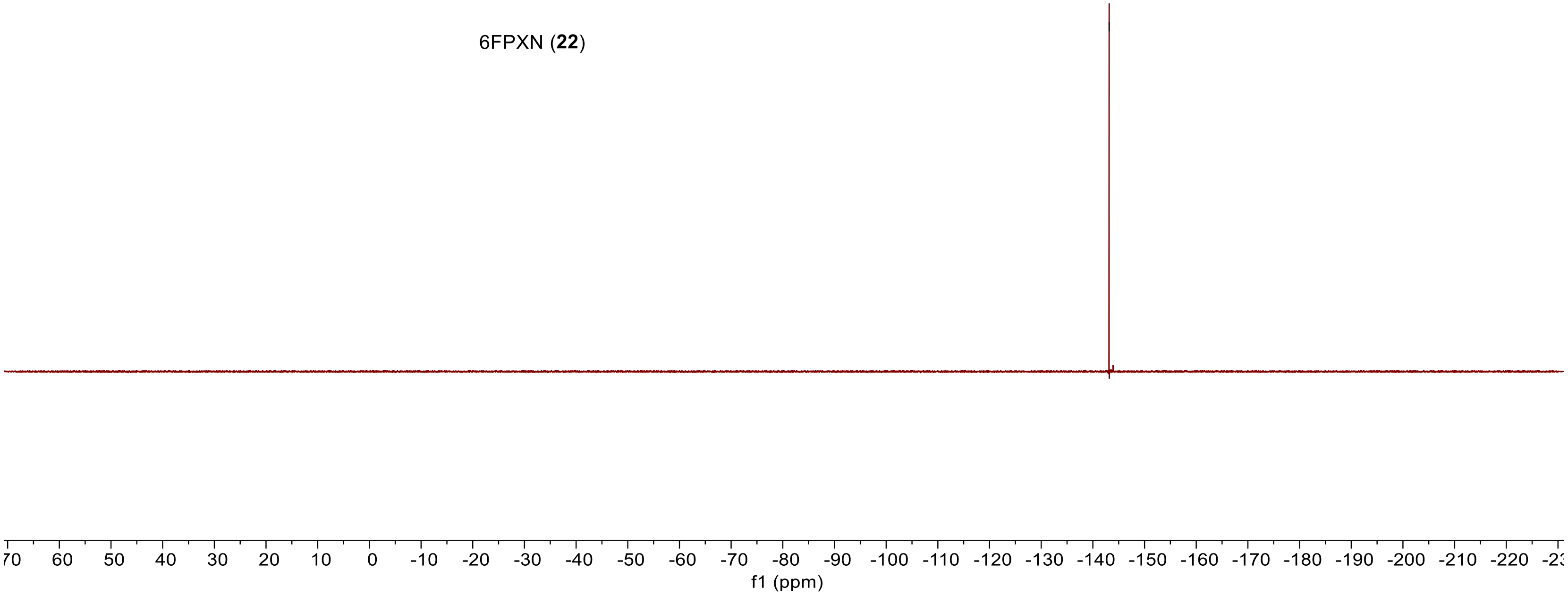

^{19}F NMR, $CDCl_3$, 375 MHz

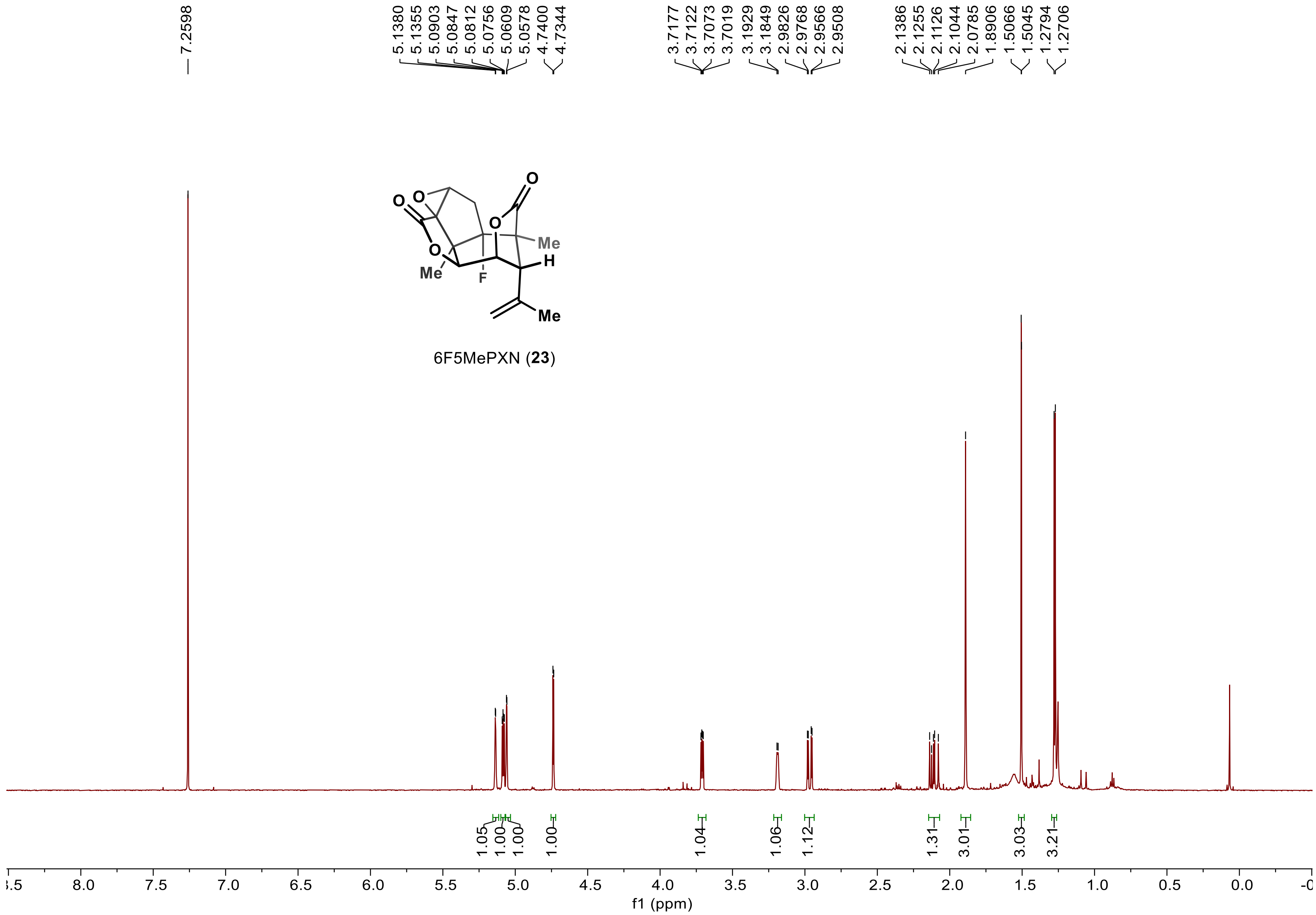

^{1}H NMR, $CDCl_3$, 600 MHz

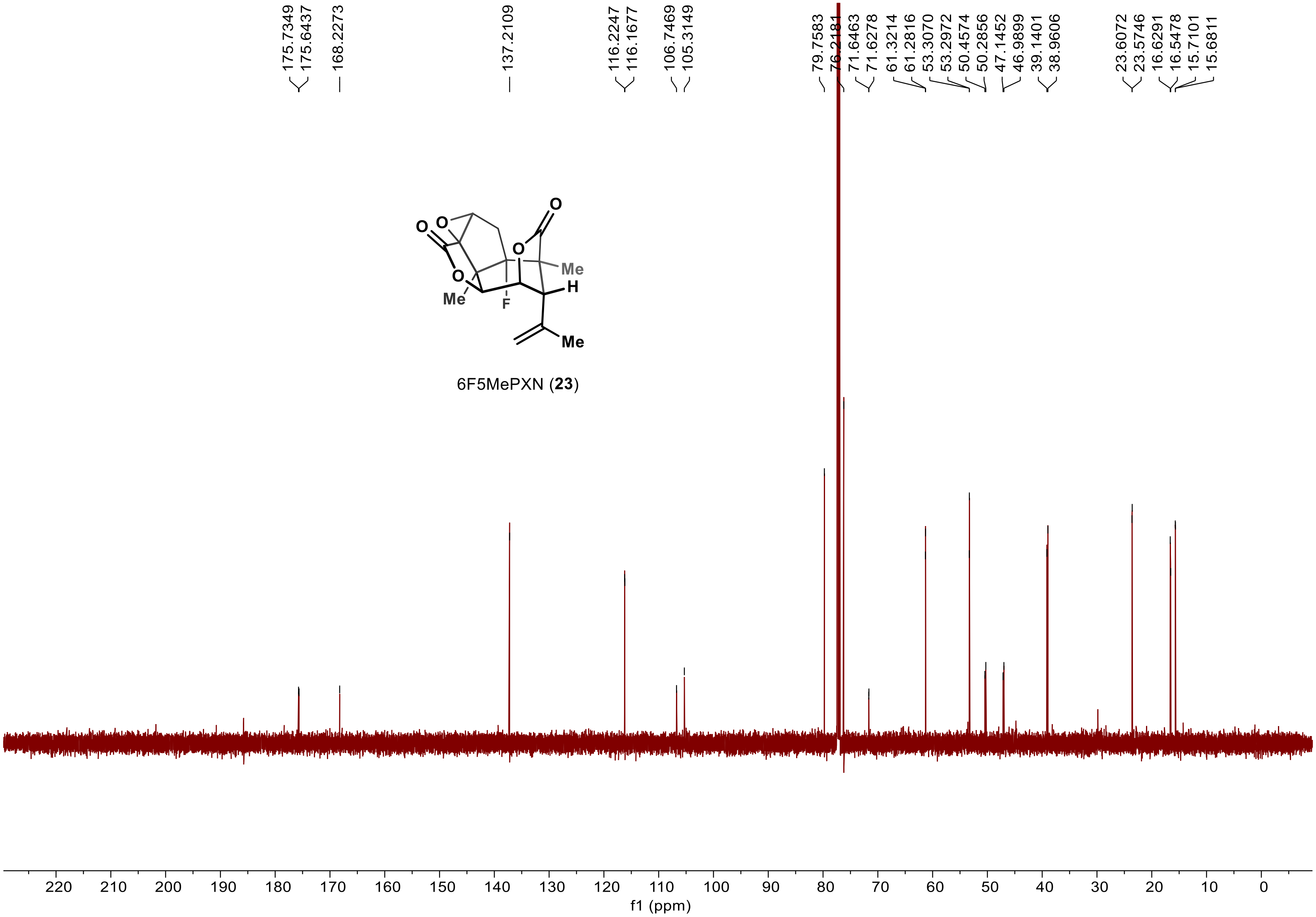

6F5MePXN (**23**)

^{13}C NMR, $CDCl_3$, 150 MHz

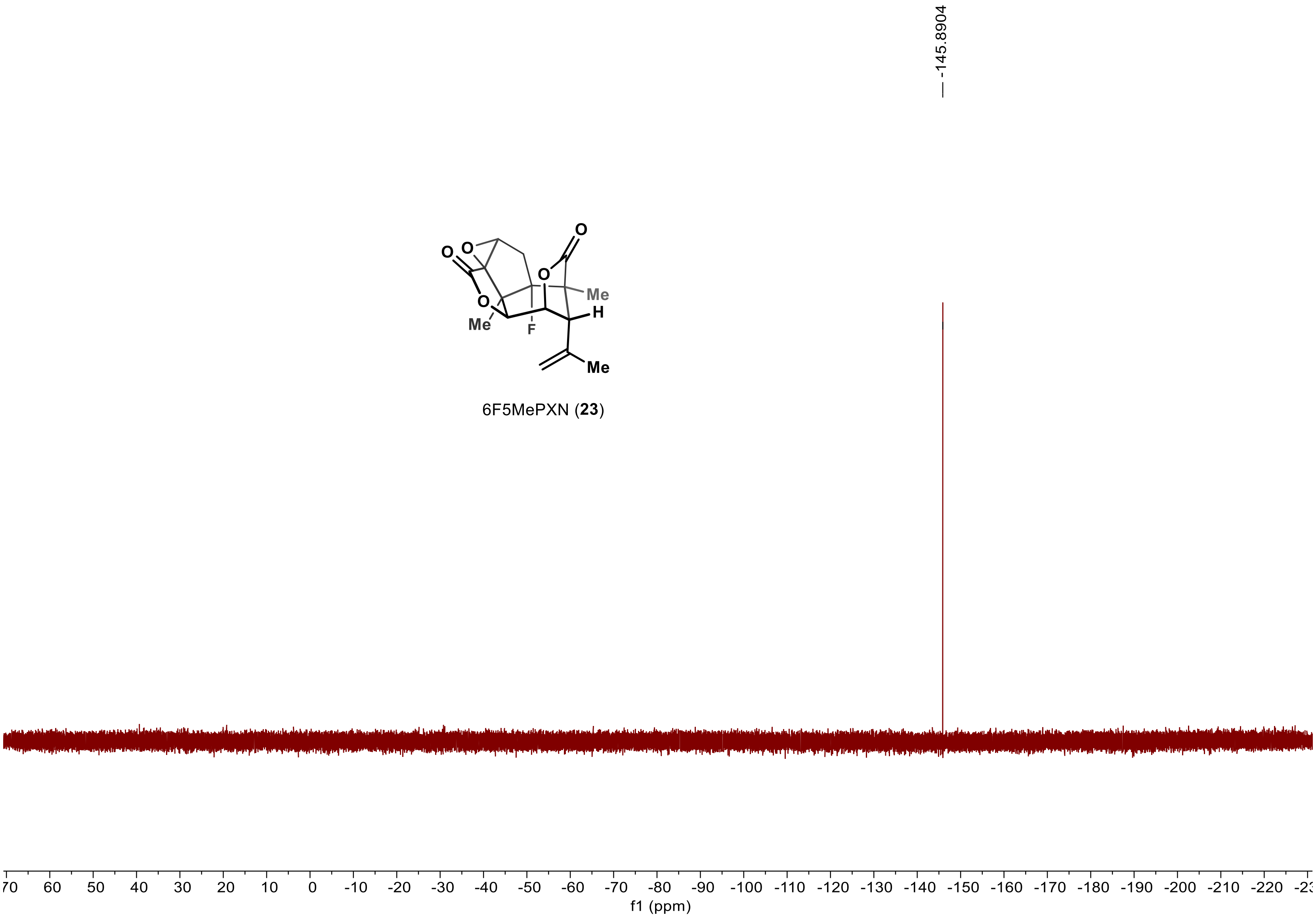

^{19}F NMR, $CDCl_3$, 375 MHz

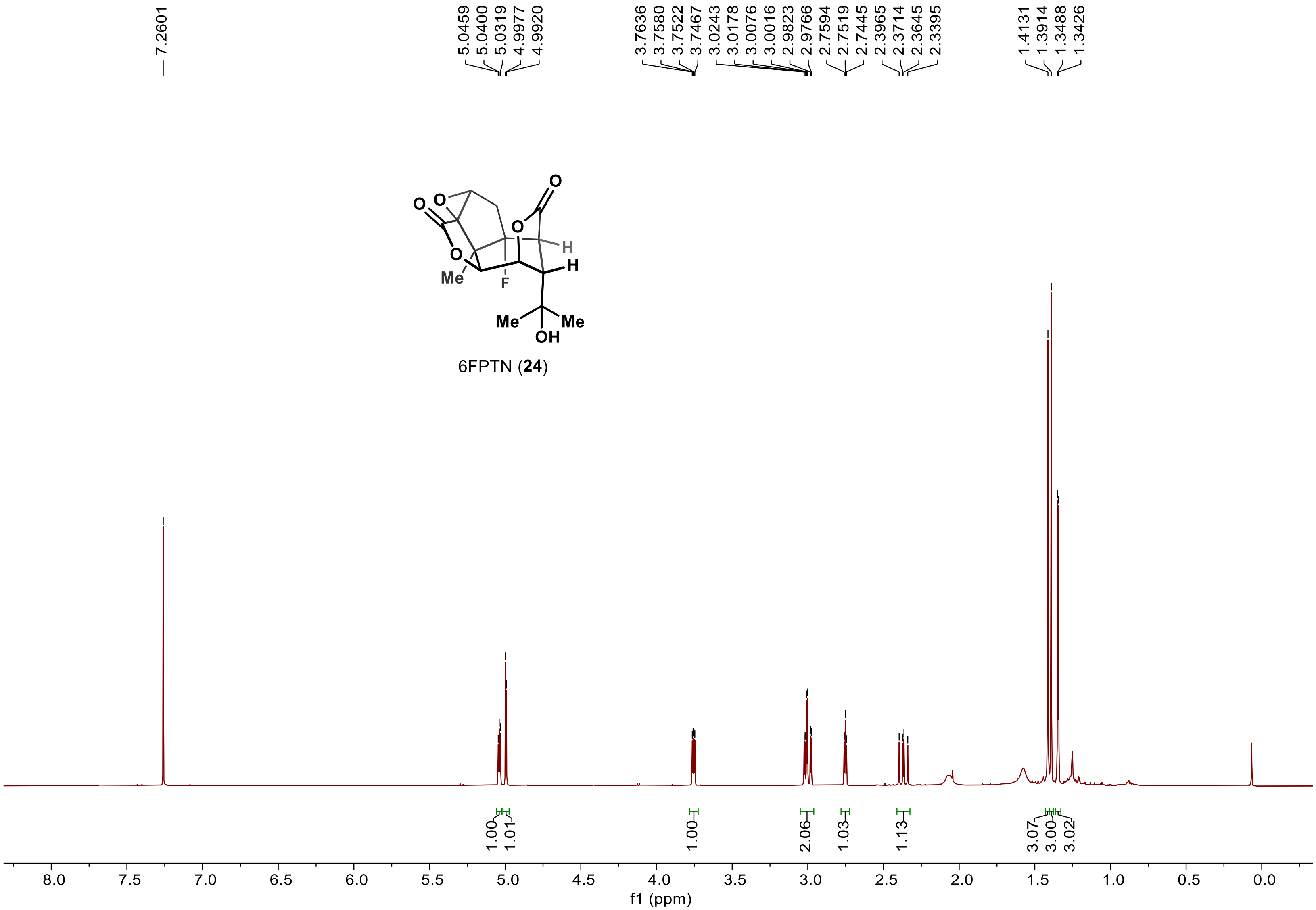

^{1}H NMR, $CDCl_3$, 600 MHz

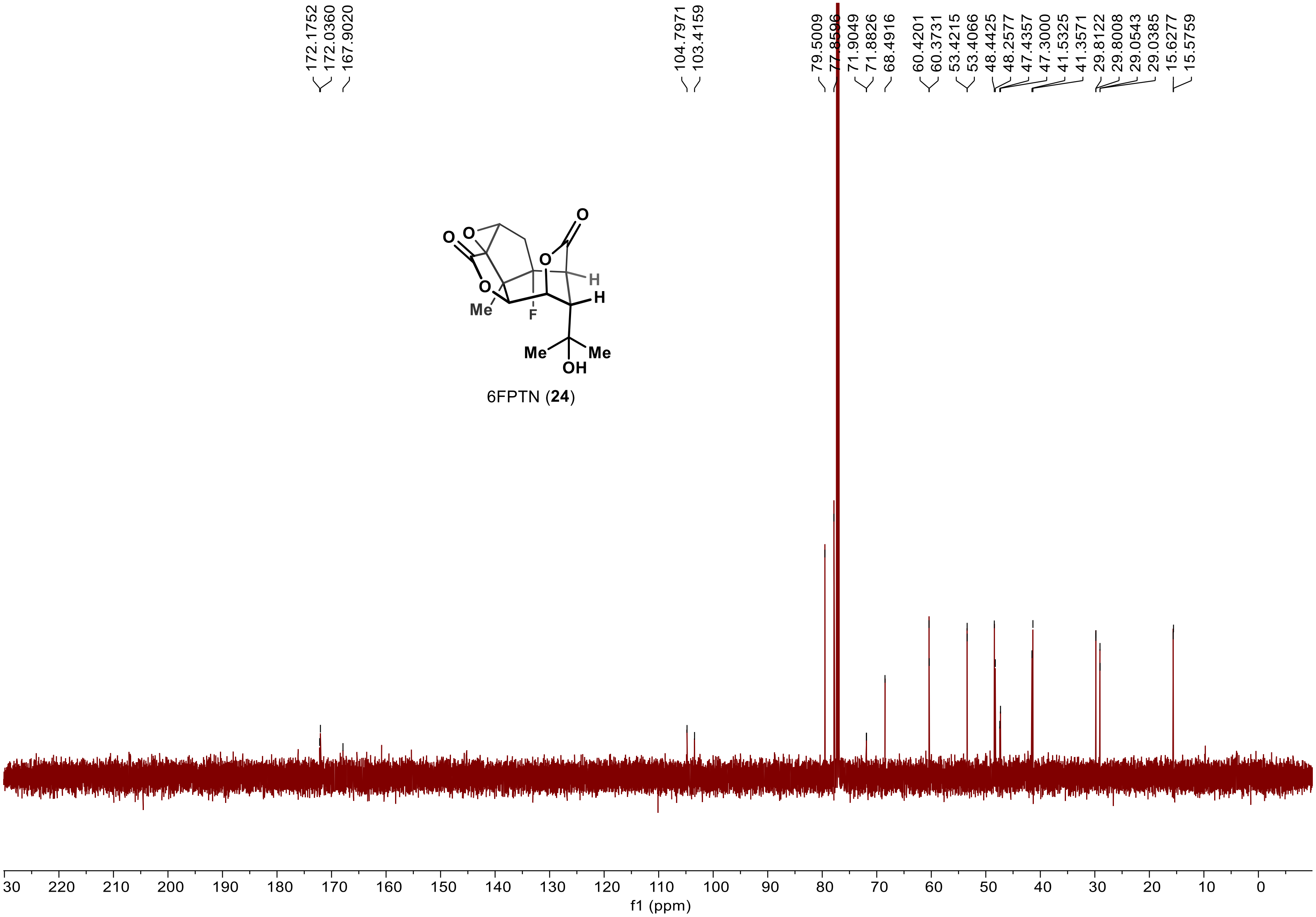

^{13}C NMR, $CDCl_3$, 150 MHz

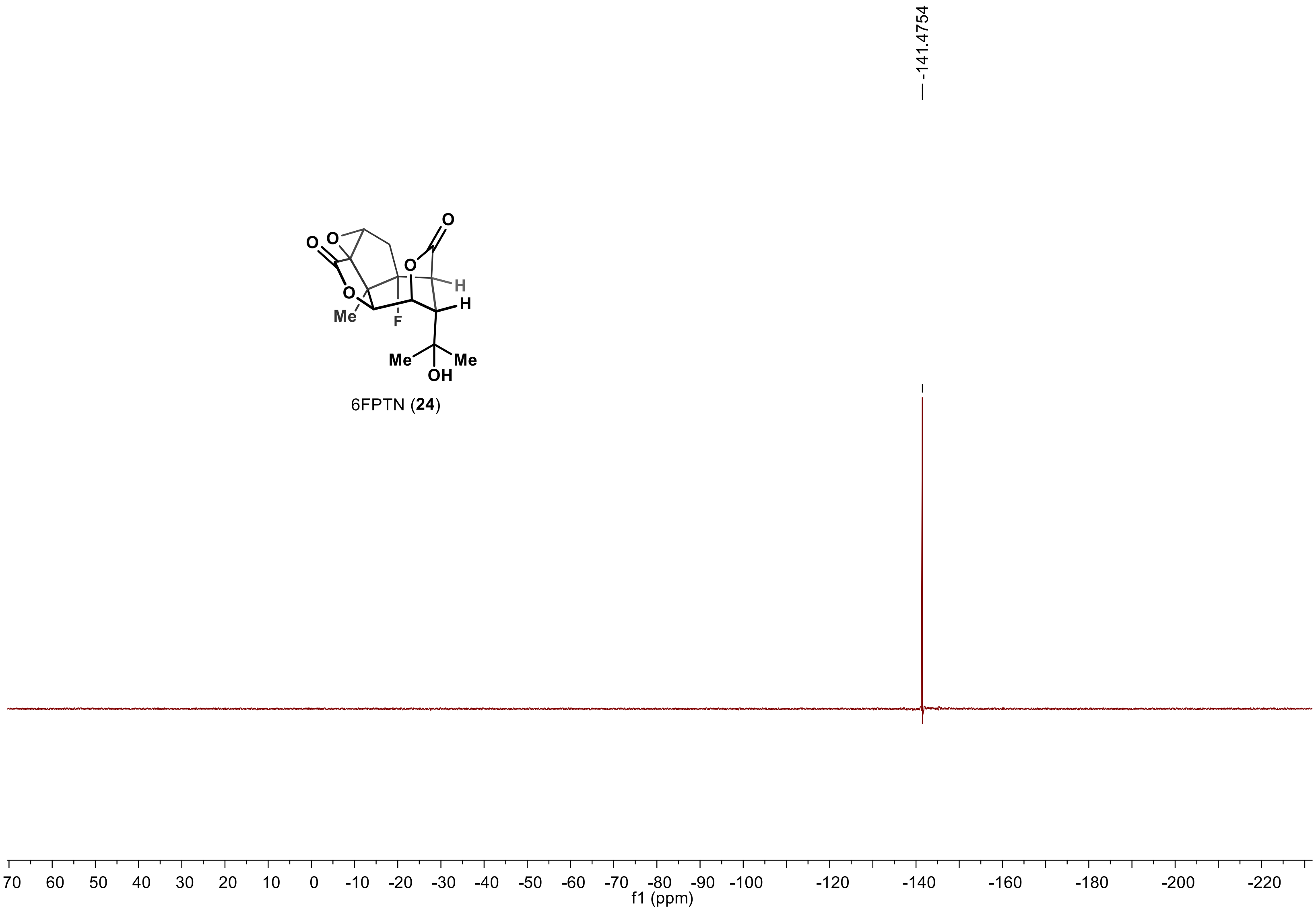

^{19}F NMR, $CDCl_3$, 375 MHz

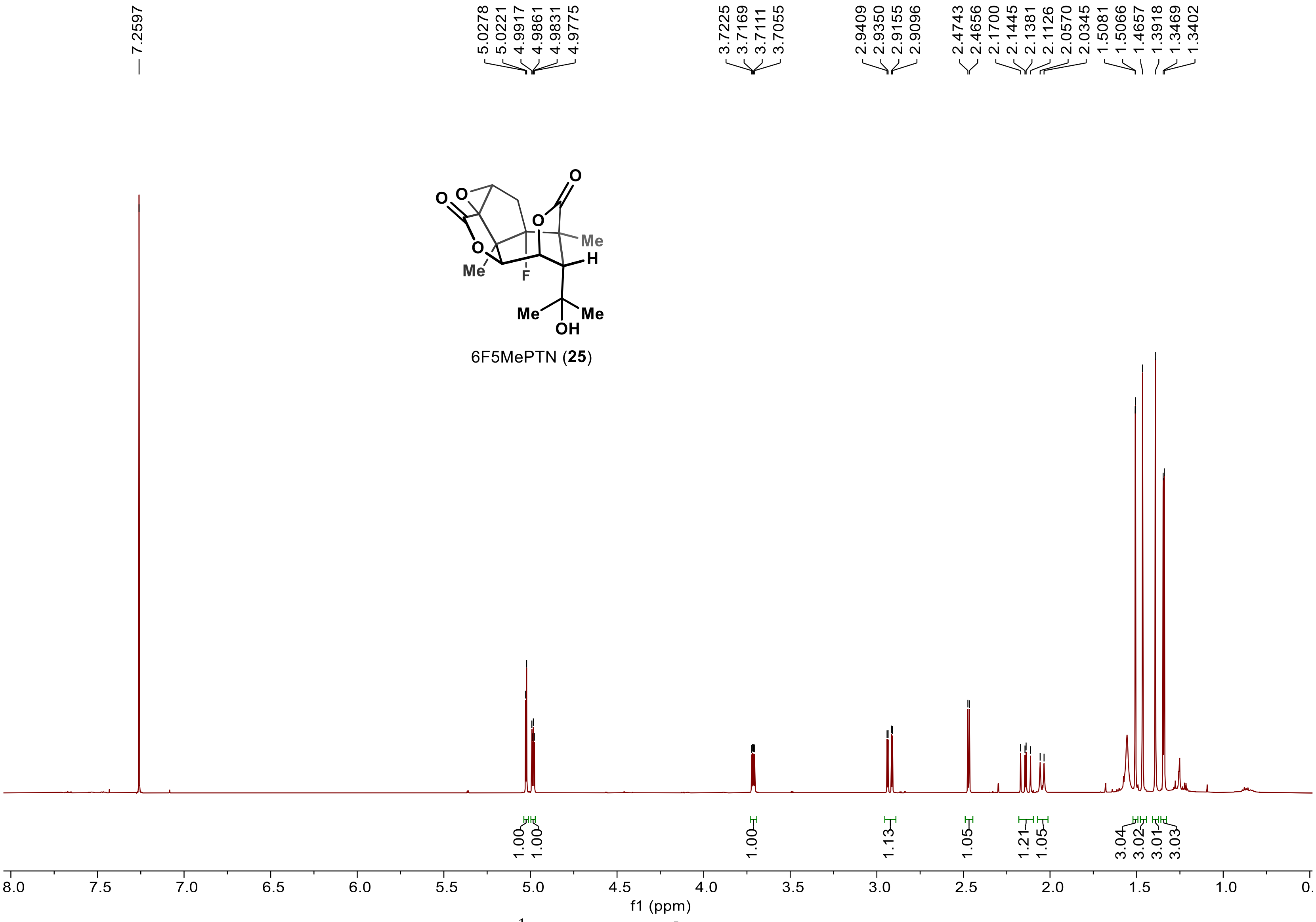

^{1}H NMR, $CDCl_3$, 600 MHz

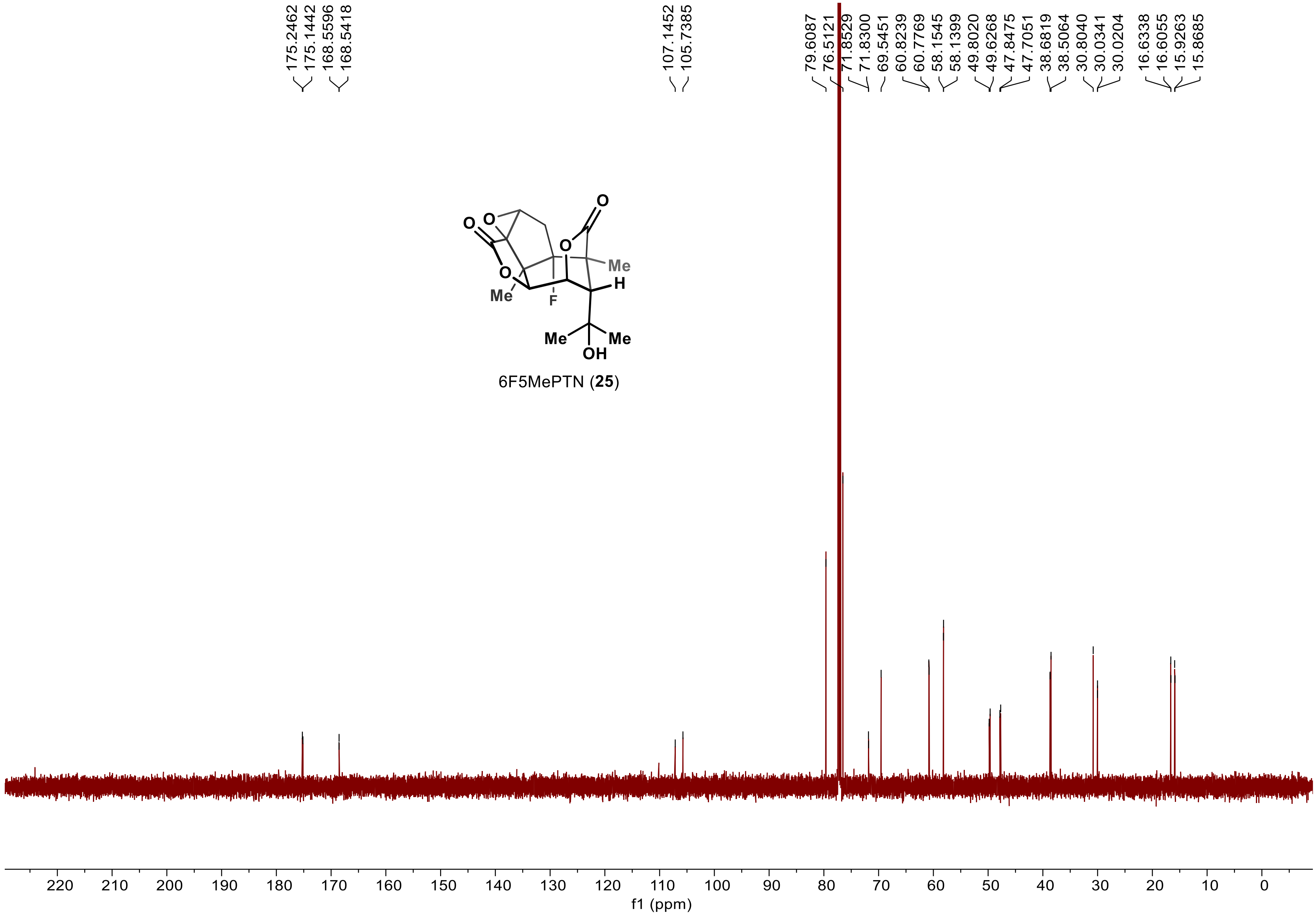

^{13}C NMR, $CDCl_3$, 150 MHz

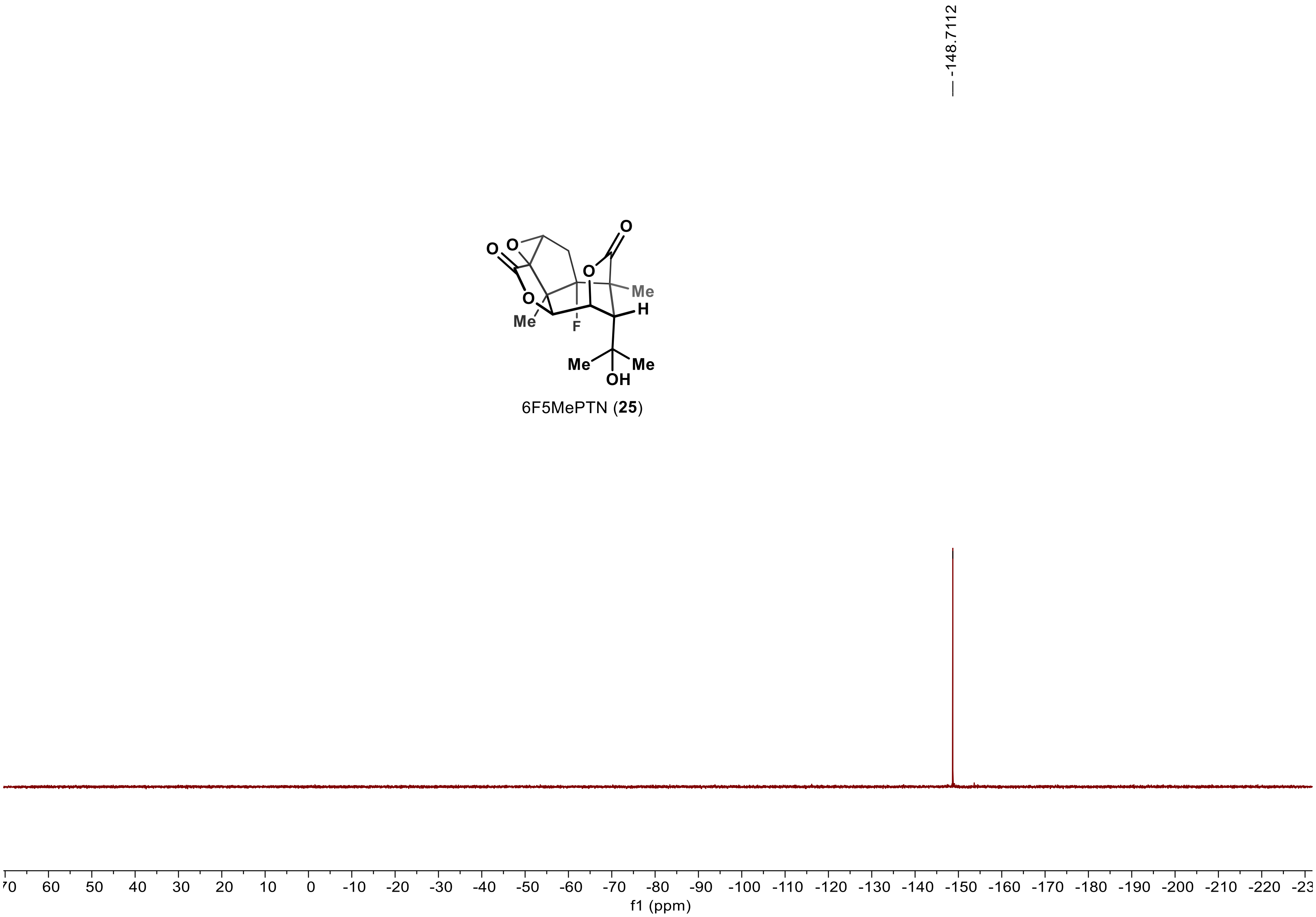

6F5MePTN (**25**)

^{19}F NMR, $CDCl_3$, 375 MHz

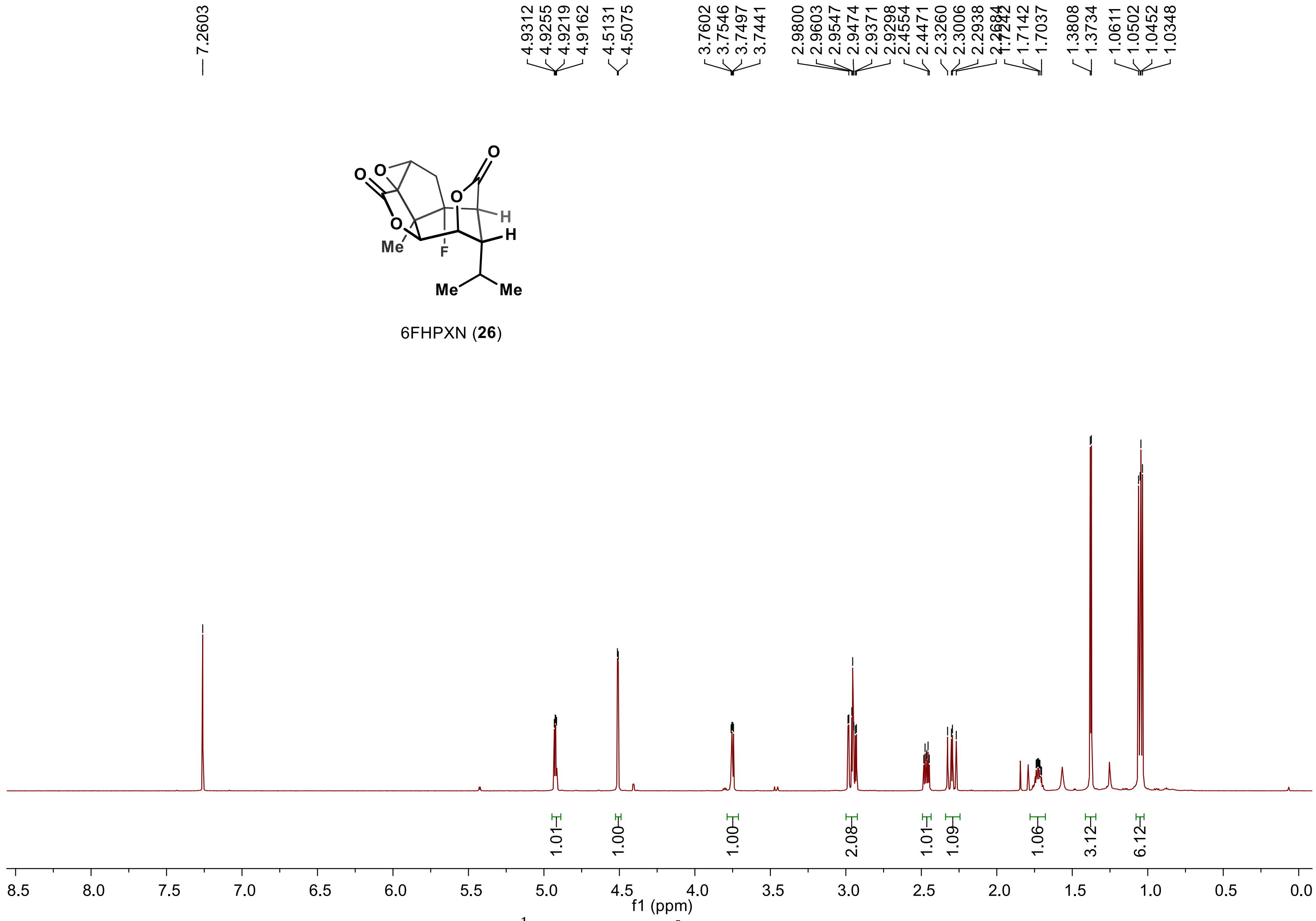

^1H NMR, $CDCl_3$, 600 MHz

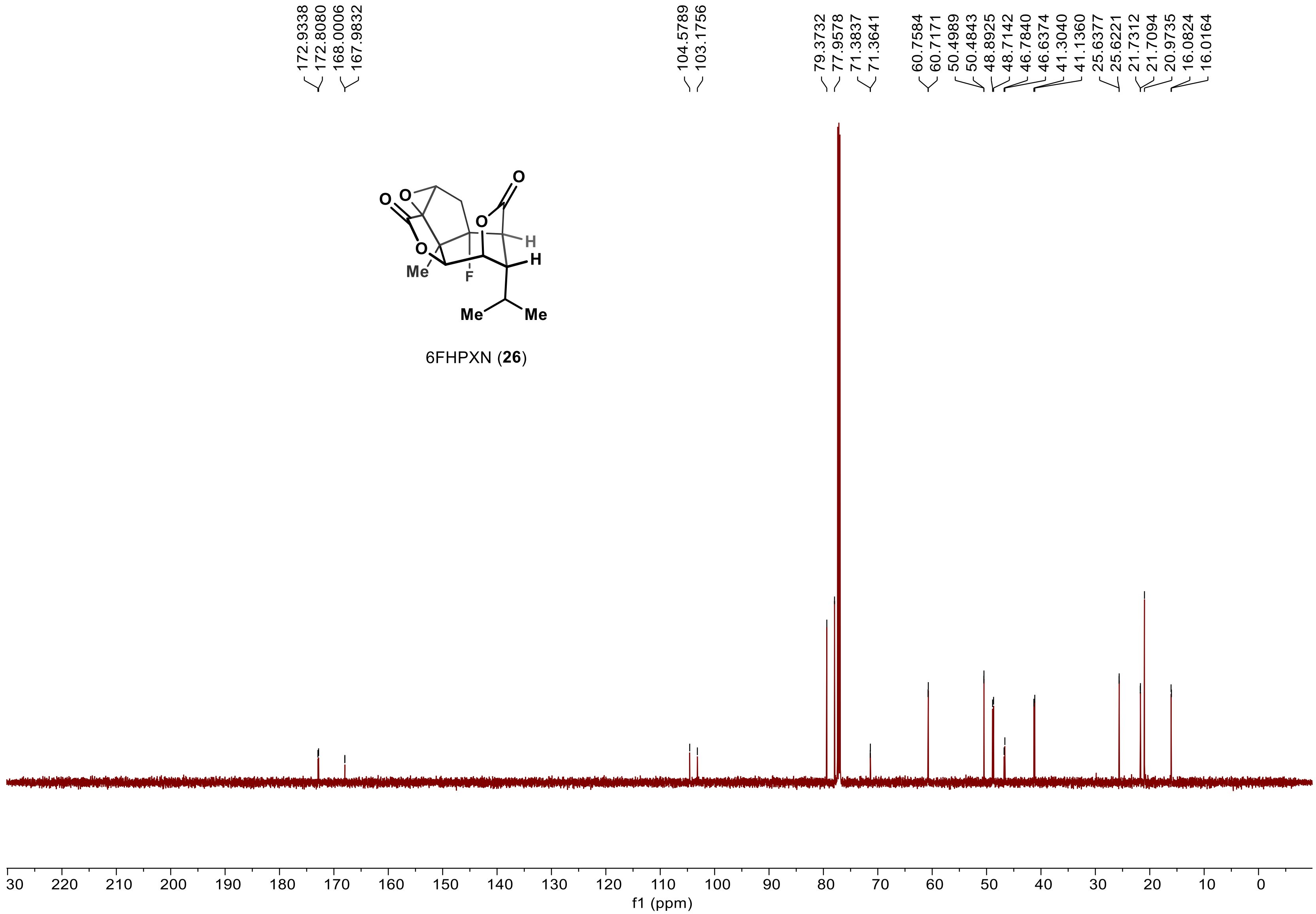

^{13}C NMR, $CDCl_3$, 150 MHz

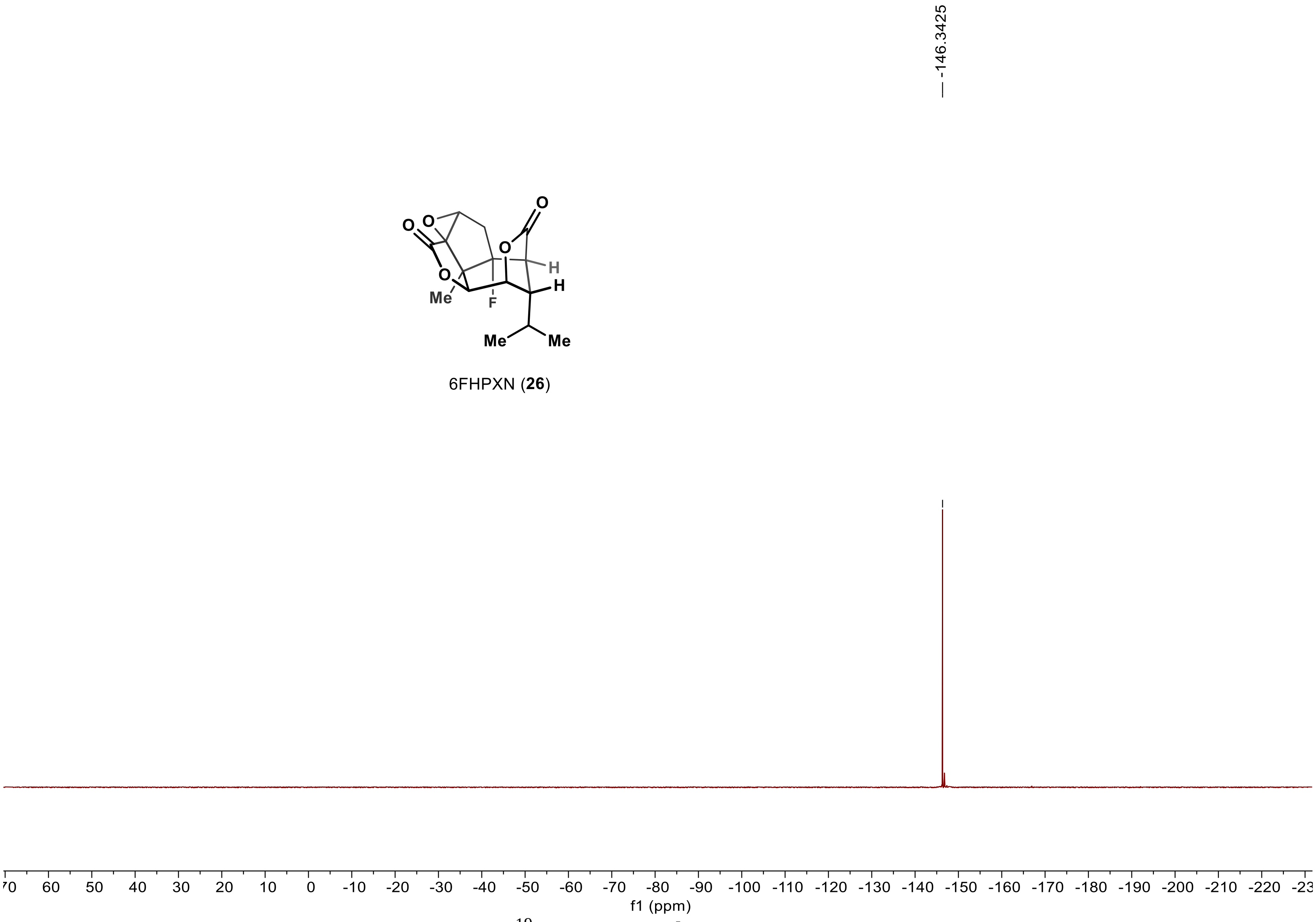

6FHPXN (**26**)

^{19}F NMR, $CDCl_3$, 375 MHz

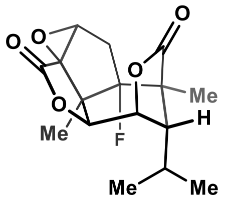

6FH5MePXN (**27**)

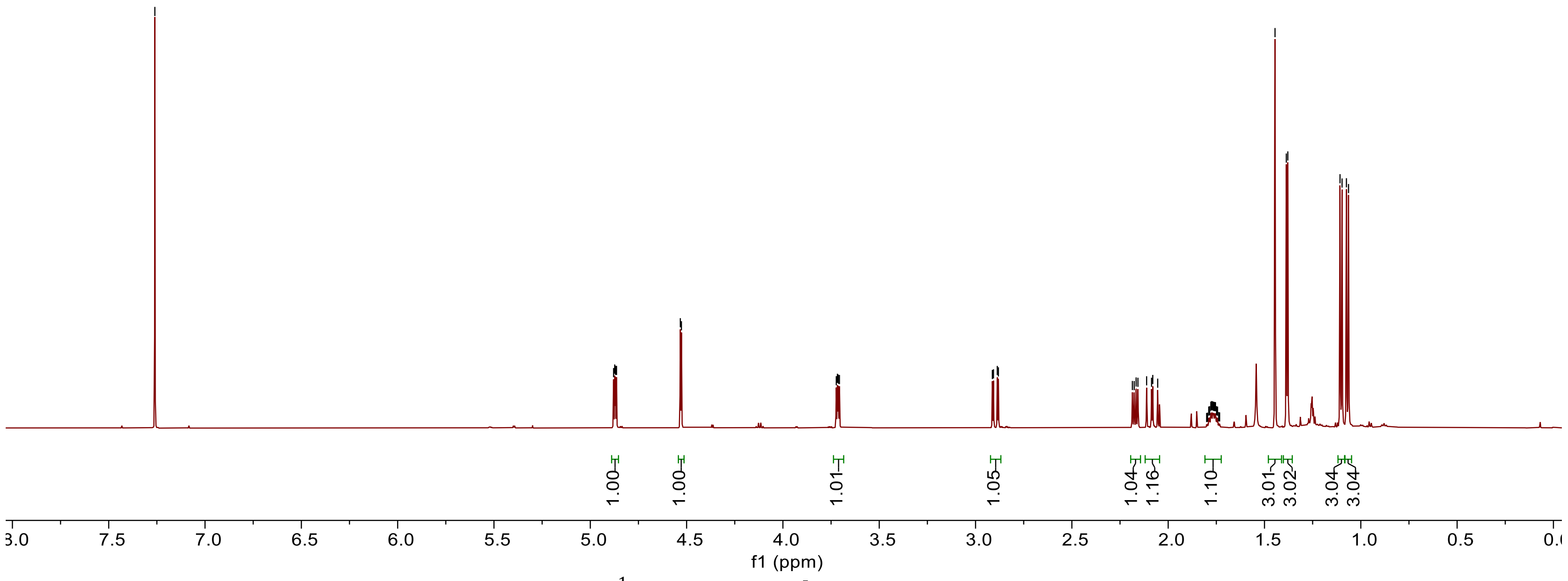

^{1}H NMR, $CDCl_3$, 600 MHz

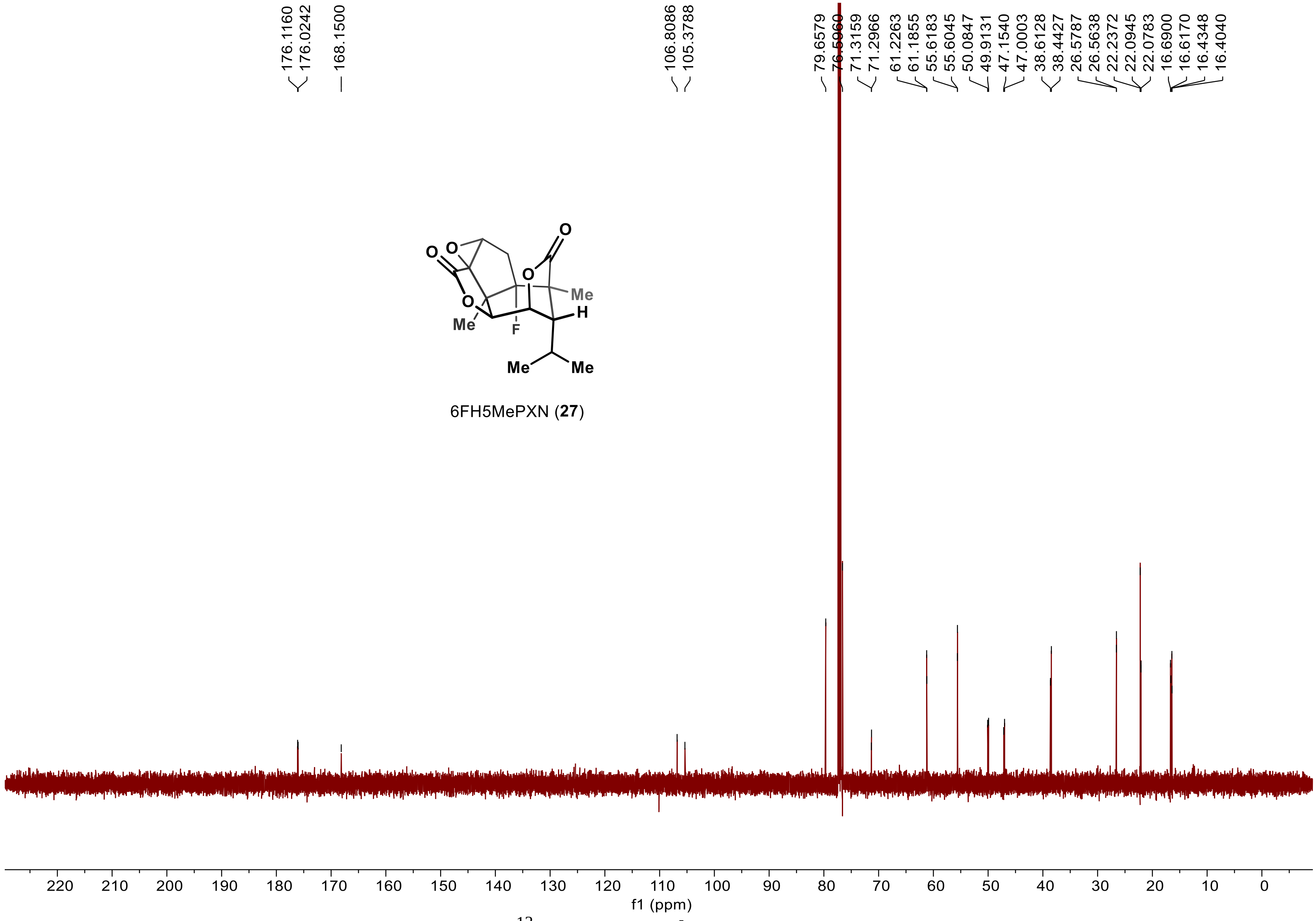

^{13}C NMR, $CDCl_3$, 150 MHz

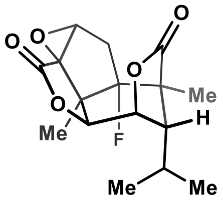

6FH5MePXN (**27**)

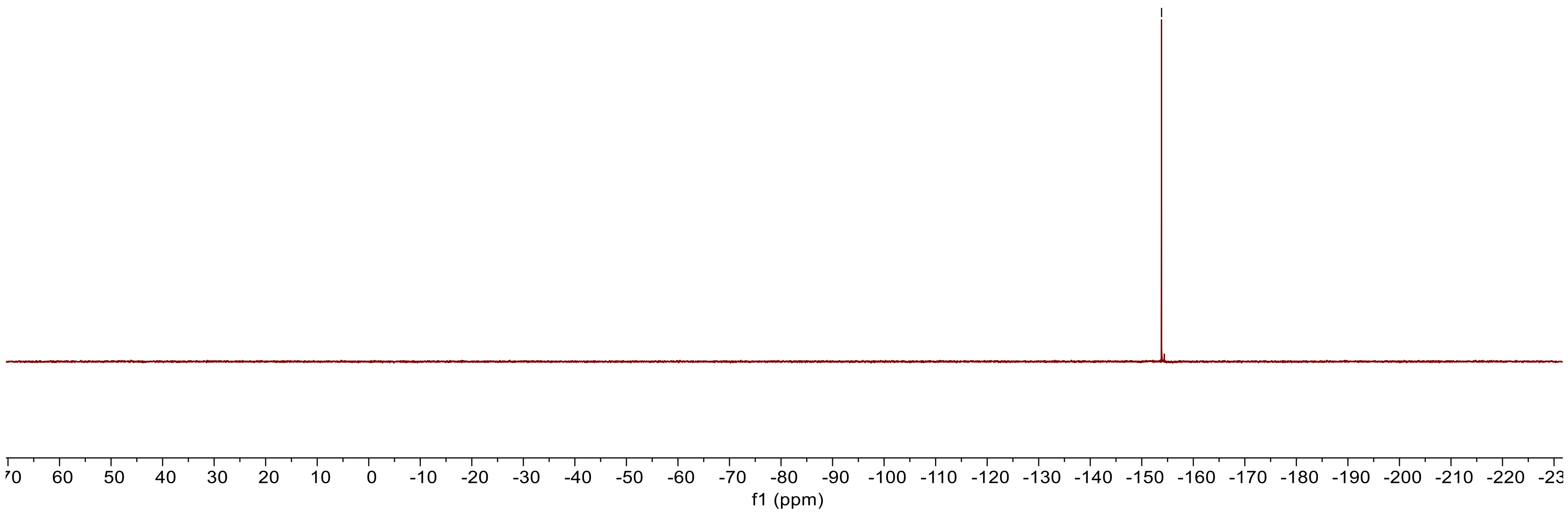

^{19}F NMR, $CDCl_3$, 375 MHz

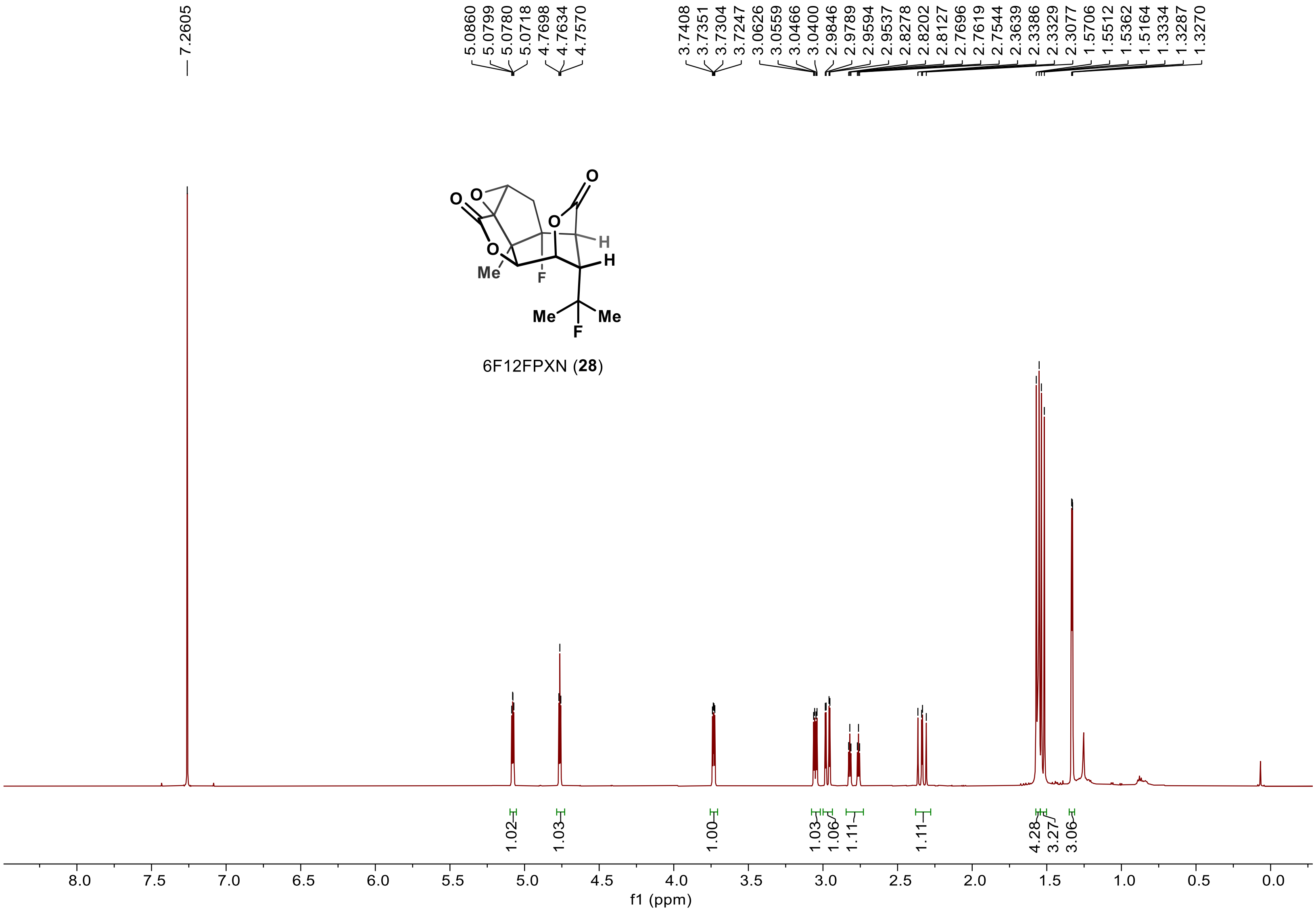

^{1}H NMR, $CDCl_3$, 600 MHz

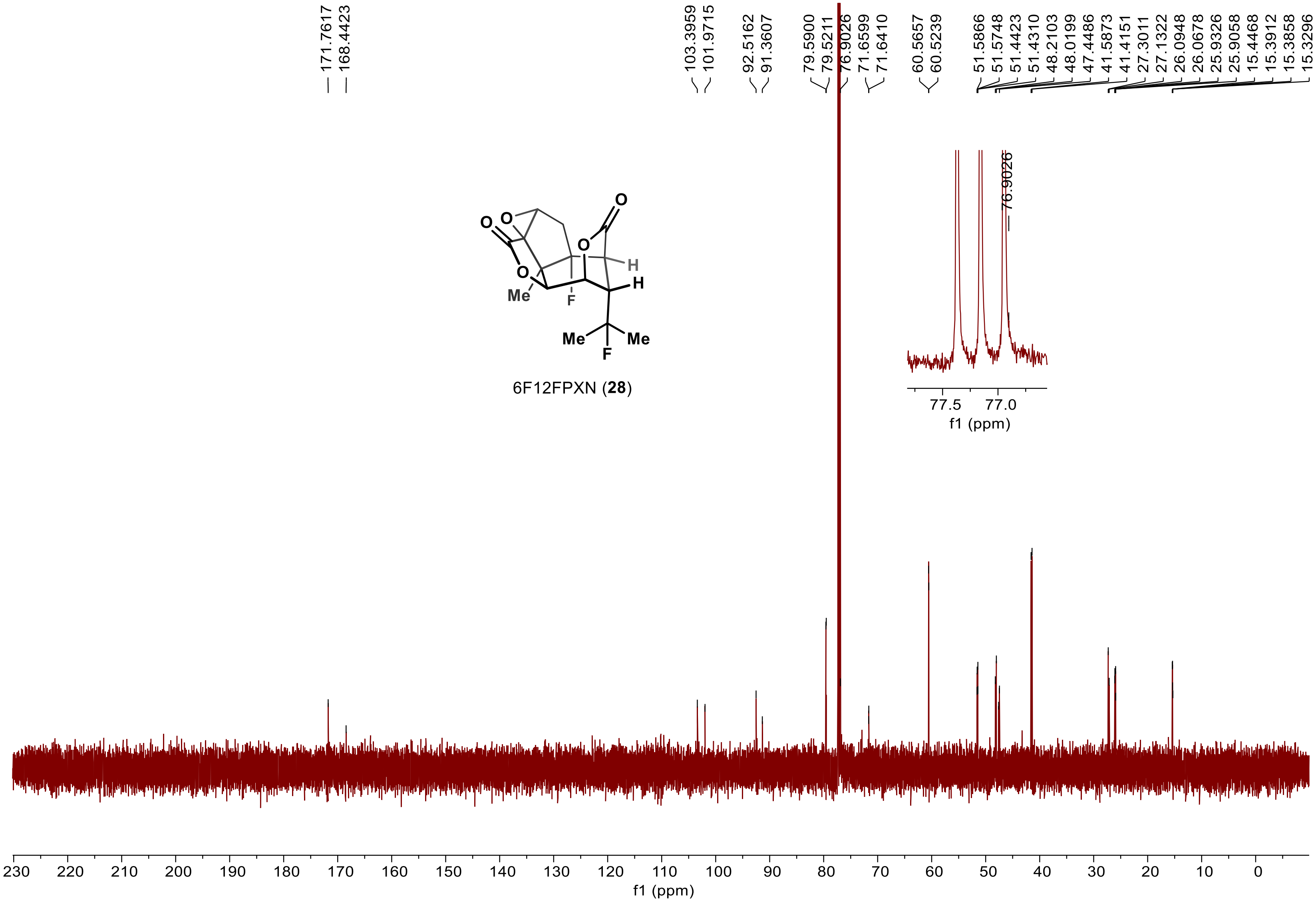

^{13}C NMR, $CDCl_3$, 150 MHz

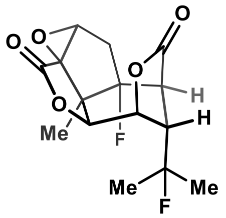

6F12FPXN (**28**)

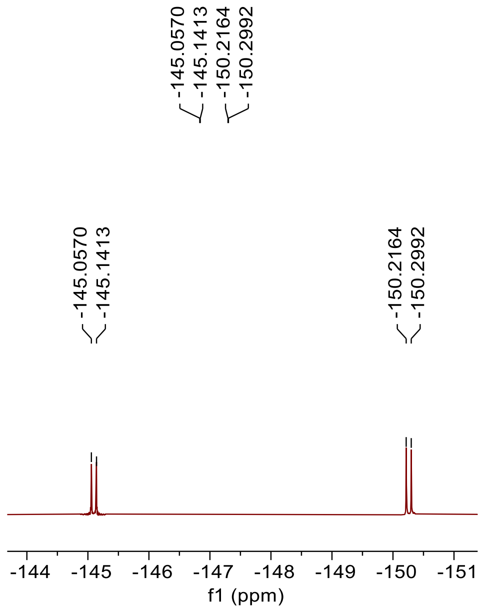

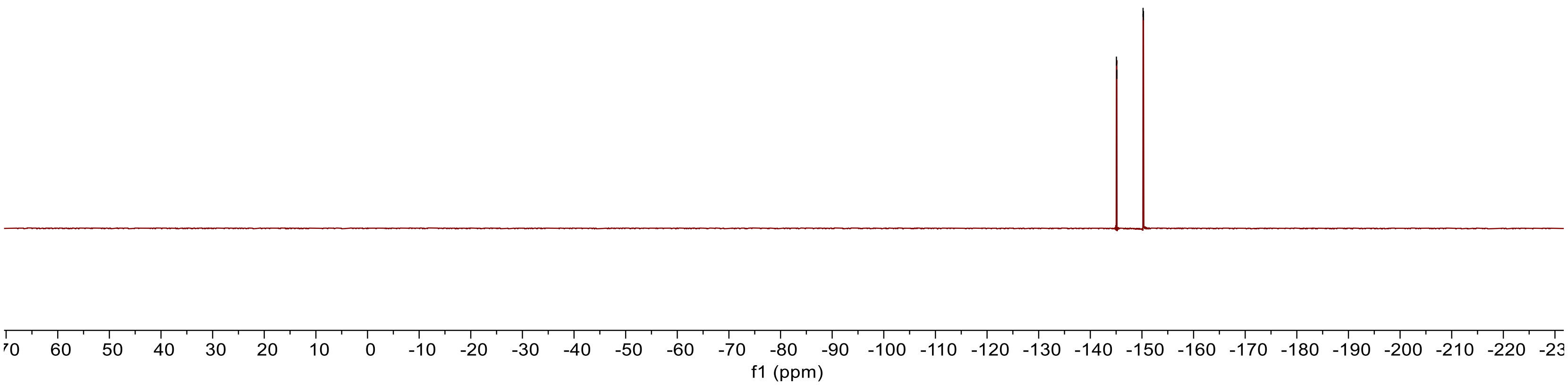

^{19}F NMR, $CDCl_3$, 375 MHz

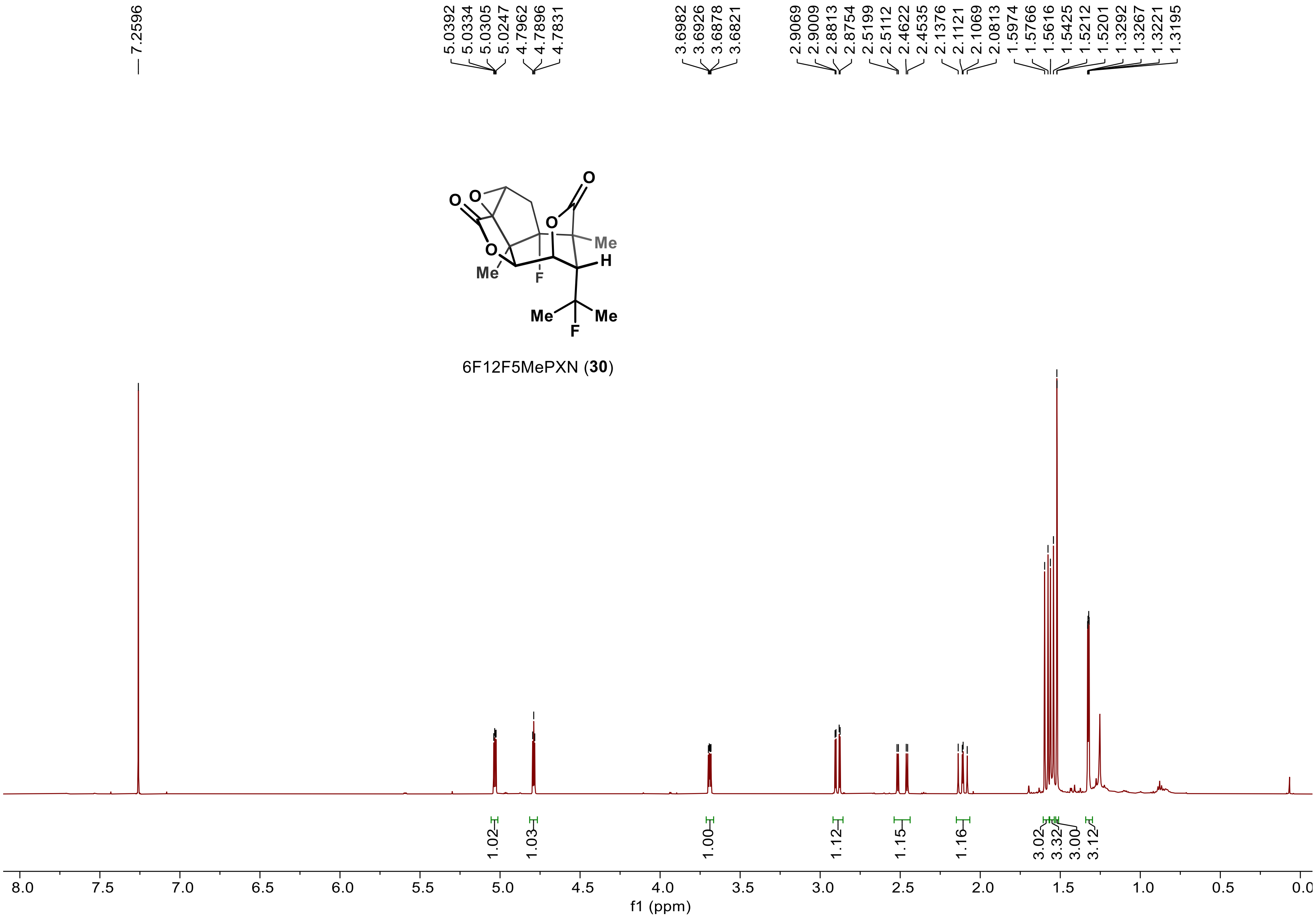

6F12F5MePXN (**30**)

^{1}H NMR, $CDCl_3$, 600 MHz

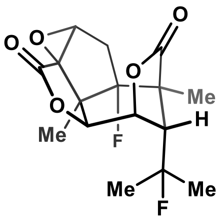

6F12F5MePXN (**30**)

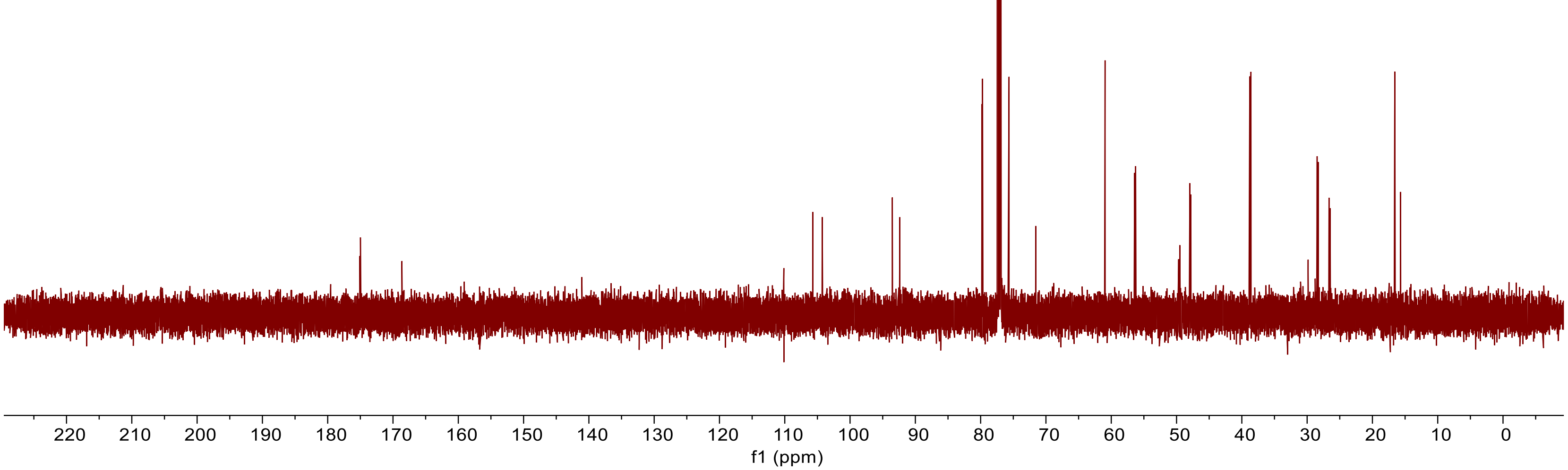

^{13}C NMR, $CDCl_3$, 150 MHz

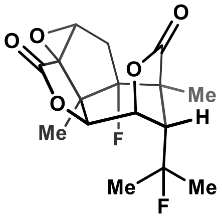

6F12F5MePXN (**30**)

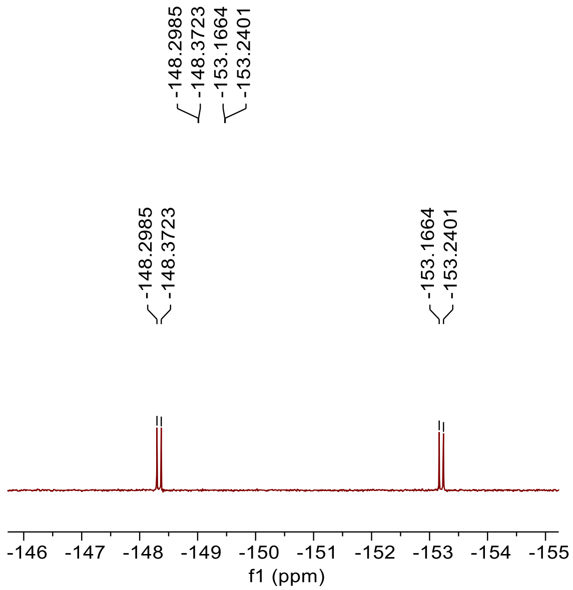

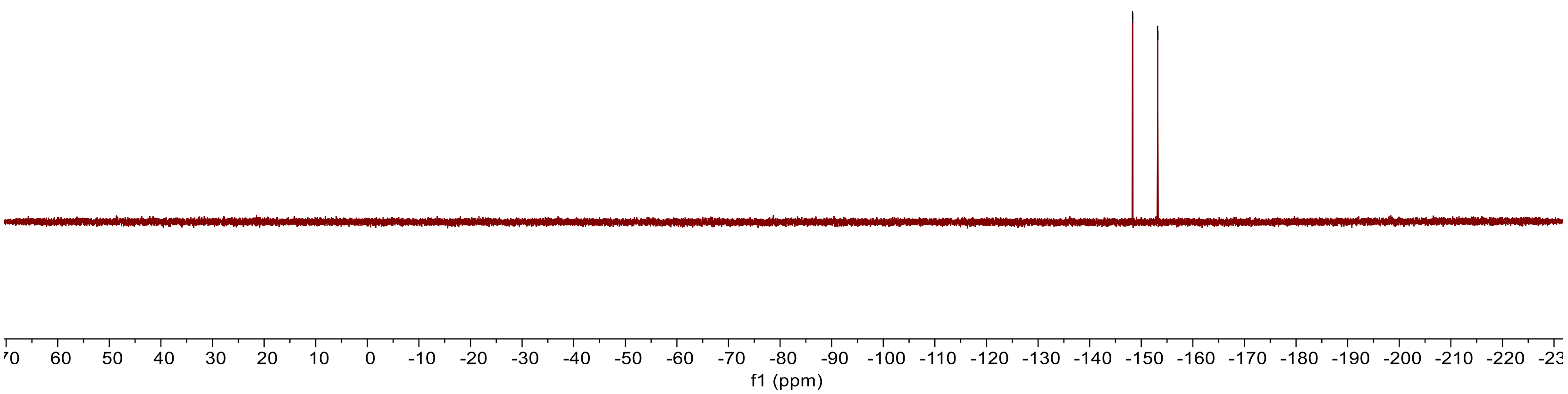

^{19}F NMR, $CDCl_3$, 375 MHz

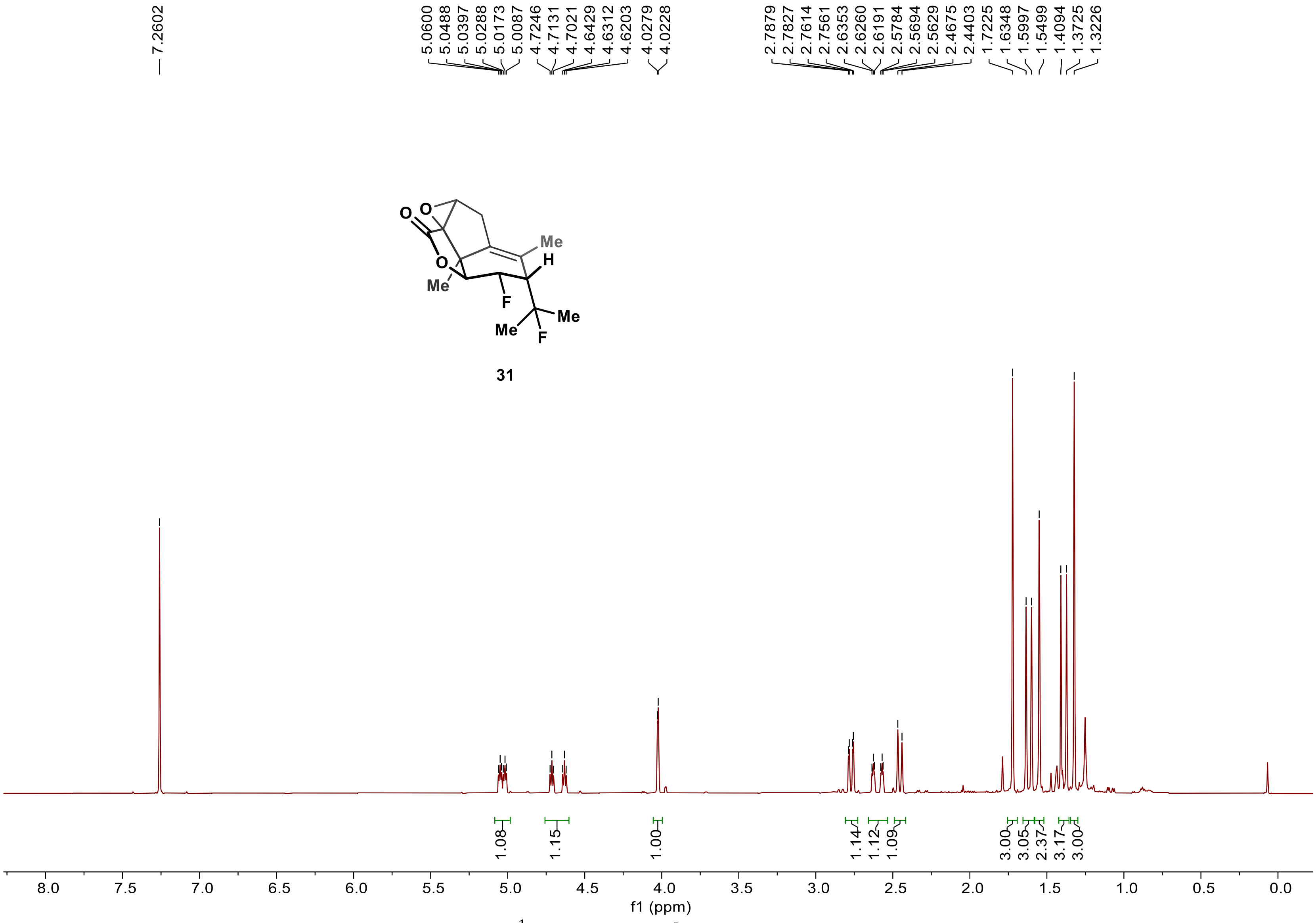

^{1}H NMR, $CDCl_3$, 600 MHz

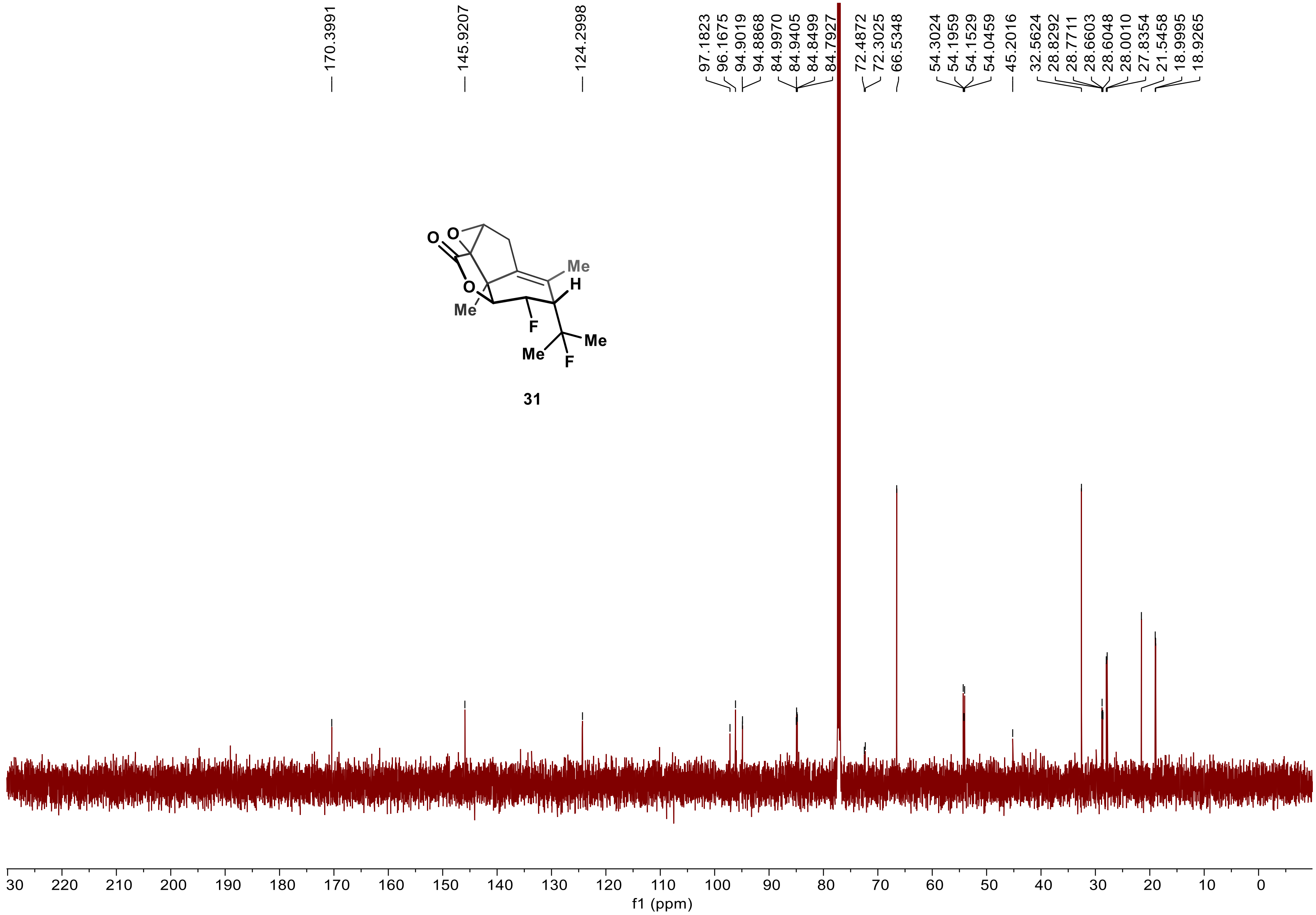

^{13}C NMR, $CDCl_3$, 150 MHz

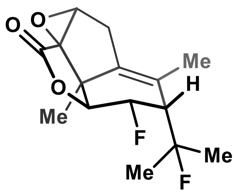

31

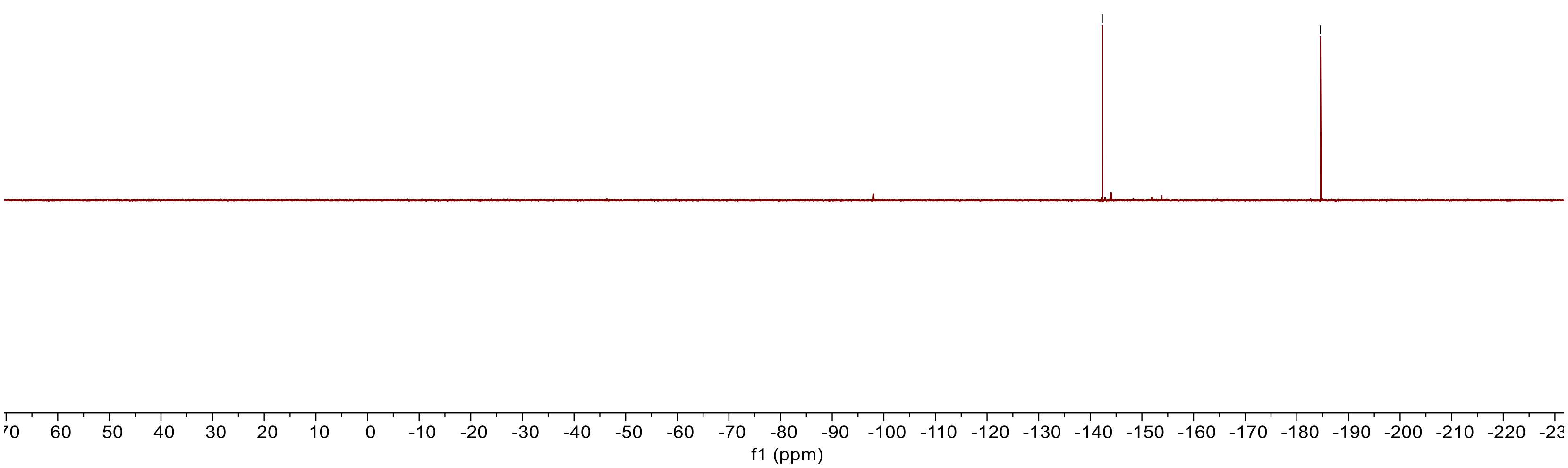

^{19}F NMR, $CDCl_3$, 375 MHz

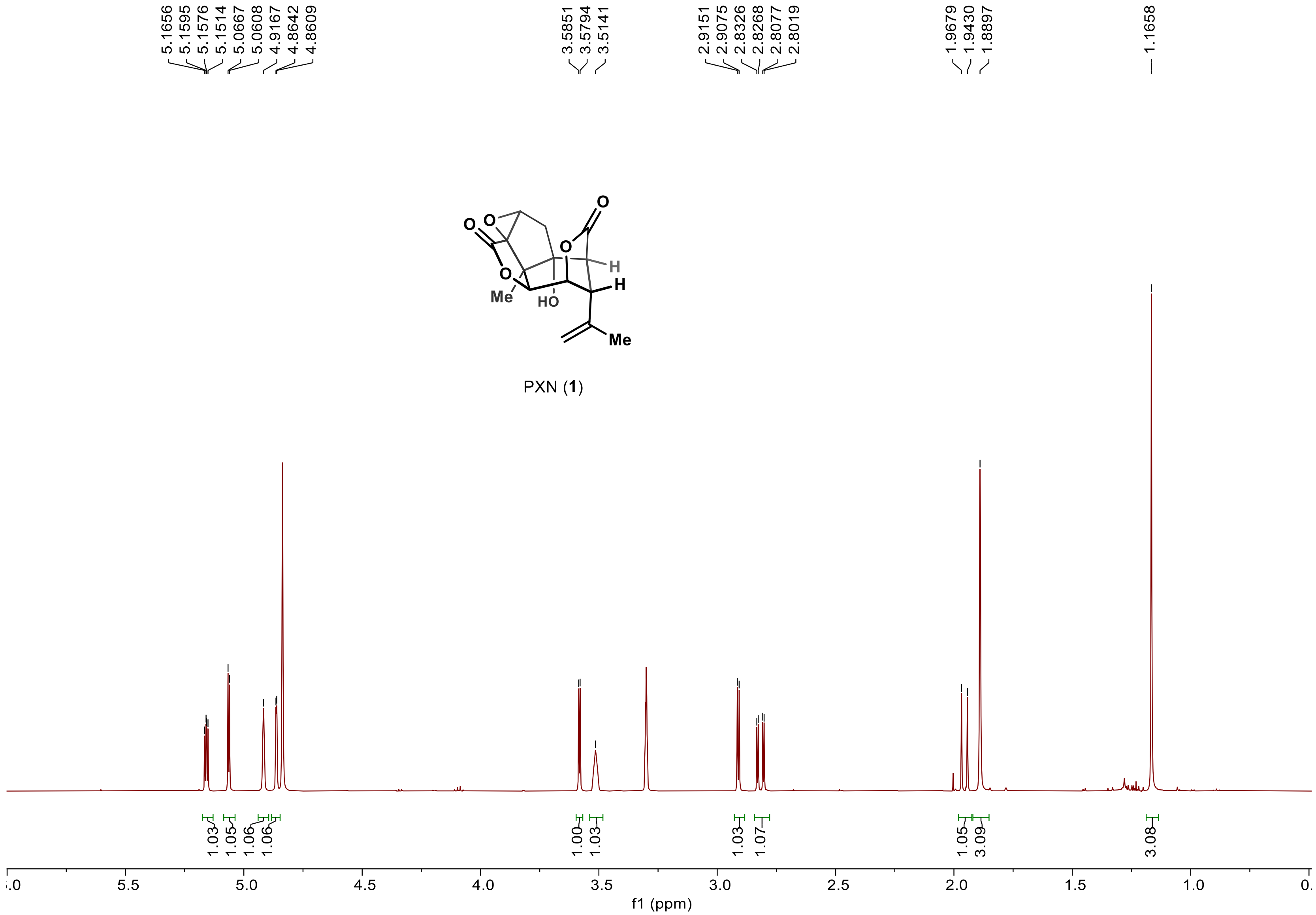

^{1}H NMR, CD_3OD, 600 MHz

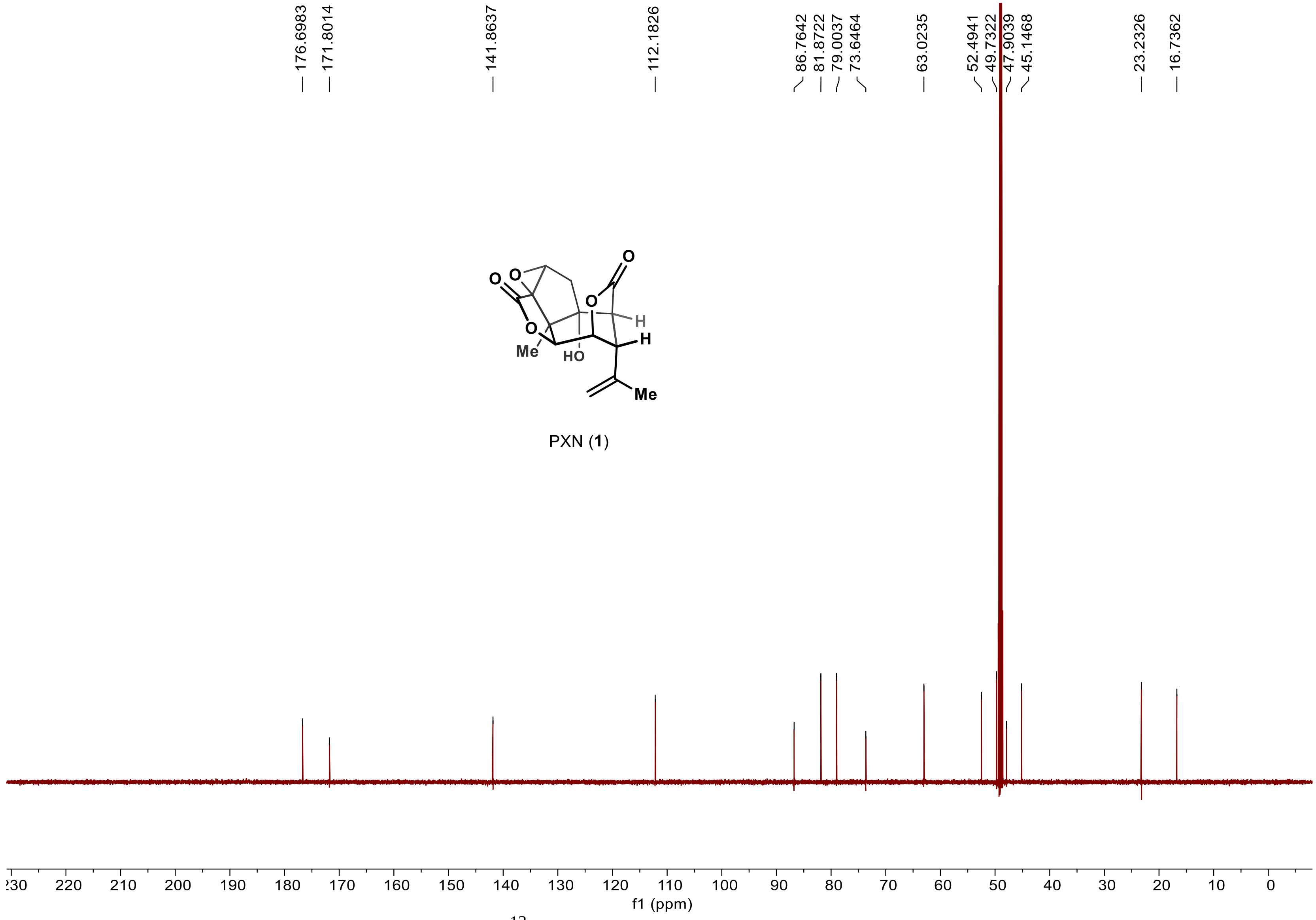

^{13}C NMR, CD_3OD, 150 MHz

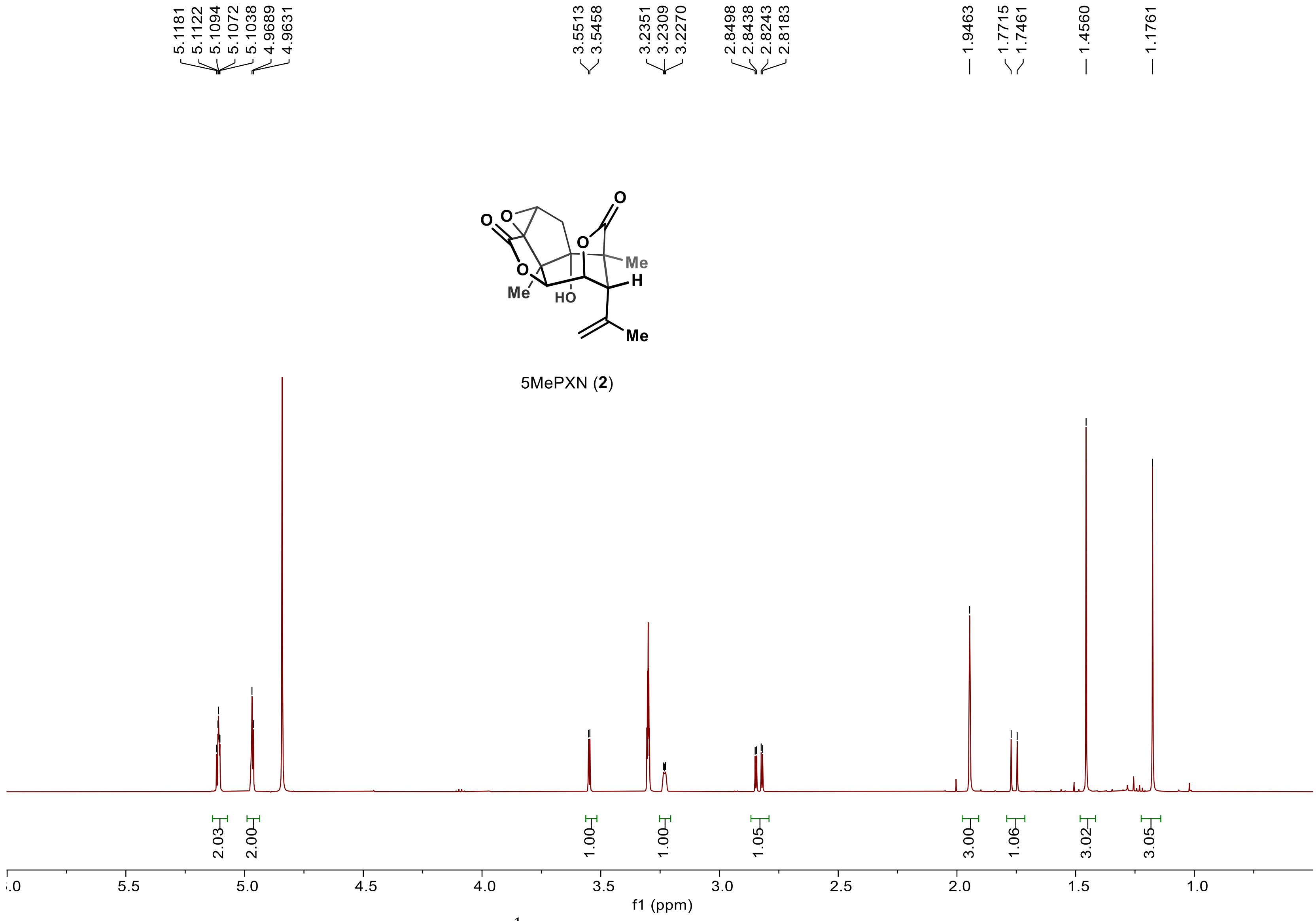

^{1}H NMR, CD_3OD, 600 MHz

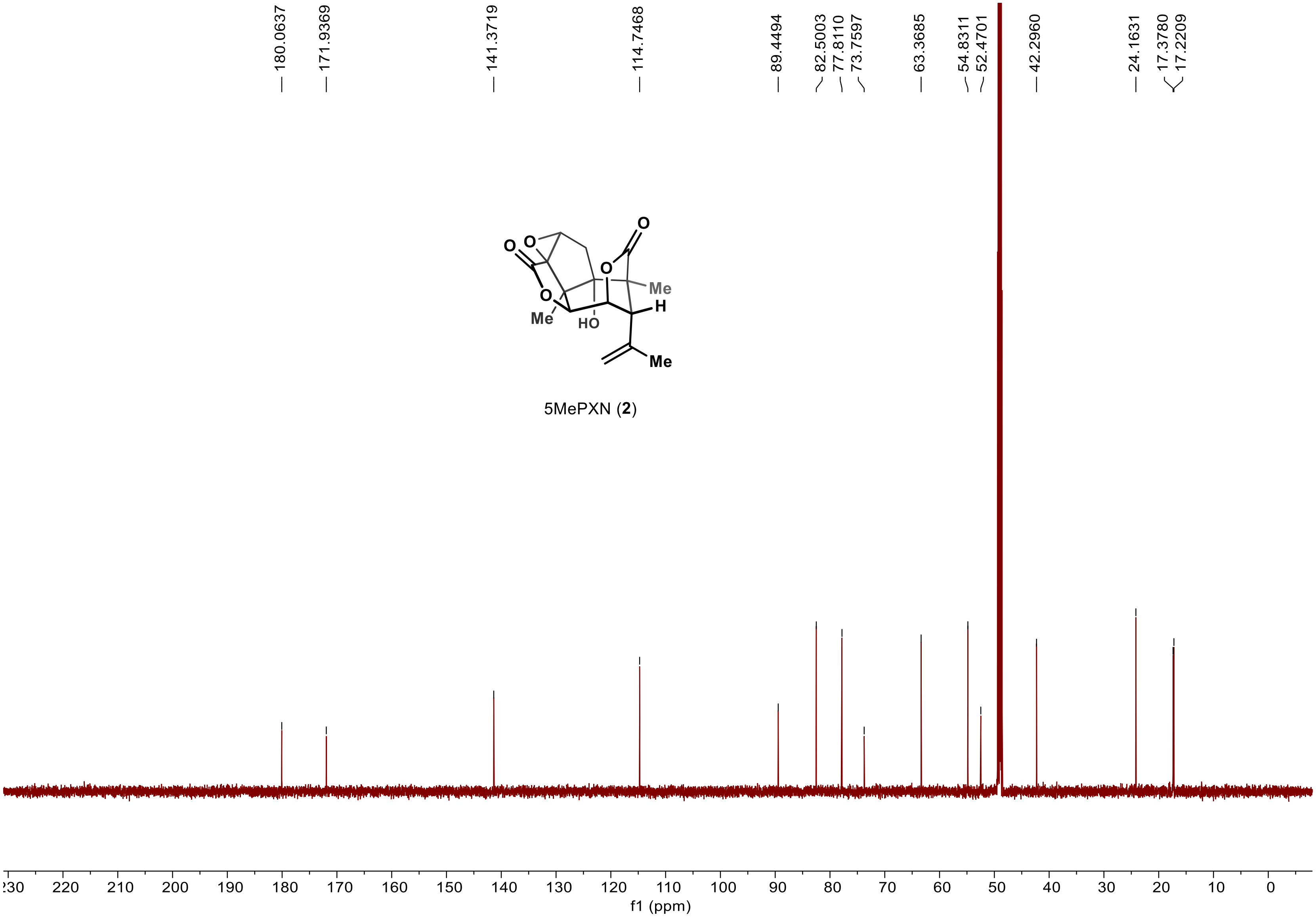

^{13}C NMR, CD_3OD, 150 MHz

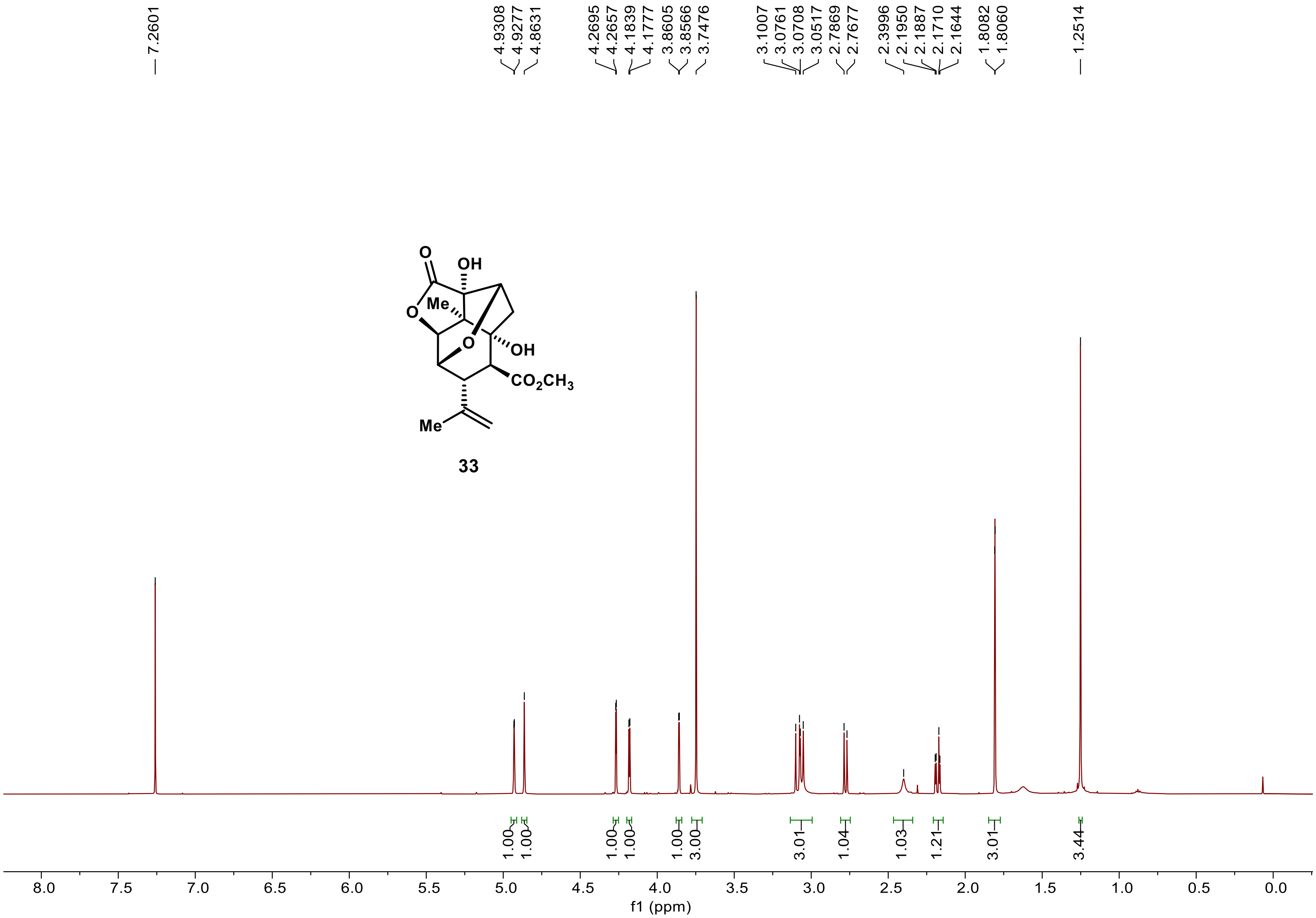

^{1}H NMR, $CDCl_3$, 600 MHz

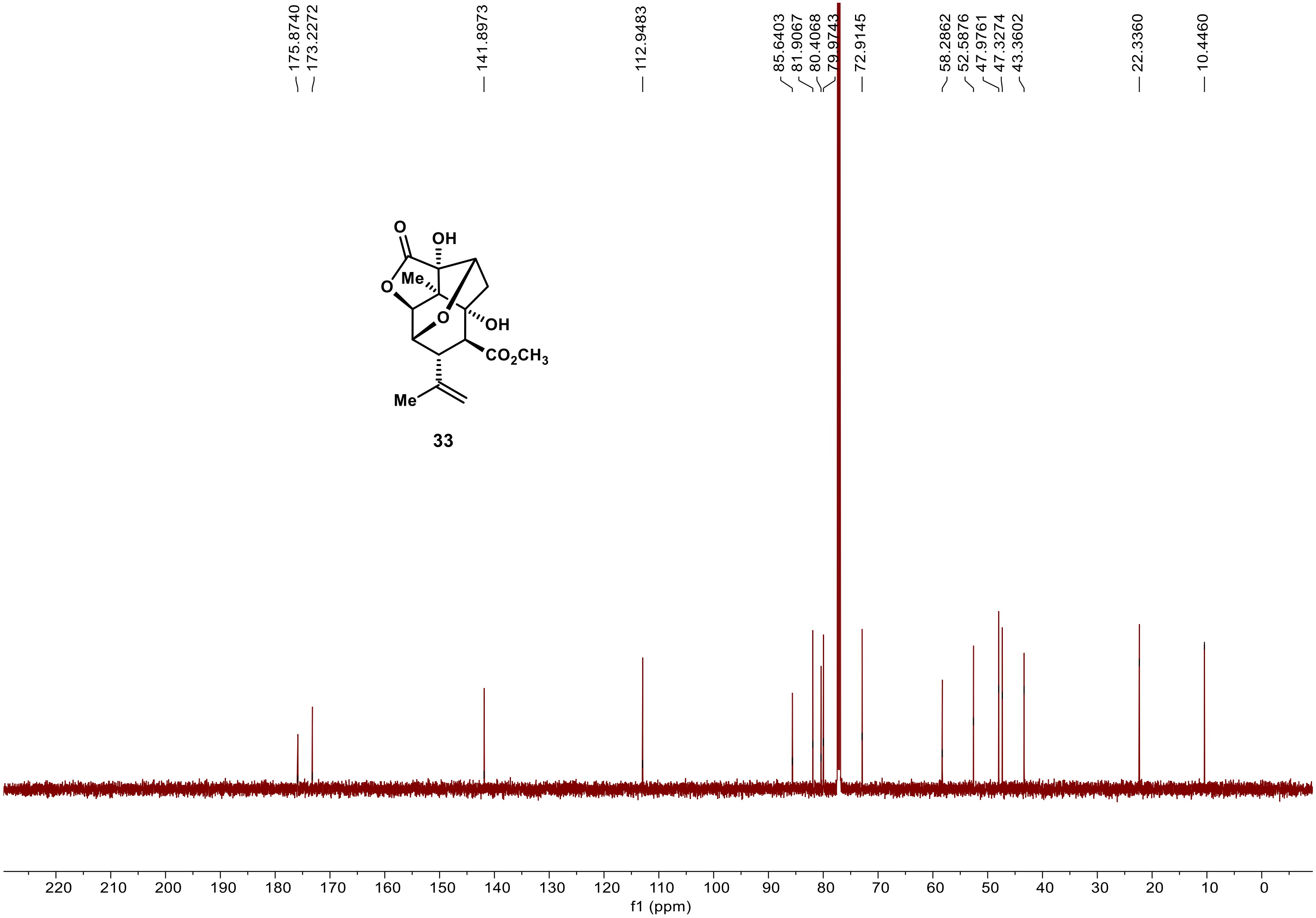

^{13}C NMR, $CDCl_3$, 150 MHz

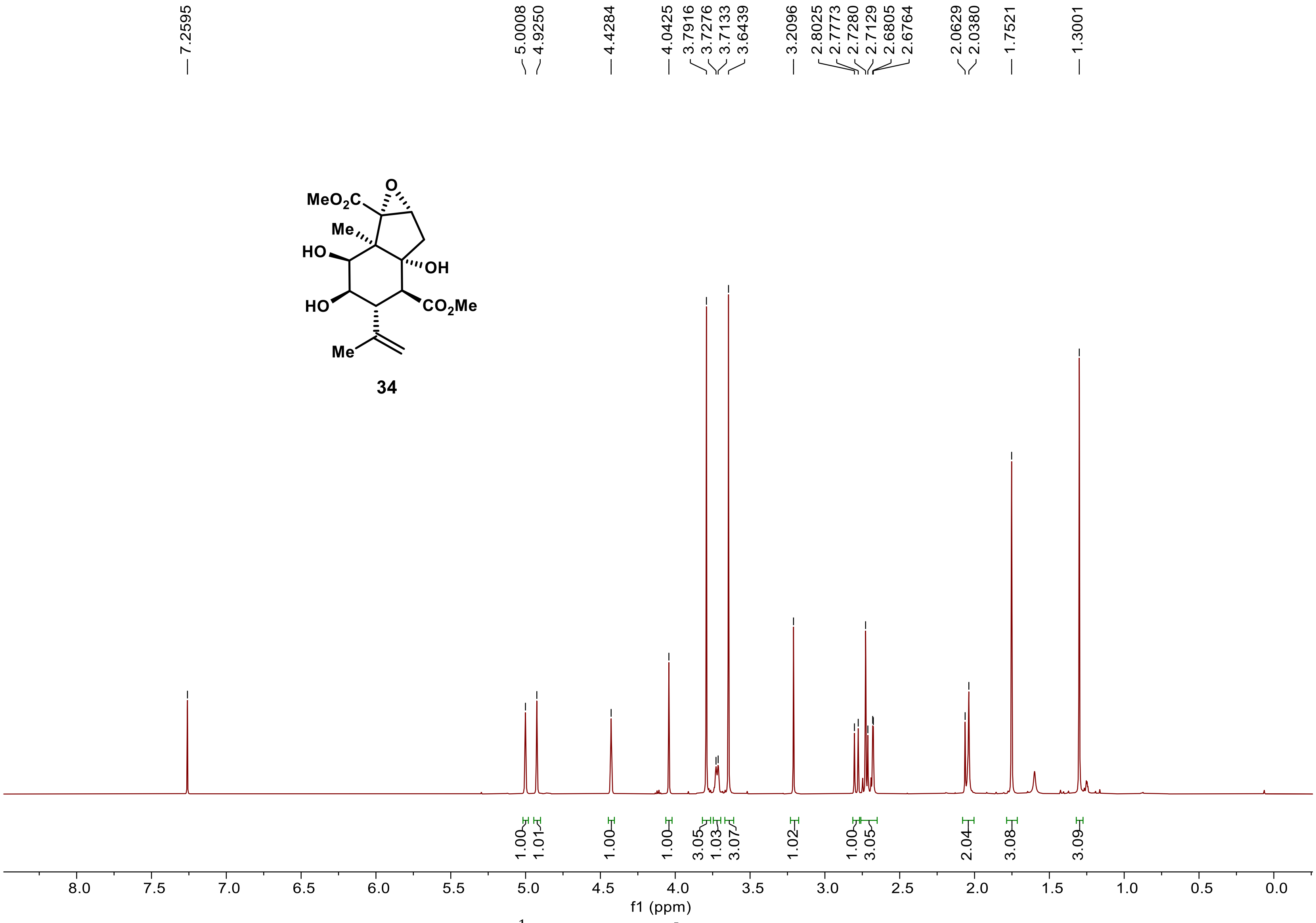

34

^{1}H NMR, $CDCl_3$, 600 MHz

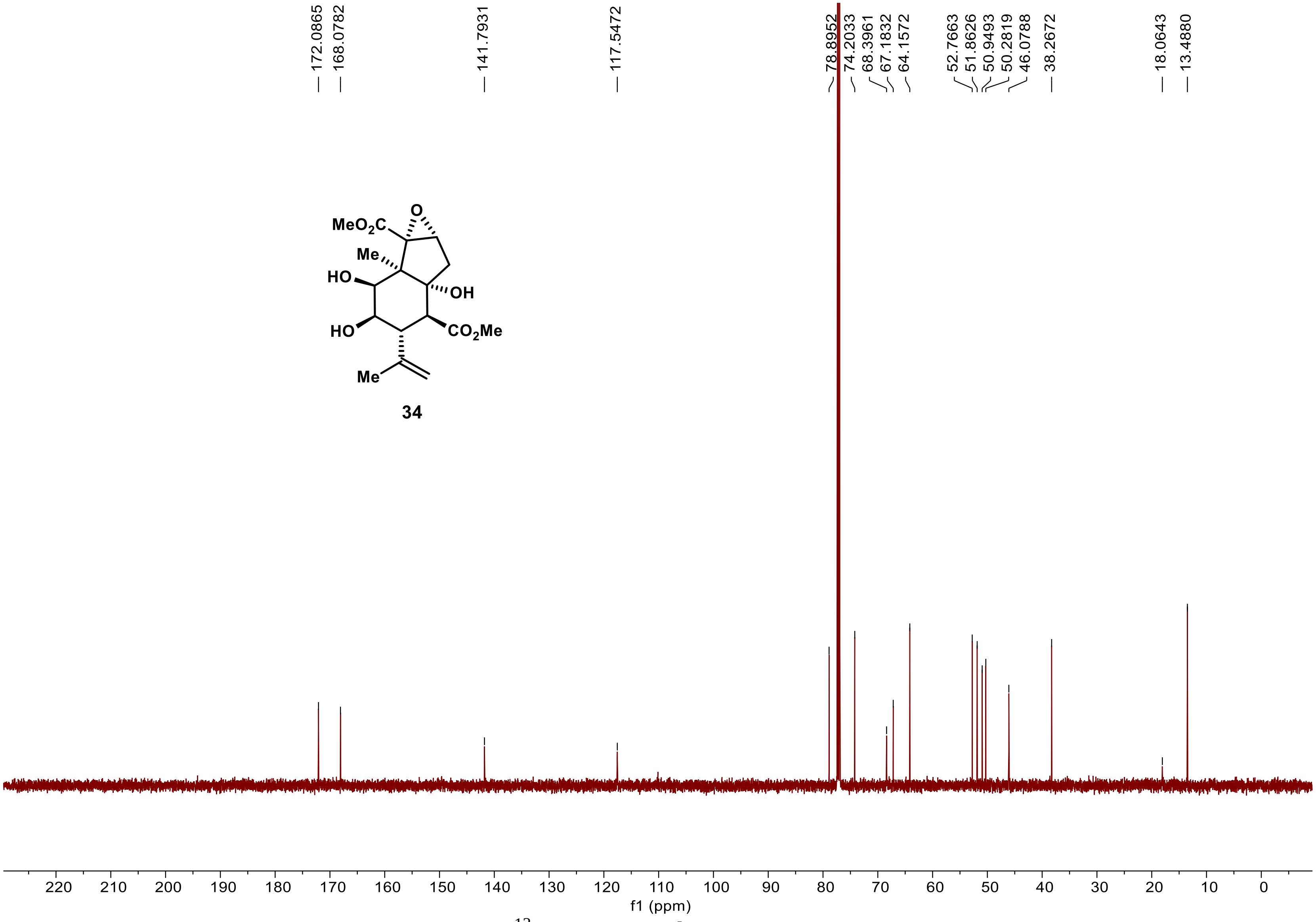

^{13}C NMR, $CDCl_3$, 150 MHz

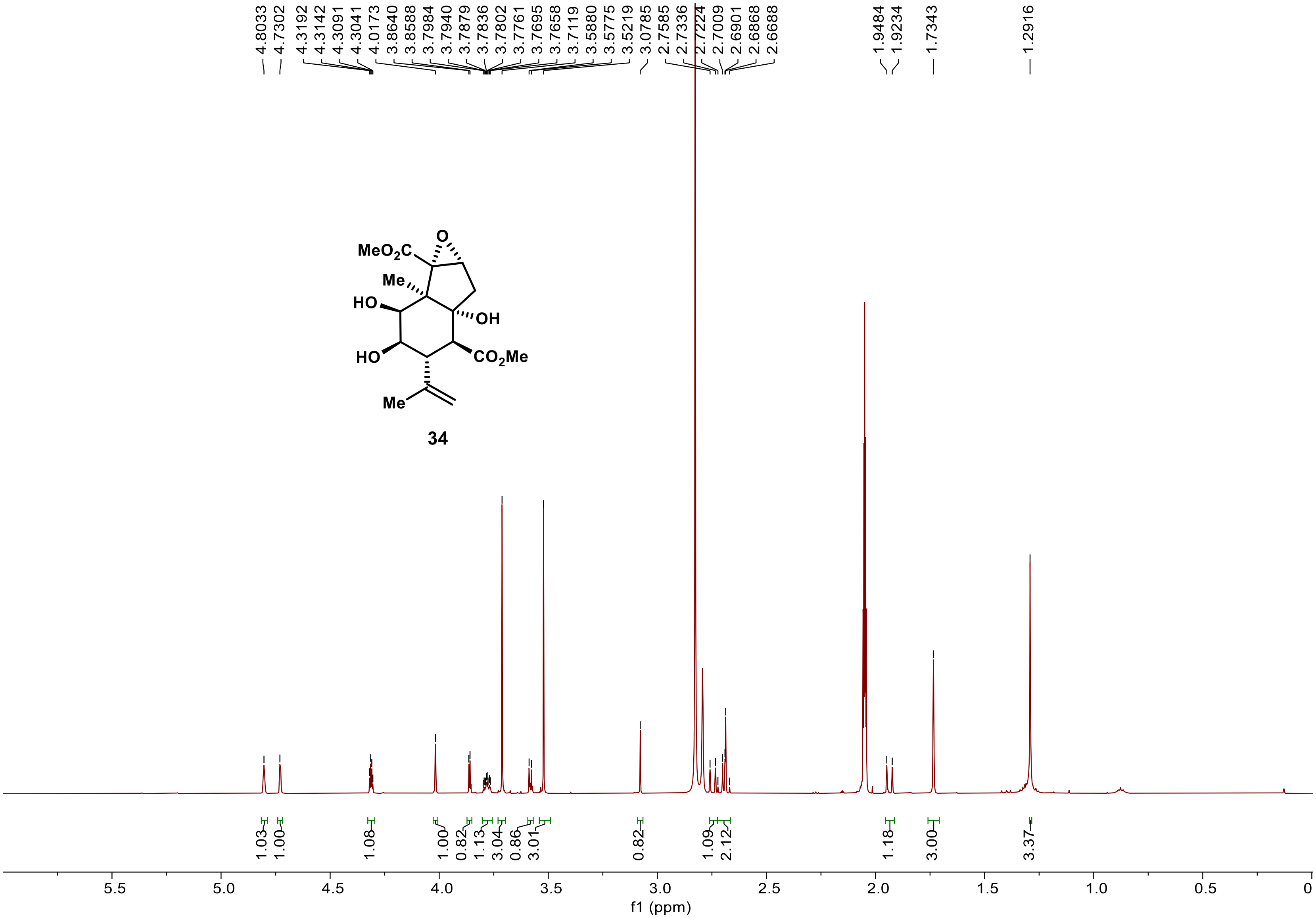

^{1}H NMR, d$_6$-acetone, 600 MHz

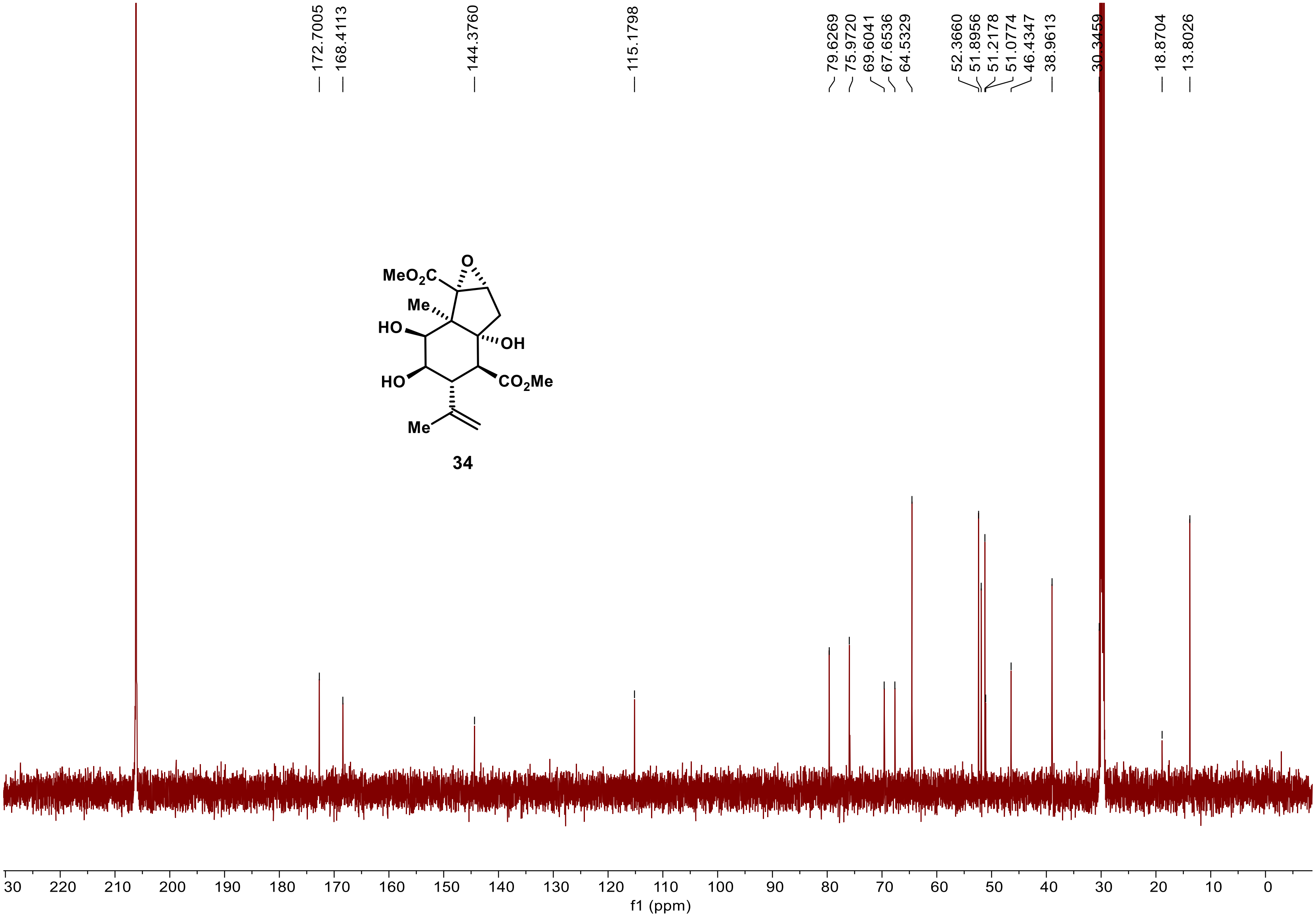

^{13}C NMR, d_6-acetone, 150 MHz

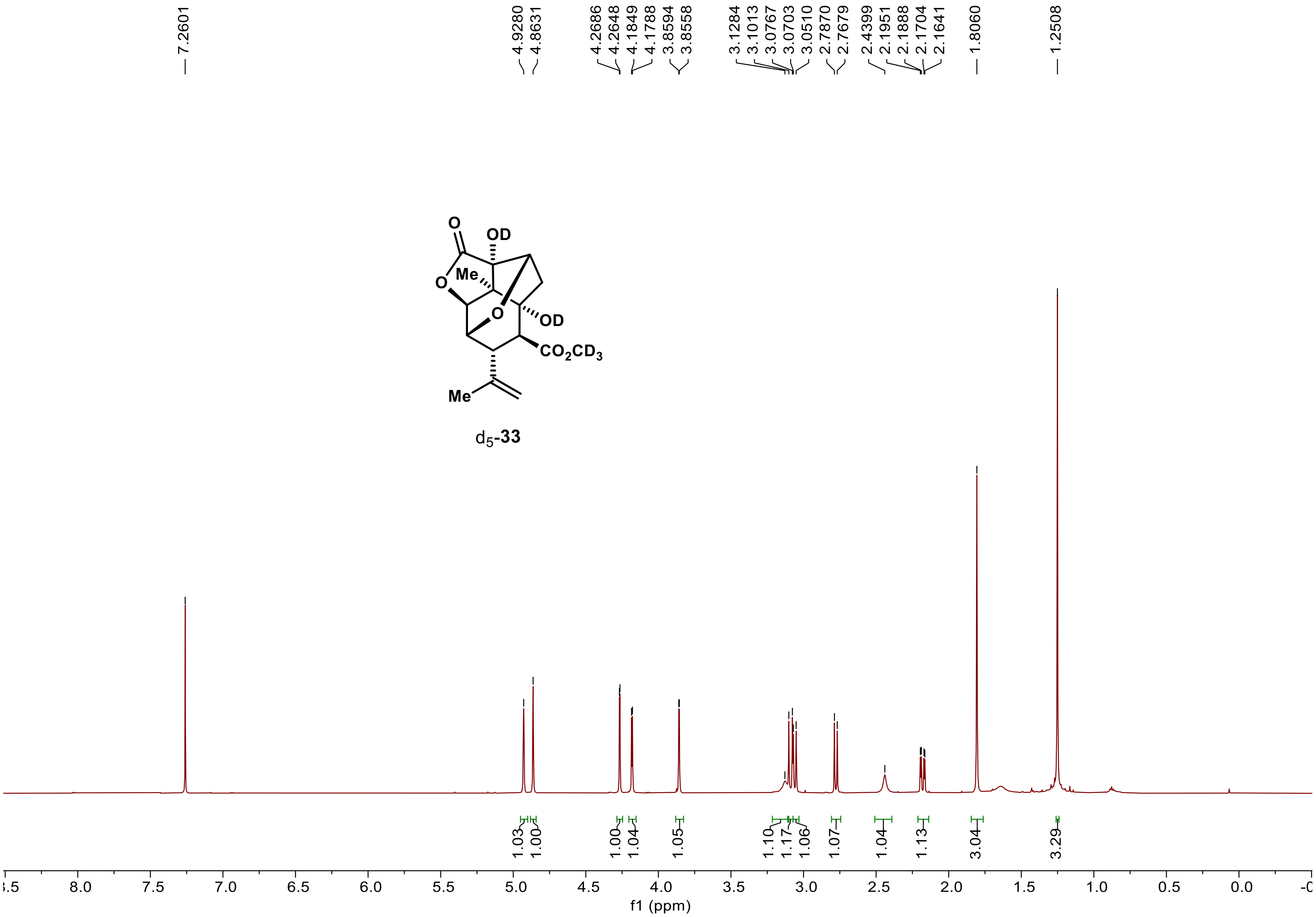

^{1}H NMR, $CDCl_3$, 600 MHz

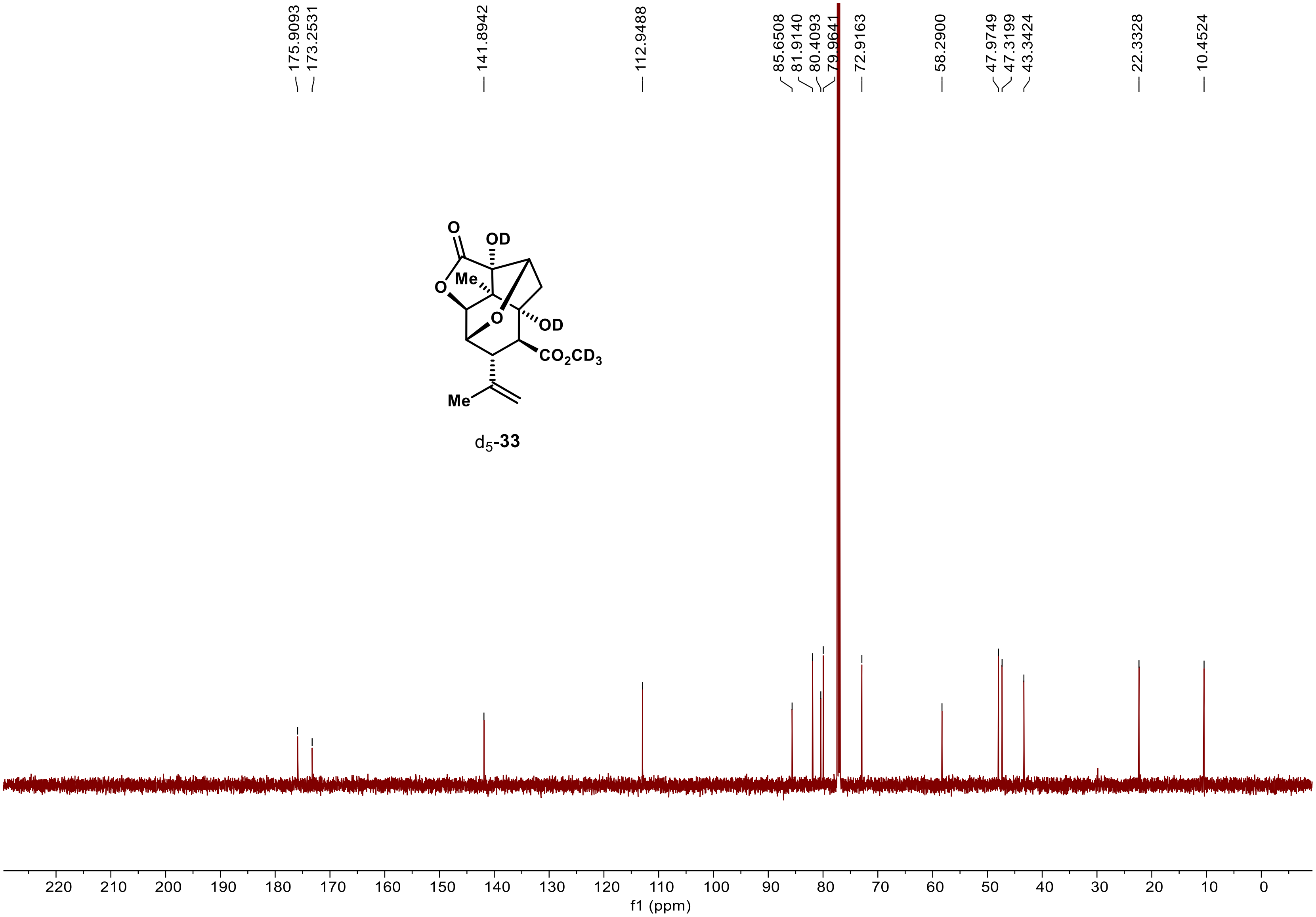

^{13}C NMR, $CDCl_3$, 150 MHz

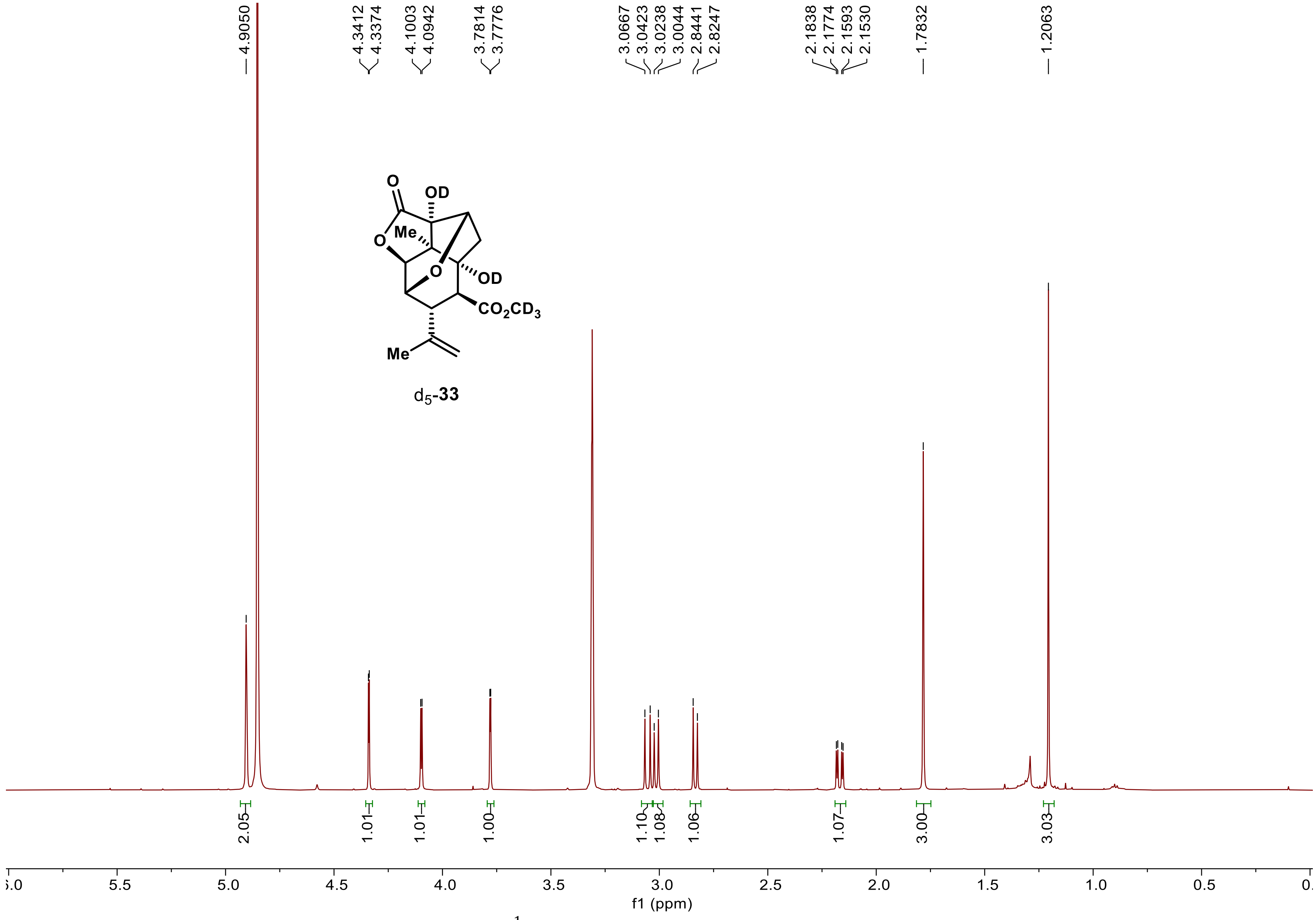

^{1}H NMR, CD_3OD, 600 MHz

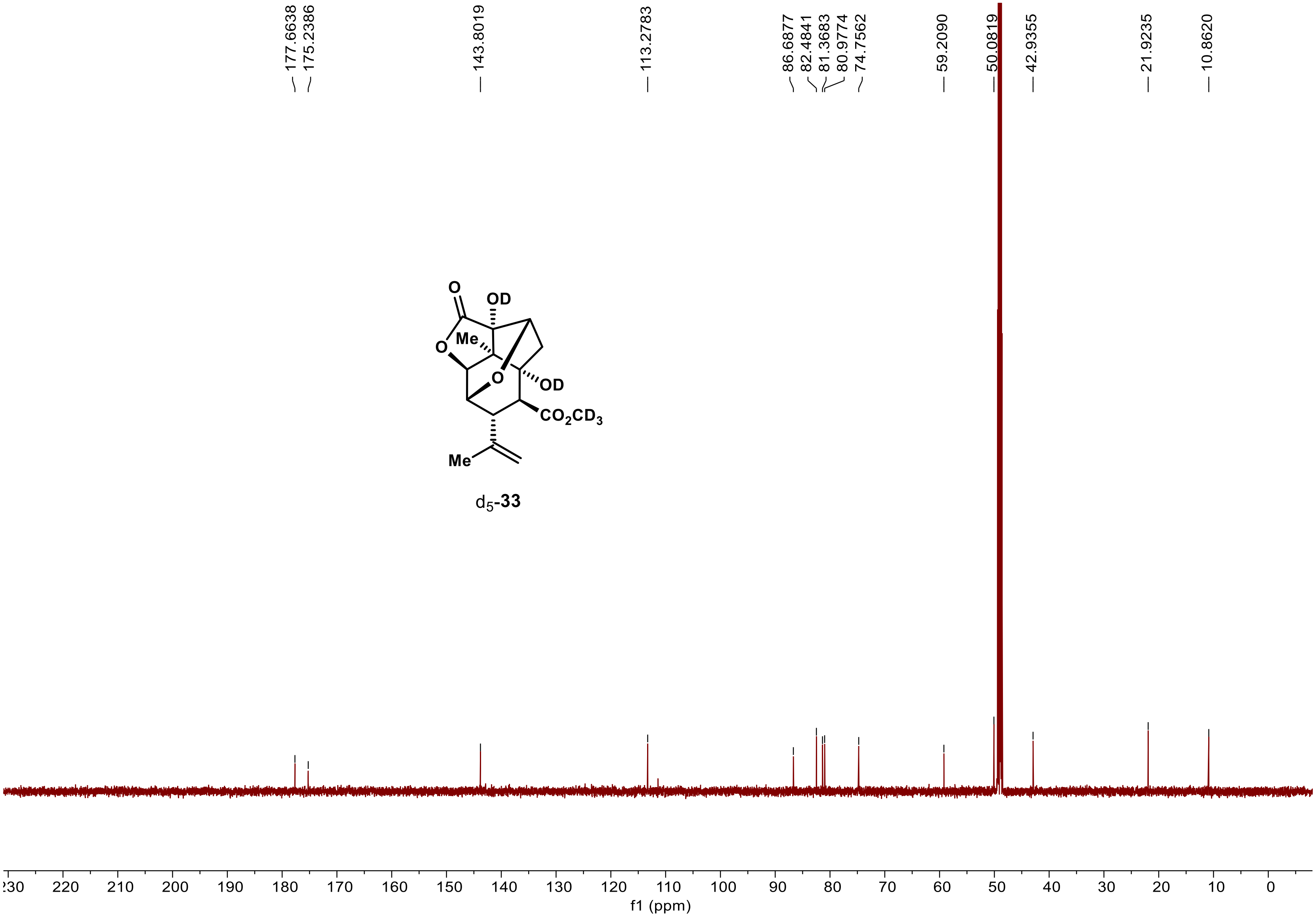

^{13}C NMR, CD_3OD, 150 MHz

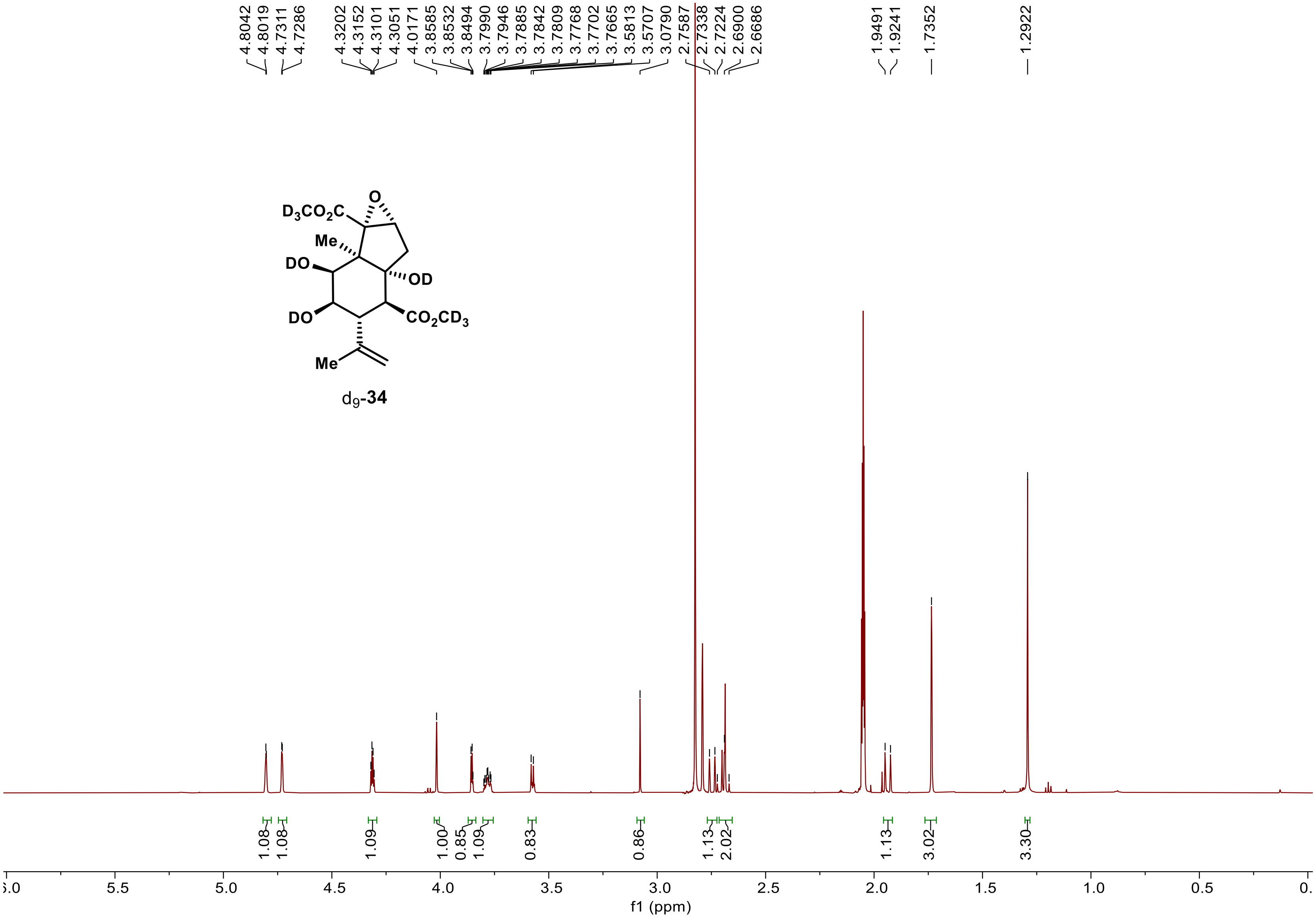

^{1}H NMR, d$_6$-acetone, 600 MHz

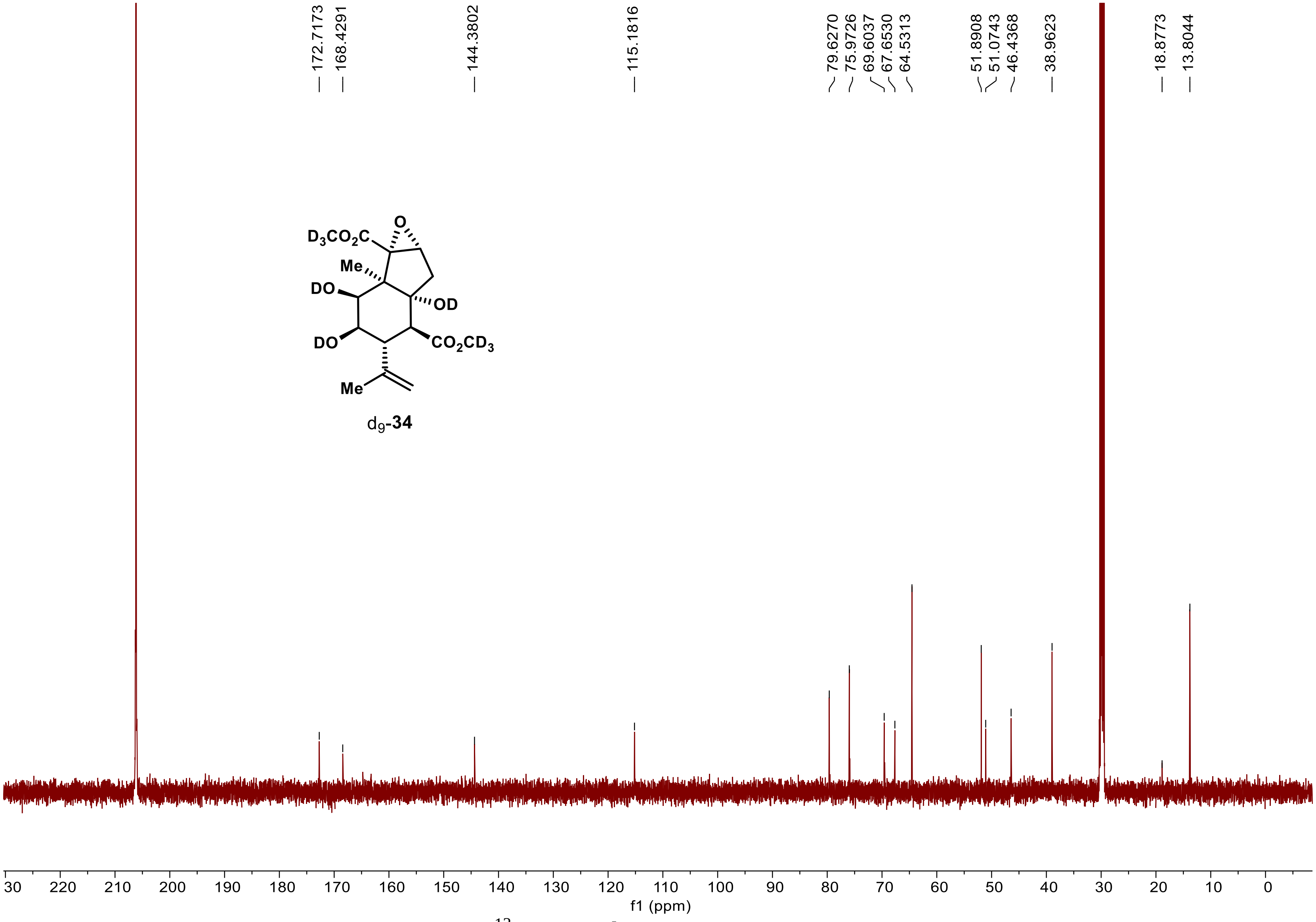

^{13}C NMR, d_6-acetone, 150 MHz

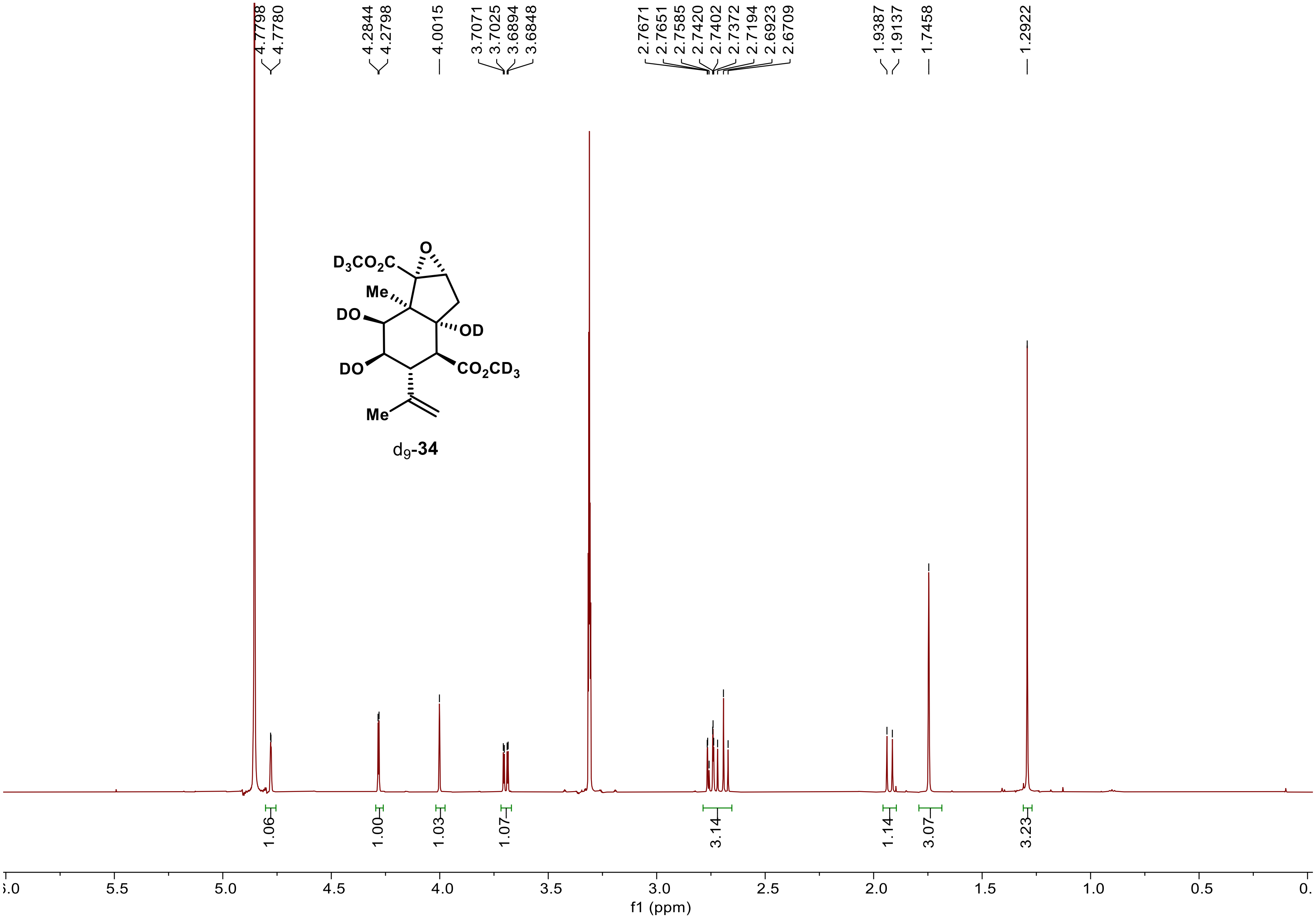

^{1}H NMR, CD_3OD, 600 MHz

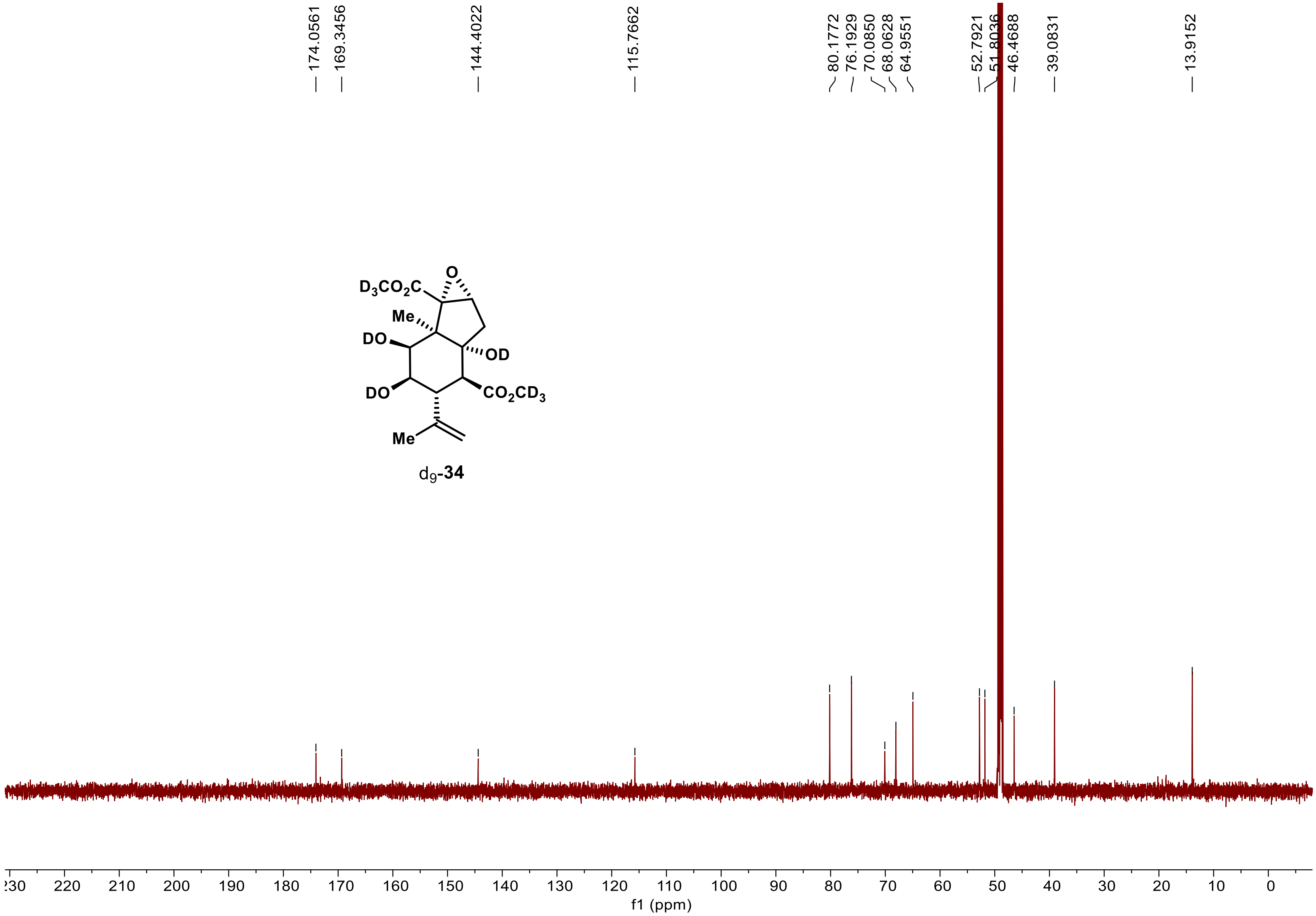

^{13}C NMR, CD_3OD, 150 MHz

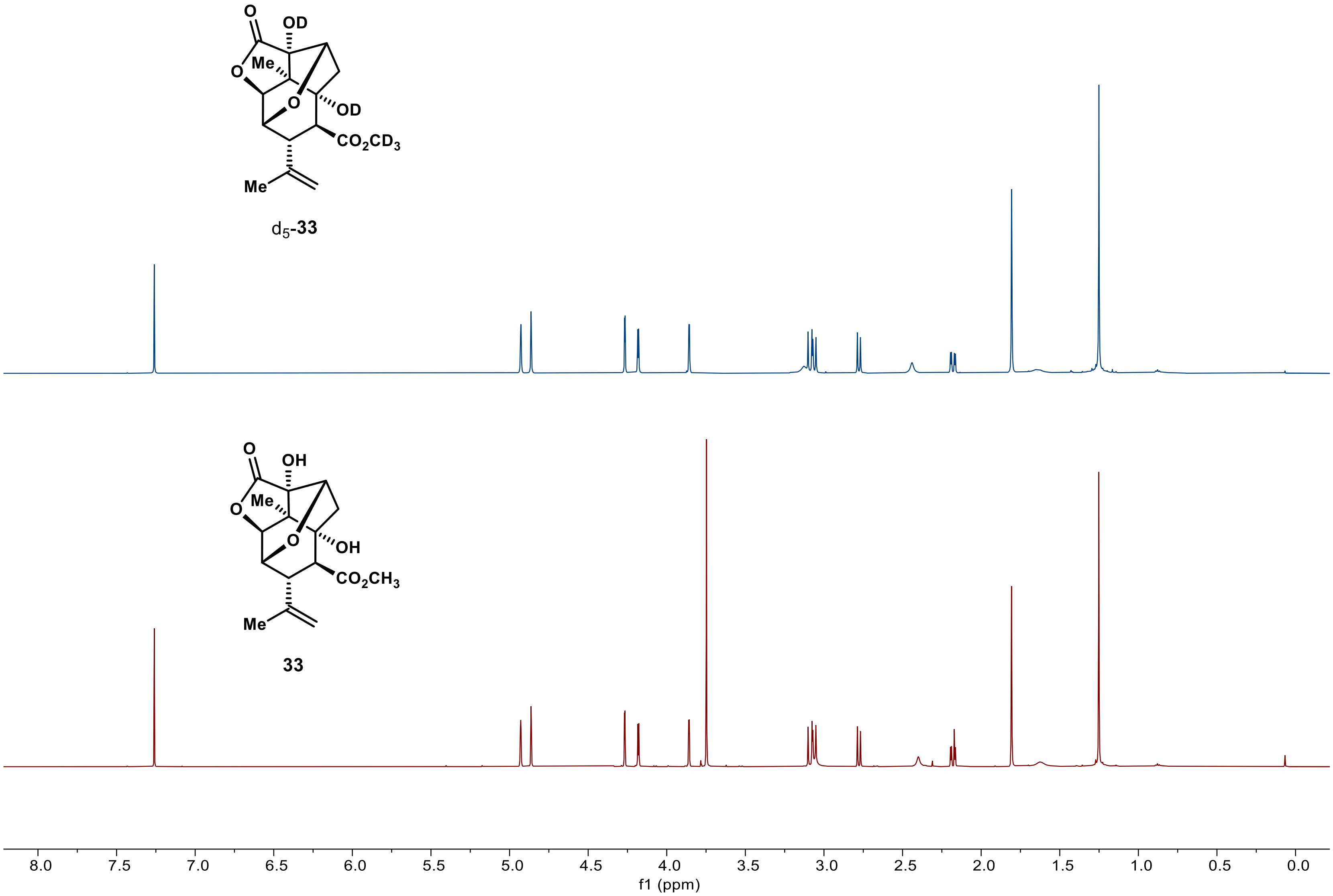

^{1}H NMR comparison of **33** and d$_5$-**33**, $CDCl_3$, 600 MHz

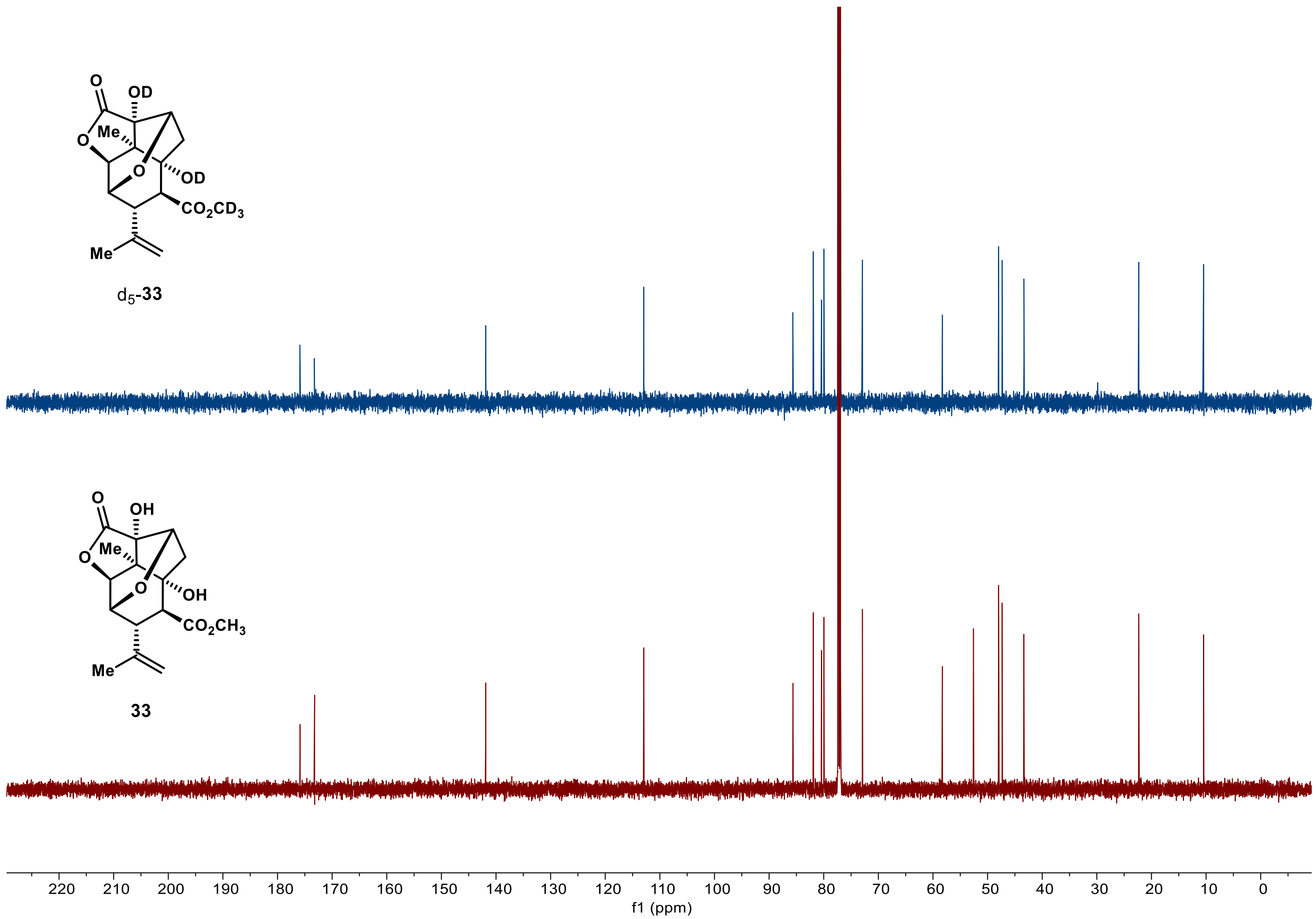

^{13}C NMR comparison of **33** and d$_5$-**33**, $CDCl_3$, 150 MHz

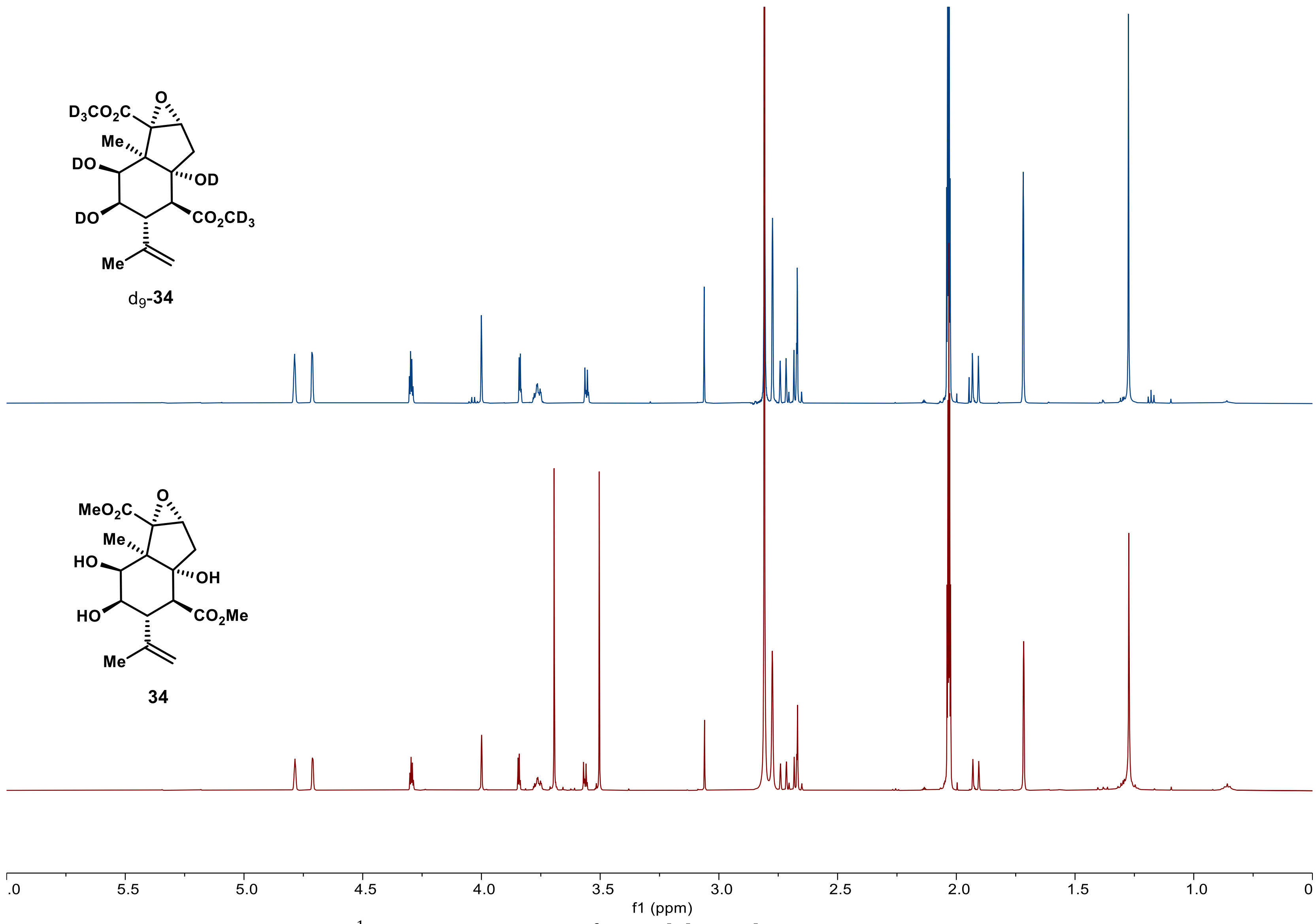

^{1}H NMR comparison of **34** and d$_9$-**34**, d$_6$-acetone, 600 MHz

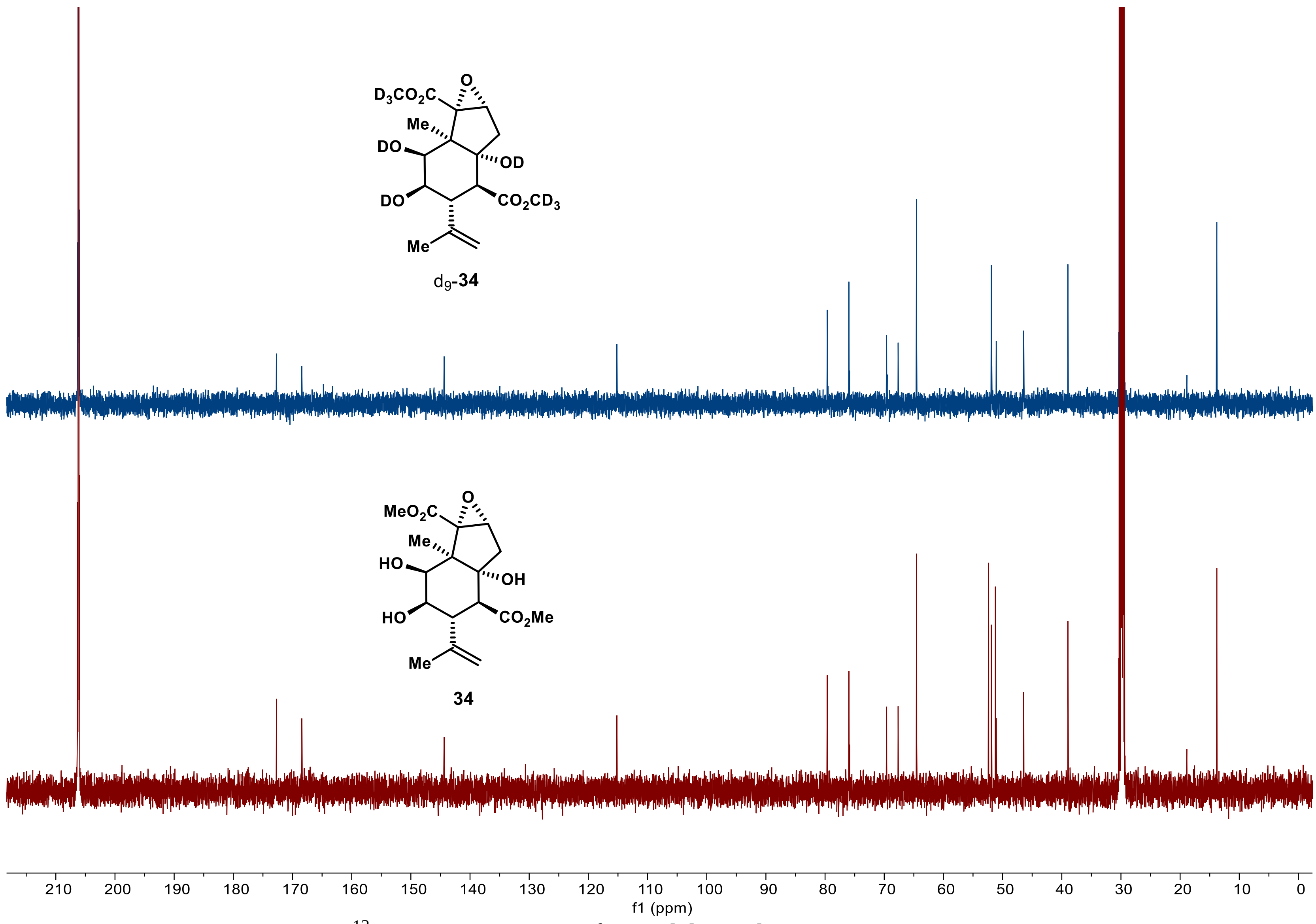

^{13}C NMR comparison of **34** and d$_9$-**34**, d$_6$-acetone, 150 MHz

9. X-ray crystallographic data

5MePTN (11) (CCDC 2048201)

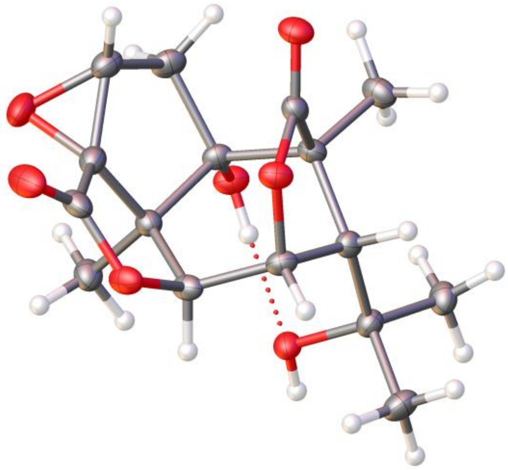

The single crystal X-ray diffraction studies were carried out on a Bruker SMART APEX II CCD diffractometer equipped with Cu K_α radiation (λ = 1.54178). Crystals of the subject compound were used as received. A 0.200 x 0.165 x 0.125 mm colorless block crystal was mounted on a Cryoloop with Paratone oil.

Data were collected in a nitrogen gas stream at 100(2) K using ϕ and ϖ scans. Crystal-to-detector distance was 40 mm using exposure time 1.0, 2.0, and 3.0 second (depending on the 2θ position) with a scan width of 1.50°. Data collection was 99.9 complete to 67.679° in θ. A total of 9230 reflections were collected. 2803 reflections were found to be symmetry independent, with a R_{int} of 0.0260. Indexing and unit cell refinement indicated a **Primitive Monoclinic** lattice. The space group was found to be ***P*2$_1$.** The data were integrated using the Bruker SAINT Software program and scaled using the SADABS software program. Solution by direct methods (SHELXT) produced a complete phasing model consistent with the proposed structure.

All nonhydrogen atoms were refined anisotropically by full-matrix least-squares (SHELXL-2014). All carbon bonded hydrogen atoms were placed using a riding model. Their positions were constrained relative to their parent atom using the appropriate HFIX command in SHELXL-2014.

Supplementary Table 11. Crystal data and structure refinement for Shenvi225.

Report date	2020-12-03	
Identification code	shenvi225	
Empirical formula	C16 H20 O7	
Molecular formula	C16 H20 O7	
Formula weight	324.32	
Temperature	100.0 K	
Wavelength	1.54178 Å	
Crystal system	Monoclinic	
Space group	P 1 21 1	
Unit cell dimensions	a = 8.0573(2) Å	α= 90°.
	b = 11.9123(2) Å	β= 116.6520(10)°.
	c = 8.6986(2) Å	γ = 90°.
Volume	746.19(3) $Å^3$	
Z	2	
Density (calculated)	1.443 Mg/m^3	
Absorption coefficient	0.959 mm^{-1}	
F(000)	344	
Crystal size	0.2 x 0.165 x 0.125 mm^3	
Crystal color, habit	colourless block	
Theta range for data collection	5.691 to 70.963°.	
Index ranges	-9<=h<=9, -14<=k<=14, -10<=l<=10	
Reflections collected	9230	
Independent reflections	2803 [R(int) = 0.0260]	
Completeness to theta = 67.679°	99.9 %	
Absorption correction	Semi-empirical from equivalents	
Max. and min. transmission	0.7534 and 0.6076	
Refinement method	Full-matrix least-squares on F^2	
Data / restraints / parameters	2803 / 1 / 215	
Goodness-of-fit on F^2	1.051	
Final R indices [I>2sigma(I)]	R1 = 0.0244, wR2 = 0.0643	
R indices (all data)	R1 = 0.0245, wR2 = 0.0643	
Absolute structure parameter	0.04(4)	
Extinction coefficient	0.0073(9)	
Largest diff. peak and hole	0.212 and -0.154 e.$Å^{-3}$	

Supplementary Table 12. Atomic coordinates (x 10^4) and equivalent isotropic displacement parameters ($Å^2$x 10^3) for Shenvi225. U(eq) is defined as one third of the trace of the orthogonalized U^{ij} tensor.

	x	y	z	U(eq)
O(1)	6448(2)	5641(1)	7374(2)	20(1)
O(2)	9305(2)	5558(1)	4218(2)	21(1)
O(3)	9794(2)	3784(1)	3697(2)	24(1)
O(4)	6300(2)	6891(1)	2663(2)	24(1)
O(5)	5544(2)	6465(1)	-67(2)	31(1)
O(6)	3743(2)	4438(1)	633(2)	29(1)
O(7)	5486(2)	3663(1)	5618(2)	22(1)
C(1)	9106(3)	4722(2)	9620(2)	24(1)
C(2)	9248(3)	6745(2)	8898(3)	28(1)
C(3)	8450(3)	5586(2)	8170(2)	19(1)
C(4)	9052(2)	5276(2)	6768(2)	18(1)
C(5)	8607(3)	6118(2)	5309(2)	19(1)
C(6)	5352(2)	5228(2)	3644(2)	18(1)
C(7)	6272(3)	4082(1)	4576(2)	18(1)
C(8)	8446(3)	4183(2)	5684(2)	18(1)
C(9)	9260(2)	4431(2)	4440(2)	19(1)
C(10)	6538(3)	6312(2)	4247(2)	19(1)
C(11)	3431(3)	5416(2)	3551(2)	24(1)
C(12)	9289(3)	3101(2)	6669(3)	25(1)
C(13)	5709(3)	6189(2)	1325(3)	23(1)
C(14)	5219(3)	5100(2)	1861(2)	21(1)
C(15)	5562(3)	3930(2)	1523(2)	23(1)
C(16)	5799(3)	3244(2)	3068(3)	22(1)

Supplementary Table 13. Bond lengths [Å] and angles [°] for Shenvi225.

O(1)-H(1)	0.8400	C(11)-H(11C)	0.9800
O(1)-C(3)	1.444(2)	C(12)-H(12A)	0.9800
O(2)-C(5)	1.463(2)	C(12)-H(12B)	0.9800
O(2)-C(9)	1.359(2)	C(12)-H(12C)	0.9800
O(3)-C(9)	1.203(2)	C(13)-C(14)	1.490(3)
O(4)-C(10)	1.474(2)	C(14)-C(15)	1.475(3)
O(4)-C(13)	1.336(3)	C(15)-H(15)	1.0000
O(5)-C(13)	1.203(3)	C(15)-C(16)	1.510(3)
O(6)-C(14)	1.428(2)	C(16)-H(16A)	0.9900
O(6)-C(15)	1.447(2)	C(16)-H(16B)	0.9900
O(7)-H(7)	0.8400		
O(7)-C(7)	1.409(2)	C(3)-O(1)-H(1)	109.5
C(1)-H(1A)	0.9800	C(9)-O(2)-C(5)	108.43(14)
C(1)-H(1B)	0.9800	C(13)-O(4)-C(10)	111.50(14)
C(1)-H(1C)	0.9800	C(14)-O(6)-C(15)	61.74(12)
C(1)-C(3)	1.527(3)	C(7)-O(7)-H(7)	109.5
C(2)-H(2A)	0.9800	H(1A)-C(1)-H(1B)	109.5
C(2)-H(2B)	0.9800	H(1A)-C(1)-H(1C)	109.5
C(2)-H(2C)	0.9800	H(1B)-C(1)-H(1C)	109.5
C(2)-C(3)	1.535(3)	C(3)-C(1)-H(1A)	109.5
C(3)-C(4)	1.545(2)	C(3)-C(1)-H(1B)	109.5
C(4)-H(4)	1.0000	C(3)-C(1)-H(1C)	109.5
C(4)-C(5)	1.529(2)	H(2A)-C(2)-H(2B)	109.5
C(4)-C(8)	1.553(2)	H(2A)-C(2)-H(2C)	109.5
C(5)-H(5)	1.0000	H(2B)-C(2)-H(2C)	109.5
C(5)-C(10)	1.518(3)	C(3)-C(2)-H(2A)	109.5
C(6)-C(7)	1.591(2)	C(3)-C(2)-H(2B)	109.5
C(6)-C(10)	1.550(3)	C(3)-C(2)-H(2C)	109.5
C(6)-C(11)	1.530(2)	O(1)-C(3)-C(1)	108.95(15)
C(6)-C(14)	1.513(3)	O(1)-C(3)-C(2)	109.19(16)
C(7)-C(8)	1.581(3)	O(1)-C(3)-C(4)	108.14(13)
C(7)-C(16)	1.554(2)	C(1)-C(3)-C(2)	109.86(16)
C(8)-C(9)	1.526(3)	C(1)-C(3)-C(4)	112.07(16)
C(8)-C(12)	1.528(2)	C(2)-C(3)-C(4)	108.56(16)
C(10)-H(10)	1.0000	C(3)-C(4)-H(4)	105.1
C(11)-H(11A)	0.9800	C(3)-C(4)-C(8)	123.70(15)
C(11)-H(11B)	0.9800	C(5)-C(4)-C(3)	117.31(15)

C(5)-C(4)-H(4)	105.1	H(11A)-C(11)-H(11B)	109.5
C(5)-C(4)-C(8)	98.82(14)	H(11A)-C(11)-H(11C)	109.5
C(8)-C(4)-H(4)	105.1	H(11B)-C(11)-H(11C)	109.5
O(2)-C(5)-C(4)	102.96(14)	C(8)-C(12)-H(12A)	109.5
O(2)-C(5)-H(5)	110.9	C(8)-C(12)-H(12B)	109.5
O(2)-C(5)-C(10)	108.11(14)	C(8)-C(12)-H(12C)	109.5
C(4)-C(5)-H(5)	110.9	H(12A)-C(12)-H(12B)	109.5
C(10)-C(5)-C(4)	112.91(15)	H(12A)-C(12)-H(12C)	109.5
C(10)-C(5)-H(5)	110.9	H(12B)-C(12)-H(12C)	109.5
C(10)-C(6)-C(7)	117.80(14)	O(4)-C(13)-C(14)	108.28(16)
C(11)-C(6)-C(7)	112.39(16)	O(5)-C(13)-O(4)	122.7(2)
C(11)-C(6)-C(10)	110.59(16)	O(5)-C(13)-C(14)	128.9(2)
C(14)-C(6)-C(7)	103.80(15)	O(6)-C(14)-C(6)	118.36(16)
C(14)-C(6)-C(10)	100.38(15)	O(6)-C(14)-C(13)	119.69(16)
C(14)-C(6)-C(11)	110.85(15)	O(6)-C(14)-C(15)	59.77(13)
O(7)-C(7)-C(6)	113.01(15)	C(13)-C(14)-C(6)	109.01(16)
O(7)-C(7)-C(8)	109.26(14)	C(15)-C(14)-C(6)	111.48(17)
O(7)-C(7)-C(16)	107.73(14)	C(15)-C(14)-C(13)	131.30(17)
C(8)-C(7)-C(6)	112.40(14)	O(6)-C(15)-C(14)	58.48(12)
C(16)-C(7)-C(6)	103.83(14)	O(6)-C(15)-H(15)	120.7
C(16)-C(7)-C(8)	110.39(15)	O(6)-C(15)-C(16)	113.31(16)
C(4)-C(8)-C(7)	113.23(14)	C(14)-C(15)-H(15)	120.7
C(9)-C(8)-C(4)	98.64(14)	C(14)-C(15)-C(16)	107.08(16)
C(9)-C(8)-C(7)	107.09(14)	C(16)-C(15)-H(15)	120.7
C(9)-C(8)-C(12)	109.68(15)	C(7)-C(16)-H(16A)	110.4
C(12)-C(8)-C(4)	115.34(15)	C(7)-C(16)-H(16B)	110.4
C(12)-C(8)-C(7)	111.73(15)	C(15)-C(16)-C(7)	106.81(15)
O(2)-C(9)-C(8)	109.85(15)	C(15)-C(16)-H(16A)	110.4
O(3)-C(9)-O(2)	121.25(17)	C(15)-C(16)-H(16B)	110.4
O(3)-C(9)-C(8)	128.89(17)	H(16A)-C(16)-H(16B)	108.6
O(4)-C(10)-C(5)	106.68(14)		
O(4)-C(10)-C(6)	105.77(15)		
O(4)-C(10)-H(10)	109.8		
C(5)-C(10)-C(6)	114.93(15)		
C(5)-C(10)-H(10)	109.8		
C(6)-C(10)-H(10)	109.8		
C(6)-C(11)-H(11A)	109.5		
C(6)-C(11)-H(11B)	109.5		
C(6)-C(11)-H(11C)	109.5		

Supplementary Table 14. Anisotropic displacement parameters ($Å^2$x 10^3) for Shenvi225. The anisotropic displacement factor exponent takes the form: $-2\pi^2[\ h^2\ a^{*2}U^{11} + ... + 2\ h\ k\ a^*\ b^*\ U^{12}]$

	U^{11}	U^{22}	U^{33}	U^{23}	U^{13}	U^{12}
O(1)	19(1)	24(1)	17(1)	-1(1)	9(1)	2(1)
O(2)	24(1)	18(1)	26(1)	0(1)	16(1)	-1(1)
O(3)	28(1)	22(1)	27(1)	0(1)	18(1)	4(1)
O(4)	35(1)	18(1)	21(1)	5(1)	16(1)	6(1)
O(5)	46(1)	32(1)	22(1)	9(1)	21(1)	13(1)
O(6)	23(1)	38(1)	20(1)	-8(1)	5(1)	-1(1)
O(7)	26(1)	20(1)	26(1)	-2(1)	17(1)	-4(1)
C(1)	23(1)	32(1)	18(1)	2(1)	9(1)	4(1)
C(2)	31(1)	28(1)	24(1)	-9(1)	12(1)	-6(1)
C(3)	19(1)	21(1)	17(1)	-2(1)	7(1)	-1(1)
C(4)	16(1)	19(1)	19(1)	-2(1)	7(1)	-1(1)
C(5)	24(1)	16(1)	20(1)	-2(1)	13(1)	-2(1)
C(6)	18(1)	19(1)	17(1)	0(1)	8(1)	2(1)
C(7)	20(1)	15(1)	20(1)	-2(1)	11(1)	-3(1)
C(8)	20(1)	16(1)	20(1)	0(1)	10(1)	2(1)
C(9)	16(1)	18(1)	21(1)	1(1)	7(1)	1(1)
C(10)	27(1)	16(1)	17(1)	3(1)	13(1)	3(1)
C(11)	19(1)	32(1)	21(1)	0(1)	9(1)	4(1)
C(12)	30(1)	21(1)	26(1)	6(1)	16(1)	7(1)
C(13)	25(1)	25(1)	22(1)	4(1)	13(1)	10(1)
C(14)	19(1)	26(1)	17(1)	-2(1)	7(1)	2(1)
C(15)	22(1)	25(1)	21(1)	-6(1)	10(1)	-3(1)
C(16)	26(1)	19(1)	26(1)	-5(1)	14(1)	-6(1)

Supplementary Table 15. Hydrogen coordinates (x 10^4) and isotropic displacement parameters ($Å^2$x 10^3) for Shenvi225.

	x	y	z	U(eq)
H(1)	6097	5853	8103	30
H(7)	5546	4157	6331	33
H(1A)	8927	5021	10585	37
H(1B)	10426	4562	10004	37
H(1C)	8387	4029	9202	37
H(2A)	8756	7305	7975	41
H(2B)	10605	6723	9381	41
H(2C)	8893	6948	9802	41
H(4)	10439	5241	7382	22
H(5)	9271	6844	5761	22
H(10)	6062	6797	4900	23
H(11A)	2681	4735	3118	36
H(11B)	2817	6044	2775	36
H(11C)	3563	5589	4702	36
H(12A)	10624	3207	7388	37
H(12B)	9093	2490	5850	37
H(12C)	8692	2911	7398	37
H(15)	6317	3763	900	27
H(16A)	6816	2693	3361	27
H(16B)	4640	2833	2827	27

Supplementary Table 16. Hydrogen bonds for Shenvi225 [Å and °].

D-H...A	d(D-H)	d(H...A)	d(D...A)	<(DHA)
O(1)-H(1)...O(5)#1	0.84	1.98	2.8111(19)	173.2
O(7)-H(7)...O(1)	0.84	1.97	2.7243(19)	148.7

Symmetry transformations used to generate equivalent atoms:

#1 x,y,z+1

12F5MePXN (13) (CCDC 2081332)

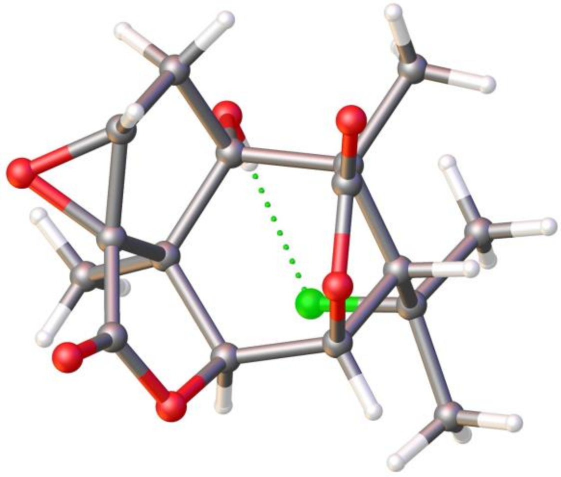

The single crystal X-ray diffraction studies were carried out on a Bruker SMART Pt135 CCD diffractometer equipped with Cu K_α radiation (λ =1.54178 Å).

Crystals of the subject compound were used as received. A 0.35 x 0.25 x 0.21 mm piece of a colorless crystal was mounted on a Cryoloop with Paratone oil. Data were collected in a nitrogen gas stream at 100(2) K using ϕ and ϖ scans. Crystal-to-detector distance was 45 mm and exposure time was 1, 3, 4, 5, 7, 9, or 10 seconds depending on the 2θ range per frame using a scan width of 1.25°. Data collection was 97.9 % complete to 67.679° in θ. A total of 19986 reflections were collected covering the indices, -9<=h<=9, -15<=k<=16, -16<=l<=17. 2736 reflections were found to be symmetry independent, with a R_{int} of 0.0408. Indexing and unit cell refinement indicated a **P**rimitive, **Orthorhombic** lattice. The space group was found to be $\boldsymbol{P2_12_12_1}$. The data were integrated using the Bruker SAINT Software program and scaled using the SADABS software program. Solution by direct methods (SHELXT) produced a complete phasing model consistent with the proposed structure.

All nonhydrogen atoms were refined anisotropically by full-matrix least-squares (SHELXL-2014). All carbon bonded hydrogen atoms were placed using a riding model. Their positions were constrained relative to their parent atom using the appropriate HFIX command in SHELXL-2014.

Supplementary Table 17. Crystal data and structure refinement for Shenvi245.

Identification code	shenvi245_0m_a	
Empirical formula	C16 H19 F O6	
Formula weight	326.31	
Temperature	100.15 K	
Wavelength	1.54178 Å	
Crystal system	Orthorhombic	
Space group	$P2_12_12_1$	
Unit cell dimensions	a = 7.4471(7) Å	α= 90°.
	b = 13.3965(14) Å	β= 90°.
	c = 14.6139(15) Å	γ = 90°.
Volume	1458.0(3) $Å^3$	
Z	4	
Density (calculated)	1.487 Mg/m^3	
Absorption coefficient	1.035 mm^{-1}	
F(000)	688	
Crystal size	0.35 x 0.25 x 0.21 mm^3	
Theta range for data collection	4.477 to 72.050°.	
Index ranges	-9<=h<=9, -15<=k<=16, -16<=l<=17	
Reflections collected	19986	
Independent reflections	2736 [R(int) = 0.0408]	
Completeness to theta = 67.679°	97.9 %	
Absorption correction	Semi-empirical from equivalents	
Max. and min. transmission	0.5220 and 0.3823	
Refinement method	Full-matrix least-squares on F^2	
Data / restraints / parameters	2736 / 0 / 213	
Goodness-of-fit on F^2	1.153	
Final R indices [I>2sigma(I)]	R1 = 0.0280, wR2 = 0.0685	
R indices (all data)	R1 = 0.0288, wR2 = 0.0709	
Absolute structure parameter	0.02(3)	
Largest diff. peak and hole	0.253 and -0.194 e.$Å^{-3}$	

Supplementary Table 18. Atomic coordinates (x 10^4) and equivalent isotropic displacement parameters ($Å^2$x 10^3) for Shenvi245. U(eq) is defined as one third of the trace of the orthogonalized U^{ij} tensor.

	x	y	z	U(eq)
F(1)	1273(2)	6426(1)	4201(1)	18(1)
O(1)	7651(2)	4727(1)	3177(1)	24(1)
C(1)	6110(3)	4982(2)	3198(2)	17(1)
O(2)	4854(2)	4545(1)	2657(1)	17(1)
C(2)	5216(3)	5779(2)	3800(1)	15(1)
O(3)	4028(2)	5952(1)	5366(1)	17(1)
C(3)	6574(3)	6604(2)	4006(2)	20(1)
O(4)	4592(2)	2986(1)	5274(1)	20(1)
C(4)	4552(3)	5237(2)	4701(1)	15(1)
O(5)	3599(2)	1983(1)	3492(1)	22(1)
C(5)	6112(3)	4621(2)	5134(2)	19(1)
O(6)	2086(2)	3402(1)	3230(1)	18(1)
C(6)	5912(3)	3567(2)	4771(2)	18(1)
C(7)	4053(3)	3457(2)	4442(1)	16(1)
C(8)	3293(3)	2839(2)	3684(1)	16(1)
C(9)	2131(3)	4445(2)	3540(1)	14(1)
C(10)	3031(3)	4430(1)	4501(1)	13(1)
C(11)	1588(3)	4381(2)	5244(2)	17(1)
C(12)	3127(3)	5029(2)	2817(1)	15(1)
C(13)	3673(3)	6074(2)	3134(1)	16(1)
C(14)	2159(3)	6794(2)	3389(1)	18(1)
C(15)	740(3)	6823(2)	2650(2)	27(1)
C(16)	2826(3)	7832(2)	3628(2)	26(1)

Supplementary Table 19. Bond lengths [Å] and angles [°] for Shenvi245.

F(1)-C(14)	1.444(2)	C(13)-H(13)	1.0000
O(1)-C(1)	1.198(3)	C(13)-C(14)	1.530(3)
C(1)-O(2)	1.358(3)	C(14)-C(15)	1.512(3)
C(1)-C(2)	1.536(3)	C(14)-C(16)	1.517(3)
O(2)-C(12)	1.459(2)	C(15)-H(15A)	0.9800
C(2)-C(3)	1.527(3)	C(15)-H(15B)	0.9800
C(2)-C(4)	1.582(3)	C(15)-H(15C)	0.9800
C(2)-C(13)	1.557(3)	C(16)-H(16A)	0.9800
O(3)-H(3)	0.8400	C(16)-H(16B)	0.9800
O(3)-C(4)	1.420(2)	C(16)-H(16C)	0.9800
C(3)-H(3A)	0.9800		
C(3)-H(3B)	0.9800	O(1)-C(1)-O(2)	121.5(2)
C(3)-H(3C)	0.9800	O(1)-C(1)-C(2)	128.9(2)
O(4)-C(6)	1.453(3)	O(2)-C(1)-C(2)	109.55(17)
O(4)-C(7)	1.427(2)	C(1)-O(2)-C(12)	108.80(15)
C(4)-C(5)	1.559(3)	C(1)-C(2)-C(4)	106.98(16)
C(4)-C(10)	1.593(3)	C(1)-C(2)-C(13)	97.94(16)
O(5)-C(8)	1.202(3)	C(3)-C(2)-C(1)	109.19(18)
C(5)-H(5A)	0.9900	C(3)-C(2)-C(4)	112.02(17)
C(5)-H(5B)	0.9900	C(3)-C(2)-C(13)	115.39(17)
C(5)-C(6)	1.516(3)	C(13)-C(2)-C(4)	113.96(16)
O(6)-C(8)	1.348(3)	C(4)-O(3)-H(3)	109.5
O(6)-C(9)	1.469(2)	C(2)-C(3)-H(3A)	109.5
C(6)-H(6)	1.0000	C(2)-C(3)-H(3B)	109.5
C(6)-C(7)	1.473(3)	C(2)-C(3)-H(3C)	109.5
C(7)-C(8)	1.494(3)	H(3A)-C(3)-H(3B)	109.5
C(7)-C(10)	1.512(3)	H(3A)-C(3)-H(3C)	109.5
C(9)-H(9)	1.0000	H(3B)-C(3)-H(3C)	109.5
C(9)-C(10)	1.556(3)	C(7)-O(4)-C(6)	61.51(13)
C(9)-C(12)	1.509(3)	C(2)-C(4)-C(10)	112.42(16)
C(10)-C(11)	1.530(3)	O(3)-C(4)-C(2)	110.16(16)
C(11)-H(11A)	0.9800	O(3)-C(4)-C(5)	106.50(16)
C(11)-H(11B)	0.9800	O(3)-C(4)-C(10)	112.86(16)
C(11)-H(11C)	0.9800	C(5)-C(4)-C(2)	110.38(17)
C(12)-H(12)	1.0000	C(5)-C(4)-C(10)	104.19(16)
C(12)-C(13)	1.529(3)	C(4)-C(5)-H(5A)	110.5

C(4)-C(5)-H(5B)	110.5
H(5A)-C(5)-H(5B)	108.7
C(6)-C(5)-C(4)	106.12(17)
C(6)-C(5)-H(5A)	110.5
C(6)-C(5)-H(5B)	110.5
C(8)-O(6)-C(9)	111.43(16)
O(4)-C(6)-C(5)	112.82(18)
O(4)-C(6)-H(6)	120.7
O(4)-C(6)-C(7)	58.37(13)
C(5)-C(6)-H(6)	120.7
C(7)-C(6)-C(5)	107.43(18)
C(7)-C(6)-H(6)	120.7
O(4)-C(7)-C(6)	60.12(14)
O(4)-C(7)-C(8)	119.58(17)
O(4)-C(7)-C(10)	118.30(16)
C(6)-C(7)-C(8)	130.79(19)
C(6)-C(7)-C(10)	111.60(17)
C(8)-C(7)-C(10)	109.22(17)
O(5)-C(8)-O(6)	123.0(2)
O(5)-C(8)-C(7)	129.0(2)
O(6)-C(8)-C(7)	107.91(17)
O(6)-C(9)-H(9)	109.6
O(6)-C(9)-C(10)	105.97(15)
O(6)-C(9)-C(12)	106.76(16)
C(10)-C(9)-H(9)	109.6
C(12)-C(9)-H(9)	109.6
C(12)-C(9)-C(10)	115.26(17)
C(7)-C(10)-C(4)	103.77(16)
C(7)-C(10)-C(9)	100.21(15)
C(7)-C(10)-C(11)	110.94(17)
C(9)-C(10)-C(4)	117.56(16)
C(11)-C(10)-C(4)	113.46(16)
C(11)-C(10)-C(9)	109.79(17)
C(10)-C(11)-H(11A)	109.5
C(10)-C(11)-H(11B)	109.5
C(10)-C(11)-H(11C)	109.5
H(11A)-C(11)-H(11B)	109.5
H(11A)-C(11)-H(11C)	109.5
H(11B)-C(11)-H(11C)	109.5
O(2)-C(12)-C(9)	108.35(16)
O(2)-C(12)-H(12)	110.8
O(2)-C(12)-C(13)	102.77(16)
C(9)-C(12)-H(12)	110.8
C(9)-C(12)-C(13)	113.16(17)
C(13)-C(12)-H(12)	110.8
C(2)-C(13)-H(13)	105.3
C(12)-C(13)-C(2)	98.83(16)
C(12)-C(13)-H(13)	105.3
C(12)-C(13)-C(14)	117.08(18)
C(14)-C(13)-C(2)	123.44(18)
C(14)-C(13)-H(13)	105.3
F(1)-C(14)-C(13)	108.74(16)
F(1)-C(14)-C(15)	106.03(17)
F(1)-C(14)-C(16)	105.89(17)
C(15)-C(14)-C(13)	110.88(18)
C(15)-C(14)-C(16)	111.75(19)
C(16)-C(14)-C(13)	113.13(19)
C(14)-C(15)-H(15A)	109.5
C(14)-C(15)-H(15B)	109.5
C(14)-C(15)-H(15C)	109.5
H(15A)-C(15)-H(15B)	109.5
H(15A)-C(15)-H(15C)	109.5
H(15B)-C(15)-H(15C)	109.5
C(14)-C(16)-H(16A)	109.5
C(14)-C(16)-H(16B)	109.5
C(14)-C(16)-H(16C)	109.5
H(16A)-C(16)-H(16B)	109.5
H(16A)-C(16)-H(16C)	109.5
H(16B)-C(16)-H(16C)	109.5

Symmetry transformations used to generate equivalent atoms:

Supplementary Table 20. Anisotropic displacement parameters ($Å^2$x 10^3) for Shenvi245. The anisotropic displacement factor exponent takes the form: $-2\pi^2$[h^2 $a^{*2}U^{11}$ + ... + 2 h k a* b* U^{12}]

	U^{11}	U^{22}	U^{33}	U^{23}	U^{13}	U^{12}
F(1)	16(1)	20(1)	19(1)	5(1)	4(1)	3(1)
O(1)	15(1)	28(1)	30(1)	-2(1)	4(1)	3(1)
C(1)	15(1)	18(1)	17(1)	3(1)	2(1)	2(1)
O(2)	15(1)	19(1)	16(1)	-3(1)	2(1)	2(1)
C(2)	12(1)	16(1)	17(1)	-1(1)	0(1)	0(1)
O(3)	19(1)	17(1)	16(1)	-3(1)	-1(1)	1(1)
C(3)	16(1)	20(1)	24(1)	-1(1)	0(1)	-4(1)
O(4)	23(1)	18(1)	17(1)	5(1)	-5(1)	1(1)
C(4)	14(1)	16(1)	15(1)	-3(1)	-3(1)	0(1)
O(5)	26(1)	16(1)	22(1)	-3(1)	3(1)	0(1)
C(5)	17(1)	19(1)	20(1)	1(1)	-5(1)	0(1)
O(6)	19(1)	15(1)	19(1)	-2(1)	-3(1)	-2(1)
C(6)	18(1)	20(1)	18(1)	2(1)	-3(1)	2(1)
C(7)	18(1)	15(1)	14(1)	2(1)	-1(1)	1(1)
C(8)	17(1)	17(1)	14(1)	1(1)	3(1)	-1(1)
C(9)	14(1)	13(1)	16(1)	-2(1)	-3(1)	0(1)
C(10)	14(1)	14(1)	12(1)	1(1)	-2(1)	0(1)
C(11)	18(1)	18(1)	16(1)	1(1)	2(1)	0(1)
C(12)	12(1)	18(1)	15(1)	-1(1)	-1(1)	4(1)
C(13)	15(1)	17(1)	15(1)	4(1)	2(1)	0(1)
C(14)	17(1)	18(1)	18(1)	6(1)	5(1)	3(1)
C(15)	22(1)	35(1)	22(1)	10(1)	0(1)	10(1)
C(16)	29(1)	16(1)	34(1)	3(1)	12(1)	4(1)

Supplementary Table 21. Hydrogen coordinates (x 10^4) and isotropic displacement parameters (Å^{2}x 10 ^{3}) for Shenvi245.

	x	y	z	U(eq)
H(3)	3101	6250	5186	26
H(3A)	6747	7016	3459	30
H(3B)	7721	6303	4185	30
H(3C)	6124	7021	4507	30
H(5A)	7289	4903	4955	22
H(5B)	6022	4627	5810	22
H(6)	6928	3231	4446	22
H(9)	877	4705	3595	17
H(11A)	739	3845	5101	26
H(11B)	947	5019	5270	26
H(11C)	2154	4249	5837	26
H(12)	2412	5064	2239	18
H(13)	4282	6385	2595	19
H(15A)	162	6167	2605	40
H(15B)	1301	6990	2063	40
H(15C)	-162	7328	2803	40
H(16A)	1799	8267	3757	39
H(16B)	3511	8104	3112	39
H(16C)	3601	7796	4170	39

CypPXN (14) (CCDC 2071501)

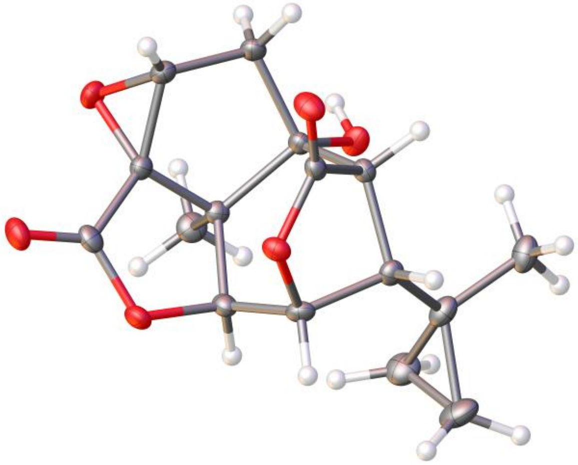

The single crystal X-ray diffraction studies were carried out on a Bruker SMART Pt135 CCD diffractometer equipped with Cu K_α radiation (λ =1.54178 Å).

Crystals of the subject compound were used as received. A 0.25 x 0.22 x 0.19 mm piece of a colorless crystal was mounted on a Cryoloop with Paratone oil. Data were collected in a nitrogen gas stream at 100(2) K using ϕ and ϖ scans. Crystal-to-detector distance was 45 mm and exposure time was 1 or 5 seconds depending on the 2θ range per frame using a scan width of 1.25°. Data collection was 100 % complete to 67.679° in θ. A total of 37096 reflections were collected covering the indices, -13<=h<=14, -16<=k<=16, -31<=l<=27. 8020 reflections were found to be symmetry independent, with a R_{int} of 0.0599. Indexing and unit cell refinement indicated a **P**rimitive, **Orthorhombic** lattice. The space group was found to be $\boldsymbol{P2_12_12_1}$. The data were integrated using the Bruker SAINT Software program and scaled using the SADABS software program. Solution by direct methods (SHELXT) produced a complete phasing model consistent with the proposed structure.

All nonhydrogen atoms were refined anisotropically by full-matrix least-squares (SHELXL-2014). All carbon bonded hydrogen atoms were placed using a riding model. Their positions were constrained relative to their parent atom using the appropriate HFIX command in SHELXL-2014.

Supplementary Table 22. Crystal data and structure refinement for Shenvi240.

Identification code	shenvi240b_0m_a	
Empirical formula	C16 H18 O6	
Formula weight	306.30	
Temperature	100.15 K	
Wavelength	1.54178 Å	
Crystal system	Orthorhombic	
Space group	$P2_12_12_1$	
Unit cell dimensions	a = 11.8989(4) Å	α= 90°.
	b = 13.7406(5) Å	β= 90°.
	c = 25.8524(10) Å	γ = 90°.
Volume	4226.8(3) $Å^3$	
Z	12	
Density (calculated)	1.444 Mg/m^3	
Absorption coefficient	0.930 mm^{-1}	
F(000)	1944	
Crystal size	0.25 x 0.22 x 0.19 mm^3	
Theta range for data collection	3.419 to 70.297°.	
Index ranges	-13<=h<=14, -16<=k<=16, -31<=l<=27	
Reflections collected	37096	
Independent reflections	8020 [R(int) = 0.0599]	
Completeness to theta = 67.679°	100.0 %	
Absorption correction	Semi-empirical from equivalents	
Max. and min. transmission	0.5220 and 0.4163	
Refinement method	Full-matrix least-squares on F^2	
Data / restraints / parameters	8020 / 0 / 604	
Goodness-of-fit on F^2	1.033	
Final R indices [I>2sigma(I)]	R1 = 0.0326, wR2 = 0.0838	
R indices (all data)	R1 = 0.0332, wR2 = 0.0844	
Absolute structure parameter	0.02(4)	
Largest diff. peak and hole	0.324 and -0.200 e.$Å^{-3}$	

Supplementary Table 23. Atomic coordinates (x 10^4) and equivalent isotropic displacement parameters (Å^{2}x 10^3) for Shenvi240. U(eq) is defined as one third of the trace of the orthogonalized U^{ij} tensor.

	x	y	z	U(eq)
O(1B)	6980(1)	8080(1)	4987(1)	17(1)
C(1B)	8093(2)	4975(2)	5465(1)	22(1)
O(2B)	5312(1)	8553(1)	5291(1)	22(1)
C(2B)	8047(2)	4902(2)	4883(1)	22(1)
O(3B)	6651(1)	6102(1)	6296(1)	17(1)
C(3B)	7080(2)	5340(2)	5182(1)	16(1)
O(4B)	7779(1)	8826(1)	6591(1)	21(1)
C(4B)	6043(2)	4711(2)	5234(1)	24(1)
O(5B)	8903(2)	9796(1)	5676(1)	30(1)
C(5B)	6776(2)	6408(2)	5104(1)	13(1)
O(6B)	9051(1)	8265(1)	5378(1)	18(1)
C(6B)	7666(2)	7191(1)	5010(1)	15(1)
C(7B)	6031(2)	7936(2)	5268(1)	16(1)
C(8B)	6089(2)	6946(2)	5519(1)	13(1)
C(9B)	6689(2)	7024(2)	6054(1)	13(1)
C(10B)	7951(2)	7370(2)	6002(1)	12(1)
C(11B)	8728(2)	6901(2)	6403(1)	18(1)
C(12B)	8484(2)	7322(2)	5454(1)	14(1)
C(13B)	6112(2)	7831(2)	6382(1)	17(1)
C(14B)	6768(2)	8756(2)	6279(1)	18(1)
C(15B)	7872(2)	8460(2)	6078(1)	15(1)
C(16B)	8631(2)	8953(2)	5700(1)	18(1)
O(1)	3271(1)	-2374(1)	6594(1)	18(1)
C(1)	2826(3)	-2378(2)	8381(1)	36(1)
O(2)	1704(2)	-2377(1)	6118(1)	22(1)
C(2)	3472(3)	-3310(2)	8265(1)	41(1)
O(3)	718(1)	-1451(1)	7697(1)	20(1)
C(3)	2431(2)	-3061(2)	7964(1)	21(1)
O(4)	1876(1)	620(1)	6705(1)	21(1)
C(4)	1385(2)	-3634(2)	8103(1)	31(1)
O(5)	4217(2)	263(1)	6293(1)	28(1)

C(5)	2546(2)	-2910(2)	7383(1)	14(1)
O(6)	4342(1)	-782(1)	6956(1)	20(1)
C(6)	3535(2)	-2356(2)	7148(1)	16(1)
C(7)	2137(2)	-2401(2)	6537(1)	15(1)
C(8)	1600(2)	-2418(2)	7070(1)	13(1)
C(9)	1336(2)	-1360(2)	7236(1)	13(1)
C(10)	2448(2)	-735(2)	7296(1)	13(1)
C(11)	2371(2)	0(2)	7736(1)	21(1)
C(12)	3589(2)	-1291(2)	7313(1)	16(1)
C(13)	665(2)	-823(2)	6804(1)	19(1)
C(14)	1525(2)	-299(2)	6476(1)	18(1)
C(15)	2561(2)	-221(1)	6784(1)	16(1)
C(16)	3770(2)	-200(2)	6626(1)	18(1)
O(1A)	1757(1)	5446(1)	6303(1)	16(1)
C(1A)	2066(2)	3284(2)	4930(1)	26(1)
O(2A)	700(1)	5038(1)	6978(1)	20(1)
C(2A)	1275(2)	4046(2)	4722(1)	30(1)
O(3A)	1747(1)	2303(1)	6135(1)	18(1)
C(3A)	1067(2)	3605(2)	5249(1)	19(1)
O(4A)	4052(1)	3428(1)	7140(1)	20(1)
C(4A)	94(2)	2896(2)	5276(1)	23(1)
O(5A)	4752(1)	5495(1)	6905(1)	22(1)
C(5A)	1106(2)	4289(2)	5711(1)	15(1)
O(6A)	3957(1)	5214(1)	6134(1)	18(1)
C(6A)	2078(2)	4998(2)	5807(1)	16(1)
C(7A)	1112(2)	4814(2)	6566(1)	14(1)
C(8A)	1022(2)	3870(2)	6265(1)	14(1)
C(9A)	1993(2)	3166(2)	6406(1)	13(1)
C(10A)	3187(2)	3597(2)	6255(1)	13(1)
C(11A)	3984(2)	2810(2)	6060(1)	20(1)
C(12A)	3204(2)	4500(2)	5891(1)	15(1)
C(13A)	2055(2)	3022(2)	7005(1)	16(1)
C(14A)	2900(2)	3761(2)	7200(1)	16(1)
C(15A)	3594(2)	4052(2)	6755(1)	14(1)
C(16A)	4158(2)	4996(2)	6633(1)	16(1)

Supplementary Table 24. Bond lengths [Å] and angles [°] for Shenvi240.

O(1B)-C(6B)	1.471(3)	C(10B)-C(15B)	1.513(3)
O(1B)-C(7B)	1.358(3)	C(11B)-H(11A)	0.9800
C(1B)-H(1BA)	0.9900	C(11B)-H(11B)	0.9800
C(1B)-H(1BB)	0.9900	C(11B)-H(11C)	0.9800
C(1B)-C(2B)	1.508(3)	C(12B)-H(12B)	1.0000
C(1B)-C(3B)	1.496(3)	C(13B)-H(13A)	0.9900
O(2B)-C(7B)	1.206(3)	C(13B)-H(13B)	0.9900
C(2B)-H(2BA)	0.9900	C(13B)-C(14B)	1.515(3)
C(2B)-H(2BB)	0.9900	C(14B)-H(14B)	1.0000
C(2B)-C(3B)	1.511(3)	C(14B)-C(15B)	1.470(3)
O(3B)-H(3B)	0.8400	C(15B)-C(16B)	1.493(3)
O(3B)-C(9B)	1.413(2)	O(1)-C(6)	1.467(3)
C(3B)-C(4B)	1.512(3)	O(1)-C(7)	1.358(3)
C(3B)-C(5B)	1.525(3)	C(1)-H(1A)	0.9900
O(4B)-C(14B)	1.452(3)	C(1)-H(1B)	0.9900
O(4B)-C(15B)	1.423(3)	C(1)-C(2)	1.524(5)
C(4B)-H(4BA)	0.9800	C(1)-C(3)	1.505(3)
C(4B)-H(4BB)	0.9800	O(2)-C(7)	1.200(3)
C(4B)-H(4BC)	0.9800	C(2)-H(2A)	0.9900
O(5B)-C(16B)	1.203(3)	C(2)-H(2B)	0.9900
C(5B)-H(5B)	1.0000	C(2)-C(3)	1.502(4)
C(5B)-C(6B)	1.528(3)	O(3)-H(3)	0.8400
C(5B)-C(8B)	1.538(3)	O(3)-C(9)	1.406(3)
O(6B)-C(12B)	1.473(3)	C(3)-C(4)	1.516(3)
O(6B)-C(16B)	1.356(3)	C(3)-C(5)	1.521(3)
C(6B)-H(6B)	1.0000	O(4)-C(14)	1.455(3)
C(6B)-C(12B)	1.515(3)	O(4)-C(15)	1.429(3)
C(7B)-C(8B)	1.508(3)	C(4)-H(4A)	0.9800
C(8B)-H(8B)	1.0000	C(4)-H(4B)	0.9800
C(8B)-C(9B)	1.559(3)	C(4)-H(4C)	0.9800
C(9B)-C(10B)	1.581(3)	O(5)-C(16)	1.195(3)
C(9B)-C(13B)	1.557(3)	C(5)-H(5)	1.0000
C(10B)-C(11B)	1.530(3)	C(5)-C(6)	1.529(3)
C(10B)-C(12B)	1.554(3)	C(5)-C(8)	1.544(3)

O(6)-C(12)	1.465(3)	O(4A)-C(14A)	1.453(3)
O(6)-C(16)	1.352(3)	O(4A)-C(15A)	1.423(3)
C(6)-H(6)	1.0000	C(4A)-H(4AA)	0.9800
C(6)-C(12)	1.526(3)	C(4A)-H(4AB)	0.9800
C(7)-C(8)	1.519(3)	C(4A)-H(4AC)	0.9800
C(8)-H(8)	1.0000	O(5A)-C(16A)	1.210(3)
C(8)-C(9)	1.548(3)	C(5A)-H(5A)	1.0000
C(9)-C(10)	1.584(3)	C(5A)-C(6A)	1.532(3)
C(9)-C(13)	1.559(3)	C(5A)-C(8A)	1.547(3)
C(10)-C(11)	1.524(3)	O(6A)-C(12A)	1.470(3)
C(10)-C(12)	1.558(3)	O(6A)-C(16A)	1.344(3)
C(10)-C(15)	1.508(3)	C(6A)-H(6A)	1.0000
C(11)-H(11G)	0.9800	C(6A)-C(12A)	1.520(3)
C(11)-H(11H)	0.9800	C(7A)-C(8A)	1.518(3)
C(11)-H(11I)	0.9800	C(8A)-H(8A)	1.0000
C(12)-H(12)	1.0000	C(8A)-C(9A)	1.550(3)
C(13)-H(13E)	0.9900	C(9A)-C(10A)	1.588(3)
C(13)-H(13F)	0.9900	C(9A)-C(13A)	1.562(3)
C(13)-C(14)	1.511(3)	C(10A)-C(11A)	1.525(3)
C(14)-H(14)	1.0000	C(10A)-C(12A)	1.557(3)
C(14)-C(15)	1.472(3)	C(10A)-C(15A)	1.515(3)
C(15)-C(16)	1.496(3)	C(11A)-H(11D)	0.9800
O(1A)-C(6A)	1.474(2)	C(11A)-H(11E)	0.9800
O(1A)-C(7A)	1.344(3)	C(11A)-H(11F)	0.9800
C(1A)-H(1AA)	0.9900	C(12A)-H(12A)	1.0000
C(1A)-H(1AB)	0.9900	C(13A)-H(13C)	0.9900
C(1A)-C(2A)	1.507(4)	C(13A)-H(13D)	0.9900
C(1A)-C(3A)	1.513(3)	C(13A)-C(14A)	1.516(3)
O(2A)-C(7A)	1.210(3)	C(14A)-H(14A)	1.0000
C(2A)-H(2AA)	0.9900	C(14A)-C(15A)	1.473(3)
C(2A)-H(2AB)	0.9900	C(15A)-C(16A)	1.493(3)
C(2A)-C(3A)	1.512(3)		
O(3A)-H(3A)	0.8400	C(7B)-O(1B)-C(6B)	108.51(15)
O(3A)-C(9A)	1.409(2)	H(1BA)-C(1B)-H(1BB)	114.9
C(3A)-C(4A)	1.515(3)	C(2B)-C(1B)-H(1BA)	117.7
C(3A)-C(5A)	1.520(3)	C(2B)-C(1B)-H(1BB)	117.7

C(3B)-C(1B)-H(1BA)	117.7	O(1B)-C(7B)-C(8B)	108.93(17)
C(3B)-C(1B)-H(1BB)	117.7	O(2B)-C(7B)-O(1B)	120.9(2)
C(3B)-C(1B)-C(2B)	60.40(15)	O(2B)-C(7B)-C(8B)	130.1(2)
C(1B)-C(2B)-H(2BA)	117.8	C(5B)-C(8B)-H(8B)	111.1
C(1B)-C(2B)-H(2BB)	117.8	C(5B)-C(8B)-C(9B)	114.07(16)
C(1B)-C(2B)-C(3B)	59.41(15)	C(7B)-C(8B)-C(5B)	99.05(16)
H(2BA)-C(2B)-H(2BB)	115.0	C(7B)-C(8B)-H(8B)	111.1
C(3B)-C(2B)-H(2BA)	117.8	C(7B)-C(8B)-C(9B)	109.93(16)
C(3B)-C(2B)-H(2BB)	117.8	C(9B)-C(8B)-H(8B)	111.1
C(9B)-O(3B)-H(3B)	109.5	O(3B)-C(9B)-C(8B)	108.47(16)
C(1B)-C(3B)-C(2B)	60.20(15)	O(3B)-C(9B)-C(10B)	109.72(16)
C(1B)-C(3B)-C(4B)	114.96(19)	O(3B)-C(9B)-C(13B)	112.50(16)
C(1B)-C(3B)-C(5B)	125.38(19)	C(8B)-C(9B)-C(10B)	112.38(16)
C(2B)-C(3B)-C(4B)	115.96(18)	C(13B)-C(9B)-C(8B)	109.29(16)
C(2B)-C(3B)-C(5B)	119.77(18)	C(13B)-C(9B)-C(10B)	104.50(16)
C(4B)-C(3B)-C(5B)	111.62(18)	C(11B)-C(10B)-C(9B)	112.97(16)
C(15B)-O(4B)-C(14B)	61.51(14)	C(11B)-C(10B)-C(12B)	110.65(17)
C(3B)-C(4B)-H(4BA)	109.5	C(12B)-C(10B)-C(9B)	116.87(16)
C(3B)-C(4B)-H(4BB)	109.5	C(15B)-C(10B)-C(9B)	103.18(17)
C(3B)-C(4B)-H(4BC)	109.5	C(15B)-C(10B)-C(11B)	111.52(17)
H(4BA)-C(4B)-H(4BB)	109.5	C(15B)-C(10B)-C(12B)	100.63(16)
H(4BA)-C(4B)-H(4BC)	109.5	C(10B)-C(11B)-H(11A)	109.5
H(4BB)-C(4B)-H(4BC)	109.5	C(10B)-C(11B)-H(11B)	109.5
C(3B)-C(5B)-H(5B)	105.0	C(10B)-C(11B)-H(11C)	109.5
C(3B)-C(5B)-C(6B)	122.26(18)	H(11A)-C(11B)-H(11B)	109.5
C(3B)-C(5B)-C(8B)	119.75(17)	H(11A)-C(11B)-H(11C)	109.5
C(6B)-C(5B)-H(5B)	105.0	H(11B)-C(11B)-H(11C)	109.5
C(6B)-C(5B)-C(8B)	98.08(16)	O(6B)-C(12B)-C(6B)	107.33(16)
C(8B)-C(5B)-H(5B)	105.0	O(6B)-C(12B)-C(10B)	105.74(16)
C(16B)-O(6B)-C(12B)	111.28(16)	O(6B)-C(12B)-H(12B)	109.3
O(1B)-C(6B)-C(5B)	101.94(16)	C(6B)-C(12B)-C(10B)	115.65(18)
O(1B)-C(6B)-H(6B)	111.2	C(6B)-C(12B)-H(12B)	109.3
O(1B)-C(6B)-C(12B)	106.82(16)	C(10B)-C(12B)-H(12B)	109.3
C(5B)-C(6B)-H(6B)	111.2	C(9B)-C(13B)-H(13A)	110.6
C(12B)-C(6B)-C(5B)	114.14(17)	C(9B)-C(13B)-H(13B)	110.6
C(12B)-C(6B)-H(6B)	111.2	H(13A)-C(13B)-H(13B)	108.7

C(14B)-C(13B)-C(9B)	105.92(17)	C(2)-C(3)-C(5)	117.9(2)
C(14B)-C(13B)-H(13A)	110.6	C(4)-C(3)-C(5)	112.28(19)
C(14B)-C(13B)-H(13B)	110.6	C(15)-O(4)-C(14)	61.39(14)
O(4B)-C(14B)-C(13B)	112.58(18)	C(3)-C(4)-H(4A)	109.5
O(4B)-C(14B)-H(14B)	121.0	C(3)-C(4)-H(4B)	109.5
O(4B)-C(14B)-C(15B)	58.27(13)	C(3)-C(4)-H(4C)	109.5
C(13B)-C(14B)-H(14B)	121.0	H(4A)-C(4)-H(4B)	109.5
C(15B)-C(14B)-C(13B)	106.87(18)	H(4A)-C(4)-H(4C)	109.5
C(15B)-C(14B)-H(14B)	121.0	H(4B)-C(4)-H(4C)	109.5
O(4B)-C(15B)-C(10B)	118.36(18)	C(3)-C(5)-H(5)	104.8
O(4B)-C(15B)-C(14B)	60.22(13)	C(3)-C(5)-C(6)	122.01(19)
O(4B)-C(15B)-C(16B)	119.71(18)	C(3)-C(5)-C(8)	120.88(18)
C(14B)-C(15B)-C(10B)	112.02(18)	C(6)-C(5)-H(5)	104.8
C(14B)-C(15B)-C(16B)	130.2(2)	C(6)-C(5)-C(8)	97.72(16)
C(16B)-C(15B)-C(10B)	109.15(18)	C(8)-C(5)-H(5)	104.8
O(5B)-C(16B)-O(6B)	122.7(2)	C(16)-O(6)-C(12)	111.86(17)
O(5B)-C(16B)-C(15B)	129.3(2)	O(1)-C(6)-C(5)	102.45(16)
O(6B)-C(16B)-C(15B)	107.90(18)	O(1)-C(6)-H(6)	111.1
C(7)-O(1)-C(6)	108.59(16)	O(1)-C(6)-C(12)	107.41(16)
H(1A)-C(1)-H(1B)	115.0	C(5)-C(6)-H(6)	111.1
C(2)-C(1)-H(1A)	117.8	C(12)-C(6)-C(5)	113.45(17)
C(2)-C(1)-H(1B)	117.8	C(12)-C(6)-H(6)	111.1
C(3)-C(1)-H(1A)	117.8	O(1)-C(7)-C(8)	108.72(17)
C(3)-C(1)-H(1B)	117.8	O(2)-C(7)-O(1)	121.6(2)
C(3)-C(1)-C(2)	59.45(18)	O(2)-C(7)-C(8)	129.7(2)
C(1)-C(2)-H(2A)	117.8	C(5)-C(8)-H(8)	111.0
C(1)-C(2)-H(2B)	117.8	C(5)-C(8)-C(9)	114.38(17)
H(2A)-C(2)-H(2B)	114.9	C(7)-C(8)-C(5)	100.14(16)
C(3)-C(2)-C(1)	59.67(18)	C(7)-C(8)-H(8)	111.0
C(3)-C(2)-H(2A)	117.8	C(7)-C(8)-C(9)	108.85(16)
C(3)-C(2)-H(2B)	117.8	C(9)-C(8)-H(8)	111.0
C(9)-O(3)-H(3)	109.5	O(3)-C(9)-C(8)	104.92(16)
C(1)-C(3)-C(4)	114.2(2)	O(3)-C(9)-C(10)	113.70(17)
C(1)-C(3)-C(5)	126.4(2)	O(3)-C(9)-C(13)	112.41(17)
C(2)-C(3)-C(1)	60.9(2)	C(8)-C(9)-C(10)	111.54(16)
C(2)-C(3)-C(4)	115.8(2)	C(8)-C(9)-C(13)	110.42(17)

C(13)-C(9)-C(10)	104.01(17)
C(11)-C(10)-C(9)	112.51(18)
C(11)-C(10)-C(12)	110.86(18)
C(12)-C(10)-C(9)	117.70(16)
C(15)-C(10)-C(9)	103.99(17)
C(15)-C(10)-C(11)	110.52(17)
C(15)-C(10)-C(12)	100.15(17)
C(10)-C(11)-H(11G)	109.5
C(10)-C(11)-H(11H)	109.5
C(10)-C(11)-H(11I)	109.5
H(11G)-C(11)-H(11H)	109.5
H(11G)-C(11)-H(11I)	109.5
H(11H)-C(11)-H(11I)	109.5
O(6)-C(12)-C(6)	107.80(17)
O(6)-C(12)-C(10)	106.37(16)
O(6)-C(12)-H(12)	109.1
C(6)-C(12)-C(10)	115.20(17)
C(6)-C(12)-H(12)	109.1
C(10)-C(12)-H(12)	109.1
C(9)-C(13)-H(13E)	110.5
C(9)-C(13)-H(13F)	110.5
H(13E)-C(13)-H(13F)	108.7
C(14)-C(13)-C(9)	106.33(18)
C(14)-C(13)-H(13E)	110.5
C(14)-C(13)-H(13F)	110.5
O(4)-C(14)-C(13)	112.27(19)
O(4)-C(14)-H(14)	120.9
O(4)-C(14)-C(15)	58.41(13)
C(13)-C(14)-H(14)	120.9
C(15)-C(14)-C(13)	107.33(18)
C(15)-C(14)-H(14)	120.9
O(4)-C(15)-C(10)	116.99(18)
O(4)-C(15)-C(14)	60.20(13)
O(4)-C(15)-C(16)	119.67(18)
C(14)-C(15)-C(10)	111.49(19)
C(14)-C(15)-C(16)	131.28(19)
C(16)-C(15)-C(10)	109.54(18)
O(5)-C(16)-O(6)	123.0(2)
O(5)-C(16)-C(15)	129.4(2)
O(6)-C(16)-C(15)	107.50(18)
C(7A)-O(1A)-C(6A)	108.60(15)
H(1AA)-C(1A)-H(1AB)	114.9
C(2A)-C(1A)-H(1AA)	117.8
C(2A)-C(1A)-H(1AB)	117.8
C(2A)-C(1A)-C(3A)	60.09(16)
C(3A)-C(1A)-H(1AA)	117.8
C(3A)-C(1A)-H(1AB)	117.8
C(1A)-C(2A)-H(2AA)	117.8
C(1A)-C(2A)-H(2AB)	117.8
C(1A)-C(2A)-C(3A)	60.13(15)
H(2AA)-C(2A)-H(2AB)	114.9
C(3A)-C(2A)-H(2AA)	117.8
C(3A)-C(2A)-H(2AB)	117.8
C(9A)-O(3A)-H(3A)	109.5
C(1A)-C(3A)-C(4A)	115.97(19)
C(1A)-C(3A)-C(5A)	125.7(2)
C(2A)-C(3A)-C(1A)	59.78(17)
C(2A)-C(3A)-C(4A)	115.1(2)
C(2A)-C(3A)-C(5A)	117.08(19)
C(4A)-C(3A)-C(5A)	112.66(19)
C(15A)-O(4A)-C(14A)	61.57(13)
C(3A)-C(4A)-H(4AA)	109.5
C(3A)-C(4A)-H(4AB)	109.5
C(3A)-C(4A)-H(4AC)	109.5
H(4AA)-C(4A)-H(4AB)	109.5
H(4AA)-C(4A)-H(4AC)	109.5
H(4AB)-C(4A)-H(4AC)	109.5
C(3A)-C(5A)-H(5A)	104.9
C(3A)-C(5A)-C(6A)	122.93(19)
C(3A)-C(5A)-C(8A)	119.61(17)
C(6A)-C(5A)-H(5A)	104.9
C(6A)-C(5A)-C(8A)	97.76(16)

C(8A)-C(5A)-H(5A)	104.9	O(6A)-C(12A)-C(10A)	106.29(16)
C(16A)-O(6A)-C(12A)	111.72(16)	O(6A)-C(12A)-H(12A)	109.1
O(1A)-C(6A)-C(5A)	102.18(16)	C(6A)-C(12A)-C(10A)	115.64(17)
O(1A)-C(6A)-H(6A)	111.2	C(6A)-C(12A)-H(12A)	109.1
O(1A)-C(6A)-C(12A)	107.00(16)	C(10A)-C(12A)-H(12A)	109.1
C(5A)-C(6A)-H(6A)	111.2	C(9A)-C(13A)-H(13C)	110.5
C(12A)-C(6A)-C(5A)	113.77(17)	C(9A)-C(13A)-H(13D)	110.5
C(12A)-C(6A)-H(6A)	111.2	H(13C)-C(13A)-H(13D)	108.7
O(1A)-C(7A)-C(8A)	109.41(17)	C(14A)-C(13A)-C(9A)	106.05(16)
O(2A)-C(7A)-O(1A)	120.76(19)	C(14A)-C(13A)-H(13C)	110.5
O(2A)-C(7A)-C(8A)	129.83(19)	C(14A)-C(13A)-H(13D)	110.5
C(5A)-C(8A)-H(8A)	110.9	O(4A)-C(14A)-C(13A)	112.25(18)
C(5A)-C(8A)-C(9A)	113.78(17)	O(4A)-C(14A)-H(14A)	121.0
C(7A)-C(8A)-C(5A)	98.77(16)	O(4A)-C(14A)-C(15A)	58.22(13)
C(7A)-C(8A)-H(8A)	110.9	C(13A)-C(14A)-H(14A)	121.0
C(7A)-C(8A)-C(9A)	111.07(16)	C(15A)-C(14A)-C(13A)	107.10(17)
C(9A)-C(8A)-H(8A)	110.9	C(15A)-C(14A)-H(14A)	121.0
O(3A)-C(9A)-C(8A)	104.62(16)	O(4A)-C(15A)-C(10A)	118.08(17)
O(3A)-C(9A)-C(10A)	112.18(17)	O(4A)-C(15A)-C(14A)	60.21(14)
O(3A)-C(9A)-C(13A)	113.41(16)	O(4A)-C(15A)-C(16A)	120.01(18)
C(8A)-C(9A)-C(10A)	112.03(16)	C(14A)-C(15A)-C(10A)	112.01(18)
C(8A)-C(9A)-C(13A)	110.38(17)	C(14A)-C(15A)-C(16A)	130.79(19)
C(13A)-C(9A)-C(10A)	104.42(16)	C(16A)-C(15A)-C(10A)	108.75(18)
C(11A)-C(10A)-C(9A)	111.90(17)	O(5A)-C(16A)-O(6A)	122.3(2)
C(11A)-C(10A)-C(12A)	110.91(17)	O(5A)-C(16A)-C(15A)	129.1(2)
C(12A)-C(10A)-C(9A)	117.29(17)	O(6A)-C(16A)-C(15A)	108.47(18)
C(15A)-C(10A)-C(9A)	103.33(16)		
C(15A)-C(10A)-C(11A)	112.02(17)		
C(15A)-C(10A)-C(12A)	100.52(16)		
C(10A)-C(11A)-H(11D)	109.5		
C(10A)-C(11A)-H(11E)	109.5		
C(10A)-C(11A)-H(11F)	109.5		
H(11D)-C(11A)-H(11E)	109.5		
H(11D)-C(11A)-H(11F)	109.5		
H(11E)-C(11A)-H(11F)	109.5		
O(6A)-C(12A)-C(6A)	107.33(16)		

Symmetry transformations used to generate equivalent atoms:

Supplementary Table 25. Anisotropic displacement parameters ($Å^2$x 10^3) for Shenvi240. The anisotropic displacement factor exponent takes the form: $-2\pi^2$[h^2 $a^{*2}U^{11}$ + ... + 2 h k a* b* U^{12}]

	U^{11}	U^{22}	U^{33}	U^{23}	U^{13}	U^{12}
O(1B)	23(1)	12(1)	15(1)	3(1)	-2(1)	1(1)
C(1B)	20(1)	17(1)	28(1)	1(1)	-2(1)	4(1)
O(2B)	20(1)	16(1)	29(1)	-4(1)	-11(1)	5(1)
C(2B)	21(1)	17(1)	29(1)	-3(1)	6(1)	1(1)
O(3B)	14(1)	19(1)	18(1)	9(1)	2(1)	-2(1)
C(3B)	16(1)	12(1)	19(1)	-1(1)	0(1)	2(1)
O(4B)	19(1)	28(1)	15(1)	-6(1)	1(1)	-3(1)
C(4B)	21(1)	16(1)	34(1)	-1(1)	1(1)	-1(1)
O(5B)	36(1)	20(1)	34(1)	-3(1)	12(1)	-11(1)
C(5B)	13(1)	13(1)	14(1)	1(1)	-1(1)	1(1)
O(6B)	18(1)	18(1)	18(1)	0(1)	6(1)	-7(1)
C(6B)	18(1)	12(1)	15(1)	1(1)	2(1)	1(1)
C(7B)	17(1)	15(1)	15(1)	-2(1)	-7(1)	1(1)
C(8B)	9(1)	13(1)	16(1)	-1(1)	-1(1)	0(1)
C(9B)	10(1)	15(1)	13(1)	2(1)	0(1)	-1(1)
C(10B)	10(1)	16(1)	11(1)	1(1)	0(1)	-2(1)
C(11B)	15(1)	24(1)	17(1)	3(1)	-4(1)	-2(1)
C(12B)	14(1)	14(1)	15(1)	0(1)	4(1)	-3(1)
C(13B)	15(1)	22(1)	13(1)	-2(1)	3(1)	0(1)
C(14B)	16(1)	20(1)	16(1)	-3(1)	1(1)	0(1)
C(15B)	15(1)	18(1)	13(1)	-1(1)	0(1)	-2(1)
C(16B)	17(1)	18(1)	19(1)	-2(1)	1(1)	-4(1)
O(1)	20(1)	17(1)	18(1)	-2(1)	6(1)	-1(1)
C(1)	57(2)	33(1)	19(1)	5(1)	-8(1)	-11(1)
O(2)	35(1)	18(1)	13(1)	-1(1)	-1(1)	-5(1)
C(2)	38(2)	46(2)	40(2)	18(1)	-16(1)	-2(1)
O(3)	19(1)	19(1)	23(1)	1(1)	10(1)	4(1)
C(3)	26(1)	18(1)	18(1)	5(1)	-4(1)	-3(1)

O(4)	26(1)	12(1)	26(1)	1(1)	-6(1)	1(1)
C(4)	37(2)	32(1)	22(1)	11(1)	3(1)	-7(1)
O(5)	32(1)	28(1)	24(1)	4(1)	6(1)	-12(1)
C(5)	12(1)	12(1)	18(1)	0(1)	0(1)	1(1)
O(6)	15(1)	19(1)	24(1)	2(1)	3(1)	-3(1)
C(6)	12(1)	17(1)	19(1)	1(1)	1(1)	2(1)
C(7)	22(1)	10(1)	14(1)	-1(1)	1(1)	-2(1)
C(8)	13(1)	12(1)	13(1)	-1(1)	2(1)	-1(1)
C(9)	11(1)	14(1)	13(1)	-1(1)	1(1)	1(1)
C(10)	16(1)	12(1)	12(1)	-2(1)	0(1)	1(1)
C(11)	25(1)	18(1)	18(1)	-6(1)	1(1)	-1(1)
C(12)	13(1)	18(1)	17(1)	2(1)	-1(1)	-2(1)
C(13)	14(1)	18(1)	24(1)	3(1)	-4(1)	2(1)
C(14)	22(1)	12(1)	19(1)	2(1)	-5(1)	-2(1)
C(15)	21(1)	9(1)	17(1)	0(1)	-2(1)	-1(1)
C(16)	22(1)	15(1)	18(1)	-2(1)	2(1)	-6(1)
O(1A)	18(1)	12(1)	17(1)	0(1)	3(1)	-2(1)
C(1A)	30(1)	28(1)	19(1)	-6(1)	2(1)	2(1)
O(2A)	21(1)	20(1)	20(1)	-5(1)	6(1)	-3(1)
C(2A)	44(2)	30(1)	15(1)	1(1)	-3(1)	0(1)
O(3A)	25(1)	9(1)	21(1)	1(1)	-6(1)	-2(1)
C(3A)	24(1)	19(1)	15(1)	-3(1)	-5(1)	3(1)
O(4A)	17(1)	23(1)	18(1)	5(1)	-6(1)	1(1)
C(4A)	22(1)	22(1)	25(1)	-7(1)	-7(1)	3(1)
O(5A)	17(1)	23(1)	25(1)	-4(1)	0(1)	-7(1)
C(5A)	15(1)	14(1)	16(1)	1(1)	-2(1)	2(1)
O(6A)	17(1)	18(1)	18(1)	3(1)	2(1)	-7(1)
C(6A)	20(1)	13(1)	13(1)	3(1)	2(1)	0(1)
C(7A)	11(1)	14(1)	16(1)	1(1)	0(1)	0(1)
C(8A)	12(1)	13(1)	15(1)	0(1)	0(1)	-1(1)
C(9A)	14(1)	12(1)	14(1)	2(1)	-1(1)	-3(1)
C(10A)	13(1)	13(1)	14(1)	1(1)	1(1)	2(1)
C(11A)	19(1)	20(1)	21(1)	-2(1)	2(1)	4(1)
C(12A)	15(1)	17(1)	13(1)	-1(1)	2(1)	-3(1)
C(13A)	17(1)	15(1)	16(1)	5(1)	-1(1)	-2(1)
C(14A)	17(1)	18(1)	14(1)	3(1)	-1(1)	-1(1)

C(15A)	12(1)	16(1)	15(1)	1(1)	-1(1)	1(1)
C(16A)	11(1)	18(1)	21(1)	-1(1)	3(1)	-1(1)

Supplementary Table 26. Hydrogen coordinates (x 10^4) and isotropic displacement parameters (Å^{2}x 10 ^{3}) for Shenvi240.

	x	y	z	U(eq)
H(1BA)	8014	4366	5667	26
H(1BB)	8607	5463	5618	26
H(2BA)	7938	4249	4729	27
H(2BB)	8530	5347	4680	27
H(3B)	6033	6037	6449	26
H(4BA)	5649	4874	5555	35
H(4BB)	6265	4024	5241	35
H(4BC)	5544	4827	4938	35
H(5B)	6293	6419	4788	16
H(6B)	8072	7077	4677	18
H(8B)	5323	6656	5556	15
H(11A)	8676	6191	6376	28
H(11B)	8501	7106	6751	28
H(11C)	9504	7106	6339	28
H(12B)	9055	6788	5445	17
H(13A)	5317	7911	6278	20
H(13B)	6138	7664	6755	20
H(14B)	6384	9354	6146	21
H(1A)	2387	-2351	8706	43
H(1B)	3167	-1753	8273	43
H(2A)	4205	-3251	8086	49
H(2B)	3424	-3848	8519	49
H(3)	396	-921	7763	30
H(4A)	1361	-4235	7899	46
H(4B)	717	-3242	8028	46
H(4C)	1401	-3796	8472	46

H(5)	2604	-3581	7236	17
H(6)	4262	-2696	7220	19
H(8)	903	-2825	7070	15
H(11G)	3029	429	7727	31
H(11H)	2349	-345	8068	31
H(11I)	1687	389	7697	31
H(12)	3905	-1251	7671	19
H(13E)	237	-1295	6592	23
H(13F)	131	-352	6958	23
H(14)	1556	-402	6093	21
H(1AA)	2821	3498	5043	31
H(1AB)	2040	2628	4773	31
H(2AA)	765	3855	4436	36
H(2AB)	1546	4726	4707	36
H(3A)	2012	1824	6296	27
H(4AA)	204	2453	5569	34
H(4AB)	59	2519	4955	34
H(4AC)	-610	3256	5322	34
H(5A)	427	4713	5672	18
H(6A)	2127	5496	5526	19
H(8A)	275	3554	6322	16
H(11D)	4726	3095	5995	30
H(11E)	3689	2532	5739	30
H(11F)	4051	2296	6322	30
H(12A)	3522	4305	5548	18
H(13C)	1310	3135	7165	19
H(13D)	2304	2353	7090	19
H(14A)	2693	4241	7475	20

HPXN (20) (CCDC 2048202)

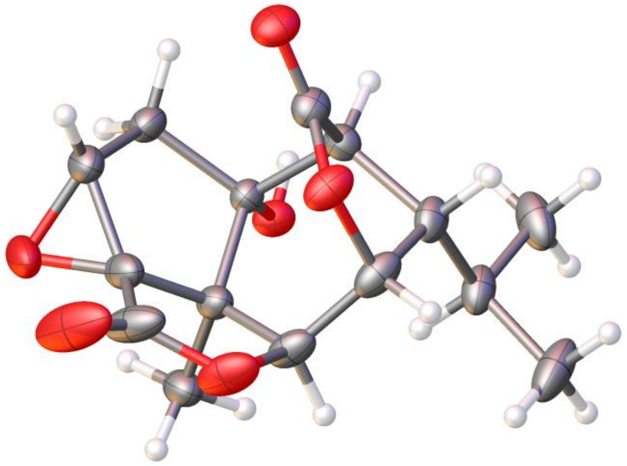

The single crystal X-ray diffraction studies were carried out on a Bruker SMART APEX II CCD diffractometer equipped with Cu K_{α} radiation (λ = 1.54178). Crystals of the subject compound were used as received. A 0.240 x 0.180 x 0.145 mm colorless block crystal was mounted on a Cryoloop with Paratone oil.

Data were collected in a nitrogen gas stream at 100(2) K using ϕ and ϖ scans. Crystal-to-detector distance was 40 mm using exposure time 1.0, 2.0, and 4.0 second (depending on the 2θ position) with a scan width of 1.50°. Data collection was 100.0% complete to 67.679° in θ. A total of 22652 reflections were collected. 2658 reflections were found to be symmetry independent, with a R_{int} of 0.0293. Indexing and unit cell refinement indicated a **Primitive Orthorhombic** lattice. The space group was found to be $\boldsymbol{P2_12_12_1}$. The data were integrated using the Bruker SAINT Software program and scaled using the SADABS software program. Solution by direct methods (SHELXT) produced a complete phasing model consistent with the proposed structure.

All nonhydrogen atoms were refined anisotropically by full-matrix least-squares (SHELXL-2014). All carbon bonded hydrogen atoms were placed using a riding model. Their positions were constrained relative to their parent atom using the appropriate HFIX command in SHELXL-2014.

Supplementary Table 27. Crystal data and structure refinement for Shenvi227.

Report date	2020-12-03	
Identification code	shenvi227	
Empirical formula	C15 H18 O6	
Molecular formula	C15 H18 O6	
Formula weight	294.29	
Temperature	100.0 K	
Wavelength	1.54178 Å	
Crystal system	Orthorhombic	
Space group	$P2_12_12_1$	
Unit cell dimensions	a = 7.83240(10) Å	α= 90°.
	b = 10.8386(2) Å	β= 90°.
	c = 16.2377(3) Å	γ = 90°.
Volume	1378.46(4) $Å^3$	
Z	4	
Density (calculated)	1.418 Mg/m^3	
Absorption coefficient	0.924 mm^{-1}	
F(000)	624	
Crystal size	0.24 x 0.18 x 0.145 mm^3	
Crystal color, habit	colorless block	
Theta range for data collection	4.906 to 71.230°.	
Index ranges	-9<=h<=9, -13<=k<=13, -19<=l<=19	
Reflections collected	22652	
Independent reflections	2658 [R(int) = 0.0293]	
Completeness to theta = 67.679°	100.0 %	
Absorption correction	Semi-empirical from equivalents	
Max. and min. transmission	0.7534 and 0.6303	
Refinement method	Full-matrix least-squares on F^2	
Data / restraints / parameters	2658 / 0 / 195	
Goodness-of-fit on F^2	1.030	
Final R indices [I>2sigma(I)]	R1 = 0.0272, wR2 = 0.0692	
R indices (all data)	R1 = 0.0273, wR2 = 0.0694	
Absolute structure parameter	0.03(3)	
Extinction coefficient	0.0024(4)	
Largest diff. peak and hole	0.192 and -0.204 e.$Å^{-3}$	

Supplementary Table 28. Atomic coordinates (x 10^4) and equivalent isotropic displacement parameters ($Å^2$x 10^3) for Shenvi227. U(eq) is defined as one third of the trace of the orthogonalized U^{ij} tensor.

	x	y	z	U(eq)
O(1)	6627(2)	2777(1)	7413(1)	35(1)
O(2)	4284(2)	3830(1)	7712(1)	34(1)
O(3)	1600(2)	4535(1)	6795(1)	46(1)
O(4)	2938(2)	5094(2)	5634(1)	74(1)
O(5)	2857(2)	2346(2)	5266(1)	44(1)
O(6)	2053(2)	210(1)	7218(1)	25(1)
C(1)	152(3)	2542(2)	9312(1)	51(1)
C(2)	1222(3)	1673(2)	8776(1)	32(1)
C(3)	1739(3)	519(2)	9260(1)	44(1)
C(4)	2832(2)	2329(2)	8471(1)	29(1)
C(5)	3986(2)	1696(2)	7829(1)	24(1)
C(6)	5159(2)	2760(2)	7625(1)	28(1)
C(7)	3011(2)	1281(1)	7040(1)	21(1)
C(8)	4303(2)	1080(2)	6320(1)	27(1)
C(9)	4300(2)	2256(2)	5823(1)	31(1)
C(10)	2744(2)	2944(2)	6045(1)	32(1)
C(11)	2493(3)	4301(2)	6101(2)	47(1)
C(12)	1744(2)	2306(2)	6719(1)	25(1)
C(13)	1383(2)	3423(2)	7295(1)	31(1)
C(14)	2574(2)	3548(2)	8024(1)	32(1)
C(15)	61(2)	1771(2)	6398(1)	32(1)

Supplementary Table 29. Bond lengths [Å] and angles [°] for Shenvi227.

O(1)-C(6)	1.201(2)	C(12)-C(13)	1.556(2)
O(2)-C(6)	1.355(2)	C(12)-C(15)	1.532(3)
O(2)-C(14)	1.464(2)	C(13)-H(13)	1.0000
O(3)-C(11)	1.350(3)	C(13)-C(14)	1.513(3)
O(3)-C(13)	1.464(2)	C(14)-H(14)	1.0000
O(4)-C(11)	1.198(3)	C(15)-H(15A)	0.9800
O(5)-C(9)	1.450(2)	C(15)-H(15B)	0.9800
O(5)-C(10)	1.423(2)	C(15)-H(15C)	0.9800
O(6)-H(6)	0.8400		
O(6)-C(7)	1.413(2)	C(6)-O(2)-C(14)	108.64(13)
C(1)-H(1A)	0.9800	C(11)-O(3)-C(13)	111.63(15)
C(1)-H(1B)	0.9800	C(10)-O(5)-C(9)	61.66(12)
C(1)-H(1C)	0.9800	C(7)-O(6)-H(6)	109.5
C(1)-C(2)	1.533(3)	H(1A)-C(1)-H(1B)	109.5
C(2)-H(2)	1.0000	H(1A)-C(1)-H(1C)	109.5
C(2)-C(3)	1.533(3)	H(1B)-C(1)-H(1C)	109.5
C(2)-C(4)	1.529(2)	C(2)-C(1)-H(1A)	109.5
C(3)-H(3A)	0.9800	C(2)-C(1)-H(1B)	109.5
C(3)-H(3B)	0.9800	C(2)-C(1)-H(1C)	109.5
C(3)-H(3C)	0.9800	C(1)-C(2)-H(2)	108.8
C(4)-H(4)	1.0000	C(3)-C(2)-C(1)	110.73(18)
C(4)-C(5)	1.542(2)	C(3)-C(2)-H(2)	108.8
C(4)-C(14)	1.521(3)	C(4)-C(2)-C(1)	110.43(16)
C(5)-H(5)	1.0000	C(4)-C(2)-H(2)	108.8
C(5)-C(6)	1.511(2)	C(4)-C(2)-C(3)	109.15(17)
C(5)-C(7)	1.557(2)	C(2)-C(3)-H(3A)	109.5
C(7)-C(8)	1.561(2)	C(2)-C(3)-H(3B)	109.5
C(7)-C(12)	1.578(2)	C(2)-C(3)-H(3C)	109.5
C(8)-H(8A)	0.9900	H(3A)-C(3)-H(3B)	109.5
C(8)-H(8B)	0.9900	H(3A)-C(3)-H(3C)	109.5
C(8)-C(9)	1.508(3)	H(3B)-C(3)-H(3C)	109.5
C(9)-H(9)	1.0000	C(2)-C(4)-H(4)	107.2
C(9)-C(10)	1.473(3)	C(2)-C(4)-C(5)	119.68(15)
C(10)-C(11)	1.487(3)	C(5)-C(4)-H(4)	107.2
C(10)-C(12)	1.513(2)	C(14)-C(4)-C(2)	116.68(17)

C(14)-C(4)-H(4)	107.2	O(4)-C(11)-C(10)	129.2(2)
C(14)-C(4)-C(5)	98.10(13)	C(10)-C(12)-C(7)	103.59(13)
C(4)-C(5)-H(5)	111.2	C(10)-C(12)-C(13)	100.01(14)
C(4)-C(5)-C(7)	113.42(14)	C(10)-C(12)-C(15)	111.86(15)
C(6)-C(5)-C(4)	99.48(14)	C(13)-C(12)-C(7)	117.61(14)
C(6)-C(5)-H(5)	111.2	C(15)-C(12)-C(7)	112.79(14)
C(6)-C(5)-C(7)	109.83(13)	C(15)-C(12)-C(13)	110.01(14)
C(7)-C(5)-H(5)	111.2	O(3)-C(13)-C(12)	106.60(14)
O(1)-C(6)-O(2)	120.09(17)	O(3)-C(13)-H(13)	109.3
O(1)-C(6)-C(5)	131.05(18)	O(3)-C(13)-C(14)	106.79(15)
O(2)-C(6)-C(5)	108.86(15)	C(12)-C(13)-H(13)	109.3
O(6)-C(7)-C(5)	109.21(13)	C(14)-C(13)-C(12)	115.32(14)
O(6)-C(7)-C(8)	112.52(13)	C(14)-C(13)-H(13)	109.3
O(6)-C(7)-C(12)	108.22(13)	O(2)-C(14)-C(4)	103.02(15)
C(5)-C(7)-C(8)	109.75(14)	O(2)-C(14)-C(13)	108.22(15)
C(5)-C(7)-C(12)	112.12(13)	O(2)-C(14)-H(14)	111.0
C(8)-C(7)-C(12)	105.00(13)	C(4)-C(14)-H(14)	111.0
C(7)-C(8)-H(8A)	110.5	C(13)-C(14)-C(4)	112.18(14)
C(7)-C(8)-H(8B)	110.5	C(13)-C(14)-H(14)	111.0
H(8A)-C(8)-H(8B)	108.7	C(12)-C(15)-H(15A)	109.5
C(9)-C(8)-C(7)	106.33(14)	C(12)-C(15)-H(15B)	109.5
C(9)-C(8)-H(8A)	110.5	C(12)-C(15)-H(15C)	109.5
C(9)-C(8)-H(8B)	110.5	H(15A)-C(15)-H(15B)	109.5
O(5)-C(9)-C(8)	113.05(16)	H(15A)-C(15)-H(15C)	109.5
O(5)-C(9)-H(9)	120.7	H(15B)-C(15)-H(15C)	109.5
O(5)-C(9)-C(10)	58.26(12)		
C(8)-C(9)-H(9)	120.7		
C(10)-C(9)-C(8)	107.36(15)		
C(10)-C(9)-H(9)	120.7		
O(5)-C(10)-C(9)	60.08(12)		
O(5)-C(10)-C(11)	120.89(17)		
O(5)-C(10)-C(12)	117.83(17)		
C(9)-C(10)-C(11)	128.67(18)		
C(9)-C(10)-C(12)	111.94(15)		
C(11)-C(10)-C(12)	109.82(16)		
O(3)-C(11)-C(10)	107.82(16)		
O(4)-C(11)-O(3)	123.0(2)		

Supplementary Table 30. Anisotropic displacement parameters ($Å^2$x 10^3) for Shenvi227. The anisotropic displacement factor exponent takes the form: $-2\pi^2[h^2 a^{*2}U^{11} + ... + 2 h k a^* b^* U^{12}]$

	U^{11}	U^{22}	U^{33}	U^{23}	U^{13}	U^{12}
O(1)	25(1)	36(1)	43(1)	-3(1)	3(1)	-6(1)
O(2)	32(1)	24(1)	46(1)	-5(1)	12(1)	-6(1)
O(3)	40(1)	28(1)	70(1)	21(1)	18(1)	11(1)
O(4)	54(1)	62(1)	106(2)	60(1)	36(1)	21(1)
O(5)	31(1)	79(1)	22(1)	11(1)	1(1)	5(1)
O(6)	24(1)	20(1)	32(1)	-1(1)	1(1)	0(1)
C(1)	50(1)	63(2)	41(1)	-24(1)	22(1)	-23(1)
C(2)	34(1)	39(1)	22(1)	-5(1)	5(1)	-13(1)
C(3)	48(1)	59(1)	27(1)	14(1)	-5(1)	-22(1)
C(4)	31(1)	34(1)	21(1)	-6(1)	2(1)	-10(1)
C(5)	24(1)	23(1)	25(1)	2(1)	-2(1)	-2(1)
C(6)	27(1)	28(1)	28(1)	-3(1)	0(1)	-4(1)
C(7)	21(1)	21(1)	21(1)	-1(1)	2(1)	0(1)
C(8)	23(1)	32(1)	26(1)	-3(1)	4(1)	3(1)
C(9)	25(1)	44(1)	24(1)	4(1)	3(1)	2(1)
C(10)	25(1)	42(1)	28(1)	14(1)	4(1)	5(1)
C(11)	29(1)	45(1)	69(2)	31(1)	15(1)	11(1)
C(12)	22(1)	29(1)	24(1)	6(1)	3(1)	3(1)
C(13)	29(1)	22(1)	43(1)	7(1)	12(1)	5(1)
C(14)	32(1)	24(1)	42(1)	-9(1)	14(1)	-6(1)
C(15)	24(1)	45(1)	28(1)	7(1)	-1(1)	2(1)

Supplementary Table 31. Hydrogen coordinates (x 10^4) and isotropic displacement parameters ($Å^2$x 10^3) for Shenvi227.

	x	y	z	U(eq)
H(6)	2715	-358	7367	38
H(1A)	844	2837	9775	77
H(1B)	-846	2097	9524	77
H(1C)	-228	3247	8982	77
H(2)	527	1416	8289	38
H(3A)	2338	-55	8894	67
H(3B)	715	118	9481	67
H(3C)	2494	754	9715	67
H(4)	3555	2503	8966	35
H(5)	4633	994	8078	29
H(8A)	5459	911	6539	32
H(8B)	3946	373	5974	32
H(9)	5390	2700	5703	37
H(13)	178	3379	7497	38
H(14)	2169	4203	8411	39
H(15A)	301	1139	5983	49
H(15B)	-623	2432	6151	49
H(15C)	-574	1401	6856	49

H5MePXN (21) (CCDC 2048203)

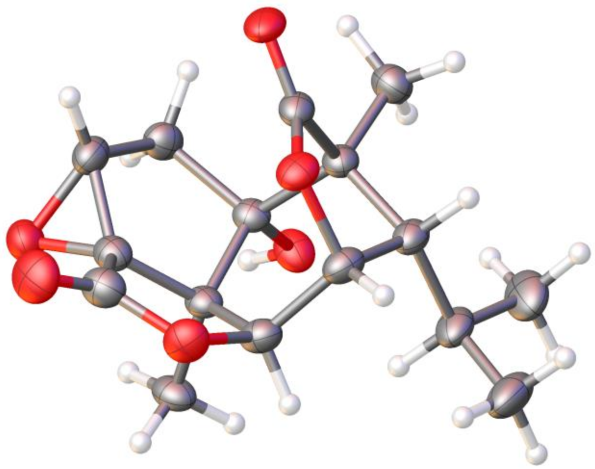

The single crystal X-ray diffraction studies were carried out on a Bruker SMART APEX II CCD diffractometer equipped with Cu K_α radiation (λ = 1.54178). Crystals of the subject compound were used as received. A 0.200 x 0.160 x 0.125 mm colorless block crystal was mounted on a Cryoloop with Paratone oil.

Data were collected in a nitrogen gas stream at 100(2) K using ϕ and ϖ scans. Crystal-to-detector distance was 40 mm using exposure time 5.0, 10.0, and 30.0 second (depending on the 2θ position) with a scan width of 1.25°. Data collection was 97.7% complete to 67.700° in θ. A total of 12737 reflections were collected. 2748 reflections were found to be symmetry independent, with a R_{int} of 0.0399. Indexing and unit cell refinement indicated a **Primitive Monoclinic** lattice. The space group was found to be $\boldsymbol{P2_1}$. The data were integrated using the Bruker SAINT Software program and scaled using the SADABS software program. Solution by direct methods (SHELXT) produced a complete phasing model consistent with the proposed structure.

All nonhydrogen atoms were refined anisotropically by full-matrix least-squares (SHELXL-2014). All carbon bonded hydrogen atoms were placed using a riding model. Their positions were constrained relative to their parent atom using the appropriate HFIX command in SHELXL-2014.

Supplementary Table 32. Crystal data and structure refinement for Shenvi228.

Report date	2020-12-03	
Identification code	shenvi228	
Empirical formula	C16 H20 O6	
Molecular formula	C16 H20 O6	
Formula weight	308.32	
Temperature	100.0 K	
Wavelength	1.54178 Å	
Crystal system	Monoclinic	
Space group	P 1 21 1	
Unit cell dimensions	a = 7.4939(3) Å	α= 90°.
	b = 12.1595(4) Å	β= 113.329(2)°.
	c = 8.7947(3) Å	γ = 90°.
Volume	735.87(5) Å^3	
Z	2	
Density (calculated)	1.391 Mg/m^3	
Absorption coefficient	0.890 mm^{-1}	
F(000)	328	
Crystal size	0.2 x 0.16 x 0.125 mm^3	
Crystal color, habit	colorless block	
Theta range for data collection	5.478 to 70.913°.	
Index ranges	-8<=h<=7, -14<=k<=14, -10<=l<=10	
Reflections collected	12737	
Independent reflections	2748 [R(int) = 0.0399]	
Completeness to theta = 67.000°	97.7 %	
Absorption correction	Semi-empirical from equivalents	
Max. and min. transmission	0.7534 and 0.5766	
Refinement method	Full-matrix least-squares on F^2	
Data / restraints / parameters	2748 / 1 / 204	
Goodness-of-fit on F^2	1.035	
Final R indices [I>2sigma(I)]	R1 = 0.0361, wR2 = 0.0923	
R indices (all data)	R1 = 0.0374, wR2 = 0.0939	
Absolute structure parameter	-0.05(6)	
Largest diff. peak and hole	0.223 and -0.174 e.Å^{-3}	

Supplementary Table 33. Atomic coordinates (x 10^4) and equivalent isotropic displacement parameters ($Å^2$x 10^3) for Shenvi228. U(eq) is defined as one third of the trace of the orthogonalized U^{ij} tensor.

	x	y	z	U(eq)
O(1)	833(3)	5125(2)	1910(2)	34(1)
O(2)	4737(3)	4123(2)	2478(3)	45(1)
O(3)	5789(3)	5156(2)	4763(2)	36(1)
O(4)	473(3)	6516(2)	5988(2)	32(1)
O(5)	2042(3)	2957(1)	6370(2)	34(1)
O(6)	4725(3)	3905(1)	6818(2)	30(1)
C(1)	6648(5)	6874(3)	9722(4)	50(1)
C(2)	4476(4)	6621(2)	8781(3)	36(1)
C(3)	3367(6)	6952(3)	9834(4)	50(1)
C(4)	4235(4)	5392(2)	8321(3)	28(1)
C(5)	2231(3)	4912(2)	7184(3)	25(1)
C(6)	2911(4)	3815(2)	6739(3)	27(1)
C(7)	1282(3)	5583(2)	5533(3)	24(1)
C(8)	2786(3)	5893(2)	4749(3)	25(1)
C(9)	4987(4)	5717(2)	5824(3)	30(1)
C(10)	5415(3)	5017(2)	7354(3)	31(1)
C(11)	-296(3)	4883(2)	4168(3)	30(1)
C(12)	697(4)	4377(2)	3139(3)	30(1)
C(13)	2466(4)	5023(2)	3436(3)	28(1)
C(14)	4381(4)	4679(2)	3447(3)	34(1)
C(15)	2445(4)	7047(2)	3989(3)	32(1)
C(16)	817(4)	4700(2)	8011(3)	33(1)

Supplementary Table 34. Bond lengths [Å] and angles [°] for Shenvi228.

O(1)-C(12)	1.447(3)	C(12)-H(12)	1.0000
O(1)-C(13)	1.419(3)	C(12)-C(13)	1.473(4)
O(2)-C(14)	1.197(3)	C(13)-C(14)	1.491(4)
O(3)-C(9)	1.463(3)	C(15)-H(15A)	0.9800
O(3)-C(14)	1.350(3)	C(15)-H(15B)	0.9800
O(4)-H(4)	0.8400	C(15)-H(15C)	0.9800
O(4)-C(7)	1.417(3)	C(16)-H(16A)	0.9800
O(5)-C(6)	1.205(3)	C(16)-H(16B)	0.9800
O(6)-C(6)	1.338(3)	C(16)-H(16C)	0.9800
O(6)-C(10)	1.458(3)		
C(1)-H(1A)	0.9800	C(13)-O(1)-C(12)	61.84(15)
C(1)-H(1B)	0.9800	C(14)-O(3)-C(9)	111.6(2)
C(1)-H(1C)	0.9800	C(7)-O(4)-H(4)	109.5
C(1)-C(2)	1.537(4)	C(6)-O(6)-C(10)	108.36(18)
C(2)-H(2)	1.0000	H(1A)-C(1)-H(1B)	109.5
C(2)-C(3)	1.524(4)	H(1A)-C(1)-H(1C)	109.5
C(2)-C(4)	1.541(3)	H(1B)-C(1)-H(1C)	109.5
C(3)-H(3A)	0.9800	C(2)-C(1)-H(1A)	109.5
C(3)-H(3B)	0.9800	C(2)-C(1)-H(1B)	109.5
C(3)-H(3C)	0.9800	C(2)-C(1)-H(1C)	109.5
C(4)-H(4A)	1.0000	C(1)-C(2)-H(2)	108.6
C(4)-C(5)	1.551(3)	C(1)-C(2)-C(4)	109.0(2)
C(4)-C(10)	1.519(4)	C(3)-C(2)-C(1)	109.7(2)
C(5)-C(6)	1.533(3)	C(3)-C(2)-H(2)	108.6
C(5)-C(7)	1.569(3)	C(3)-C(2)-C(4)	112.4(2)
C(5)-C(16)	1.526(3)	C(4)-C(2)-H(2)	108.6
C(7)-C(8)	1.582(3)	C(2)-C(3)-H(3A)	109.5
C(7)-C(11)	1.562(3)	C(2)-C(3)-H(3B)	109.5
C(8)-C(9)	1.557(3)	C(2)-C(3)-H(3C)	109.5
C(8)-C(13)	1.514(3)	H(3A)-C(3)-H(3B)	109.5
C(8)-C(15)	1.531(3)	H(3A)-C(3)-H(3C)	109.5
C(9)-H(9)	1.0000	H(3B)-C(3)-H(3C)	109.5
C(9)-C(10)	1.516(3)	C(2)-C(4)-H(4A)	107.3
C(10)-H(10)	1.0000	C(2)-C(4)-C(5)	121.2(2)
C(11)-H(11A)	0.9900	C(5)-C(4)-H(4A)	107.3
C(11)-H(11B)	0.9900	C(10)-C(4)-C(2)	113.9(2)
C(11)-C(12)	1.511(4)	C(10)-C(4)-H(4A)	107.3

C(10)-C(4)-C(5)	98.97(17)	C(12)-C(11)-H(11B)	110.5
C(4)-C(5)-C(7)	111.66(18)	O(1)-C(12)-C(11)	112.9(2)
C(6)-C(5)-C(4)	99.01(18)	O(1)-C(12)-H(12)	120.8
C(6)-C(5)-C(7)	106.93(17)	O(1)-C(12)-C(13)	58.16(15)
C(16)-C(5)-C(4)	115.73(19)	C(11)-C(12)-H(12)	120.8
C(16)-C(5)-C(6)	109.78(19)	C(13)-C(12)-C(11)	107.4(2)
C(16)-C(5)-C(7)	112.54(19)	C(13)-C(12)-H(12)	120.8
O(5)-C(6)-O(6)	120.9(2)	O(1)-C(13)-C(8)	118.5(2)
O(5)-C(6)-C(5)	128.6(2)	O(1)-C(13)-C(12)	59.99(16)
O(6)-C(6)-C(5)	110.46(19)	O(1)-C(13)-C(14)	119.9(2)
O(4)-C(7)-C(5)	104.05(17)	C(12)-C(13)-C(8)	111.5(2)
O(4)-C(7)-C(8)	112.98(18)	C(12)-C(13)-C(14)	130.4(2)
O(4)-C(7)-C(11)	112.04(19)	C(14)-C(13)-C(8)	109.3(2)
C(5)-C(7)-C(8)	112.79(18)	O(2)-C(14)-O(3)	122.3(3)
C(11)-C(7)-C(5)	110.82(18)	O(2)-C(14)-C(13)	129.7(3)
C(11)-C(7)-C(8)	104.38(18)	O(3)-C(14)-C(13)	107.9(2)
C(9)-C(8)-C(7)	117.97(19)	C(8)-C(15)-H(15A)	109.5
C(13)-C(8)-C(7)	103.96(18)	C(8)-C(15)-H(15B)	109.5
C(13)-C(8)-C(9)	100.16(18)	C(8)-C(15)-H(15C)	109.5
C(13)-C(8)-C(15)	111.02(19)	H(15A)-C(15)-H(15B)	109.5
C(15)-C(8)-C(7)	112.6(2)	H(15A)-C(15)-H(15C)	109.5
C(15)-C(8)-C(9)	110.12(19)	H(15B)-C(15)-H(15C)	109.5
O(3)-C(9)-C(8)	106.32(19)	C(5)-C(16)-H(16A)	109.5
O(3)-C(9)-H(9)	109.3	C(5)-C(16)-H(16B)	109.5
O(3)-C(9)-C(10)	107.95(19)	C(5)-C(16)-H(16C)	109.5
C(8)-C(9)-H(9)	109.3	H(16A)-C(16)-H(16B)	109.5
C(10)-C(9)-C(8)	114.6(2)	H(16A)-C(16)-H(16C)	109.5
C(10)-C(9)-H(9)	109.3	H(16B)-C(16)-H(16C)	109.5
O(6)-C(10)-C(4)	104.42(19)		
O(6)-C(10)-C(9)	108.11(18)		
O(6)-C(10)-H(10)	110.9		
C(4)-C(10)-H(10)	110.9		
C(9)-C(10)-C(4)	111.3(2)		
C(9)-C(10)-H(10)	110.9		
C(7)-C(11)-H(11A)	110.5		
C(7)-C(11)-H(11B)	110.5		
H(11A)-C(11)-H(11B)	108.7		
C(12)-C(11)-C(7)	106.24(19)		
C(12)-C(11)-H(11A)	110.5		

Supplementary Table 35. Anisotropic displacement parameters ($Å^2 x 10^3$) for Shenvi228. The anisotropic displacement factor exponent takes the form: $-2\pi^2[h^2 a^{*2}U^{11} + ... + 2 h k a^* b^* U^{12}]$

	U^{11}	U^{22}	U^{33}	U^{23}	U^{13}	U^{12}
O(1)	38(1)	34(1)	28(1)	2(1)	12(1)	1(1)
O(2)	53(1)	44(1)	48(1)	-9(1)	29(1)	8(1)
O(3)	30(1)	39(1)	43(1)	-3(1)	18(1)	1(1)
O(4)	39(1)	25(1)	33(1)	4(1)	17(1)	11(1)
O(5)	46(1)	18(1)	34(1)	0(1)	11(1)	-4(1)
O(6)	33(1)	19(1)	37(1)	-2(1)	12(1)	4(1)
C(1)	58(2)	32(1)	43(1)	-6(1)	1(1)	-9(1)
C(2)	51(2)	20(1)	30(1)	-5(1)	10(1)	-2(1)
C(3)	81(2)	31(1)	42(2)	-10(1)	29(1)	1(1)
C(4)	34(1)	19(1)	27(1)	-1(1)	7(1)	0(1)
C(5)	30(1)	18(1)	26(1)	0(1)	11(1)	0(1)
C(6)	34(1)	18(1)	25(1)	2(1)	9(1)	1(1)
C(7)	27(1)	18(1)	28(1)	2(1)	12(1)	3(1)
C(8)	32(1)	17(1)	29(1)	1(1)	14(1)	0(1)
C(9)	31(1)	24(1)	37(1)	-3(1)	16(1)	-4(1)
C(10)	28(1)	23(1)	35(1)	-2(1)	7(1)	-2(1)
C(11)	26(1)	31(1)	31(1)	2(1)	9(1)	-1(1)
C(12)	33(1)	26(1)	27(1)	-2(1)	6(1)	-2(1)
C(13)	34(1)	21(1)	30(1)	1(1)	15(1)	2(1)
C(14)	38(1)	29(1)	40(1)	1(1)	21(1)	3(1)
C(15)	45(1)	20(1)	38(1)	4(1)	23(1)	2(1)
C(16)	40(1)	30(1)	33(1)	3(1)	18(1)	1(1)

Supplementary Table 36. Hydrogen coordinates (x 10^4) and isotropic displacement parameters (Å^{2}x 10^3) for Shenvi228.

	x	y	z	U(eq)
H(4)	-408	6783	5142	47
H(1A)	7161	6442	10745	75
H(1B)	6817	7660	9993	75
H(1C)	7352	6684	9027	75
H(2)	3975	7064	7738	43
H(3A)	1969	6860	9190	75
H(3B)	3643	7723	10168	75
H(3C)	3774	6486	10823	75
H(4A)	4703	4965	9379	33
H(9)	5633	6449	6160	36
H(10)	6836	5018	8071	37
H(11A)	-803	4302	4679	36
H(11B)	-1392	5355	3472	36
H(12)	680	3564	2970	37
H(15A)	3345	7178	3449	49
H(15B)	2667	7595	4863	49
H(15C)	1104	7106	3169	49
H(16A)	-161	4162	7359	50
H(16B)	172	5390	8074	50
H(16C)	1531	4413	9130	50

6F5MePXN (23) (CCDC 2069870)

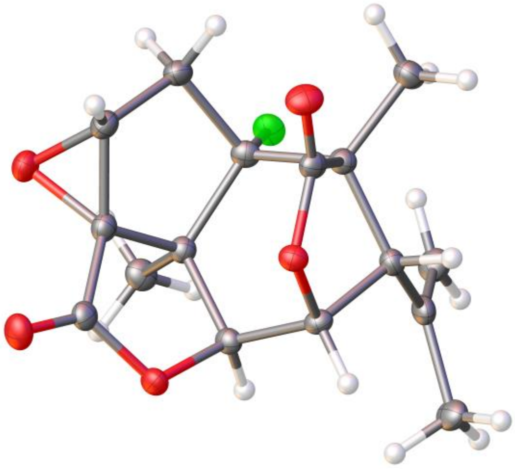

The single crystal X-ray diffraction studies were carried out on a Bruker Microstar APEX II CCD diffractometer equipped with Cu K_α radiation (λ =1.54178 Å).

Crystals of the subject compound were used as received. A 0.25 x 0.13 x 0.10 mm piece of a colorless crystal was mounted on a Cryoloop with Paratone oil. Data were collected in a nitrogen gas stream at 100(2) K using ϕ and ϖ scans. Crystal-to-detector distance was 40 mm and exposure time was 1, 2, 3, or 5 seconds depending on the 2θ range per frame using a scan width of 1.25°. Data collection was 99.8 % complete to 67.679° in θ. A total of 8824 reflections were collected covering the indices, -11<=h<=11, -8<=k<=8, -11<=l<=12. 2527 reflections were found to be symmetry independent, with a R_{int} of 0.0273. Indexing and unit cell refinement indicated a **P**rimitive, **Monoclinic** lattice. The space group was found to be ***P*2₁**. The data were integrated using the Bruker SAINT Software program and scaled using the SADABS software program. Solution by direct methods (SHELXT) produced a complete phasing model consistent with the proposed structure.

All nonhydrogen atoms were refined anisotropically by full-matrix least-squares (SHELXL-2014). All carbon bonded hydrogen atoms were placed using a riding model. Their positions were constrained relative to their parent atom using the appropriate HFIX command in SHELXL-2014.

Supplementary Table 37. Crystal data and structure refinement for Shenvi233.

Identification code	shenvi233_0m_a	
Empirical formula	C16 H17 F O5	
Formula weight	308.29	
Temperature	100 K	
Wavelength	1.54178 Å	
Crystal system	Monoclinic	
Space group	P 1 21 1	
Unit cell dimensions	a = 9.8088(13) Å	α= 90°.
	b = 7.4118(10) Å	β= 105.674(3)°.
	c = 9.9966(14) Å	γ = 90°.
Volume	699.74(16) Å^3	
Z	2	
Density (calculated)	1.463 Mg/m^3	
Absorption coefficient	0.991 mm^{-1}	
F(000)	324	
Crystal size	0.25 x 0.13 x 0.1 mm^3	
Theta range for data collection	4.594 to 68.860°.	
Index ranges	-11<=h<=11, -8<=k<=8, -11<=l<=12	
Reflections collected	8824	
Independent reflections	2527 [R(int) = 0.0273]	
Completeness to theta = 67.679°	99.8 %	
Absorption correction	Semi-empirical from equivalents	
Max. and min. transmission	0.5213 and 0.4303	
Refinement method	Full-matrix least-squares on F^2	
Data / restraints / parameters	2527 / 1 / 202	
Goodness-of-fit on F^2	1.056	
Final R indices [I>2sigma(I)]	R1 = 0.0241, wR2 = 0.0594	
R indices (all data)	R1 = 0.0243, wR2 = 0.0595	
Absolute structure parameter	0.00(4)	
Largest diff. peak and hole	0.193 and -0.155 e.Å^{-3}	

Supplementary Table 38. Atomic coordinates (x 10^4) and equivalent isotropic displacement parameters ($Å^2$x 10^3) for Shenvi233. U(eq) is defined as one third of the trace of the orthogonalized U^{ij} tensor.

	x	y	z	U(eq)
F(1)	9423(1)	5815(2)	9160(1)	19(1)
O(1)	4582(1)	7134(2)	6875(1)	17(1)
O(2)	3192(1)	5929(2)	8095(1)	22(1)
O(3)	5916(1)	5304(2)	10297(1)	21(1)
O(4)	6650(2)	1500(2)	6894(1)	23(1)
O(5)	5724(1)	4110(2)	5966(1)	16(1)
C(1)	7995(2)	8496(3)	4675(2)	23(1)
C(2)	8683(2)	7257(3)	5862(2)	19(1)
C(3)	9957(2)	7610(3)	6685(2)	22(1)
C(4)	7842(2)	5553(3)	5910(2)	15(1)
C(5)	6266(2)	5891(2)	5767(2)	15(1)
C(6)	6085(2)	7136(3)	6906(2)	15(1)
C(7)	6915(2)	6566(3)	8402(2)	15(1)
C(8)	7454(2)	8231(3)	9293(2)	20(1)
C(9)	5752(2)	5602(3)	8853(2)	16(1)
C(10)	4348(2)	6174(2)	7943(2)	16(1)
C(11)	6217(2)	3826(3)	9465(2)	19(1)
C(12)	7782(2)	3656(3)	9577(2)	18(1)
C(13)	8089(2)	5072(3)	8548(2)	16(1)
C(14)	8158(2)	4210(3)	7144(2)	16(1)
C(15)	9466(2)	3030(3)	7300(2)	20(1)
C(16)	6810(2)	3065(3)	6679(2)	16(1)

Supplementary Table 39. Bond lengths [Å] and angles [°] for Shenvi233.

F(1)-C(13)	1.399(2)	C(12)-H(12A)	0.9900
O(1)-C(6)	1.466(2)	C(12)-H(12B)	0.9900
O(1)-C(10)	1.353(2)	C(12)-C(13)	1.555(3)
O(2)-C(10)	1.198(2)	C(13)-C(14)	1.560(2)
O(3)-C(9)	1.426(2)	C(14)-C(15)	1.525(3)
O(3)-C(11)	1.454(2)	C(14)-C(16)	1.534(3)
O(4)-C(16)	1.198(2)	C(15)-H(15A)	0.9800
O(5)-C(5)	1.456(2)	C(15)-H(15B)	0.9800
O(5)-C(16)	1.353(2)	C(15)-H(15C)	0.9800
C(1)-H(1A)	0.9800		
C(1)-H(1B)	0.9800	C(10)-O(1)-C(6)	111.54(13)
C(1)-H(1C)	0.9800	C(9)-O(3)-C(11)	61.44(12)
C(1)-C(2)	1.508(3)	C(16)-O(5)-C(5)	109.05(14)
C(2)-C(3)	1.324(3)	H(1A)-C(1)-H(1B)	109.5
C(2)-C(4)	1.516(3)	H(1A)-C(1)-H(1C)	109.5
C(3)-H(3A)	0.9500	H(1B)-C(1)-H(1C)	109.5
C(3)-H(3B)	0.9500	C(2)-C(1)-H(1A)	109.5
C(4)-H(4)	1.0000	C(2)-C(1)-H(1B)	109.5
C(4)-C(5)	1.534(2)	C(2)-C(1)-H(1C)	109.5
C(4)-C(14)	1.550(3)	C(1)-C(2)-C(4)	113.61(17)
C(5)-H(5)	1.0000	C(3)-C(2)-C(1)	121.01(19)
C(5)-C(6)	1.513(3)	C(3)-C(2)-C(4)	125.15(18)
C(6)-H(6)	1.0000	C(2)-C(3)-H(3A)	120.0
C(6)-C(7)	1.555(3)	C(2)-C(3)-H(3B)	120.0
C(7)-C(8)	1.530(3)	H(3A)-C(3)-H(3B)	120.0
C(7)-C(9)	1.514(3)	C(2)-C(4)-H(4)	106.0
C(7)-C(13)	1.575(3)	C(2)-C(4)-C(5)	113.87(15)
C(8)-H(8A)	0.9800	C(2)-C(4)-C(14)	124.50(16)
C(8)-H(8B)	0.9800	C(5)-C(4)-H(4)	106.0
C(8)-H(8C)	0.9800	C(5)-C(4)-C(14)	99.15(13)
C(9)-C(10)	1.492(3)	C(14)-C(4)-H(4)	106.0
C(9)-C(11)	1.471(3)	O(5)-C(5)-C(4)	103.59(14)
C(11)-H(11)	1.0000	O(5)-C(5)-H(5)	111.1
C(11)-C(12)	1.514(3)	O(5)-C(5)-C(6)	109.18(14)

C(4)-C(5)-H(5)	111.1	C(11)-C(12)-H(12A)	110.6
C(6)-C(5)-C(4)	110.46(14)	C(11)-C(12)-H(12B)	110.6
C(6)-C(5)-H(5)	111.1	C(11)-C(12)-C(13)	105.53(15)
O(1)-C(6)-C(5)	107.74(14)	H(12A)-C(12)-H(12B)	108.8
O(1)-C(6)-H(6)	109.2	C(13)-C(12)-H(12A)	110.6
O(1)-C(6)-C(7)	106.44(13)	C(13)-C(12)-H(12B)	110.6
C(5)-C(6)-H(6)	109.2	F(1)-C(13)-C(7)	109.75(15)
C(5)-C(6)-C(7)	114.87(15)	F(1)-C(13)-C(12)	107.57(14)
C(7)-C(6)-H(6)	109.2	F(1)-C(13)-C(14)	106.92(14)
C(6)-C(7)-C(13)	117.39(15)	C(12)-C(13)-C(7)	105.61(15)
C(8)-C(7)-C(6)	110.44(16)	C(12)-C(13)-C(14)	112.44(15)
C(8)-C(7)-C(13)	112.58(15)	C(14)-C(13)-C(7)	114.37(14)
C(9)-C(7)-C(6)	99.98(14)	C(4)-C(14)-C(13)	113.88(15)
C(9)-C(7)-C(8)	112.12(15)	C(15)-C(14)-C(4)	115.59(14)
C(9)-C(7)-C(13)	103.37(15)	C(15)-C(14)-C(13)	112.49(15)
C(7)-C(8)-H(8A)	109.5	C(15)-C(14)-C(16)	110.26(16)
C(7)-C(8)-H(8B)	109.5	C(16)-C(14)-C(4)	98.23(14)
C(7)-C(8)-H(8C)	109.5	C(16)-C(14)-C(13)	104.80(14)
H(8A)-C(8)-H(8B)	109.5	C(14)-C(15)-H(15A)	109.5
H(8A)-C(8)-H(8C)	109.5	C(14)-C(15)-H(15B)	109.5
H(8B)-C(8)-H(8C)	109.5	C(14)-C(15)-H(15C)	109.5
O(3)-C(9)-C(7)	119.13(15)	H(15A)-C(15)-H(15B)	109.5
O(3)-C(9)-C(10)	119.63(15)	H(15A)-C(15)-H(15C)	109.5
O(3)-C(9)-C(11)	60.20(12)	H(15B)-C(15)-H(15C)	109.5
C(10)-C(9)-C(7)	109.26(16)	O(4)-C(16)-O(5)	121.77(17)
C(11)-C(9)-C(7)	111.83(16)	O(4)-C(16)-C(14)	128.67(18)
C(11)-C(9)-C(10)	129.72(17)	O(5)-C(16)-C(14)	109.54(15)
O(1)-C(10)-C(9)	107.78(15)		
O(2)-C(10)-O(1)	123.21(17)		
O(2)-C(10)-C(9)	128.96(18)		
O(3)-C(11)-C(9)	58.36(12)		
O(3)-C(11)-H(11)	120.9		
O(3)-C(11)-C(12)	112.31(16)		
C(9)-C(11)-H(11)	120.9		
C(9)-C(11)-C(12)	107.46(16)		
C(12)-C(11)-H(11)	120.9		

Symmetry transformations used to generate equivalent atoms:

Supplementary Table 40. Anisotropic displacement parameters ($Å^2$x 10^3) for Shenvi233. The anisotropic displacement factor exponent takes the form: $-2\pi^2$[h^2 $a^{*2}U^{11}$ + ... + 2 h k a* b* U^{12}]

	U^{11}	U^{22}	U^{33}	U^{23}	U^{13}	U^{12}
F(1)	13(1)	24(1)	18(1)	-3(1)	0(1)	-1(1)
O(1)	13(1)	20(1)	18(1)	2(1)	5(1)	3(1)
O(2)	16(1)	30(1)	23(1)	-2(1)	7(1)	-1(1)
O(3)	24(1)	24(1)	15(1)	1(1)	7(1)	2(1)
O(4)	28(1)	15(1)	24(1)	0(1)	4(1)	-3(1)
O(5)	15(1)	14(1)	18(1)	0(1)	3(1)	0(1)
C(1)	21(1)	24(1)	27(1)	8(1)	11(1)	1(1)
C(2)	19(1)	19(1)	22(1)	1(1)	12(1)	2(1)
C(3)	21(1)	23(1)	24(1)	2(1)	8(1)	-3(1)
C(4)	15(1)	16(1)	16(1)	-1(1)	5(1)	2(1)
C(5)	15(1)	14(1)	15(1)	2(1)	3(1)	-1(1)
C(6)	12(1)	15(1)	17(1)	1(1)	4(1)	0(1)
C(7)	16(1)	15(1)	15(1)	0(1)	4(1)	0(1)
C(8)	22(1)	19(1)	19(1)	-4(1)	4(1)	-1(1)
C(9)	18(1)	17(1)	14(1)	-1(1)	5(1)	0(1)
C(10)	19(1)	16(1)	15(1)	-3(1)	6(1)	1(1)
C(11)	22(1)	18(1)	16(1)	2(1)	6(1)	1(1)
C(12)	21(1)	19(1)	16(1)	3(1)	4(1)	4(1)
C(13)	13(1)	16(1)	15(1)	-1(1)	1(1)	-1(1)
C(14)	16(1)	14(1)	16(1)	1(1)	4(1)	1(1)
C(15)	19(1)	20(1)	21(1)	0(1)	5(1)	4(1)
C(16)	19(1)	16(1)	12(1)	-1(1)	3(1)	1(1)

Supplementary Table 41. Hydrogen coordinates (x 10^4) and isotropic displacement parameters ($Å^2$x 10^3) for Shenvi233.

	x	y	z	U(eq)
H(1A)	7781	7820	3800	35
H(1B)	8642	9490	4635	35
H(1C)	7117	8982	4818	35
H(3A)	10453	8658	6533	27
H(3B)	10377	6816	7429	27
H(4)	7882	4831	5076	19
H(5)	5779	6384	4828	18
H(6)	6372	8386	6721	18
H(8A)	7852	7863	10261	30
H(8B)	6667	9069	9233	30
H(8C)	8188	8828	8954	30
H(11)	5577	2754	9243	22
H(12A)	8009	2424	9318	22
H(12B)	8353	3911	10537	22
H(15A)	9490	2101	8004	30
H(15B)	10319	3778	7589	30
H(15C)	9429	2454	6409	30

6F12F5MePXN (30) (CCDC 2079783)

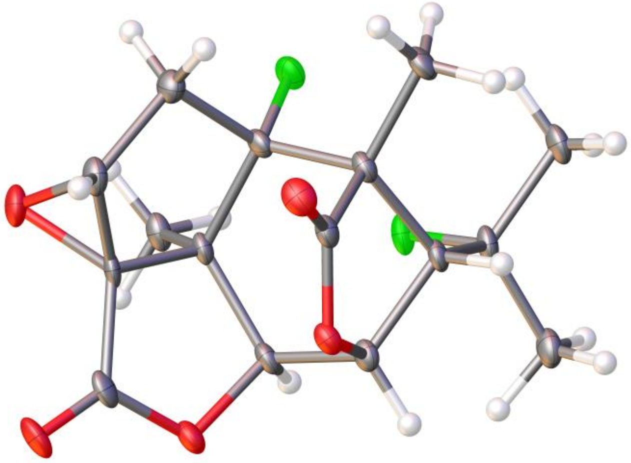

The single crystal X-ray diffraction studies were carried out on a Bruker Smart APEX II CCD diffractometer equipped with Cu K_α radiation (λ =1.54178 Å).

Crystals of the subject compound were used as received. A 0.23 x 0.15 x 0.12 mm piece of a colorless crystal was mounted on a Cryoloop with Paratone oil. Data were collected in a nitrogen gas stream at 100(2) K using ϕ and ϖ scans. Crystal-to-detector distance was 40 mm and exposure time was 1, 2, 6, or 10 seconds depending on the 2θ range per frame using a scan width of 1.25°. Data collection was 99.9 % complete to 67.679° in θ. A total of 11148 reflections were collected covering the indices, -10<=h<=10, -9<=k<=9, -14<=l<=14. 2737 reflections were found to be symmetry independent, with a R_{int} of 0.0493. Indexing and unit cell refinement indicated a **P**rimitive, **Monoclinic** lattice. The space group was found to be ***P*2₁**. The data were integrated using the Bruker SAINT Software program and scaled using the SADABS software program. Solution by direct methods (SHELXT) produced a complete phasing model consistent with the proposed structure.

All nonhydrogen atoms were refined anisotropically by full-matrix least-squares (SHELXL-2014). All carbon bonded hydrogen atoms were placed using a riding model. Their positions were constrained relative to their parent atom using the appropriate HFIX command in SHELXL-2014.

Supplementary Table 42. Crystal data and structure refinement for Shenvi244.

Identification code	shenvi244_0m_a	
Empirical formula	C16 H18 F2 O5	
Formula weight	328.30	
Temperature	100.0 K	
Wavelength	1.54178 Å	
Crystal system	Monoclinic	
Space group	P 1 21 1	
Unit cell dimensions	a = 8.6175(3) Å	α= 90°.
	b = 7.4251(3) Å	β= 108.6030(10)°.
	c = 11.9211(5) Å	γ = 90°.
Volume	722.93(5) Å^3	
Z	2	
Density (calculated)	1.508 Mg/m^3	
Absorption coefficient	1.097 mm^{-1}	
F(000)	344	
Crystal size	0.23 x 0.15 x 0.12 mm^3	
Theta range for data collection	3.912 to 70.459°.	
Index ranges	-10<=h<=10, -9<=k<=9, -14<=l<=14	
Reflections collected	11148	
Independent reflections	2737 [R(int) = 0.0493]	
Completeness to theta = 67.679°	99.9 %	
Absorption correction	Semi-empirical from equivalents	
Max. and min. transmission	0.5220 and 0.3213	
Refinement method	Full-matrix least-squares on F^2	
Data / restraints / parameters	2737 / 1 / 212	
Goodness-of-fit on F^2	1.029	
Final R indices [I>2sigma(I)]	R1 = 0.0323, wR2 = 0.0846	
R indices (all data)	R1 = 0.0326, wR2 = 0.0847	
Absolute structure parameter	0.02(6)	
Largest diff. peak and hole	0.274 and -0.238 e.Å^{-3}	

Supplementary Table 43. Atomic coordinates (x 10^4) and equivalent isotropic displacement parameters (Å^{2}x 10^3) for Shenvi244. U(eq) is defined as one third of the trace of the orthogonalized U^{ij} tensor.

	x	y	z	U(eq)
F(1)	6355(1)	8319(2)	8337(1)	18(1)
F(3)	5103(1)	5732(2)	9354(1)	17(1)
O(2)	4623(2)	1509(2)	6640(2)	20(1)
O(1)	4709(2)	4140(2)	5771(1)	15(1)
O(5)	592(2)	4856(2)	7197(1)	21(1)
O(4)	2501(2)	6823(2)	5237(1)	18(1)
O(3)	152(2)	5271(2)	4591(1)	23(1)
C(7)	4996(2)	3065(3)	6727(2)	15(1)
C(9)	3196(3)	3446(3)	8378(2)	18(1)
C(12)	1344(3)	5713(3)	5379(2)	17(1)
C(8)	4387(2)	4936(3)	8246(2)	14(1)
C(11)	1816(2)	5218(3)	6663(2)	15(1)
C(14)	3276(2)	6341(3)	7345(2)	14(1)
C(16)	5306(2)	5955(3)	6155(2)	15(1)
C(13)	3890(2)	7015(3)	6322(2)	15(1)
C(2)	7518(2)	7282(3)	8004(2)	17(1)
C(4)	6674(2)	5601(3)	7320(2)	14(1)
C(10)	1786(2)	3463(3)	7225(2)	18(1)
C(15)	2756(3)	7931(3)	7963(2)	18(1)
C(5)	5811(2)	4161(3)	7858(2)	14(1)
C(6)	6939(3)	2924(3)	8791(2)	19(1)
C(1)	8139(3)	8477(3)	7202(2)	21(1)
C(3)	8864(3)	6832(3)	9147(2)	21(1)

Supplementary Table 44. Bond lengths [Å] and angles [°] for Shenvi244.

F(1)-C(2)	1.417(3)	C(6)-H(6A)	0.9800
F(3)-C(8)	1.398(2)	C(6)-H(6B)	0.9800
O(2)-C(7)	1.195(3)	C(6)-H(6C)	0.9800
O(1)-C(7)	1.348(3)	C(1)-H(1A)	0.9800
O(1)-C(16)	1.463(3)	C(1)-H(1B)	0.9800
O(5)-C(11)	1.421(2)	C(1)-H(1C)	0.9800
O(5)-C(10)	1.452(3)	C(3)-H(3A)	0.9800
O(4)-C(12)	1.345(3)	C(3)-H(3B)	0.9800
O(4)-C(13)	1.463(2)	C(3)-H(3C)	0.9800
O(3)-C(12)	1.196(3)		
C(7)-C(5)	1.539(3)	C(7)-O(1)-C(16)	109.20(16)
C(9)-H(9A)	0.9900	C(11)-O(5)-C(10)	61.51(14)
C(9)-H(9B)	0.9900	C(12)-O(4)-C(13)	112.27(16)
C(9)-C(8)	1.551(3)	O(2)-C(7)-O(1)	121.8(2)
C(9)-C(10)	1.516(3)	O(2)-C(7)-C(5)	128.5(2)
C(12)-C(11)	1.498(3)	O(1)-C(7)-C(5)	109.71(18)
C(8)-C(14)	1.582(3)	H(9A)-C(9)-H(9B)	108.8
C(8)-C(5)	1.554(3)	C(8)-C(9)-H(9A)	110.6
C(11)-C(14)	1.512(3)	C(8)-C(9)-H(9B)	110.6
C(11)-C(10)	1.470(3)	C(10)-C(9)-H(9A)	110.6
C(14)-C(13)	1.559(3)	C(10)-C(9)-H(9B)	110.6
C(14)-C(15)	1.533(3)	C(10)-C(9)-C(8)	105.49(17)
C(16)-H(16)	1.0000	O(4)-C(12)-C(11)	107.94(17)
C(16)-C(13)	1.518(3)	O(3)-C(12)-O(4)	123.7(2)
C(16)-C(4)	1.530(3)	O(3)-C(12)-C(11)	128.4(2)
C(13)-H(13)	1.0000	F(3)-C(8)-C(9)	108.23(16)
C(2)-C(4)	1.540(3)	F(3)-C(8)-C(14)	110.61(17)
C(2)-C(1)	1.522(3)	F(3)-C(8)-C(5)	106.58(15)
C(2)-C(3)	1.518(3)	C(9)-C(8)-C(14)	105.25(16)
C(4)-H(4)	1.0000	C(9)-C(8)-C(5)	112.13(18)
C(4)-C(5)	1.553(3)	C(5)-C(8)-C(14)	113.98(16)
C(10)-H(10)	1.0000	O(5)-C(11)-C(12)	120.37(17)
C(15)-H(15A)	0.9800	O(5)-C(11)-C(14)	118.19(17)
C(15)-H(15B)	0.9800	O(5)-C(11)-C(10)	60.27(13)
C(15)-H(15C)	0.9800	C(12)-C(11)-C(14)	108.82(18)
C(5)-C(6)	1.528(3)	C(10)-C(11)-C(12)	130.16(19)

C(10)-C(11)-C(14)	112.09(17)	C(14)-C(15)-H(15B)	109.5
C(11)-C(14)-C(8)	102.77(17)	C(14)-C(15)-H(15C)	109.5
C(11)-C(14)-C(13)	100.86(16)	H(15A)-C(15)-H(15B)	109.5
C(11)-C(14)-C(15)	111.24(16)	H(15A)-C(15)-H(15C)	109.5
C(13)-C(14)-C(8)	117.40(16)	H(15B)-C(15)-H(15C)	109.5
C(15)-C(14)-C(8)	112.81(17)	C(7)-C(5)-C(8)	105.88(15)
C(15)-C(14)-C(13)	110.70(18)	C(7)-C(5)-C(4)	98.46(16)
O(1)-C(16)-H(16)	111.2	C(4)-C(5)-C(8)	113.29(17)
O(1)-C(16)-C(13)	107.55(16)	C(6)-C(5)-C(7)	109.06(18)
O(1)-C(16)-C(4)	102.61(16)	C(6)-C(5)-C(8)	112.64(17)
C(13)-C(16)-H(16)	111.2	C(6)-C(5)-C(4)	115.95(17)
C(13)-C(16)-C(4)	112.87(17)	C(5)-C(6)-H(6A)	109.5
C(4)-C(16)-H(16)	111.2	C(5)-C(6)-H(6B)	109.5
O(4)-C(13)-C(14)	105.90(16)	C(5)-C(6)-H(6C)	109.5
O(4)-C(13)-C(16)	107.39(16)	H(6A)-C(6)-H(6B)	109.5
O(4)-C(13)-H(13)	109.4	H(6A)-C(6)-H(6C)	109.5
C(14)-C(13)-H(13)	109.4	H(6B)-C(6)-H(6C)	109.5
C(16)-C(13)-C(14)	115.07(17)	C(2)-C(1)-H(1A)	109.5
C(16)-C(13)-H(13)	109.4	C(2)-C(1)-H(1B)	109.5
F(1)-C(2)-C(4)	109.40(16)	C(2)-C(1)-H(1C)	109.5
F(1)-C(2)-C(1)	106.30(19)	H(1A)-C(1)-H(1B)	109.5
F(1)-C(2)-C(3)	105.91(17)	H(1A)-C(1)-H(1C)	109.5
C(1)-C(2)-C(4)	109.95(18)	H(1B)-C(1)-H(1C)	109.5
C(3)-C(2)-C(4)	113.19(19)	C(2)-C(3)-H(3A)	109.5
C(3)-C(2)-C(1)	111.77(18)	C(2)-C(3)-H(3B)	109.5
C(16)-C(4)-C(2)	116.01(18)	C(2)-C(3)-H(3C)	109.5
C(16)-C(4)-H(4)	105.7	H(3A)-C(3)-H(3B)	109.5
C(16)-C(4)-C(5)	99.30(16)	H(3A)-C(3)-H(3C)	109.5
C(2)-C(4)-H(4)	105.7	H(3B)-C(3)-H(3C)	109.5
C(2)-C(4)-C(5)	123.11(18)		
C(5)-C(4)-H(4)	105.7		
O(5)-C(10)-C(9)	112.60(18)		
O(5)-C(10)-C(11)	58.22(13)		
O(5)-C(10)-H(10)	120.9		
C(9)-C(10)-H(10)	120.9		
C(11)-C(10)-C(9)	107.18(18)		
C(11)-C(10)-H(10)	120.9		
C(14)-C(15)-H(15A)	109.5		

Symmetry transformations used to generate equivalent atoms:

Supplementary Table 45. Anisotropic displacement parameters ($Å^2$x 10^3) for Shenvi244. The anisotropic displacement factor exponent takes the form: $-2\pi^2[h^2 a^{*2}U^{11} + ... + 2 h k a^* b^* U^{12}]$

	U^{11}	U^{22}	U^{33}	U^{23}	U^{13}	U^{12}
F(1)	9(1)	18(1)	26(1)	-4(1)	4(1)	1(1)
F(3)	13(1)	22(1)	14(1)	-3(1)	0(1)	-1(1)
O(2)	19(1)	15(1)	23(1)	-2(1)	3(1)	0(1)
O(1)	11(1)	17(1)	15(1)	-1(1)	0(1)	1(1)
O(5)	9(1)	25(1)	28(1)	-2(1)	6(1)	-2(1)
O(4)	11(1)	21(1)	18(1)	2(1)	-1(1)	2(1)
O(3)	11(1)	28(1)	22(1)	-2(1)	-6(1)	2(1)
C(7)	9(1)	16(1)	18(1)	0(1)	2(1)	2(1)
C(9)	14(1)	20(1)	20(1)	1(1)	4(1)	-3(1)
C(12)	9(1)	17(1)	21(1)	-1(1)	0(1)	6(1)
C(8)	9(1)	17(1)	13(1)	1(1)	-1(1)	-1(1)
C(11)	5(1)	18(1)	20(1)	-3(1)	1(1)	0(1)
C(14)	6(1)	17(1)	17(1)	0(1)	0(1)	1(1)
C(16)	10(1)	15(1)	17(1)	-1(1)	1(1)	-2(1)
C(13)	8(1)	16(1)	16(1)	1(1)	-2(1)	2(1)
C(2)	7(1)	20(1)	22(1)	-2(1)	2(1)	-1(1)
C(4)	7(1)	17(1)	17(1)	1(1)	2(1)	2(1)
C(10)	10(1)	20(1)	24(1)	-2(1)	4(1)	-3(1)
C(15)	10(1)	20(1)	22(1)	-3(1)	2(1)	0(1)
C(5)	9(1)	15(1)	16(1)	0(1)	0(1)	1(1)
C(6)	12(1)	20(1)	20(1)	4(1)	-1(1)	5(1)
C(1)	10(1)	23(1)	27(1)	1(1)	3(1)	-2(1)
C(3)	9(1)	25(1)	23(1)	-2(1)	-1(1)	-1(1)

Supplementary Table 46. Hydrogen coordinates (x 10^4) and isotropic displacement parameters (Å^{2}x 10^3) for Shenvi244.

	x	y	z	U(eq)
H(9A)	3746	2257	8505	22
H(9B)	2802	3708	9055	22
H(16)	5742	6551	5565	17
H(13)	4202	8315	6449	18
H(4)	7544	4941	7098	17
H(10)	1462	2346	6738	22
H(15A)	2208	7477	8512	27
H(15B)	2000	8705	7370	27
H(15C)	3725	8627	8403	27
H(6A)	6359	1806	8838	28
H(6B)	7265	3529	9562	28
H(6C)	7916	2646	8572	28
H(1A)	7209	8916	6546	31
H(1B)	8874	7782	6888	31
H(1C)	8734	9504	7657	31
H(3A)	9422	7941	9504	31
H(3B)	9654	6014	8978	31
H(3C)	8385	6249	9697	31

Alkene (31) (CCDC 2078168)

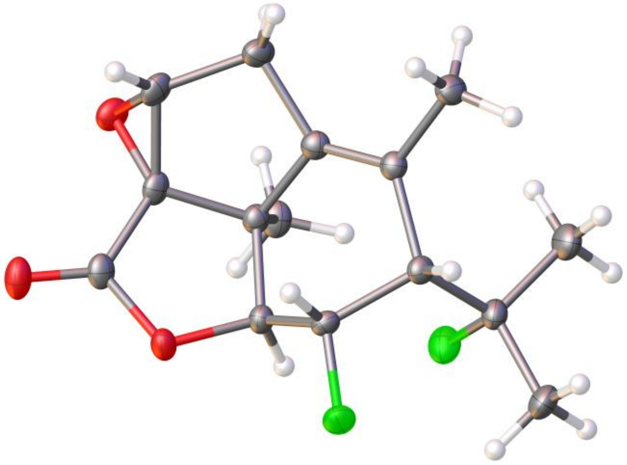

The single crystal X-ray diffraction studies were carried out on a Bruker Smart APEX II CCD diffractometer equipped with Cu K_α radiation (λ =1.54178 Å).

Crystals of the subject compound were used as received. A 0.18 x 0.12 x 0.07 mm piece of a colorless crystal was mounted on a Cryoloop with Paratone oil. Data were collected in a nitrogen gas stream at 100(2) K using ϕ and ϖ scans. Crystal-to-detector distance was 40 mm and exposure time was 5, 10, or 30 seconds depending on the 2θ range per frame using a scan width of 1.25°. Data collection was 97.4 % complete to 67.500° in θ. A total of 9972 reflections were collected. 2478 reflections were found to be symmetry independent, with a R_{int} of 0.0477. Indexing and unit cell refinement indicated a **P**rimitive, **Monoclinic** lattice. The space group was found to be $\boldsymbol{P2_1}$. The data were integrated using the Bruker SAINT Software program and scaled using the SADABS software program. Solution by direct methods (SHELXT) produced a complete phasing model consistent with the proposed structure.

All nonhydrogen atoms were refined anisotropically by full-matrix least-squares (SHELXL-2014). All carbon bonded hydrogen atoms were placed using a riding model. Their positions were constrained relative to their parent atom using the appropriate HFIX command in SHELXL-2014.

Supplementary Table 47. Crystal data and structure refinement for Shenvi242.

Identification code	shenvi242_0m_4_a	
Empirical formula	C15 H18 F2 O3	
Formula weight	284.29	
Temperature	100 K	
Wavelength	1.54178 Å	
Crystal system	Monoclinic	
Space group	P 1 21 1	
Unit cell dimensions	a = 9.3340(2) Å	α= 90°.
	b = 7.3614(2) Å	β= 115.4671(16)°.
	c = 10.8073(3) Å	γ = 90°.
Volume	670.43(3) $Å^3$	
Z	2	
Density (calculated)	1.408 Mg/m^3	
Absorption coefficient	0.975 mm^{-1}	
F(000)	300	
Crystal size	0.18 x 0.12 x 0.07 mm^3	
Theta range for data collection	4.532 to 70.060°.	
Reflections collected	9972	
Independent reflections	2478 [R(int) = 0.0477]	
Completeness to theta = 67.500°	97.4 %	
Absorption correction	Semi-empirical from equivalents	
Refinement method	Full-matrix least-squares on F^2	
Data / restraints / parameters	2478 / 1 / 185	
Goodness-of-fit on F^2	1.062	
Final R indices [I>2sigma(I)]	R1 = 0.0287, wR2 = 0.0736	
R indices (all data)	R1 = 0.0305, wR2 = 0.0743	
Absolute structure parameter	0.03(6)	
Largest diff. peak and hole	0.196 and -0.144 e.$Å^{-3}$	

Supplementary Table 48. Atomic coordinates (x 10^4) and equivalent isotropic displacement parameters ($Å^2$x 10^3) for Shenvi242. U(eq) is defined as one third of the trace of the orthogonalized U^{ij} tensor.

	x	y	z	U(eq)
F(1)	3567(1)	8040(2)	3707(1)	24(1)
F(2)	4092(2)	7722(2)	1000(1)	26(1)
O(1)	9251(2)	7030(3)	7051(2)	27(1)
O(2)	9813(2)	4674(2)	4949(2)	22(1)
O(3)	6988(2)	7801(2)	5251(2)	22(1)
C(1)	8334(2)	6798(3)	5880(2)	20(1)
C(2)	8371(2)	5448(3)	4863(2)	19(1)
C(3)	8618(2)	3471(3)	5039(3)	22(1)
C(4)	7497(2)	2569(3)	3706(3)	23(1)
C(5)	6240(3)	4029(3)	3050(2)	18(1)
C(6)	7058(2)	5858(3)	3468(2)	17(1)
C(7)	7664(3)	6555(4)	2439(2)	23(1)
C(8)	6068(2)	7284(3)	3803(2)	18(1)
C(9)	4475(2)	6556(3)	3635(2)	17(1)
C(10)	3588(2)	5422(3)	2332(2)	17(1)
C(11)	2917(3)	6461(3)	951(2)	20(1)
C(12)	1442(3)	7564(4)	711(3)	30(1)
C(13)	2630(3)	5223(4)	-246(2)	29(1)
C(14)	4670(2)	3807(3)	2457(2)	17(1)
C(15)	3910(3)	1958(3)	2056(3)	22(1)

Supplementary Table 49. Bond lengths [Å] and angles [°] for Shenvi242.

F(1)-C(9)	1.405(2)	C(13)-H(13A)	0.9800
F(2)-C(11)	1.421(3)	C(13)-H(13B)	0.9800
O(1)-C(1)	1.197(3)	C(13)-H(13C)	0.9800
O(2)-C(2)	1.428(2)	C(14)-C(15)	1.509(3)
O(2)-C(3)	1.459(3)	C(15)-H(15A)	0.9800
O(3)-C(1)	1.360(3)	C(15)-H(15B)	0.9800
O(3)-C(8)	1.475(3)	C(15)-H(15C)	0.9800
C(1)-C(2)	1.493(3)		
C(2)-C(3)	1.473(3)	C(2)-O(2)-C(3)	61.34(14)
C(2)-C(6)	1.509(3)	C(1)-O(3)-C(8)	112.42(17)
C(3)-H(3)	1.0000	O(1)-C(1)-O(3)	122.5(2)
C(3)-C(4)	1.522(3)	O(1)-C(1)-C(2)	129.9(2)
C(4)-H(4A)	0.9900	O(3)-C(1)-C(2)	107.58(18)
C(4)-H(4B)	0.9900	O(2)-C(2)-C(1)	122.50(18)
C(4)-C(5)	1.523(3)	O(2)-C(2)-C(3)	60.39(14)
C(5)-C(6)	1.518(3)	O(2)-C(2)-C(6)	118.87(18)
C(5)-C(14)	1.334(3)	C(1)-C(2)-C(6)	109.67(18)
C(6)-C(7)	1.536(3)	C(3)-C(2)-C(1)	127.9(2)
C(6)-C(8)	1.541(3)	C(3)-C(2)-C(6)	109.99(19)
C(7)-H(7A)	0.9800	O(2)-C(3)-C(2)	58.27(13)
C(7)-H(7B)	0.9800	O(2)-C(3)-H(3)	119.8
C(7)-H(7C)	0.9800	O(2)-C(3)-C(4)	115.83(19)
C(8)-H(8)	1.0000	C(2)-C(3)-H(3)	119.8
C(8)-C(9)	1.516(3)	C(2)-C(3)-C(4)	107.64(19)
C(9)-H(9)	1.0000	C(4)-C(3)-H(3)	119.8
C(9)-C(10)	1.538(3)	C(3)-C(4)-H(4A)	111.3
C(10)-H(10)	1.0000	C(3)-C(4)-H(4B)	111.3
C(10)-C(11)	1.550(3)	C(3)-C(4)-C(5)	102.18(19)
C(10)-C(14)	1.528(3)	H(4A)-C(4)-H(4B)	109.2
C(11)-C(12)	1.521(3)	C(5)-C(4)-H(4A)	111.3
C(11)-C(13)	1.509(3)	C(5)-C(4)-H(4B)	111.3
C(12)-H(12A)	0.9800	C(6)-C(5)-C(4)	107.46(17)
C(12)-H(12B)	0.9800	C(14)-C(5)-C(4)	127.2(2)
C(12)-H(12C)	0.9800	C(14)-C(5)-C(6)	124.1(2)

C(2)-C(6)-C(5)	101.99(18)	C(11)-C(12)-H(12A)	109.5
C(2)-C(6)-C(7)	113.35(17)	C(11)-C(12)-H(12B)	109.5
C(2)-C(6)-C(8)	102.28(17)	C(11)-C(12)-H(12C)	109.5
C(5)-C(6)-C(7)	112.07(19)	H(12A)-C(12)-H(12B)	109.5
C(5)-C(6)-C(8)	113.50(17)	H(12A)-C(12)-H(12C)	109.5
C(7)-C(6)-C(8)	112.80(19)	H(12B)-C(12)-H(12C)	109.5
C(6)-C(7)-H(7A)	109.5	C(11)-C(13)-H(13A)	109.5
C(6)-C(7)-H(7B)	109.5	C(11)-C(13)-H(13B)	109.5
C(6)-C(7)-H(7C)	109.5	C(11)-C(13)-H(13C)	109.5
H(7A)-C(7)-H(7B)	109.5	H(13A)-C(13)-H(13B)	109.5
H(7A)-C(7)-H(7C)	109.5	H(13A)-C(13)-H(13C)	109.5
H(7B)-C(7)-H(7C)	109.5	H(13B)-C(13)-H(13C)	109.5
O(3)-C(8)-C(6)	106.89(16)	C(5)-C(14)-C(10)	119.7(2)
O(3)-C(8)-H(8)	109.6	C(5)-C(14)-C(15)	122.1(2)
O(3)-C(8)-C(9)	108.17(17)	C(15)-C(14)-C(10)	117.73(17)
C(6)-C(8)-H(8)	109.6	C(14)-C(15)-H(15A)	109.5
C(9)-C(8)-C(6)	112.79(18)	C(14)-C(15)-H(15B)	109.5
C(9)-C(8)-H(8)	109.6	C(14)-C(15)-H(15C)	109.5
F(1)-C(9)-C(8)	107.60(17)	H(15A)-C(15)-H(15B)	109.5
F(1)-C(9)-H(9)	107.5	H(15A)-C(15)-H(15C)	109.5
F(1)-C(9)-C(10)	111.90(16)	H(15B)-C(15)-H(15C)	109.5
C(8)-C(9)-H(9)	107.5		
C(8)-C(9)-C(10)	114.57(17)		
C(10)-C(9)-H(9)	107.5		
C(9)-C(10)-H(10)	105.7		
C(9)-C(10)-C(11)	116.71(18)		
C(11)-C(10)-H(10)	105.7		
C(14)-C(10)-C(9)	105.45(17)		
C(14)-C(10)-H(10)	105.7		
C(14)-C(10)-C(11)	116.60(17)		
F(2)-C(11)-C(10)	107.51(17)		
F(2)-C(11)-C(12)	106.58(19)		
F(2)-C(11)-C(13)	106.02(19)		
C(12)-C(11)-C(10)	112.90(19)		
C(13)-C(11)-C(10)	112.11(19)		
C(13)-C(11)-C(12)	111.2(2)		

Symmetry transformations used to generate equivalent atoms:

Supplementary Table 50. Anisotropic displacement parameters ($Å^2$x 10^3) for Shenvi242. The anisotropic displacement factor exponent takes the form: $-2\pi^2$[h^2 $a^{*2}U^{11}$ + ... + 2 h k a* b* U^{12}]

	U^{11}	U^{22}	U^{33}	U^{23}	U^{13}	U^{12}
F(1)	18(1)	25(1)	28(1)	-4(1)	9(1)	5(1)
F(2)	26(1)	25(1)	24(1)	5(1)	7(1)	-3(1)
O(1)	17(1)	41(1)	20(1)	-3(1)	4(1)	-3(1)
O(2)	13(1)	25(1)	29(1)	0(1)	9(1)	0(1)
O(3)	16(1)	26(1)	19(1)	-6(1)	4(1)	0(1)
C(1)	13(1)	26(1)	20(1)	-1(1)	7(1)	-4(1)
C(2)	10(1)	25(1)	20(1)	1(1)	6(1)	-1(1)
C(3)	11(1)	26(1)	27(1)	3(1)	7(1)	2(1)
C(4)	16(1)	20(1)	31(1)	-2(1)	8(1)	1(1)
C(5)	17(1)	19(1)	18(1)	0(1)	8(1)	0(1)
C(6)	14(1)	20(1)	18(1)	-1(1)	6(1)	-1(1)
C(7)	20(1)	29(1)	21(1)	1(1)	9(1)	-3(1)
C(8)	14(1)	19(1)	17(1)	-1(1)	4(1)	0(1)
C(9)	16(1)	16(1)	20(1)	1(1)	7(1)	3(1)
C(10)	14(1)	17(1)	20(1)	0(1)	6(1)	-2(1)
C(11)	18(1)	20(1)	18(1)	0(1)	3(1)	0(1)
C(12)	22(1)	29(1)	26(1)	2(1)	0(1)	7(1)
C(13)	31(1)	31(1)	19(1)	1(1)	3(1)	4(1)
C(14)	16(1)	18(1)	16(1)	1(1)	7(1)	0(1)
C(15)	19(1)	19(1)	27(1)	-2(1)	8(1)	-2(1)

Supplementary Table 51. Hydrogen coordinates (x 10^4) and isotropic displacement parameters (Å^{2}x 10^3) for Shenvi242.

	x	y	z	U(eq)
H(3)	8812	2896	5936	26
H(4A)	7030	1446	3881	28
H(4B)	8045	2274	3125	28
H(7A)	8432	5689	2385	35
H(7B)	8176	7738	2743	35
H(7C)	6769	6681	1534	35
H(8)	5903	8375	3205	21
H(9)	4672	5754	4439	21
H(10)	2644	4904	2418	21
H(12A)	1685	8417	1472	44
H(12B)	590	6744	657	44
H(12C)	1103	8242	-149	44
H(13A)	2209	5933	-1096	44
H(13B)	1864	4282	-299	44
H(13C)	3632	4652	-120	44
H(15A)	4411	1291	1561	34
H(15B)	2775	2101	1464	34
H(15C)	4052	1282	2882	34

d_9-34 (CCDC 2087432)

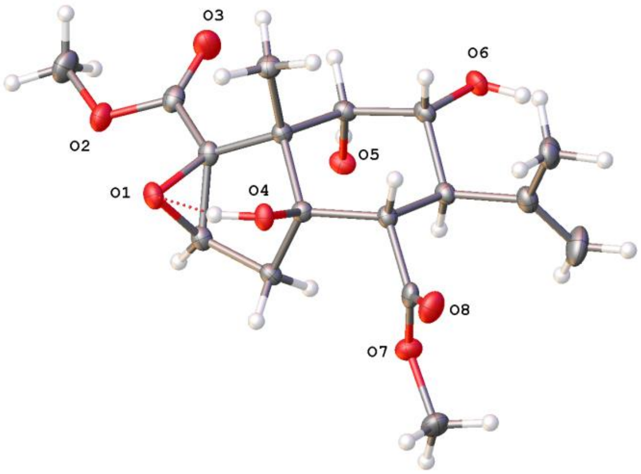

The single crystal X-ray diffraction studies were carried out on a Bruker SMART Pt135 CCD diffractometer equipped with Cu K_{α} radiation (λ = 1.54178). Crystals of the subject compound were used as received. A 0.2 x 0.2 x 0.12 mm colorless block crystal was mounted on a Cryoloop with Paratone oil.

Data were collected in a nitrogen gas stream at 100(2) K using ϕ and ϖ scans. Crystal-to-detector distance was 45 mm using exposure time 2.0, 4.0, and 16.0 second (depending on the 2θ position) with a scan width of 1.40°. Data collection was 98.3% complete to 67.5° in θ. A total of 13976 reflections were collected. 3213 reflections were found to be symmetry independent, with a R_{int} of 0.0419. Indexing and unit cell refinement indicated a **Primitive Monoclinic** lattice. The space group was found to be ***P*2$_1$.** The data were integrated using the Bruker SAINT Software program and scaled using the SADABS software program. Solution by direct methods (SHELXT) produced a complete phasing model consistent with the proposed structure.

All nonhydrogen atoms were refined anisotropically by full-matrix least-squares (SHELXL-2014). All carbon bonded hydrogen atoms were placed using a riding model. Their positions were constrained relative to their parent atom using the appropriate HFIX command in SHELXL-2014.

Supplementary Table 52. Crystal data and structure refinement for Shenvi247_0m.

Identification code	shenvi247_0m	
Empirical formula	C17 H24 O8	
Formula weight	356.36	
Temperature	100.15 K	
Wavelength	1.54178 Å	
Crystal system	Monoclinic	
Space group	P 1 21 1	
Unit cell dimensions	a = 10.7543(6) Å	α= 90°.
	b = 6.2538(3) Å	β= 105.464(3)°.
	c = 13.0275(7) Å	γ = 90°.
Volume	844.45(8) Å^3	
Z	2	
Density (calculated)	1.402 Mg/m^3	
Absorption coefficient	0.942 mm^{-1}	
F(000)	380	
Crystal size	0.2 x 0.2 x 0.1 mm^3	
Theta range for data collection	3.520 to 71.232°.	
Index ranges	-13<=h<=13, -7<=k<=7, -13<=l<=15	
Reflections collected	13976	
Independent reflections	3213 [R(int) = 0.0419]	
Completeness to theta = 67.500°	98.3 %	
Absorption correction	Semi-empirical from equivalents	
Max. and min. transmission	0.7534 and 0.6398	
Refinement method	Full-matrix least-squares on F^2	
Data / restraints / parameters	3213 / 1 / 236	
Goodness-of-fit on F^2	1.061	
Final R indices [I>2sigma(I)]	R1 = 0.0273, wR2 = 0.0698	
R indices (all data)	R1 = 0.0280, wR2 = 0.0703	
Absolute structure parameter	0.06(5)	
Extinction coefficient	0.0042(8)	
Largest diff. peak and hole	0.196 and -0.178 e.Å^{-3}	

Supplementary Table 53. Atomic coordinates (x 10^4) and equivalent isotropic displacement parameters ($Å^2$x 10^3) for Shenvi242. U(eq) is defined as one third of the trace of the orthogonalized U^{ij} tensor.

	x	y	z	U(eq)
F(1)	3567(1)	8040(2)	3707(1)	24(1)
F(2)	4092(2)	7722(2)	1000(1)	26(1)
O(1)	9251(2)	7030(3)	7051(2)	27(1)
O(2)	9813(2)	4674(2)	4949(2)	22(1)
O(3)	6988(2)	7801(2)	5251(2)	22(1)
C(1)	8334(2)	6798(3)	5880(2)	20(1)
C(2)	8371(2)	5448(3)	4863(2)	19(1)
C(3)	8618(2)	3471(3)	5039(3)	22(1)
C(4)	7497(2)	2569(3)	3706(3)	23(1)
C(5)	6240(3)	4029(3)	3050(2)	18(1)
C(6)	7058(2)	5858(3)	3468(2)	17(1)
C(7)	7664(3)	6555(4)	2439(2)	23(1)
C(8)	6068(2)	7284(3)	3803(2)	18(1)
C(9)	4475(2)	6556(3)	3635(2)	17(1)
C(10)	3588(2)	5422(3)	2332(2)	17(1)
C(11)	2917(3)	6461(3)	951(2)	20(1)
C(12)	1442(3)	7564(4)	711(3)	30(1)
C(13)	2630(3)	5223(4)	-246(2)	29(1)
C(14)	4670(2)	3807(3)	2457(2)	17(1)
C(15)	3910(3)	1958(3)	2056(3)	22(1)

Supplementary Table 54. Bond lengths [Å] and angles [°] for Shenvi242.

F(1)-C(9)	1.405(2)	C(13)-H(13A)	0.9800
F(2)-C(11)	1.421(3)	C(13)-H(13B)	0.9800
O(1)-C(1)	1.197(3)	C(13)-H(13C)	0.9800
O(2)-C(2)	1.428(2)	C(14)-C(15)	1.509(3)
O(2)-C(3)	1.459(3)	C(15)-H(15A)	0.9800
O(3)-C(1)	1.360(3)	C(15)-H(15B)	0.9800
O(3)-C(8)	1.475(3)	C(15)-H(15C)	0.9800
C(1)-C(2)	1.493(3)		
C(2)-C(3)	1.473(3)	C(2)-O(2)-C(3)	61.34(14)
C(2)-C(6)	1.509(3)	C(1)-O(3)-C(8)	112.42(17)
C(3)-H(3)	1.0000	O(1)-C(1)-O(3)	122.5(2)
C(3)-C(4)	1.522(3)	O(1)-C(1)-C(2)	129.9(2)
C(4)-H(4A)	0.9900	O(3)-C(1)-C(2)	107.58(18)
C(4)-H(4B)	0.9900	O(2)-C(2)-C(1)	122.50(18)
C(4)-C(5)	1.523(3)	O(2)-C(2)-C(3)	60.39(14)
C(5)-C(6)	1.518(3)	O(2)-C(2)-C(6)	118.87(18)
C(5)-C(14)	1.334(3)	C(1)-C(2)-C(6)	109.67(18)
C(6)-C(7)	1.536(3)	C(3)-C(2)-C(1)	127.9(2)
C(6)-C(8)	1.541(3)	C(3)-C(2)-C(6)	109.99(19)
C(7)-H(7A)	0.9800	O(2)-C(3)-C(2)	58.27(13)
C(7)-H(7B)	0.9800	O(2)-C(3)-H(3)	119.8
C(7)-H(7C)	0.9800	O(2)-C(3)-C(4)	115.83(19)
C(8)-H(8)	1.0000	C(2)-C(3)-H(3)	119.8
C(8)-C(9)	1.516(3)	C(2)-C(3)-C(4)	107.64(19)
C(9)-H(9)	1.0000	C(4)-C(3)-H(3)	119.8
C(9)-C(10)	1.538(3)	C(3)-C(4)-H(4A)	111.3
C(10)-H(10)	1.0000	C(3)-C(4)-H(4B)	111.3
C(10)-C(11)	1.550(3)	C(3)-C(4)-C(5)	102.18(19)
C(10)-C(14)	1.528(3)	H(4A)-C(4)-H(4B)	109.2
C(11)-C(12)	1.521(3)	C(5)-C(4)-H(4A)	111.3
C(11)-C(13)	1.509(3)	C(5)-C(4)-H(4B)	111.3
C(12)-H(12A)	0.9800	C(6)-C(5)-C(4)	107.46(17)
C(12)-H(12B)	0.9800	C(14)-C(5)-C(4)	127.2(2)
C(12)-H(12C)	0.9800	C(14)-C(5)-C(6)	124.1(2)

C(2)-C(6)-C(5)	101.99(18)	C(11)-C(12)-H(12A)	109.5
C(2)-C(6)-C(7)	113.35(17)	C(11)-C(12)-H(12B)	109.5
C(2)-C(6)-C(8)	102.28(17)	C(11)-C(12)-H(12C)	109.5
C(5)-C(6)-C(7)	112.07(19)	H(12A)-C(12)-H(12B)	109.5
C(5)-C(6)-C(8)	113.50(17)	H(12A)-C(12)-H(12C)	109.5
C(7)-C(6)-C(8)	112.80(19)	H(12B)-C(12)-H(12C)	109.5
C(6)-C(7)-H(7A)	109.5	C(11)-C(13)-H(13A)	109.5
C(6)-C(7)-H(7B)	109.5	C(11)-C(13)-H(13B)	109.5
C(6)-C(7)-H(7C)	109.5	C(11)-C(13)-H(13C)	109.5
H(7A)-C(7)-H(7B)	109.5	H(13A)-C(13)-H(13B)	109.5
H(7A)-C(7)-H(7C)	109.5	H(13A)-C(13)-H(13C)	109.5
H(7B)-C(7)-H(7C)	109.5	H(13B)-C(13)-H(13C)	109.5
O(3)-C(8)-C(6)	106.89(16)	C(5)-C(14)-C(10)	119.7(2)
O(3)-C(8)-H(8)	109.6	C(5)-C(14)-C(15)	122.1(2)
O(3)-C(8)-C(9)	108.17(17)	C(15)-C(14)-C(10)	117.73(17)
C(6)-C(8)-H(8)	109.6	C(14)-C(15)-H(15A)	109.5
C(9)-C(8)-C(6)	112.79(18)	C(14)-C(15)-H(15B)	109.5
C(9)-C(8)-H(8)	109.6	C(14)-C(15)-H(15C)	109.5
F(1)-C(9)-C(8)	107.60(17)	H(15A)-C(15)-H(15B)	109.5
F(1)-C(9)-H(9)	107.5	H(15A)-C(15)-H(15C)	109.5
F(1)-C(9)-C(10)	111.90(16)	H(15B)-C(15)-H(15C)	109.5
C(8)-C(9)-H(9)	107.5		
C(8)-C(9)-C(10)	114.57(17)		
C(10)-C(9)-H(9)	107.5		
C(9)-C(10)-H(10)	105.7		
C(9)-C(10)-C(11)	116.71(18)		
C(11)-C(10)-H(10)	105.7		
C(14)-C(10)-C(9)	105.45(17)		
C(14)-C(10)-H(10)	105.7		
C(14)-C(10)-C(11)	116.60(17)		
F(2)-C(11)-C(10)	107.51(17)		
F(2)-C(11)-C(12)	106.58(19)		
F(2)-C(11)-C(13)	106.02(19)		
C(12)-C(11)-C(10)	112.90(19)		
C(13)-C(11)-C(10)	112.11(19)		
C(13)-C(11)-C(12)	111.2(2)		

Symmetry transformations used to generate equivalent atoms:

Supplementary Table 55. Anisotropic displacement parameters ($Å^2$x 10^3) for Shenvi242. The anisotropic displacement factor exponent takes the form: $-2\pi^2$[h^2 $a^{*2}U^{11}$ + ... + 2 h k a* b* U^{12}]

	U^{11}	U^{22}	U^{33}	U^{23}	U^{13}	U^{12}
F(1)	18(1)	25(1)	28(1)	-4(1)	9(1)	5(1)
F(2)	26(1)	25(1)	24(1)	5(1)	7(1)	-3(1)
O(1)	17(1)	41(1)	20(1)	-3(1)	4(1)	-3(1)
O(2)	13(1)	25(1)	29(1)	0(1)	9(1)	0(1)
O(3)	16(1)	26(1)	19(1)	-6(1)	4(1)	0(1)
C(1)	13(1)	26(1)	20(1)	-1(1)	7(1)	-4(1)
C(2)	10(1)	25(1)	20(1)	1(1)	6(1)	-1(1)
C(3)	11(1)	26(1)	27(1)	3(1)	7(1)	2(1)
C(4)	16(1)	20(1)	31(1)	-2(1)	8(1)	1(1)
C(5)	17(1)	19(1)	18(1)	0(1)	8(1)	0(1)
C(6)	14(1)	20(1)	18(1)	-1(1)	6(1)	-1(1)
C(7)	20(1)	29(1)	21(1)	1(1)	9(1)	-3(1)
C(8)	14(1)	19(1)	17(1)	-1(1)	4(1)	0(1)
C(9)	16(1)	16(1)	20(1)	1(1)	7(1)	3(1)
C(10)	14(1)	17(1)	20(1)	0(1)	6(1)	-2(1)
C(11)	18(1)	20(1)	18(1)	0(1)	3(1)	0(1)
C(12)	22(1)	29(1)	26(1)	2(1)	0(1)	7(1)
C(13)	31(1)	31(1)	19(1)	1(1)	3(1)	4(1)
C(14)	16(1)	18(1)	16(1)	1(1)	7(1)	0(1)
C(15)	19(1)	19(1)	27(1)	-2(1)	8(1)	-2(1)

Supplementary Table 56. Hydrogen coordinates (x 10^4) and isotropic displacement parameters (Å^{2}x 10 ^{3}) for Shenvi242.

	x	y	z	U(eq)
H(3)	8812	2896	5936	26
H(4A)	7030	1446	3881	28
H(4B)	8045	2274	3125	28
H(7A)	8432	5689	2385	35
H(7B)	8176	7738	2743	35
H(7C)	6769	6681	1534	35
H(8)	5903	8375	3205	21
H(9)	4672	5754	4439	21
H(10)	2644	4904	2418	21
H(12A)	1685	8417	1472	44
H(12B)	590	6744	657	44
H(12C)	1103	8242	-149	44
H(13A)	2209	5933	-1096	44
H(13B)	1864	4282	-299	44
H(13C)	3632	4652	-120	44
H(15A)	4411	1291	1561	34
H(15B)	2775	2101	1464	34
H(15C)	4052	1282	2882	34

10. References

1. Crossley, S. W. M.; Tong, G.; Lambrecht, M.; Burdge, H. E.; Shenvi, R. A. Synthesis of (–) Picrotoxinin by Late-Stage Strong Bond Activation. *J. Am. Chem. Soc.* **142**, 11376–11381 (2020).
2. Dao, H. T.; Li, C.; Michaudel, Q.; Maxwell, B. D.; Baran, P. S. Hydromethylation of Unactivated Olefins. *J. Am. Chem. Soc.* **137**, 8046–8049 (2015).
3. Krische, M. J.; Trost, B. M. Transformations of the Picrotoxanes: The Synthesis of Corianin and Structural Analogues from Picrotoxinin. *Tetrahedron* **54**, 7109–7120 (1998).
4. Zhao, W.; Ye, Q.; Dai, J.; Martin, M.-T.; Zhu, J. *allo*-Aromadendrane- and Picrotoxane-Type Sesquiterpenes from *Dendrobium moniliforme*. *Planta Med.* **69**, 1136–1140 (2003).
5. Shirai, Y.; Hosie, A. M.; Buckingham, S. D.; Holyoke, C. W.; Baylis, H. A.; Sattelle, D. B. Actions of Picrotoxinin Analogues on an Expressed, Homo-Oligormeric GABA Receptor of *Drosophila melanogaster*. *Neurosci. Lett.* **189**, 1–4 (1995). 6FPXN was first reported in this paper, however, no characterization data of compound could be found.
6. Krische, M. J.; Trost, B. M. Total Synthesis of Methyl Picrotoxate via the Palladium Catalyzed Enyne Cycloisomerization Reaction. *Tetrahedron* **54**, 3693–3704 (1998).
7. Agarwal, S. K.; Singh, S. S.; Verma, S.; Kumar, S. Two Picrotoxin Derivatives from *Anamirta cocculus*. *Phytochemistry* **50**, 1365–1368 (1999).
8. Tong, G.; Shenvi, R. A. Revision of the Unstable Picrotoxinin Hydrolysis Product. *Angew. Chem. Int. Ed.* **60**, 19113–19116 (2021).
9. Schwartz, R. D.; Mindlin, M. C. Inhibition of the GABA Receptor-Gated Chloride Ion Channel in Brain by Noncompetitive Inhibitors of the Nicotinic Receptor-Gated Cation Channel. *J. Pharmacol. Exp. Ther.* **244**, 963–970 (1987).
10. Ref. 1 estimated an IC_{50} of 9 μM, but this value has been refined.
11. Frisch, M. J. Trucks, G. W.; Schlegel, H. B.; Scuseria, G. E.; Robb, M. A.; Cheeseman, J. R.; Scalmani, G.; Barone, V.; Petersson, G. A.; Nakatsuji, H.; Li, X.; Caricato, M.; Marenich, A. V.; Bloino, J.; Janesko, B. G.; Gomperts, R.; Mennucci, B.; Hratchian, H. P.; Ortiz, J. V.; Izmaylov, A. F.; Sonnenberg, J. L.; Williams-Young, D.; Ding, F.; Lipparini, F.; Egidi, F.; Goings, J.; Peng, B.; Petrone, A.; Henderson, T.; Ranasinghe, D.; Zakrzewski, V. G.; Gao, J.; Rega, N.; Zheng, G.; Liang, W.; Hada, M.; Ehara, M.; Toyota, K.; Fukuda, R.; Hasegawa, J.; Ishida, M.; Nakajima, T.; Honda, Y.; Kitao, O.; Nakai, H.; Vreven, T.; Throssell, K.; Montgomery, Jr., J. A.; Peralta, J. E.; Ogliaro, F.; Bearpark, M. J.; Heyd, J. J.; Brothers, E. N.; Kudin, K. N.; Staroverov, V. N.; Keith, T. A.; Kobayashi, R.; Normand, J.; Raghavachari, K.; Rendell, A. P.; Burant, J. C.; Iyengar, S. S.; Tomasi, J.; Cossi, M.; Millam, J. M.; Klene, M.; Adamo, C.; Cammi, R.; Ochterski, J. W.; Martin, R. L.; Morokuma, K.; Farkas, O.; Foresman, J. B.; Fox, D. J. Gaussian 16, Revision A.03. Gaussian, Inc., Wallingford CT, (2019).
12. Chai, J.-D.; Head-Gordon, M. Long-Range Corrected Hybrid Density Functionals with Damped Atom–Atom Dispersion Corrections. *Phys. Chem. Chem. Phys.* **10**, 6615–6620 (2008).
13. Grimme, S. Supramolecular Binding Thermodynamics by Dispersion-Corrected Density Functional Theory. *Chem. Eur. J.* **18**, 9955–9964 (2012).
14. Luchini, G.; Alegre-Requena, J. V.; Funes-Ardoiz, I.; Paton, R. S. GoodVibes: Automated Thermochemistry for Heterogeneous Computational Chemistry Data. *F1000Research* **9**, 291. (2020)
15. Marenich, A. V.; Cramer, C. J.; Truhlar, D. G. Universal Solvation Model Based on Solute Electron Density and on a Continuum Model of the Solvent Defined by the Bulk Dielectric Constant and Atomic Surface Tensions. *J. Phy. Chem. B* **113**, 6378–6396 (2009).
16. Legault, C. Y. CYLview, 1.0b; Université de Sherbrooke (2009). http://www.cylview.org.
17. Spartan ’20, Wavefunction, Inc.
18. Ryu, H.; Park, J.; Kim, H. K.; Park, J. Y.; Kim, S.-T.; Baik, M.-H. Pitfalls in Computational Modeling of Chemical Reactions and How to Avoid Them. *Organometallics* **37**, 3228−3239 (2018).
19. Kim, J. J.; Gharpure, A.; Teng, J.; Zhuange, Y.; Howard, R. J.; Zhu, S.; Noviello, C. M.; Walsh Jr., R. M.; Lindahl, E. Shared Structural Mechanisms of General Anaesthetics and Benzodiazepines. *Nature* **585**, 303–308 (2020).

20. *Molecular Operating Environment (MOE)*, 2020.09 Chemical Computing Group ULC, 1010 Sherbooke St. West, Suite #910, Montreal, QC, Canada, H3A 2R7 (2022).
21. Maier, J. A.; Martinez, C.; Kasavajhala, K.; Wickstrom, L.; Hauser, K. E.; Simmerling, C. ff14SB: Improving the Accuracy of Protein Side Chain and Backbone Parameters from ff99SB. *J. Chem. Theory Comput.* **11**, 3696–3713 (2015).
22. Case, D. A.; Aktulga, H. M.; Belfon, K.; Ben-Shalom, I. Y.; Brozell, S. R.; Cerutti, D. S.; Cheatham, T. E.; III, Cisneros, G. A.; Cruzeiro, V. W. D.; Darden, T. A.; Duke, R. E.; Giambasu, G.; Gilson, M. K.; Gohlke, H.; Goetz, A. W.; Harris, R.; Izadi, S.; Izmailov, S. A.; Jin, C.; Kasavajhala, K.; Kaymak, M. C.; King, E.; Kovalenko, A.; Kurtzman, T.; Lee, T. S.; LeGrand, S.; Li, P.; Lin, C.; Liu, J.; Luchko, T.; Luo, R.; Machado, M.; Man, V.; Manathunga, M.; Merz, K. M.; Miao, Y.; Mikhailovskii, O.; Monard, G.; Nguyen, H.; O'Hearn, K. A.; Onufriev, A.; Pan, F.; Pantano, S.; Qi, R.; Rahnamoun, A.; Roe, D. R.; Roitberg, A.; Sagui, C.; Schott-Verdugo, S.; Shen, J.; Simmerling, C. L.; Skrynnikov, N. R.; Smith, J.; Swails, J.; Walker, R. C.; Wang, J.; Wei, H., Wolf, R. M.; Wu, X.; Xue, Y.; York, D. M.; Zhao, S.; Kollman P. A. Amber 2021, University of California, San Francisco (2021).
